# Construction of azaheterocycles via Pd-catalyzed migratory cycloannulation reaction of unactivated alkenes

**DOI:** 10.1038/s41467-022-32726-x

**Published:** 2022-08-27

**Authors:** Jin-Ping Wang, Shuo Song, Yichen Wu, Peng Wang

**Affiliations:** 1 State Key Laboratory of Organometallic Chemistry, Shanghai Institute of Organic Chemistry, University of Chinese Academy of Sciences, CAS 345 Lingling Road, Shanghai, 200032 PR China; 2 CAS Key Laboratory of Energy Regulation Materials, Shanghai Institute of Organic Chemistry, CAS 345 Lingling Road, Shanghai, 200032 PR China; 3 School of Chemistry and Materials Science, Hangzhou Institute for Advanced Study, University of Chinese Academy of Sciences, 1 Sub-lane Xiangshan, Hangzhou, 310024 PR China

**Keywords:** Synthetic chemistry methodology, Homogeneous catalysis

## Abstract

Azahetereocycles constitute important structural components in many biologically active natural compounds and marketed drugs, and represent the most promising scaffolds in drug discovery. Accordingly, the development of efficient and general synthetic methods for the construction of diverse azaheterocycles is the major goal in synthetic chemistry. Herein, we report the efficient construction of a wide range of azaheterocycles via a Pd-catalyzed migratory cycloannulation strategy with unactivated alkenes. This strategy enables the rapid synthesis of a series of 6-, 7- and 8-membered azaheterocycles in high efficiency, and features a broad substrate scope, excellent functional group tolerance under redox-neutral conditions. The significance of this finding is demonstrated by the efficient synthesis of drug-like molecules with high step-economy. Preliminary mechanistic investigations reveal that this reaction underwent a sequentially migratory insertion to alkenes, metal migration process, and the aza-Michael addition to a quinone methide intermediate.

## Abstract (web summary)

Azahetereocycles represent promising scaffolds in drug discovery. Here, the authors report a Pd-catalyzed migratory cycloannulation strategy for efficient construction of a wide range of azahetereocycles from unactivated aliphatic alkenes.

## Introduction

**Fig. 1:**
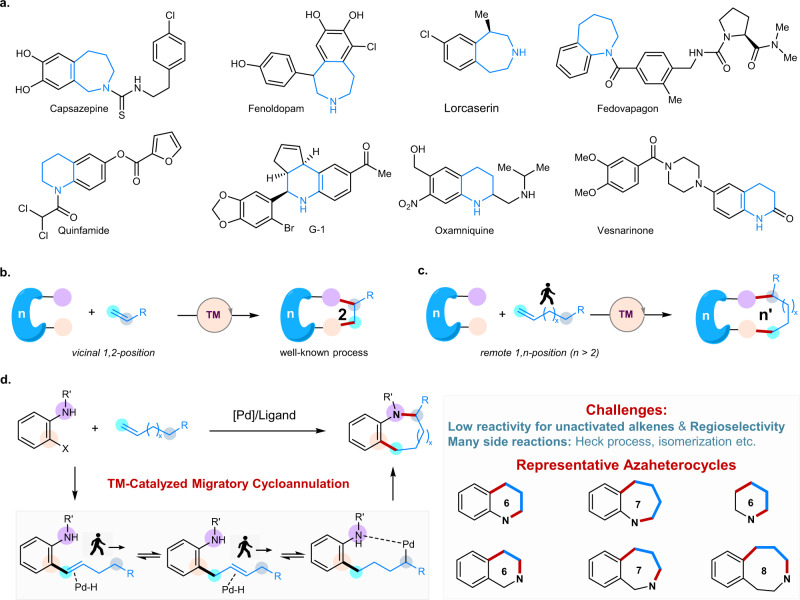
Pd-Catalyzed migratory cycloannulation reaction. **a** Bioactive azaheterocycle containing drugs and natural products. **b** Transition metal-catalyzed [*n* + 2] cycloannulation with alkenes. **c** Transition metal-catalyzed migratory cycloannulation with unactivated alkenes. **d** Pd-Catalyzed migratory cycloannulation with alkenes for the synthesis of azaheterocycles.

The discovery of powerful synthetic methodology to access high-value azaheterocycles has been at the forefront of synthetic organic chemistry for more than a century, as 58% of FDA-approved small molecule drugs contain at least one azaheterocycle^1–3^ (Fig. 1a). Accordingly, great research efforts have been made to develop efficient synthetic methods for the construction of azaheterocycle-containing molecules^4–13^. Notably, the Larock-type annulation^14–31^, transition metal-catalyzed cycloannulation of ambiphilic arylhalides with carbon-carbon double bonds, represents one of the most widely used and efficient methods^14–19^ since the early contributions by Dieck^20^ and Larock^21–31^. Numerous [*n* + 2] reactions for the synthesis of 5-, 6-membered azaheterocycles have been reported with highly reactive styrene, 1,3-diene, allene, and strained cyclic alkene, in which the cyclization normally occurred at the vicinal 1,2-position of those alkenes (Fig. 1b). In sharp contrast, the transition metal-catalyzed migratory cycloannulation, the cyclization happens at the remote (1,*n*)-position of the unactivated alkenes, is barely mentioned in the literatures^32,33^ (Fig. 1c). Overcoming the limitation of the vicinal 1,2-functionalization of alkenes in transition metal-catalyzed [*n* + 2] cyclisation reactions will open an avenue for the rapid construction of diverse azaheterocycles, and will find wide synthetic applications in pharmaceutical industry. In general, this approach will allow the synthesis of various hetero- or carbocycles starting from the commercially available unactivated alkenes with different ambiphilic synthetic blocks by precisely controlling the regioselectivity in the migratory process of alkenes.

Recently, the transition metal-catalyzed migratory hydrofunctionalization or difunctionalization of alkenes has emerged as an attractive approach to enrich the molecular complexity, and has extended synthetic chemists’ toolbox^34–50^. Inspired by the development of intermolecular three-component migratory difunctionalization reactions^45–50^, we envisioned that the transition metal-catalyzed migratory cycloannulation could become a general approach for the construction of various ring-sized azaheterocycles starting from the ambiphilic coupling partners and unactivated alkenes. Mechanistically, transition metal-catalyzed migratory cycloannulation will undergo the sequentially oxidative addition of arylhalide, migratory addition of unactivated alkene, chain-walking process, and cyclization (Fig. 1d). However, this process remains a paramount challenge due to the following reasons: 1) the low reactivity of the unactivated nonconjugated alkenes, 2) difficulty in controlling the cyclization position along the carbon chain of the alkenes during the metal migratory process, and 3) challenges for inhibiting various predictable side reactions (isomerization of aliphatic alkenes, Heck-type or reductive Heck-type side reactions).

Herein, we report our efforts on developing a general and efficient approach for the construction of azaheterocycles via the Pd-catalyzed migratory cycloannulation of unactivated alkenes. The key to rendering the reactivity of the unactivated alkenes and to controlling the ring size (the cyclization position along the carbon chain of the alkenes) of the azaheterocycles is the use of a hydroxyl group, which enables the formation of a quinone methide intermediate after the metal migratory process. The methodology is capable of the efficient construction of diverse azaheterocycles with various ring sizes (6–8 membered azaheterocycles), including tetrahydroquinoline, tetrahydroisoquinoline, tetrahydrobenzo[b]azepine, tetrahydrobenzo[c]azepine, tetrahydrobenzo[d]azepine, hexahydrobenzo[d]azocine, piperidine etc., which are privileged scaffolds in natural products and pharmaceuticals. Moreover, this method is applicable for the efficient synthesis of azaheterocycle-containing complex bioactive molecules.

## Results and discussion

### Reaction optimization

**Fig. 2:**
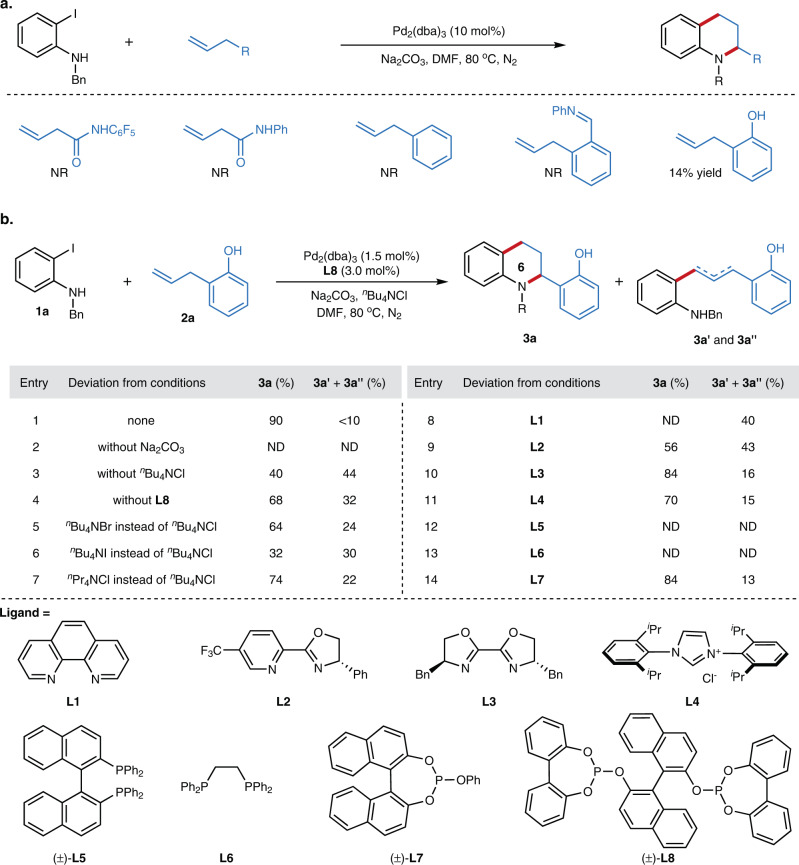
Reaction parameters evaluation for Pd-catalyzed migratory cycloannulation of unactivated alkenes. **a** Pd-catalyzed migratory cycloannulation of unactivated alkenes with different unactivated alkenes. **b** Reaction parameters evaluation. Bu, butyl; Bn, benzyl; Pr, propyl; Ph, phenyl; DMF, *N*,*N*-dimethylformamide; dba, dibenzylideneacetone; NR, no reaction; ND, not detected. The yield was determined by analysis of the crude ^1^H NMR using dibromomethane as the internal standard. See Supplementary Information for experimental details.

With the concept in mind, we investigated the intramolecular cycloannulation with various unactivated alkenes with *N*-benzyl-2-iodoaniline **1a**. Unfortunately, no reaction happened for simple unactivated alkenes, such as but-3-enamide, allylbenzene (Fig. 2a). Inspiring by the directing group approach which could render the reactivity of unactivated alkenes and precisely control the regioselectivity in the alkene functionalization^51^, we next tested the allylbenzene bearing an *ortho*-imine or hydroxyl directing group. We are pleased to find the desired migratory cycloannulation product could be formed in 14% yield, along with isomerization and oxidative Heck-type byproducts, in the presence of Pd_2_(dba)_3_, and Na_2_CO_3_ in *N,N-*dimethylformamide (DMF). After systematically evaluating the reaction parameters, the yield was improved to 90% in the presence of Pd_2_(dba)_3_ (1.5 mol%), BINOL-derived bisphosphite ligand **L8** (3.0 mol%), Na_2_CO_3_ and ^*n*^Bu_4_NCl in DMF. Control experiments indicate all reaction parameters are essential for this highly efficient migratory cycloannulation reaction (Fig. 2b). The reaction didn’t happen in the absence of base. Significant ligand effect has been observed for this reaction which could inhibit the formation of the Heck-type byproducts. 68% yield of tetrahydroquinoline **3a** was obtained with 32% Heck-type byproducts without ligand. Notably, the addition of ^*n*^Bu_4_NCl was also indispensable, leading to significant increase of the efficiency. Generally, the ^*n*^Bu_4_NCl gave better result in comparison with ^*n*^Bu_4_NBr or ^*n*^Bu_4_NI. The crucial anion effects might be accounted from the strong coordination ability of chloride, which could facilitate the oxidative addition of aryl halide and stabilize the Pd intermediate during the reaction process. Given the importance of ligand in transition metal catalysis, we also investigated the ligand effect for this reaction. 1,10-phenanthroline ligand (**L1**) gave the Heck byproducts in 40% yield without the desired product. Pyridine-oxazoline ligand (**L2**) led to inferior results, providing the targeted compound in 56% yield, while NHC ligand (**L4**) showed similar outcome (70% yield) comparing to the data without ligand. Di-phosphine ligand, BINAP (**L5**) and DIPPE (**L6**), were inert under our conditions, providing the olefin isomerization byproducts in high yields. Monophosphite ligand **L7** showed similar reactivity as bis-oxazoline ligand (**L3**), providing the desired product **3a** in 84% yield. Overall, the BINOL-derived bisphosphite ligand **L8** proved to be the optimal ligand, accelerating this migratory cycloannulation in maximum extent. Given the Heck-type byproducts were inhibited with the optimal ligand, we hypothesized that the ligand could stabilize the Pd-H intermediates during the migratory process, which further enhance the reaction outcomes.

### Mechanistic insights

**Fig. 3:**
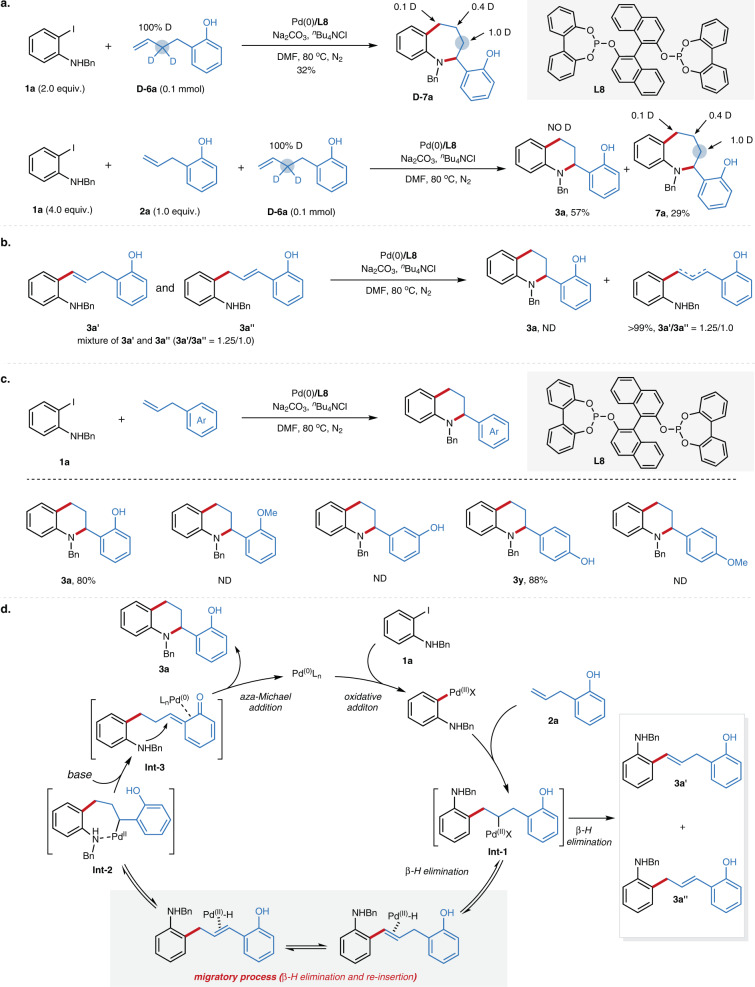
Mechanistic studies and proposed mechanism. **a** Deuterium experiments. **b** Transformation of isolated Heck-product. **c** Control experiments **d** Proposed mechanism. Ar, aryl; Bn, benzyl; L_n_, ligand; Me, methyl; DMF, *N*,*N*-dimethylformamide; ND, not detected.

To gain mechanistic insights of this reaction, we performed deuterium-labeling experiments using deuterium-labeled terminal alkene **D-6a** (Fig. 3a). Treatment of the alkene **D-6a** with *N*-benzyl-2-iodoaniline **1a** led to the formation of desired product in 32% yield with a deuterium distribution at the various positions of aliphatic cycle, which indicates the metal walking event via a *β*-H elimination and reinsertion process. The reaction of **2a** and deuterium-labeled terminal alkene **D-6a** with *N*-benzyl-2-iodoaniline gave undeuterated **3a** and deuterated **7a** in 57 and 29% yield, respectively. The lack of H/D scrambling between **3a** and **7a** might unveil the Pd(II)-H could not dissociate from the alkene during the migration. The isolated Heck-products **3a’** and **3a”** could not transform to the cyclic product under the standard conditions, which further confirm this hypothesis (Fig. 3b). To elucidate the role of the *ortho*-hydroxyl group, detailed control experiments were next carried out. Replacement of the hydroxy group with methoxy group resulted in no reaction, and the *meta*-hydroxyl group led to no reaction as well. Interestingly, the *para*-hydroxyl group showed similar reactivity as the *ortho*-hydroxyl group, which indicates the hydroxyl group might not serve as a directing group in our migratory cycloannulation reaction (Fig. 3c). Given the formation of a quinone methide intermediate^52,53^ might happen in the Pd-catalyzed functionalization of α-hydroxy styrene^54^ pioneered by Sigman, we hypothesized that our reaction might proceed under similar reaction process, in which the azaheterocycle was formed via an aza-Michael addition to the corresponding quinone methide intermediates. The absence of chiral induction with various chiral ligands also supports this hypothesis as the chiral carbon center was generated by the aza-Michael addition rather than the Pd-catalyzed carbon-nitrogen formation. Based on the aforementioned mechanism experiments, a proposed mechanism was depicted in Fig. 3d. The oxidative addition of *N*-benzyl-2-iodoaniline with Pd(0) led to the formation of aryl-Pd(II) intermediate, which underwent the migratory insertion with alkene led to the alkyl-Pd(II) intermediate **Int-1**. The palladium could migrate to the *α*-position of the phenol side via a rapid *β*-hydrogen elimination and reinsertion process (chain-walking process), followed by formation of a quinone methide intermediate **Int-3** in the presence of a base with concomitant reduction of Pd(II). Finally, the intramolecular aza-Michael addition to quinone methide intermediate provided the desired products.

### Substrate scope

**Fig. 4:**
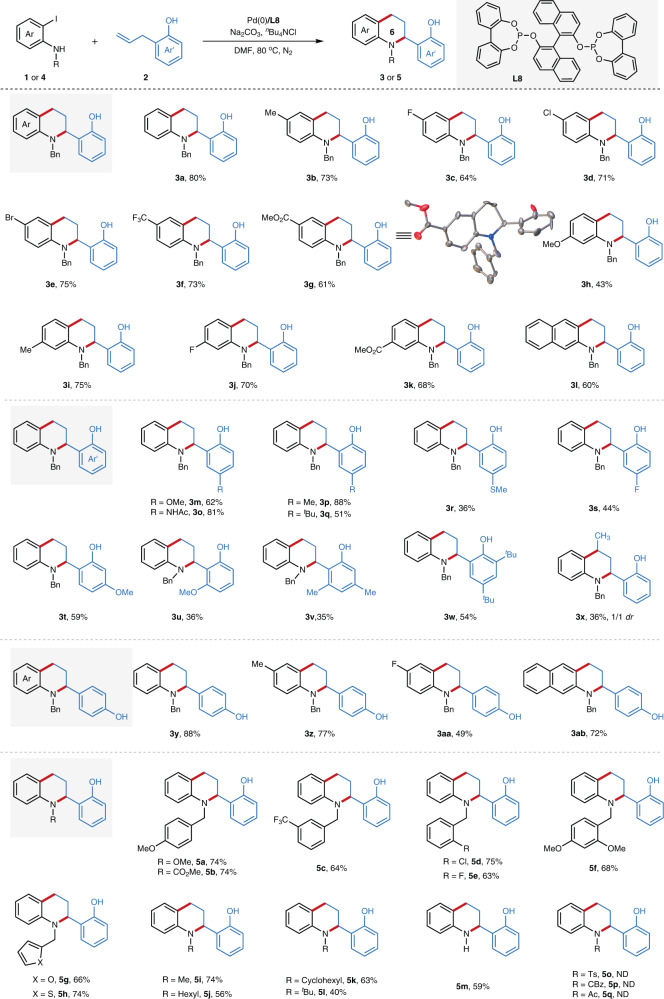
The synthesis of tetrahydroquinoline derivatives via Pd-catalyzed migratory cycloannulation. The values under each structure indicate isolated yields (See Supplementary Information for experimental details). Reaction conditions: **1** (0.4 mmol), **2** (0.2 mmol), Pd_2_(dba)_3_ (2.8 mg, 1.5 mol%), **L8** (4.3 mg, 3.0 mol%), Na_2_CO_3_ (53.0 mg, 0.5 mmol), ^*n*^Bu_4_NCl (111.2 mg, 0.4 mmol), DMF (3.0 mL), 80 °C, 18 h. For **5i**, the reaction was conducted for 24 h. Bn, benzyl; Me, methyl; ^*t*^Bu, *tert*-butyl; Ar, aryl; Ac, acetyl; Ts, 4-toluolsulfonyl; Cbz, benzyloxycarbonyl; DMF, *N*,*N*-dimethylformamide; ND, not detected., *dr*, diastereomeric ratio.

After understanding the reaction mechanism, we set out to evaluate the substrate scope regarding the 2-iodoaniline derivatives under the optimal conditions. As summarized in Fig. 4, the scope of this reaction is very broad, providing corresponding tetrahydroquinoline in high yields. Good functional group compatibility was observed with the tolerance of methyl (**3b**, **3i**), methoxy (**3h**), fluoro (**3c**, **3j**), chloro (**3d**), bromo (**3e**), trifluoromethyl (**3f**), ester (**3g**, **3k**). It is noteworthy that the bromo functionality (**3e**), which is commonly incompatible with Pd-catalyzed coupling reaction, was well tolerated in this reaction. *N-*benzyl-3-iodonaphthalen-2-amine was also suitable ambiphilic partner for this migratory cycloannulation reaction, giving the desired product (**3l**) in 60% yield. We next checked the breadth of the 2-allylphenol derivatives, which also showed high level of functional group tolerance (**3m-x**). For instance, acetylamino (**3o**) and methylthio (**3r**) containing substrates proceeded in 81 and 36% yields, respectively. 6-Substituted 2-allylphenols are also suitable substrates, albeit with lower yields probably due to the steric hindrance (**3u-v**). The allylbenzene bearing a *para*-hydroxyl group (4-allylphenol) is also well tolerated, providing the desired tetrahydroquinoline in moderate to good yields (**3y-3ab**). Pleasingly, internal unactivated alkene could also be employed in this reaction, albeit moderate yield was obtained with an inferior diastereoselectivity (**3x**, 1/1 dr). The electron-rich and electron-deficient benzyl substituents on nitrogen of 2-iodoaniline are well tolerated, providing corresponding tetrahydroquinoline derivatives **5a-h** in moderate to good yields. And aliphatic alkyl groups (**5i**-**5l**) including methyl, hexyl, cyclohexyl, even bulky *tert*-butyl group are all compatible, which further underscores the generality of this methodology. Notably, protecting group free substrate (2-iodoaniline) gave the desired tetrahydroquinoline **5m** in 59% yield. The 2-iodoanilines with electron-withdrawing protecting group (such as Ts, Cbz, and Ac etc.) on nitrogen of (**5o**-**5p**) could not be tolerated, resulting in no reaction.

**Fig. 5:**
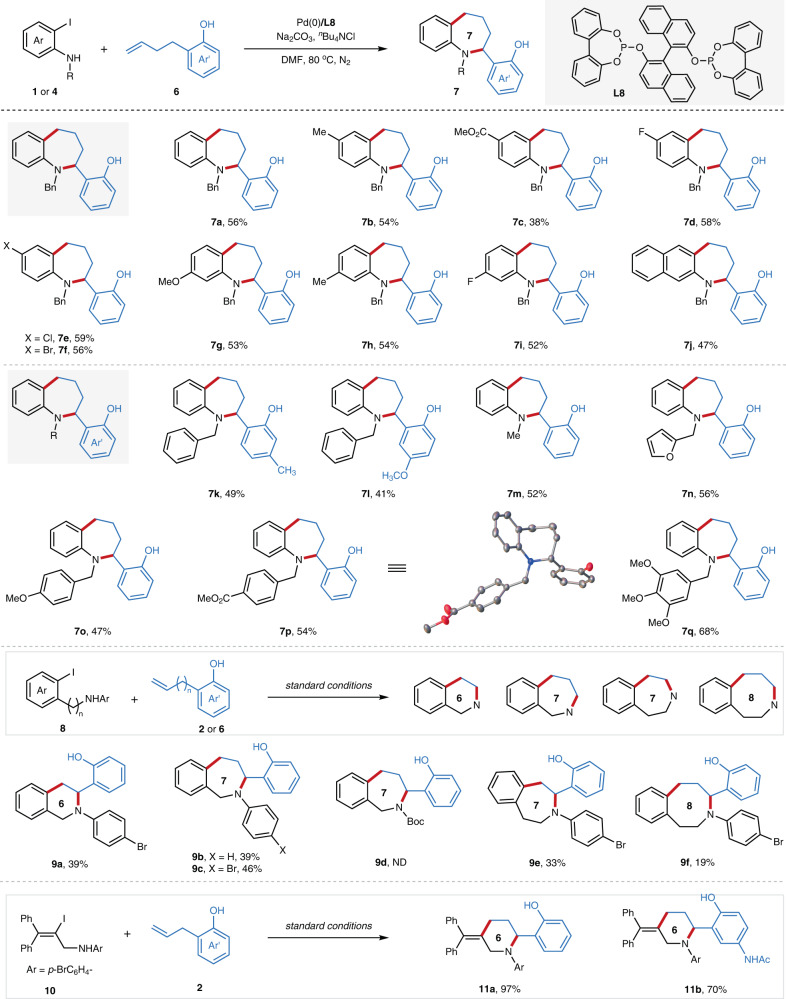
The synthesis of other 6-, 7-, 8-membered azaheterocycles via Pd-catalyzed migratory cycloannulation. The values under each structure indicate isolated yields (See Supplementary Information for experimental details). Reaction conditions: **1** or **4** (0.4 mmol, 4.0 equiv.), **2** or **6** (0.2 mmol), Pd_2_(dba)_3_ (2.8 mg, 1.5 mol%), **L8** (4.3 mg, 3.0 mol%), Na_2_CO_3_ (53.0 mg, 2.5 equiv.), ^*n*^Bu_4_NCl (111.2 mg, 2.0 equiv.), DMF (3.0 mL), 80 °C,18 h. For **7** **f**, **7** **m** and **7n** the reactions were conducted for 36 h. For **9a**, **9b** and **9c**, the reactions were conducted with Pd_2_(dba)_3_ (4.6 mg, 2.5 mol%), **L8** (7.2 mg, 5.0 mol%). Bn, benzyl; Me, methyl; Ph, phenyl; Ar, aryl; Ac, acetyl; Boc, *t*-butoxycarbonyl; DMF, *N*,*N*-dimethylformamide; ND, not detected.

Having thoroughly examined the scope with respect to the construction of tetrahydroquinoline derivatives, we turned our focus to evaluating other azaheterocycles with this approach (Fig. 5). With 2-(but-3-en-1-yl)phenol (**6a**) as the substrate, seven-membered tetrahydrobenzo[*b*]azepines were synthesized in synthetic useful yields under the standard conditions with a variety of functional groups (**7a-j**), including methyl, ester, fluoro, chloro, bromo etc. Substituents on the ω-alkenyl 2-phenols were also compatible (**7k-l**), while substituents at the amines provide moderate yields (**7m-q**). Given the prevalence of all types of tetrahydrobenzoazepines in pharmaceuticals and bioactive molecules, *ortho*-iodide benzylamines and *ortho*-iodide phenylethanamines were also evaluated, giving the 6-, 7-, and 8-membered azaheterocycles (**9a-c** and **9e-9f**) in synthetic useful yields. As mentioned previously, electron-withdrawing protecting group on nitrogen of 2-iodoaniline (**9d**) resulted in no cyclization product. Gratifyingly, 2-iodide allylic amine was also suitable ambiphilic coupling partner for this process, providing an efficient way for the synthesis of piperidine in high yields (**11a-b**).

### Applications

**Fig. 6:**
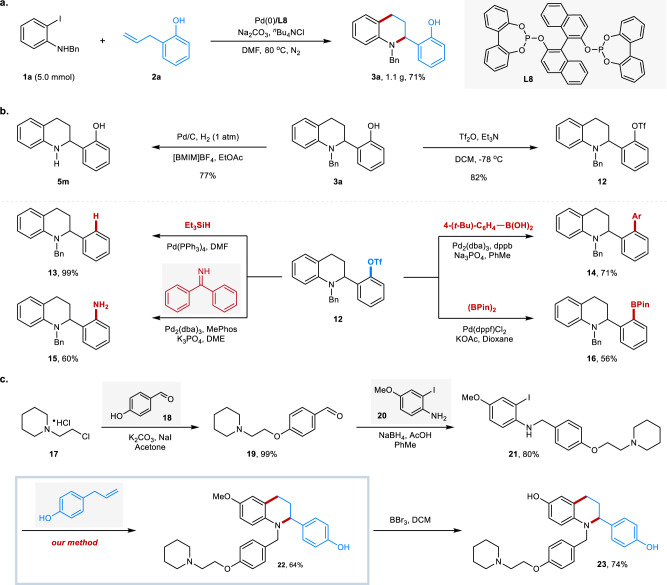
Gram-scale reaction and synthetic application. **a** Gram-scale reaction. **b** Transformation of the benzyl-protected tetrahydroquinoline. **c** Synthesis of potential selective estrogen receptor modulator (SERMs) **23**. Ac, acetyl; Ar, aryl; Bn, benzyl; Me, methyl; Et, ethyl; Ph, phenyl; (Bpin)_2_, bis(pinacolato)diboron; DMF, *N*,*N*-dimethylformamide; DME, 1,2-dimethoxyethane; DCM, dichloromethane; [BMIM]BF_4_, 1-butyl-3-methylimidazolium tetrafluoroborate; dppb, 1,4-bis(diphenylphosphino)butane; MePhos, 2-(dicyclohexylphosphino)−2’-methylbiphenyl.

The scalability of this reaction was demonstrated using **1a** and 2-allylphenol (**2a**) as the model substrates, giving tetrahydroquinoline **3a** in 71% yield on 5.0 mmol scale (Fig. 6a). To further demonstrate the utilities of our method, several derivatizations of the heterocyclic product were conducted (Fig. 6b). The benzyl group could be readily removed in the presence of Pd/C under hydrogen atmosphere, providing the 2-arylated tetrahydroquinoline **5m** in 77% yield. Notably, the hydroxyl group could serve as a versatile linchpin for further decoration of the azaheterocycles, which allows the divergent synthesis of a series of functional azaheterocycles. Treatment of **3a** with trifluoromethanesulfonic anhydride and triethylamine led to corresponding aryl triflate (**12**) in 82% yield, which could transfer to other functionalities via reduction (**13**), Suzuki coupling (**14**), amination (**15**), and borylation (**16**). Our methods could also pave a way for the rapid synthesis of some pharmaceutical molecules with short synthetic routes and high efficiency. A potential selective estrogen receptor modulator (SERMs) **23** has been synthesized with our protocol in 38% total yield in four steps (Fig. 6c), compared with the known synthetic route with 7 steps^55^.

In summary, a Pd-catalyzed migratory cycloannulation strategy for efficient construction of a wide range of azahetereocycles from unactivated aliphatic alkenes has been disclosed. The choice of the *ortho*-hydroxyl group as a locating group to favor the formation of quinone methide intermediates offers an efficient method for controlling the ring-size of the azaheterocycles. We are currently applying this design principle to achieve Pd-catalyzed migratory cycloannulation reactions with other coupling partners.

## Methods

### General procedure for Pd-catalyzed migratory cycloannulation of unactivated alkenes

Pd_2_(dba)_3_ (2.8 mg, 1.5 mol%), **L8** (4.3 mg, 3.0 mol%), Na_2_CO_3_ (53.0 mg, 0.5 mmol) and ^*n*^Bu_4_NCl (111.2 mg, 0.4 mmol) were added to a 10 mL vial in a glovebox. The tube was sealed using a cap with PTFE cap liner and moved outside of the glovebox. DMF (3.0 mL) was added followed by addition of aniline derivatives **1** (0.4 mmol), alkene **2** (0.2 mmol). The reaction mixture was stirred at 80 °C for 18 h. After cooling to room temperature, the reaction mixture was diluted with EtOAc, and the resulted solution was washed with brine for three times. The organic phase was concentrated, and the residue was then purified by column chromatography on silica gel or preparative thin-layer chromatography as mentioned. Full experimental details and characterization of new compounds can be found in the Supplementary Information.

## Supplementary information

Supplementary Information

## Data Availability

X-ray structural data of compound **3g** (ccdc 2161960) and **7p** (ccdc 2161962) are available free of charge from the Cambridge Crystallographic Data Center via www.ccdc.cam.ac.uk/data_request/cif.
