# Supplementary material for: Construction of azaheterocycles via Pd-catalyzed migratory cycloannulation reaction of unactivated alkenes
Source: Nat Commun. 2022 Aug 27;13:5059. doi: 10.1038/s41467-022-32726-x (PMC9420149; doi:10.1038/s41467-022-32726-x)
Supplement: Supplementary file 1 — Supplementary Information [file 41467_2022_32726_MOESM1_ESM.pdf]

Supplementary Information

for

Construction of Azaheterocycles via Pd-Catalyzed Migratory Cycloannulation Reaction of Unactivated Alkenes

Jin-Ping Wang[1], Shuo Song[1], Yichen Wu[1], Peng Wang[1,2,3]

[1]*State Key Laboratory of Organometallic Chemistry, Shanghai Institute of Organic Chemistry, University of Chinese Academy of Sciences, CAS 345 Lingling Road, Shanghai 200032, P.R. China*
[2]*CAS Key Laboratory of Energy Regulation Materials, Shanghai Institute of Organic Chemistry, CAS 345 Lingling Road, Shanghai 200032, P.R. China*
[3]*School of Chemistry and Materials Science, Hangzhou Institute for Advanced Study, University of Chinese Academy of Sciences, 1 Sub-lane Xiangshan, Hangzhou 310024, China*

Supplementary Methods

1. General Information

$Pd_2(dba)_3$ were purchased from LAAJOO. Other reagents were purchased from TCI, Sigma-Aldrich, Acros, Adamas-beta, J&K, 9-Ding, Bidepharm and Energy Chemical of the highest purity grade and used without further purification, unless otherwise indicated. *N, N*-dimethylformamide (DMF) were dried using CaH_2. Other anhydrous solvents were purchased from J&K. The extent of reaction was monitored by thin–layer chromatography (TLC), performed on 0.25 mm silica gel HSGF254. The TLC plates were visualized by ultraviolet light (254 nm) or treatment with potassium permanganate stain followed by gentle heating.

NMR spectra were recorded on Varian 400, Bruker 400 and Agilent 400 (400 MHz for ^{1}H; 375 MHz for ^{19}F; 100 MHz for ^{13}C) spectrometer. The chemical shifts (δ) were quoted in parts per million (ppm) referenced to TMS (0.0 ppm for ^{1}H NMR), $CDCl_3$ (77.0 ppm for ^{13}C NMR), acetone-d^6 (29.8 ppm for ^{13}C NMR) and CD_3OD (49.00 for ^{13}C NMR). The following abbreviations were used to explain multiplicities: s = singlet, d = doublet, t = triplet, q = quartet, m = multiplet and br = broad. Coupling constants, *J*, were reported in Hertz unit (Hz). ^{13}C NMR spectra were fully decoupled by broad band proton decoupling. ^{19}F NMR spectra were recorded on Agilent 400 MR DD2 instrument (375 MHz), and were fully decoupled by broad band proton decoupling. High-resolution mass spectra (HRMS) were recorded on an Agilent Mass spectrometer and Thermo Fisher Scientific LTQ FTICR-MS using ESI–TOF.

2. Experimental Section

2.1 Preparation of Substrates

2.1.1 Preparation of 2-Iodoaniline Derivatives

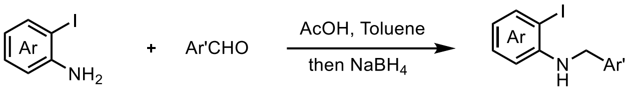

General Procedure A[1]**:** To a solution of 2-iodoaniline derivatives (1.0 equiv.) and aldehyde (1.4 equiv.) in toluene (0.45 M) was added acetic acid (0.15 M). Then the mixture was refluxed for 6 h with a Dean-Stark apparatus. After cooling, $NaBH_4$ (2.0 equiv.) was added portion-wise to the stirring reaction mixture at 0°C. After stirring for another 12 h at room temperature, the mixture was quenched with saturated $NaHCO_3$ (aq). The organic layer was separated, and the water phase was extracted with ethyl acetate for three times. The combined organic layers were washed with saturated $NaHCO_3$ (aq.), dried over Na_2SO_4, filtered, and concentrated under vacuum. The crude product was purified by column chromatography on silica gel to provide the desired product **1** or **4**.

Substrates **1a**, **1g**, **4b**, **4c**, **4d**, **4e**, **4h** were prepared according to the procedure A.

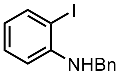

1a

1a was prepared according to procedure A using 2-iodoaniline (12.7 g, 60.0 mmol, 1.0 equiv.), benzaldehyde (8.6 mL, 84 mmol, 1.4 equiv.), $NaBH_4$ (4.5 g, 120 mmol, 2.0 equiv.). Purification by flash chromatography on silica gel (PE) gave the product as a yellow liquid (17.4 g, 94% yield)[1]. ^{1}H NMR (400 MHz, $CDCl_3$) δ 7.67 (dd, *J* = 7.6, 1.6 Hz, 1H), 7.38–7.32 (m, 4H), 7.30–7.27 (m, 1H), 7.18–7.13 (m, 1H), 6.54 (dd, *J* = 8.0, 1.4 Hz, 1H), 6.44 (td, *J* = 7.6, 1.6 Hz, 1H), 4.64 (brs, 1H), 4.40 (s, 2H).

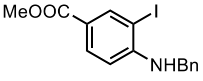

1g

1g was prepared according to procedure A using methyl 4-amino-3-iodobenzoate (554.1 mg, 2.0 mmol, 1.0 equiv.), benzaldehyde (0.2 mL, 2.0 mmol, 1.0 equiv.), $NaBH_4$ (90.8 mg, 2.4 mmol, 1.2 equiv.). Purification by flash chromatography on silica gel (PE/EA= 20/1) gave the product as a white solid (220.4 mg, 30% yield). ^{1}H NMR (400 MHz, $CDCl_3$) δ 8.37 (d, *J* = 2.0 Hz, 1H), 7.84 (dd, *J* = 8.8, 2.0 Hz, 1H), 7.41–7.29 (m, 5H), 6.50 (d, *J* = 8.8 Hz, 1H), 5.08 (brs, 1H), 4.46 (d, *J* = 5.6 Hz, 2H), 3.84 (s, 3H); ^{13}C NMR (100 MHz, $CDCl_3$) δ 165.92, 150.32, 140.76, 137.56, 131.47, 128.87, 127.64, 127.12, 120.11, 109.33, 83.67, 51.77, 48.01; HRMS (ESI-TOF) *m/z* Calcd for $C_{15}H_{15}NO_2I$ $[M+H]^+$: 368.0142, found: 368.0140.

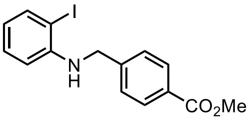

4b

4b was prepared according to procedure A using methyl 2-iodoaniline (2.2 g, 10.0 mmol, 1.0 equiv.), methyl

4-formylbenzoate (2.3 g, 14.0 mmol, 1.4 equiv.), $NaBH_4$ (756.6 mg, 20.0 mmol, 2.0 equiv.). Purification by flash chromatography on silica gel (PE/EA= 15/1) gave the product as a yellow solid (1.9 g, 51% yield). ^{1}H NMR (400 MHz, $CDCl_3$) δ 8.05–7.99 (m, 2H), 7.70–7.68 (m, 1H), 7.42 (d, *J* = 8.0 Hz, 2H), 7.15–7.11 (m, 1H), 6.49–6.44 (m, 2H), 4.65 (br, 1H), 4.48 (s, 2H), 3.91 (s, 3H); ^{13}C NMR (100 MHz, $CDCl_3$) δ 166.83, 146.64, 144.06, 139.04, 130.00, 129.39, 129.19, 126.86, 119.13, 110.94, 85.34, 52.06, 48.00; HRMS (ESI-TOF) *m/z* Calcd for $C_{15}H_{15}NO_2I$ $[M+H]^+$: 368.0142, found: 368.0141.

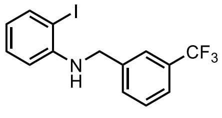

4c

4c was prepared according to procedure A using methyl 2-iodoaniline (1.1 g, 5.0 mmol, 1.0 equiv.), 3-(trifluoromethyl)benzaldehyde (1.0 mL, 7.0 mmol, 1.4 equiv.), $NaBH_4$ (378.3 mg, 10.0 mmol, 2.0 equiv.). Purification by flash chromatography on silica gel (PE) gave the product as a light-yellow liquid (1.2 g, 65% yield). ^{1}H NMR (400 MHz, $CDCl_3$) 7.69 (d, *J* = 8.0 Hz, 1H), 7.62 (s, 1H), 7.54 (d, *J* = 8.0 Hz, 2H), 7.49–7.45 (m, 1H), 7.15 (t, *J* = 7.6 Hz, 1H), 6.47 (t, *J* = 7.6 Hz, 2H), 4.68 (brs, 1H), 4.47 (d, *J* = 5.6 Hz, 2H); ^{13}C NMR (100 MHz, $CDCl_3$) δ 146.53, 139.74, 139.00, 130.77 (q, *J* = 32.2 Hz), 130.19, 129.40, 129.09, 124.07 (q, *J* = 272 Hz), 124.02 (q, *J* = 3.8 Hz), 123.69 (q, *J* = 3.8 Hz), 119.22, 110.89, 85.45, 47.68; ^{19}F NMR (375 MHz, $CDCl_3$) δ -62.61; HRMS (ESI-TOF) *m/z* Calcd for $C_{14}H_{12}NF_3I$ $[M+H]^+$: 377.9961, found: 377.9959.

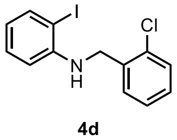

4d

4d was prepared according to procedure A using methyl 2-iodoaniline (2.2 g, 10.0 mmol, 1.0 equiv.), 2-chlorobenzaldehyde (1.6 mL, 14.0 mmol, 1.4 equiv.), $NaBH_4$ (756.6 mg, 20.0 mmol, 2.0 equiv.). Purification by flash chromatography on silica gel (PE) gave the product as a white solid (3.3 g, 95% yield). ^{1}H NMR (400 MHz, $CDCl_3$) 7.70–7.67 (m, 1H), 7.39 (dd, *J* = 5.6, 3.6 Hz, 1H), 7.34 (dd, *J* = 5.6, 3.6 Hz, 1H), 7.24–7.20 (m, 2H), 7.17–7.12 (m, 1H), 6.48–6.44 (m, 2H), 4.72 (br, 1H), 4.51 (s, 2H); ^{13}C NMR (100 MHz, $CDCl_3$) δ 146.65, 139.05, 135.82, 133.12, 129.55, 129.44, 128.51, 128.44, 126.98, 119.04, 110.93, 85.30, 45.88; HRMS (ESI-TOF) *m/z* Calcd for $C_{13}H_{12}NClI$ $[M+H]^+$:343.9697, found: 3434.9693.

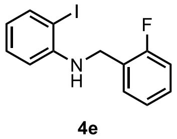

4e

4e was prepared according to procedure A using methyl 2-iodoaniline (2.2 g, 10.0 mmol, 1.0 equiv.), 2-fluorobenzaldehyde (1.5 mL, 14.0 mmol, 1.4 equiv.), $NaBH_4$ (756.6 mg, 20.0 mmol, 2.0 equiv.). Purification by flash chromatography on silica gel (PE) gave the product as a yellow liquid (1.2 g, 35% yield). ^{1}H NMR (400 MHz, $CDCl_3$) 7.68 (dd, *J* = 8.0, 1.6 Hz, 1H), 7.34 (td, *J* = 7.6, 1.6 Hz, 1H), 7.30–7.22 (m, 1H), 7.18–7.14 (m, 1H), 7.12–7.04

(m, 2H), 6.55 (dd, J = 8.4, 1.6 Hz, 1H), 6.46 (td, J = 7.6, 1.6 Hz, 1H), 4.65 (brs, 1H), 4.48 (s, 2H); ^{13}C NMR (100 MHz, $CDCl_3$) δ 160.78 (d, J = 245.9 Hz), 146.72, 139.04, 129.43, 128.92 (d, J = 4.7 Hz), 128.86 (d, J = 8.6 Hz), 125.55 (d, J = 14.4 Hz), 124.26 (d, J = 3.6 Hz), 119.04, 115.34 (d, J = 21.2 Hz), 110.85, 85.38, 41.81 (d, J = 4.6 Hz); ^{19}F NMR (375 MHz, $CDCl_3$) δ -118.86; HRMS (ESI-TOF) *m/z* Calcd for $C_{13}H_{12}NFI$ $[M+H]^+$: 327.9993, found: 327.9991.

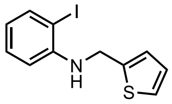

4h

4h was prepared according to procedure A using methyl 2-iodoaniline (2.2 g, 10.0 mmol, 1.0 equiv.), 2-thenaldehyde (1.1 mL, 12.0 mmol, 1.2 equiv.), $NaBH_4$ (756.6 mg, 20.0 mmol, 2.0 equiv.). Purification by flash chromatography on silica gel (PE/EA= 20/1) gave the product as a yellow liquid (3.1 g, 99% yield)[1]. ^{1}H NMR (400 MHz, $CDCl_3$) 7.68 (dd, J = 7.6, 1.6 Hz, 1H), 7.23 (dd, J = 5.2, 1.2 Hz, 1H), 7.21–7.17 (m, 1H), 7.02 (dd, J = 3.4, 1.2 Hz, 1H), 6.98 (dd, J = 5.2, 3.6 Hz, 1H), 6.65–6.62 (m, 1H), 6.48 (td, J = 7.6, 1.6 Hz, 1H), 4.62 (brs, 1H), 4.57 (d, J = 3.6 Hz, 2H).

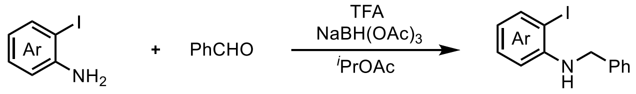

General Procedure B[2]: An oven-dried flask was charged with 2-iodoaniline derivatives (1.0 equiv.) and aldehyde (1.1 equiv.) in ^{i}PrOAc (0.3 M). TFA (2.0 equiv.) was added dropwise at room temperature, and the resulting mixture was stirred for 10 min at the same temperature. Then $NaBH(OAc)_3$ (1.2 equiv.) was added portion-wise. After stirring at room temperature overnight, the mixture was quenched with 10% NaOH (aq.) and extracted with ethyl acetate for three times. The combined organic layers were washed with brine and dried over Na_2SO_4. The solvent was evaporated under reduced pressure. The crude product was purified by column chromatography on silica gel to give desired substrate.

Substrates **1j**, **1k**, **4j**, **4k** were prepared according to the procedure B.

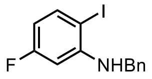

1j

1j was prepared according to procedure B using 5-fluoro-2-iodoaniline (0.24 mL, 2.0 mmol, 1.0 equiv.), benzaldehyde (0.22 mL, 2.2 mmol, 1.1 equiv.), TFA (0.3 mL, 4.0 mmol, 2.0 equiv.), $NaBH(OAc)_3$ (508.7 mg, 2.4 mmol, 1.2 equiv.). Purification by flash chromatography on silica gel (PE) gave the product as a colorless liquid (659.1 mg, 96% yield). ^{1}H NMR (400 MHz, $CDCl_3$) δ 7.43 (dd, J = 8.0, 2.8 Hz, 1H), 7.37–7.24 (m, 5H), 6.91 (td, J = 8.0, 2.8 Hz, 1H), 6.44 (dd, J = 8.8, 4.8 Hz, 1H), 4.44 (brs, 1H), 4.36 (d, J = 5.2 Hz, 2H); ^{13}C NMR (100 MHz, $CDCl_3$) δ 154.72 (d, J = 239.3 Hz), 143.90 (d, J = 1.8 Hz), 138.43, 128.74, 127.38, 127.14, 125.47 (d, J = 24.8 Hz), 115.85 (d, J = 21.7 Hz), 110.66 (d, J = 7.4 Hz), 83.68 (d, J = 8.8 Hz), 48.86; ^{19}F NMR (375 MHz, $CDCl_3$) δ -126.94; HRMS (ESI-TOF) *m/z* Calcd for $C_{13}H_{12}NFI$ $[M+H]^+$: 327.9993, found: 327.9989.

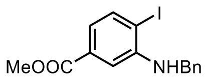

1k

1k was prepared according to procedure B using methyl 3-amino-4-iodobenzoate (554.1 mg, 2.0 mmol, 1.0 equiv.), benzaldehyde (0.22 mL, 2.2 mmol, 1.1 equiv.), TFA (0.3 mL, 4.0 mmol, 2.0 equiv.), $NaBH(OAc)_3$ (508.7 mg, 2.4 mmol, 1.2 equiv.). Purification by flash chromatography on silica gel (PE) gave the product as a white liquid (781.6 mg, 99% yield)[2]. ^{1}H NMR (400 MHz, $CDCl_3$) δ 7.75 (d, J = 8.0 Hz, 1H), 7.38–7.28 (m, 5H), 7.22 (d, J = 2.0 Hz, 1H), 7.10 (dd, J = 8.0, 2.0 Hz, 1H), 4.66 (brs, 1H), 4.44 (d, J = 5.6 Hz, 2H), 3.87 (s, 3H).

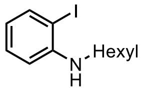

4j

4j was prepared according to procedure B using iodoaniline (1.1 mL, 5.0 mmol, 1.0 equiv.), hexanal (0.72 mL, 6.0 mmol, 1.2 equiv.), TFA (0.8 mL, 10.0 mmol, 2.0 equiv.), $NaBH(OAc)_3$ (2.4 g, 11.5 mmol, 2.3 equiv.). Purification by flash chromatography on silica gel (PE) gave the product as a light-yellow liquid (934.7 mg, 62% yield)[3]. ^{1}H NMR (400 MHz, $CDCl_3$) δ 7.64 (d, J = 8.0 Hz, 1H), 7.20 (t, J = 7.6 Hz, 1H), 6.55 (d, J = 8.0 Hz, 1H), 6.42 (t, J = 7.6 Hz, 1H), 4.12 (brs, 1H), 3.14 (q, J = 6.4 Hz, 2H), 1.67 (p, J = 7.2 Hz, 2H), 1.46–1.39 (m, 1H), 1.35-–1.33 (m, 4H), 0.93–0.89 (m, 3H).

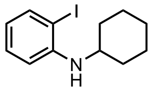

4k

4k was prepared according to procedure B using iodoaniline (1.1 mL, 5.0 mmol, 1.0 equiv.), cyclohexanecarbaldehyde (0.52 mL, 5.0 mmol, 1.0 equiv.), TFA (0.8 mL, 10.0 mmol, 2.0 equiv.), $NaBH(OAc)_3$ (1.8 g, 8.5 mmol, 1.7 equiv.). Purification by flash chromatography on silica gel (PE) gave the product as a colorless liquid (1.0 mg, 62% yield)[3]. ^{1}H NMR (400 MHz, $CDCl_3$) δ 7.64 (d, J = 7.6 Hz, 1H), 7.17 (t, J = 7.6 Hz, 1H), 6.57 (d, J = 8.2 Hz, 1H), 6.39 (t, J = 7.6 Hz, 1H), 4.10 (brs, 1H), 3.32 (brs, 1H), 2.03 (d, J = 12.2 Hz, 2H), 1.79–1.56 (m, 2H), 1.66–1.63 (m, 1H), 1.44–1.23 (m, 5H).

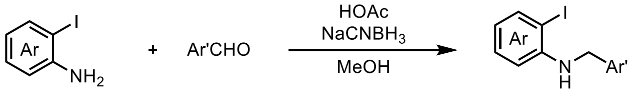

4

General Procedure C[4]: An oven-dried flask was charged with 2-iodoaniline derivatives (1.0 equiv.) and aldehyde (1.0 equiv.) in MeOH (0.3 M). HOAc (1.5 equiv.) was added dropwise at room temperature and the resulting mixture was stirred for 30 min at the same temperature. Then $NaCNBH_3$ (1.3 equiv.) was added portion-wise at 0 ^{o}C. After stirring at room temperature overnight, the mixture was quenched with $NaHCO_3$ (aq.) and extracted with ethyl acetate for three times. The combined organic layers were washed with brine, dried over Na_2SO_4. filtered, and concentrated under vacuum. The crude product was purified by column chromatography on silica gel to provide the desired product.

Substrates **4a**, **4f**, **4g** were prepared according to the procedure C.

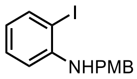

4a

4a was prepared according to procedure C using 2-iodoaniline (2.2 g, 10.0 mmol, 1.0 equiv.), 4-methoxybenzaldehyde (1.2 mL, 84.0 mmol, 1.0 equiv.), HOAc (0.87 mL, 15.0 mmol, 1.5 equiv.), $NaCNBH_3$ (816.9 mg, 13.0 mmol, 1.3 equiv.f). Purification by flash chromatography on silica gel (PE/EA= 20/1) gave the product as a white solid (1.8 g, 52% yield)[4]. ^{1}H NMR (400 MHz, $CDCl_3$) δ 7.67 (d, *J* = 8.0 Hz, 1H), 7.28 (d, *J* = 8.4 Hz, 2H), 7.16 (t, *J* = 7.2 Hz, 1H), 6.89 (d, *J* = 8.4 Hz, 2H), 6.55 (d, *J* = 8.0 Hz, 1H), 6.44 (t, *J* = 7.2 Hz, 1H), 4.53 (brs, 1H), 4.32 (s, 2H), 3.81 (s, 3H).

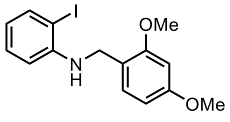

4f

4f was prepared according to procedure C using 2-iodoaniline (657.1 mg, 3.0 mmol, 1.0 equiv.), 4-methoxybenzaldehyde (598.2 mg, 3.6 mmol, 1.2 equiv.), HOAc (0.3 mL, 4.5 mmol, 1.5 equiv.), $NaCNBH_3$ (245.1 mg, 3.9 mmol, 1.3 equiv.). Purification by flash chromatography on silica gel (PE/EA= 50/1) gave the product as a colorless liquid (1.1 g, 99% yield). ^{1}H NMR (400 MHz, $CDCl_3$) δ 7.65 (dd, *J* = 7.6, 1.6 Hz, 1H), 7.18–7.14 (m, 2H), 6.60 (dd, *J* = 8.0, 1.6 Hz, 1H), 6.48 (d, *J* = 2.4 Hz, 1H), 6.44–6.40 (m, 2H), 4.69 (brs, 1H), 4.31 (s, 2H), 3.85 (s, 3H), 3.79 (s, 3H); ^{13}C NMR (100 MHz, $CDCl_3$) δ 160.24, 158.39, 147.41, 138.91, 129.32, 118.91, 118.53, 111.13, 103.85, 98.61, 85.53, 55.34, 43.38; HRMS (ESI-TOF) *m/z* Calcd for $C_{15}H_{17}O_2NI$ $[M+H]^+$: 370.0298, found: 370.0297.

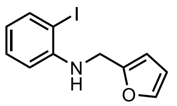

4g

4g was prepared according to procedure C using 2-iodoaniline (2.2 g, 10.0 mmol, 1.0 equiv.), 2-furaldehyde (0.8 mL, 10.0 mmol, 1.0 equiv.), HOAc (0.9 mL, 15.0 mmol, 1.5 equiv.), $NaCNBH_3$ (816.9 mg, 13.0 mmol, 1.3 equiv.). Purification by flash chromatography on silica gel (PE/EA= 50/1) gave the product as a yellow liquid (2.3 g, 78% yield)[1]. ^{1}H NMR (400 MHz, $CDCl_3$) δ 7.67 (dd, *J* = 7.6, 1.76 Hz, 1H), 7.38–7.37 (m, 1H), 7.20 (td, *J* = 7.6 Hz, 1H), 6.65 (dd, *J* = 8.0 Hz, 1H), 6.47 (td, *J* = 7.6, 1.6 Hz, 1H), 6.33 (dd, *J* = 3.2, 1.6 Hz, 1H), 6.25–6.24 (m, 1H), 4.56 (brs, 1H), 4.37 (d, *J* = 4.4 Hz, 2H).

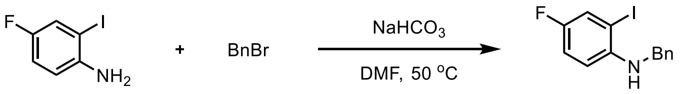

1c

An oven-dried flask was charged with 4-fluoro-2-iodoaniline (1.2 mL, 10.0 mmol, 1.0 equiv.), benzyl bromide (1.4 mL, 12.0 mmol, 1.2 equiv.), $NaHCO_3$ (1.7 g, 20.0 mmol, 2.0 equiv.) and DMF (0.3 M). The mixture was stirred at 50 °C until complete consumption of starting material indicated by TLC. Then the mixture was cooled to room temperature, quenched with H_2O and extracted with EA for three times. The combined organic layers were washed with brine and dried over Na_2SO_4, filtered, and concentrated under vacuum. The crude product was purified by

column chromatography on silica gel (PE) to give the product **1c** as a yellow liquid (1.8 g, 55% yield).[2] ^{1}H NMR (400 MHz, $CDCl_3$) δ 7.43 (dd, *J* = 8.0, 2.8 Hz, 1H), 7.38–7.33 (m, 4H), 7.31–7.27 (m, 1H), 6.93–6.89 (m, 1H), 6.44 (dd, *J* = 9.2, 4.8 Hz, 1H), 4.46 (brs, 1H), 4.36 (s, 2H).

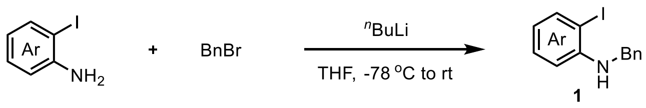

General Procedure D: An oven-dried flask was charged with 2-iodoaniline derivatives (2.0 equiv.) and THF (0.15 M), a solution of ^{n}BuLi (1.0 equiv., 2.5 M in Hexane) was added slowly at −78 °C and the resulting mixture was stirred for 30 min at the same temperature. Then benzyl bromide (1.0 equiv.) was added. After stirring at room temperature overnight, the mixture was quenched with silica gel and concentrated under vacuum. The crude product was purified by column chromatography on silica gel to provide the desired product.

Substrates **1b**, **1d**, **1e**, **1f**, **1h**, **1i** were prepared according to the above procedure D.

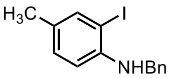

1b

1b was prepared according to procedure D using 2-iodo-4-methylaniline (1.4 g, 6.0 mmol, 2.0 equiv.), benzyl bromide (0.36 mL, 3.0 mmol, 1.0 equiv.), ^{n}BuLi (2.5 M in Hexane, 1.2 mL, 3.0 mmol, 1.0 equiv.) Purification by flash chromatography on silica gel (PE) gave the product as a yellow liquid (830.1 mg, 86% yield)[2]. ^{1}H NMR (400 MHz, $CDCl_3$) δ 7.52 (d, *J* = 2.0 Hz, 1H), 7.37–7.32 (m, 4H), 7.30–7.26 (m, 1H), 6.96 (dd, *J* = 8.2, 2.0 Hz, 1H), 6.44 (d, *J* = 8.2 Hz, 1H), 4.47 (brs, 1H), 4.38 (d, *J* = 3.6 Hz, 2H), 2.19 (s, 3H).

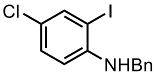

1d

1d was prepared according to procedure D using 4-chloro-2-iodoaniline (2.5 g, 10.0 mmol, 2.0 equiv.), benzyl bromide (0.6 mL, 5.0 mmol, 1.0 equiv.), ^{n}BuLi (2.0 mL, 2.5 M in Hexane, 5.0 mmol, 1.0 equiv.) Purification by flash chromatography on silica gel (PE) gave the product as a yellow liquid (1.4 g, 82% yield)[5]. ^{1}H NMR (400 MHz, $CDCl_3$) δ 7.64 (d, *J* = 2.4 Hz, 1H), 7.38–7.27 (m, 5H), 7.11 (dd, *J* = 8.8, 2.4 Hz, 1H), 6.42 (d, *J* = 8.8 Hz, 1H), 4.63 (brs, 1H), 4.38 (d, *J* = 5.6 Hz, 2H).

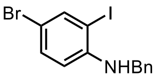

1e

1e was prepared according to procedure D using 4-bromo-2-iodoaniline (1.8 g, 6.0 mmol, 2.0 equiv.), benzyl bromide (0.36 mL, 3.0 mmol, 1.0 equiv.), ^{n}BuLi (1.2 mL, 2.5 M in Hexane, 3.0 mmol, 1.0 equiv.) Purification by flash chromatography on silica gel (PE) gave the product as a yellow liquid (774.6 mg, 67% yield)[1]. ^{1}H NMR (400 MHz, $CDCl_3$) δ 7.76 (d, *J* = 2.4 Hz, 1H), 7.37–7.28 (m, 4H), 7.26–7.23 (m, 2H), 6.37 (d, *J* = 8.8 Hz, 1H), 4.64 (brs, 1H), 4.38 (d, *J* = 5.6 Hz, 2H).

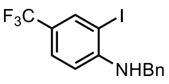

1f

1f was prepared according to procedure D using 2-iodo-4-(trifluoromethyl)aniline (1.4 g, 5.0 mmol, 2.0 equiv.), benzyl bromide (0.30 mL, 2,5 mmol, 1.0 equiv.), ^{n}BuLi (1.0 mL, 2.5 M in Hexane, 2.5 mmol, 1.0 equiv.) Purification by flash chromatography on silica gel (PE) gave the product as a yellow liquid (525.5 mg, 56% yield)[2]. ^{1}H NMR (400 MHz, $CDCl_3$) δ7.90–7.89 (m, 1H), 7.40–7.26 (m, 6H), 6.52 (d, *J* = 8.8 Hz, 1H), 4.99 (brs, 1H), 4.45 (d, *J* = 5.6 Hz, 2H).

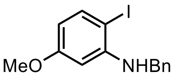

1h

1h was prepared according to procedure D using 2-iodo-5-methoxyaniline (3.7 g, 14.7 mmol, 2.0 equiv.), benzyl bromide (0.88 mL, 7.4 mmol, 1.0 equiv.), ^{n}BuLi (3.0 mL, 2.5 M in Hexane, 7.4 mmol, 1.0 equiv.) Purification by flash chromatography on silica gel (PE) gave the product as a white liquid (1.3 g, 53% yield)[6]. ^{1}H NMR (400 MHz, $CDCl_3$) δ 7.53 (d, *J* = 8.4 Hz, 1H), 7.38–7.33 (m, 4H), 7.30–7.28 (m, 1H), 6.14 (d, *J* = 2.8 Hz, 1H), 6.09 (dd, *J* = 8.4, 2.8 Hz, 1H), 4.59 (brs, 1H), 4.38 (d, *J* = 5.2 Hz, 2H), 3.70 (s, 3H).

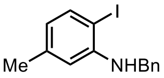

1i

1i was prepared according to procedure D using 2-iodo-5-methylaniline (1.4 g, 6.0 mmol, 2.0 equiv.), benzyl bromide (0.36 mL, 3.0 mmol, 1.0 equiv.), ^{n}BuLi (1.2 mL, 2.5 M in Hexane, 3.0 mmol, 1.0 equiv.) Purification by flash chromatography on silica gel (PE) gave the product as a yellow liquid (677.0 mg, 70% yield)[2]. ^{1}H NMR (400 MHz, $CDCl_3$) δ 7.53 (d, *J* = 8.0 Hz, 1H), 7.37–7.33 (m, 4H), 7.32–7.27 (m, 1H), 6.39 (d, *J* = 2.0 Hz, 1H), 6.32–6.29 (m, 1H), 4.52 (brs, 1H), 4.38 (d, *J* = 5.6 Hz, 2H), 2.22 (s, 3H).

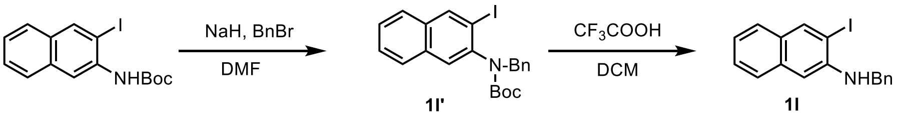

An oven-dried flask was charged with *tert*-butyl (3-iodonaphthalen-2-yl)carbamate (3.7 g, 10.0 mmol, 1.0 equiv.) and DMF (0.2 M). NaH (800.0 mg, 20.0 mmol, 2.0 equiv.) was added slowly for several portions at room temperature, and the resulting mixture was stirred for 30 min at the same temperature. Then benzyl bromide (1.4 mL, 12 mmol, 1.2 equiv.) was added at 0 ^{o}C. After stirring at same temperature for 20 min, the mixture was quenched with NH_4Cl aqueous and extracted with ethyl acetate for three times. The combined organic layers were washed with brine and dried over Na_2SO_4, filtered, and concentrated under vacuum. The crude product **1l'** was used directly without further purification.

To a solution of **1l'** (5.0 g, 10.0 mmol, 1.0 equiv.) in DCM (0.2 M) was added CF_3COOH (10 mL, 1.0 M) at room temperature. After stirring at same temperature for 20 min, the mixture was quenched with $NaHCO_3$ aqueous and

extracted with DCM for three times. The combined organic layers were washed with brine and dried over Na_2SO_4, filtered, and concentrated under vacuum. The crude product was purified by column chromatography on silica gel (PE/EA= 20/1) to give the product **1l** as a yellow liquid (2.8 g, 79% yield).[6] ^{1}H NMR (400 MHz, $CDCl_3$) 8.27 (s, 1H), 7.56 (t, *J* = 8.0 Hz, 2H), 7.44–7.25 (m, 6H), 7.21–7.17 (m, 1H), 6.78 (s, 1H), 4.74 (brs, 1H), 4.48 (s, 2H); ^{13}C NMR (100 MHz, $CDCl_3$) δ 143.68, 138.74, 138.25, 134.71, 128.70, 128.53, 127.34, 127.31, 126.77, 126.46, 125.91, 122.53, 104.56, 89.43, 48.55; HRMS (ESI-TOF) *m/z* Calcd for $C_{15}H_{15}NI$ $[M+H]^+$: 360.0244, found: 360.0243.

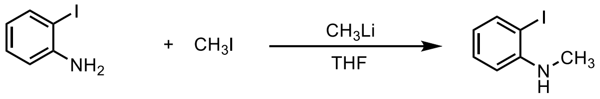

4i

An oven-dried flask was charged with 2-iodoaniline (2.2 g, 10.0 mmol, 1.0 equiv.) and THF (0.4 M). A solution of CH_3Li (6.3 mL, 1.6 M in diethoxy-methane, 10.0 mmol, 1.0 equiv.) was added dropwise at −78 °C and the resulting mixture was stirred for 30 min at the same temperature. Then, CH_3I (0.8 mL, 13.0 mmol, 1.3 equiv.) was added dropwise. The mixture was warmed to room temperature and stirred for another 2 h. The mixture was quenched with NH_4Cl (aq.) and extracted with Et_2O for three times. The combined organic layers were washed with brine, dried over Na_2SO_4, filtered, and concentrated under vacuum. The crude product was purified by column chromatography on silica gel (PE/EA= 10/1) to give the product **4i** as a yellow liquid (2.0 g, 94% yield).[1] ^{1}H NMR (400 MHz, $CDCl_3$) 7.65 (dd, *J* = 7.6, 1.6 Hz, 1H), 7.26–7.21 (m, 1H), 6.56 (dd, *J* = 8.0, 1.6 Hz, 1H), 6.45 (td, *J* = 7.6, 1.6 Hz, 1H), 4.20 (brs, 1H), 2.89 (s, 3H).

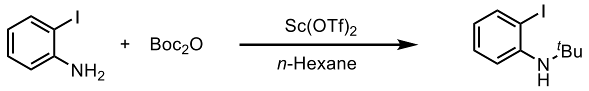

4l

An oven-dried flask was charged with 2-iodoaniline (1.1 g, 5.0 mmol, 1.0 equiv.), $Sc(OTf)_3$ (37.9 mg, 0.075 mmol, 1.5 mol%), *n*-Hexane (1.0 M) and Boc_2O (0.5 M) under N_2 atmosphere. The mixture was stirred at the 50 °C overnight. Upon completion, the reaction mixture was evaporated under reduced pressure. The crude product was purified by column chromatography on silica gel (PE/EA= 20/1) to give the product **4l** as a yellow liquid (158.7 mg, 11% yield).[7] ^{1}H NMR (400 MHz, $CDCl_3$) 7.67 (d, *J* = 8.0 Hz, 1H), 7.16 (t, *J* = 8.0 Hz, 1H), 6.92 (d, *J* = 8.0 Hz, 1H), 6.42 (t, *J* = 7.6 Hz, 1H), 4.15 (brs, 1H), 1.41 (s, 9H).

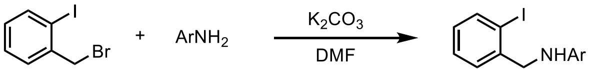

8

A round bottle was charged with K_2CO_3 (2.0 equiv.) and DMF (0.5 M). Then aniline (1.4 mL, 2.0 equiv.) was added slowly and stirred at the room temperature for 10 min, followed by addition of 1-(bromomethyl)-2-iodobenzene (1.0 equiv.). After stirring at the 80 °C overnight, the mixture was quenched with H_2O at room temperature and extracted with Et_2O for three times. The combined organic layers were washed with brine, dried over Na_2SO_4, filtered, and concentrated under vacuum. The crude product was purified by column

chromatography on silica gel.

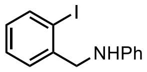

8a

8a was prepared according to above procedure using 1-(bromomethyl)-2-iodobenzene (1.5 g, 5.0 mmol, 1.0 equiv.), aniline (1.4 mL, 15.0 mmol, 3.0 equiv.), K_2CO_3 (1.4 g, 10.0 mmol, 2.0 equiv.). Purification by flash chromatography on silica gel (PE) gave the product as a colorless liquid (1.5 g, 97% yield)[8]. ^{1}H NMR (400 MHz, $CDCl_3$) δ 7.85 (dd, *J* = 8.0, 1.2 Hz, 1H), 7.39 (dd, *J* = 7.6, 1.6 Hz, 1H), 7.32–7.27 (m, 1H), 7.20–7.15 (m, 2H), 6.97 (td, *J* = 7.6, 1.6 Hz, 1H), 6.73 (tt, *J* = 7.6, 1.2 Hz, 1H), 6.62–6.60 (m, 2H), 4.33 (s, 3H).

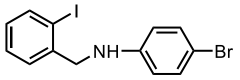

8b

8b was prepared according to above procedure using 1-(bromomethyl)-2-iodobenzene (1.5 g, 5.0 mmol, 1.0 equiv.), aniline (2.6 g, 15.0 mmol, 3.0 equiv.), K_2CO_3 (1.4 g, 10.0 mmol, 2.0 equiv.). Purification by flash chromatography on silica gel (PE/EA= 50/1) gave the product as a yellow liquid (1.7 g, 90% yield). ^{1}H NMR (400 MHz, $CDCl_3$) δ 7.85 (d, *J* = 8.0 Hz, 1H), 7.33–7.28 (m, 2H), 7.24–7.22 (m, 2H), 6.98 (t, *J* = 7.2 Hz, 1H), 6.47–6.44 (m, 2H), 4.28 (s, 2H), 4.20 (brs, 1H); ^{13}C NMR (100 MHz, $CDCl_3$) δ 146.50, 140.30, 139.53, 131.94, 129.08, 128.61, 128.42, 114.50, 109.36, 98.45, 53.08; HRMS (ESI-TOF) *m/z* Calcd for $C_{13}H_{12}NBrI$ $[M+H]^+$: 387.9192, found:387.9190.

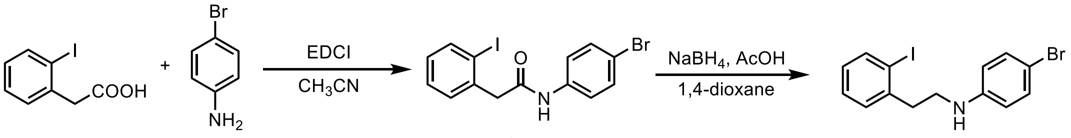

The synthesis of **8d'** was performed according to the reported literature.[9] An oven-dried flask was charged with 2-(2-iodophenyl)acetic acid (1.3 g, 5.0 mmol, 1.0 equiv.), 4-bromoaniline (860.2 mg, 5.0 mmol, 1.0 equiv.), EDCI (1.4 g, 7.5 mmol, 1.5 equiv.) and CH_3CN (0.25 M) under N_2 atmosphere. After stirring at room temperature overnight, the mixture was quenched with H_2O and extracted with EA for three times. The combined organic layers were washed with 10% HCl (aq.), $NaHCO_3$ (aq.) and brine sequentially, dried over Na_2SO_4, filtered, and concentrated under vacuum. The crude product was purified by column chromatography on silica gel (PE/EA= 5/1) to give the product **8d'** as a yellow solid (1.0 g, 48% yield). ^{1}H NMR (400 MHz, $CDCl_3$) δ 7.91 (d, *J* = 8.0 Hz, 1H), 7.42–7.34 (m, 6H), 7.10 (brs, 1H), 7.06–7.02 (m, 1H), 3.88 (s, 2H).

An oven-dried flask was charged with **8d'** (749.1 mg, 1.8 mmol, 1.0 equiv.) and CH_3CN (0.25 M) under N_2 atmosphere. Then NaBH4 (340.5 mg, 9.0 mmol, 5.0 equiv.) and AcOH (0.2 mL, 3.0 mmol, 2.0 equiv.) was added sequentially at room temperature. After stirring at 80 ^{o}C overnight, the mixture was quenched with H_2O at room temperature and extracted with ethyl acetate for three times. The combined organic layers were washed with brine, dried over Na_2SO_4, filtered, and concentrated under vacuum. The crude product was purified by column chromatography on silica gel (PE/EA= 20/1) to give the product **8d** as a colorless liquid (307.2 mg, 42% yield). ^{1}H

NMR (400 MHz, $CDCl_3$) δ 7.81 (d, *J* = 8.0, 1H), 7.32–7.27 (m, 2H), 7.25–7.24 (m, 1H),, 7.20 (dd, *J* = 7.6, 1.6 Hz, 1H), 6.96–6.91 (m, 1H), 6.53–6.51 (m, 2H), 3.76 (brs 1H), 3.37 (t, *J* = 7.2 Hz, 2H), 3.02 (t, *J* = 7.2 Hz, 2H); ^{13}C NMR (100 MHz, $CDCl_3$) δ 146.74, 141.68, 139.63, 131.89, 129.89, 128.42, 128.34, 114.41, 108.87, 100.56, 43.61, 40.06; HRMS (ESI-TOF) *m/z* Calcd for $C_{14}H_{14}NBrI$ $[M+H]^+$: 401.9349, found: 401.9344.

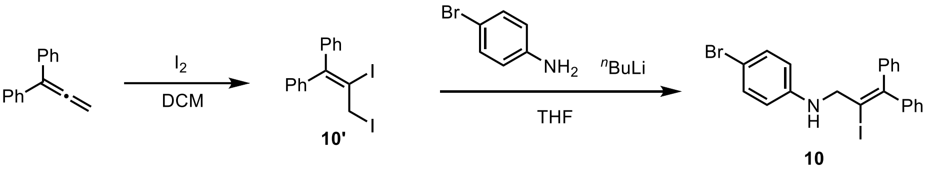

An oven-dried flask was charged with allene (5.6 g, 28.9 mmol, 1.0 equiv.) and DCM (0.6 M) under N_2 atmosphere, followed by the slow addition of the solution of iodine (7.3 g, 0.6 M in DCM, 28.9 mmol, 1.0 equiv.) at 0 °C. The mixture was warmed to room temperature gradually. After stirring overnight, the mixture was quenched with saturated sodium thiosulfate (aq.) and extracted with DCM for three times. The combined organic layers were washed with brine, dried over Na_2SO_4, filtered, and concentrated under vacuum. The crude product was purified by column chromatography on silica gel (PE/DCM= 5/1) to give the product **10'** as a yellow solid (9.4 g, 73% yield). ^1H NMR (400 MHz, $CDCl_3$) δ 7.39–7.29 (m, 8H), 7.24–7.21 (m, 2H), 4.55 (s, 2H); ^{13}C NMR (100 MHz, $CDCl_3$) δ 150.98, 145.67, 139.22, 128.66, 128.53, 128.05, 127.98, 127.90, 127.46, 103.20, 19.22.

An oven-dried flask was charged with 4-bromoaniline (412.8 mg, 2.4 mmol, 1.2 equiv.) and THF (0.2 M) under N_2 atmosphere, a solution of ^{n}BuLi (1.0 mL, 2.5 M in Hexane, 2.4 mmol, 1.2 equiv.) was added dropwise at 0 °C and the resulting mixture was stirred for 30 min at the same temperature. Then a solution of (2,3-diiodoprop-1-ene-1,1-diyl)dibenzene (891.8 mg, 2.0 mmol, 1.0 equiv.) in THF (5.0 mL) was added dropwise. After stirring overnight at room temperature, the mixture was quenched with H_2O at 0 °C, and extracted with EA for three times. The combined organic layers were washed with brine, dried over Na_2SO_4, filtered, and concentrated under vacuum. The crude product was purified by column chromatography on silica gel (PE/EA= 20/1) to give the product **10** as a yellow viscous fluid (904.3 mg, 92% yield). ^1H NMR (400 MHz, $CDCl_3$) δ 7.37–7.27 (m, 6H), 7.22–7.18 (m, 4H), 7.12–7.09 (m, 2H), 6.33 (d, *J* = 8.4 Hz, 2H), 4.21 (brs, 1H), 4.06 (s, 2H); ^{13}C NMR (100 MHz, $CDCl_3$) δ 151.20, 145.66, 145.33, 139.39, 131.59, 128.69, 128.54, 128.38, 128.07, 127.91, 127.55, 115.29, 109.67, 107.46 51.88; HRMS (ESI-TOF) *m/z* Calcd for $C_{21}H_{18}NBrI$ $[M+H]^+$: 489.9662, found: 489.9661.

2.1.2 Preparation of Unactivated Alkenes

Allyl phenol derivatives **2m**, **2o**, **2p**, **2q**, **2r**, **2s**, **2t**, **2u**, **2v**, **2w** were prepared according to the following procedure.

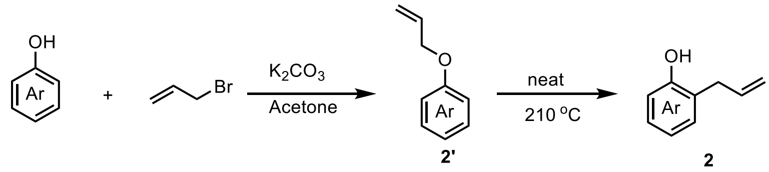

A round bottle was charged with phenol derivatives (1.0 equiv.), allyl bromide (1.2 equiv.), K_2CO_3 (2.0 equiv.) and acetone (0.25 M), After the mixture was stirring at 80 °C overnight, the mixture was quenched with H_2O and

extracted with DCM for three times. The combined organic layers were washed with brine, dried over Na_2SO_4, filtered and the solvent was evaporated under reduced pressure. The crude product **2'** was added in a sealed tube, and stirred at 210 °C oil bath for 16 h. The crude product was purified by column chromatography on silica gel.

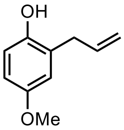

2m

2m was prepared according to above procedure using 4-methoxyphenol (821.0 mg, 5.0 mmol, 1.0 equiv.), allyl bromide (0.5 mL, 6.0 mmol, 1.2 equiv.), K_2CO_3 (1.4 g, 10.0 mmol, 2.0 equiv.). Purification by flash chromatography on silica gel (PE/EA= 50/1) gave the product as a colorless liquid (374.6 mg, 47% yield)[10]. ^{1}H NMR (400 MHz, $CDCl_3$) δ 6.76–6.74 (m, 1H), 6.68–6.66 (m, 2H), 6.00 (ddt, *J* = 17.6, 10.0, 6.4 Hz, 1H), 5.18–5.13 (m, 2H), 4.60 (s, 1H), 3.75 (s, 3H), 3.38 (d, *J* = 6.4, Hz, 2H).

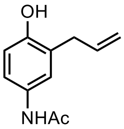

2o

2o was prepared according to above procedure using *N*-(4-hydroxyphenyl)acetamide (1.5 g, 10.0 mmol, 1.0 equiv.), allyl bromide (1.0 mL, 12.0 mmol, 1.2 equiv.), K_2CO_3 (2.8 g, 20.0 mmol, 2.0 equiv.). Purification by flash chromatography on silica gel (PE/EA= 2/1) gave the product as a white solid (1.3 g, 67% yield). ^{1}H NMR (400 MHz, $CDCl_3$) δ 7.22–7.19 (m, 2H), 7.14 (brs, 1H), 6.74 (d, *J* = 8.2 Hz, 1H), 5.98 (ddt, *J* = 18.0, 9.6, 6.4 Hz, 1H), 5.50 (brs, 1H), 5.17–5.12 (m, 2H), 3.37 (d, *J* = 5.2 Hz, , 2H), 2.14 (s, 3H); ^{13}C NMR (100 MHz, $CDCl_3$) δ 168.46, 151.27, 136.09, 130.60, 126.08, 122.97, 120.49, 116.60, 116.05, 34.93, 24.29; HRMS (ESI-TOF) *m/z* Calcd for $C_{11}H_{14}NO_2$ $[M+H]^+$: 192.1019, found: 192.1017.

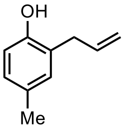

2p

2p was prepared according to above procedure using *p*-cresol (0.5 mL, 5.0 mmol, 1.0 equiv.), allyl bromide (0.5 mL, 6.0 mmol, 1.2 equiv.), K_2CO_3 (1.4 g, 10.0 mmol, 2.0 equiv.). Purification by flash chromatography on silica gel (PE/EA= 10/1) gave the product as a colorless liquid (531.5 mg, 72% yield)[11]. ^{1}H NMR (400 MHz, $CDCl_3$) δ 6.94–6.92 (m, 2H), 6.71 (d, *J* = 7.6 Hz, 1H), 6.01 (ddt, *J* = 16.8, 10.4, 6.4 Hz, 1H), 5.19–5.13 (m, 2H), 4.78 (s, 1H), 3.38 (dt, *J* = 6.4, 1.6 Hz, 2H), 2.26 (s, 3H).

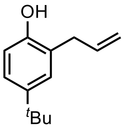

2q

2q was prepared according to above procedure using 4-(tert-butyl)phenol (751.1 mg, 5.0 mmol, 1.0 equiv.), allyl

bromide (0.5 mL, 6.0 mmol, 1.2 equiv.), K_2CO_3 (1.4 g, 10.0 mmol, 2.0 equiv.). Purification by flash chromatography on silica gel (PE/EA= 20/1) gave the product as a colorless liquid (677.7 mg, 68% yield)[12]. ^{1}H NMR (400 MHz, $CDCl_3$) δ 7.15 (dd, *J* = 8.4, 2.4 Hz, 1H), 7.11 (d, *J* = 2.4 Hz, 1H), 6.75 (d, *J* = 8.4 Hz, 1H), 6.03 (ddt, *J* = 16.8, 10.0, 6.4 Hz, 1H), 5.21–5.14 (m, 2H), 4.82 (s, 1H), 3.41 (dt, *J* = 6.4, 1.6 Hz, 2H), 1.29 (s, 9H).

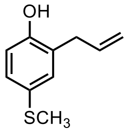

2r

2r was prepared according to above procedure using 4-(tert-butyl)phenol (701.0 mg, 5.0 mmol, 1.0 equiv.), allyl bromide (0.5 mL, 6.0 mmol, 1.2 equiv.), K_2CO_3 (1.4 g, 10.0 mmol, 2.0 equiv.). Purification by flash chromatography on silica gel (PE/EA= 30/1) gave the product as a yellow liquid (91.2 mg, 11% yield). ^{1}H NMR (400 MHz, $CDCl_3$) δ 7.13–7.10 (m, 2H), 6.78–6.75 (m, 1H), 6.00 (ddt, *J* = 17.6, 9.6, 6.4 Hz, 1H), 5.20–5.14 (m, 2H), 4.94 (brs, 1H), 3.39 (dt, *J* = 6.4, 1.6 Hz, 2H), 2.44 (s, 3H); ^{13}C NMR (100 MHz, $CDCl_3$) δ 152.69, 135.94, 130.97, 128.81, 128.35, 126.19, 116.76, 116.50, 35.00, 17.99; HRMS (ESI-TOF) *m/z* Calcd for $C_{10}H_{11}SO$ $[M-H]^+$: 179.0525, found: 179.0532.

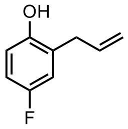

2s

2s was prepared according to above procedure using 4-fluorophenol (560.5 mg, 5.0 mmol, 1.0 equiv.), allyl bromide (0.5 mL, 6.0 mmol, 1.2 equiv.), K_2CO_3 (1.4 g, 10.0 mmol, 2.0 equiv.). Purification by flash chromatography on silica gel (PE/EA= 20/1) gave the product as a colorless liquid (483.6 mg, 64% yield)[12]. ^{1}H NMR (400 MHz, $CDCl_3$) δ 6.85–6.79 (m, 2H), 6.76–6.73 (m, 1H), 5.99 (ddt, *J* = 16.8, 10.4, 6.4 Hz, 1H), 5.20–5.14 (m, 2H), 4.76 (brs, 1H), 3.38 (dt, *J* = 6.4, 1.6 Hz, 2H).

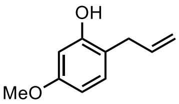

2t

2t was prepared according to above procedure using 3-methoxyphenol (0.6 mL, 5.0 mmol, 1.0 equiv.), allyl bromide (0.5 mL, 6.0 mmol, 1.2 equiv.), K_2CO_3 (1.4 g, 10.0 mmol, 2.0 equiv.). Purification by flash chromatography on silica gel (PE/EA= 50/1) gave the product as a colorless liquid (448.6 mg, 55% yield)[12]. ^{1}H NMR (400 MHz, $CDCl_3$) δ 6.99 (d, *J* = 8.4 Hz, 1H), 6.46 (dd, *J* = 8.4, 2.4 Hz, 1H), 6.42 (d, *J* = 2.4 Hz, 1H), 6.05–5.95 (m, 1H), 5.19–5.13 (m, 2H), 5.02 (brs, 1H), 3.77 (s, 3H), 3.35 (dt, *J* = 6.4, 1.6 Hz, 2H).

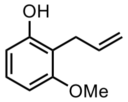

2u

2u was prepared according to above procedure using 3-methoxyphenol (0.6 mL, 5.0 mmol, 1.0 equiv.), allyl bromide (0.5 mL, 6.0 mmol, 1.2 equiv.), K_2CO_3 (1.4 g, 10.0 mmol, 2.0 equiv.). Purification by flash chromatography on silica gel (PE/EA= 50/1) gave the product as a colorless liquid (271.5 mg, 33% yield)[10]. ^{1}H NMR (400 MHz, $CDCl_3$) δ 7.08 (t, *J* = 8.0 Hz, 1H), 6.51–6.48 (m, 2H), 5.98 (ddt, *J* = 17.6, 10.4, 6.0 Hz, 1H), 5.14–5.06 (m, 2H), 4.97 (brs, 1H), 3.81 (s, 3H), 3.46 (dt, *J* = 6.0, 1.6 Hz, 2H).

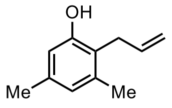

2v

2v was prepared according to above procedure using 3,5-dimethylphenol (610.8 mg, 5.0 mmol, 1.0 equiv.), allyl bromide (0.5 mL, 6.0 mmol, 1.2 equiv.), K_2CO_3 (1.4 g, 10.0 mmol, 2.0 equiv.). Purification by flash chromatography on silica gel (PE/EA= 50/1) gave the product as a white solid (549.0 mg, 81% yield)[12]. ^{1}H NMR (400 MHz, $CDCl_3$) δ 6.60 (s, 1H), 6.50 (s, 1H), 5.95 (ddt, *J* = 17.2, 10.4, 6.0 Hz, 1H), 5.07–4.99 (m, 2H), 4.73 (brs, 1H), 3.38 (dt, *J* = 6.0, 1.6 Hz, 2H), 2.25 (s, 3H), 2.24 (s, 3H).

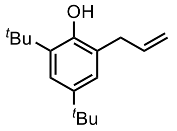

2w

2w was prepared according to above procedure using 2,4-di-*tert*-butylphenol (1.0 g, 5.0 mmol, 1.0 equiv.), allyl bromide (0.5 mL, 6.0 mmol, 1.2 equiv.), K_2CO_3 (1.4 g, 10.0 mmol, 2.0 equiv.). Purification by flash chromatography on silica gel (PE) gave the product as a colorless liquid (1.0 g, 84% yield)[14]. ^{1}H NMR (400 MHz, $CDCl_3$) δ 7.22 (d, *J* = 2.4 Hz, 1H), 6.97 (d, *J* = 2.4 Hz, 1H), 6.04 (ddt, *J* = 16.8, 10.0, 6.4 Hz, 1H), 5.29–5.21 (m, 2H), 5.09 (s, 1H), 3.42 (dt, *J* = 6.4, 1.6 Hz, 2H), 1.41 (s, 9H), 1.30 (s, 9H).

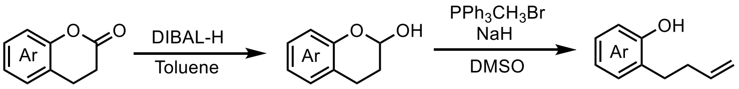

Chroman-2-one derivates (1.0 equiv.) in dry toluene (0.2 M) under a nitrogen atmosphere was added dropwise with diisobutylaluminum hydride (1.5 M in toluene, 1.1 equiv.) at -78 ^{o}C. After a further 2 h at -78 ^{o}C, H_2O was added slowly to the stirred. The mixture was allowed to warm to room temperature and more water was added, followed by the addition of Et_2O. The mixture was filtered through a pad of celite, and the filter cake was further washed with ether. The filtrate was washed with brine and dried over Na_2SO_4. Removal of solvent gave **6'** as a colorless oil.

Under a nitrogen atmosphere, a 60% dispersion of NaH (2.1 equiv.) in oil was washed with dry ether, and then treated with DMSO (1.5 M). The mixture was heated at 80°C until gas evolution ceased. To the deep gray solution was added methyl triphenylphosphonium bromide (2.1 equiv.) in DMSO (1.5 M) at room temperature. After

stirring for 15 min, a solution of **6'** (1.0 equiv.) in DMSO (5.0 M) was added. After stirring for an another 30 min, the reaction mixture was quenched with H_2O. The solution was further neutralized with 10% HCl (aq.) and extracted with ether for several times. The combined organic layers were washed with brine, dried over Na_2SO_4, filtered and concentrated under vacuum. The crude product was purified by column chromatography on silica gel. Substrates **6a, 6k,** and **6l** were prepared according to the above procedure[16].

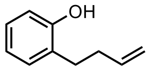

6a

6a was prepared according to above procedure using chroman-2-one (6.2 mL, 50.0 mmol, 1.0 equiv.). Purification by flash chromatography on silica gel (PE/EA= 20/1) gave the product as a colorless liquid (5.9 g, 79% yield)[16]. ^{1}H NMR (400 MHz, $CDCl_3$) δ 7.13–7.06 (m, 2H), 6.87 (t, *J* = 7.6 Hz, 1H), 6.75 (d, *J* = 7.6 Hz, 1H), 5.90 (ddt, *J* = 16.8, 9.6, 6.8 Hz, 1H), 5.09–4.99 (m, 2H), 4.67 (brs, 1H), 2.71 (t, *J* = 7.6 Hz, 2H), 2.38 (q, *J* = 7.6 Hz, 2H).

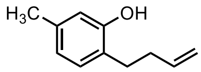

6k

6k was prepared according to above procedure using 7-methylchroman-2-one (4.0 g, 25.7 mmol, 1.0 equiv.). Purification by flash chromatography on silica gel (PE/EA= 10/1) gave the product as a light-yellow liquid (90.4 mg, 3% yield). ^{1}H NMR (400 MHz, $CDCl_3$) δ 7.00 (d, *J* = 7.6 Hz, 1H), 6.69 (d, *J* = 7.6 Hz, 1H), 6.59 (s, 1H), 5.88 (dt, *J* = 17.2, 8.4 Hz, 1H), 5.09–5.98 (m, 2H), 4.60 (brs, 1H), 2.67 (t, *J* = 7.6 Hz, 2H), 2.35 (q, *J* = 7.6 Hz, 2H), 2.27 (s, 3H); ^{13}C NMR (100 MHz, $CDCl_3$) δ 153.24, 138.46, 137.14, 130.08, 121.45, 116.16, 114.99, 114.95, 33.98, 29.22, 20.93; HRMS (EI-TOF) *m/z* Calcd for $C_{11}H_{14}O$ $[M]^+$: 162.1039, found: 162.1042.

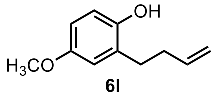

6l

6l was prepared according to above procedure using 6-methoxychroman-2-one (1.8 g, 10.0 mmol, 1.0 equiv.). Purification by flash chromatography on silica gel (PE/EA= 10/1) gave the product as a light-yellow liquid (89.6 mg, 6% yield). ^{1}H NMR (400 MHz, $CDCl_3$) δ 6.70 (dd, *J* = 6.0, 2.4 Hz, 2H), 6.64–6.62 (m, 1H), 5.90 (dt, *J* = 17.2, 8.8 Hz, 1H), 5.10–4.99 (m, 2H), 4.39 (brs, 1H), 3.76 (s, 3H), 2.68 (t, *J* = 7.6 Hz, 2H), 2.37 (q, *J* = 6.8 Hz, 2H). ^{13}C NMR (100 MHz, $CDCl_3$) δ 153.54, 147.44, 138.13, 129.07, 115.93, 115.13, 111.84, 55.72, 33.76, 29.84; HRMS (EI-TOF) *m/z* Calcd for $C_{11}H_{14}O_2$ $[M]^+$: 178.0988, found: 178.0992.

2.2 Experimental Optimization

2.2.1 Evaluation of Pallidum Sources[a,b]

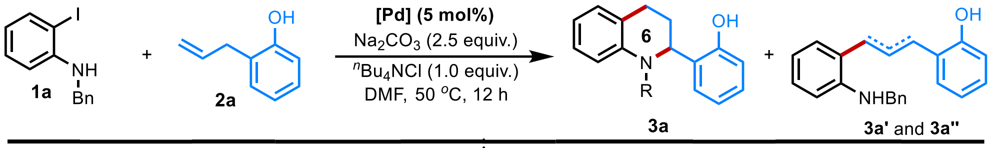

Entry	[Pd]	3a (%)	3a' + 3a'' (%)	1a (%)	Entry	[Pd]	3a (%)	3a' + 3a'' (%)	1a (%)
1	$Pd_2(dba)_3$	66	28	-	5	$Pd(PPh_3)_2Cl_2$	-	-	100
2	$Pd(PPh_3)_4$	52	44	-	6	$[(allyl)PdCl]_2$	-	-	92
3	$Pd(OAc)_2$	10	61	-	7	$Pd(dppp)Cl_2$	-	-	100
4	$PdCl_2$	20	31	-	8	$Pd(dppf)Cl_2$	-	58	33

[a]Reaction conditions: **1a** (0.1 mmol, 1.0 equiv.), **2a** (0.2 mmol, 2.0 equiv), Na_2CO_3 (0.25 mmol, 2.5 equiv.), ^{n}Bu$_4$NCl (0.1 mmol, 1.0 equiv.), [Pd] (5 mol%), N_2, 50 °C, 12 h, DMF (1.0 mL). [b]Yield was determined by ^{1}H NMR using pyrene as the internal standard.

2.2.2 Loading of Pallidum Catalyst[a,b]

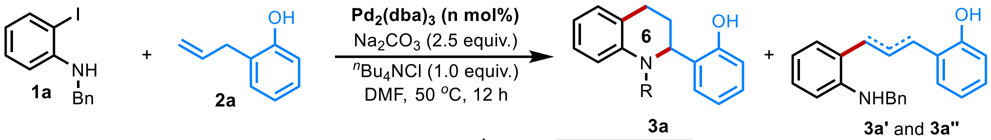

Entry	n	3a (%)	3a' + 3a'' (%)	1a (%)	Entry	n	3a (%)	3a' + 3a'' (%)	1a (%)
1	0.5	68	27	5	5	2.5	66	28	-
2	1.0	70	30	-	6	3.0	34	36	-
3	1.5	76	24	-	7	4.0	32	48	-
4	2.0	68	24	-	8	5.0	34	40	-

[a]Reaction conditions: **1a** (0.1 mmol, 1.0 equiv.), **2a** (0.2 mmol, 2.0 equiv.), Na_2CO_3 (0.25 mmol, 2.5 equiv.), ^{n}Bu$_4$NCl (0.1 mmol, 1.0 equiv.), N_2, 50 °C, 12 h, DMF (1.0 mL). [b]Yield was determined by ^{1}H NMR using pyrene as an internal standard.

2.2.3 Loading of (^{n}Bu)$_4$NCl[a,b]

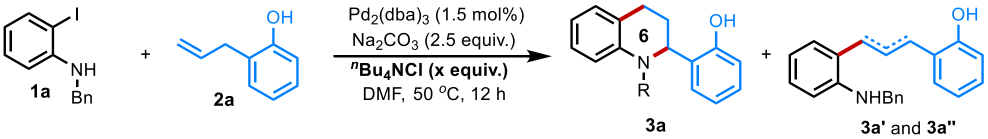

Entry	x	3a (%)	3a' + 3a'' (%)	1a (%)	Entry	x	3a (%)	3a' + 3a'' (%)	1a (%)
1	0.1	36	42	-	5	1.5	76	23	-
2	0.5	44	34	-	6	2.0	80	20	-
3	0.7	60	27	-	7	2.5	66	26	-
4	1.0	76	24	-	8	3.0	40	19	41

[a]Reaction conditions: **1a** (0.1 mmol, 1.0 equiv.), **2a** (0.2 mmol, 2.0 equiv.), Na_2CO_3 (0.25 mmol, 2.5 equiv.), $Pd_2(dba)_3$ (0.0015 mmol, 1.5 mol%), N_2, 50 °C, 12 h, DMF (1.0 mL). [b]Yield was determined by ^{1}H NMR using pyrene as an internal standard.

2.2.4 Evaluation of Additives[a,b]

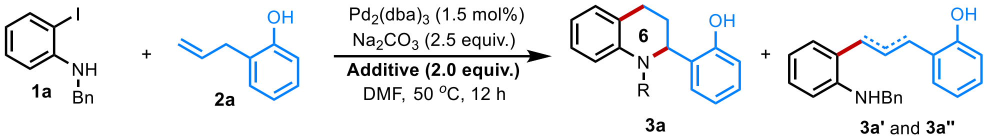

Entry	Additive	**3a** (%)	**3a'** + **3a''** (%)	**1a** (%)	Entry	Additive	**3a** (%)	**3a'** + **3a''** (%)	**1a** (%)
1	$^nBu_4NCl$	80	20	-	5	Et_4NI	34	41	-
2	Et_4NCl	64	35	-	6	NH_4Cl	12	11	75
3	$(CH_3)_4NCl$	56	32	-	7	NaCl	42	33	-
4	Et_4NBr	58	36	-	8	LiCl	2	69	29

[a]Reaction conditions: **1a** (0.1 mmol, 1.0 equiv.), **2a** (0.2 mmol, 2.0 equiv.), Na_2CO_3 (0.25 mmol, 2.5 equiv.), Additive (0.2 mmol, 2.0 equiv.), $Pd_2(dba)_3$ (0.0015 mmol, 1.5 mol%), N_2, 50 °C, 12 h, DMF (1.0 mL). [b]Yield was determined by ^1H NMR using pyrene as an internal standard.

2.2.5 Evaluation of Ligands[a,b]

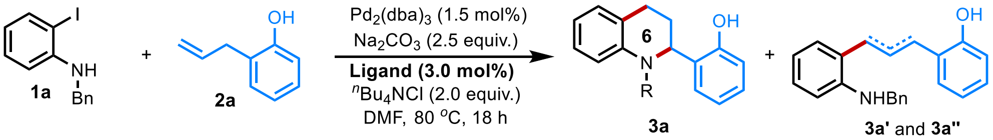

Ligand =

no Ligand

68% **3a**
32% **3a'** + **3a''**

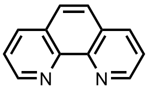

L1

40% **3a'** + **3a''**

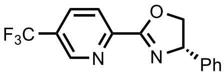

L2

56% **3a**
43% **3a'** + **3a''**

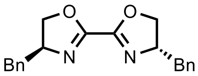

L3

84% **3a**
16% **3a'** + **3a''**

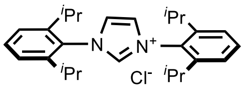

L4

70% **3a**
15% **3a'** + **3a''**

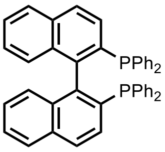

(±)-**L5**

ND

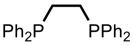

L6

ND

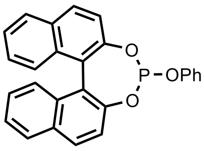

(±)-**L7**

84% **3a**
13% **3a'** + **3a''**

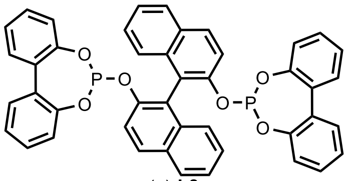

(±)-**L8**

90% **3a**
<10% **3a'** + **3a''**

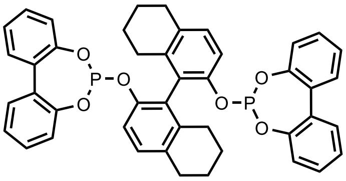

(±)-**L9**

66% **3a**
19% **3a'** + **3a''**

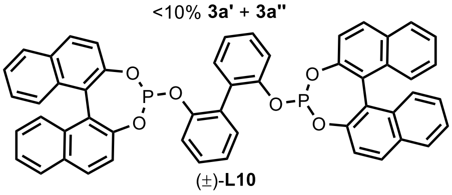

(±)-**L10**

80% **3a**
<10% **3a'** + **3a''**

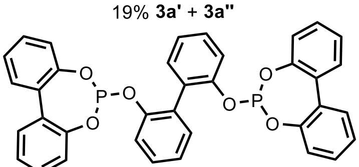

L11

72% **3a**
17% **3a'** + **3a''**

[a]Reaction conditions: **1a** (0.1 mmol, 1.0 equiv.), **2a** (0.2 mmol, 2.0 equiv.), Na_2CO_3 (0.25 mmol, 2.5 equiv.), $^nBu_4NCl$ (0.2 mmol, 2.0 equiv.), $Pd_2(dba)_3$ (0.0015 mmol, 1.5 mol%), **L1~L6**, **L8-L11** (0.003 mmol, 3.0 mol%), **L7** (0.006 mmol, 6.0 mol%), N_2, 80 °C, 18 h, DMF (1.0 mL). [b]Yield was determined by ^1H NMR using dibromomethane as an internal standard.

2.2.6 Control Experiments[a,b]

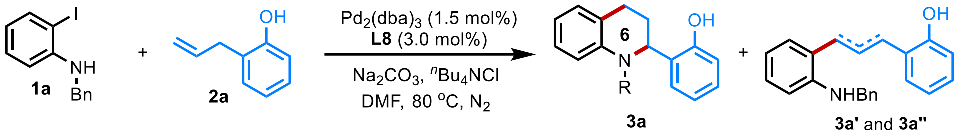

Entry	Deviation from conditions	**3a** (%)	**3a'** + **3a''** (%)
1	none	90	<10
2	without Na_2CO_3	ND	ND
3	without ^{n}Bu$_4$NCl	40	44
4	without **L8**	68	32
5	^{n}Bu$_4$NBr instead of ^{n}Bu$_4$NCl	64	24
6	^{n}Bu$_4$NI instead of ^{n}Bu$_4$NCl	32	30
7	^{n}Pr$_4$NCl instead of ^{n}Bu$_4$NCl	74	22

[a]Reaction conditions: **1a** (0.1 mmol, 1.0 equiv.), **2a** (0.2 mmol, 2.0 equiv.), Na_2CO_3 (0.25 mmol, 2.5 equiv.), ^{n}Bu$_4$NCl (0.2 mmol, 2.0 equiv.), $Pd_2(dba)_3$ (0.0015 mmol, 1.5 mol%), **L8** (0.003 mmol, 3.0 mol%), N_2, 80 °C, 18 h, DMF (1.0 mL). [b]Yield was determined by ^{1}H NMR using dibromomethane as an internal standard.

2.3 General Procedure for Pd-Catalyzed Migratory Cycloannulation Reaction

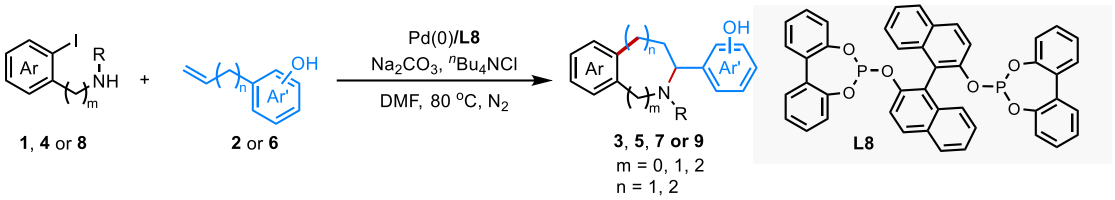

General Procedure for Pd-Catalyzed Migratory Cycloannulation Reaction: $Pd_2(dba)_3$ (2.8 mg, 1.5 mol%), **L8** (4.3 mg, 3.0 mol%), Na_2CO_3 (53.0 mg, 0.5 mmol) and $^{n}Bu_4NCl$ (111.2 mg, 0.4 mmol) were added to a 10 mL vial in dry box. The tube was sealed using a cap with PTFE cap liner and moved outside of the glovebox. DMF (3.0 mL) was added followed by addition of aniline derivatives **1**, **4** or **8** (0.4 mmol) and alkene **2** or **6** (0.2 mmol). The reaction mixture was stirred at 80 °C for 18 h. After cooling to room temperature, the reaction mixture was diluted with ethyl acetate, and the resulted solution was washed with brine for three times. The organic phase was concentrated, and the residue was then purified by silica gel chromatography or preparative thin-layer chromatography.

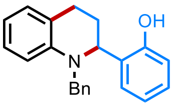

3a

2-(1-Benzyl-1,2,3,4-tetrahydroquinolin-2-yl)phenol

3a was synthesized following the procedure. After purification by flash column chromatography using PE/EA (15/1) as the eluent, **3a** was obtained in 80% yield (50.6 mg) as a yellow oil. ^{1}H NMR (400 MHz, $CDCl_3$) δ 7.74 (brs, 1H), 7.24–7.06 (m, 7H), 7.02–6.94 (m, 3H), 6.86–6.80 (m, 2H), 6.76 (t, J = 7.2 Hz, 1H), 4.86 (d, J = 16.4 Hz, 1H), 4.54 (dd, J = 8.0, 4.4 Hz, 1H), 4.17 (d, J = 16.4 Hz, 1H), 2.77–2.62 (m, 2H), 2.32–2.23 (m 1H), 2.16–2.07 (m, 1H); ^{13}C NMR (100 MHz, $CDCl_3$) δ 155.01, 144.48, 136.99, 129.26, 128.58, 128.44, 128.41, 127.68, 127.38, 127.30, 127.10, 125.33, 120.18, 118.63, 116.41, 115.17, 60.05, 52.96, 28.50, 26.20; HRMS (ESI-TOF) *m/z* Calcd for $C_{22}H_{22}NO$ $[M+H]^+$: 316.1696, found: 316.1702.

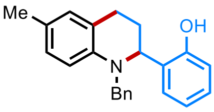

3b

2-(1-Benzyl-6-methyl-1,2,3,4-tetrahydroquinolin-2-yl)phenol

3b was synthesized following the procedure. After purification by flash column chromatography using PE/EA (15/1) as the eluent, **3b** was obtained in 73% yield (48.3 mg) as a yellow oil. ^{1}H NMR (400 MHz, $CDCl_3$) δ 8.31 (brs, 1H), 7.25–7.20 (m, 3H), 7.16 (td, J = 7.6, 1.6 Hz, 1H), 7.07–7.04 (m, 2H), 6.98–6.92 (m, 3H), 6.86–6.82 (m, 3H), 4.85 (d, J = 16.0 Hz, 1H), 4.44–4.40 (m, 1H), 4.14 (d, J = 16.2 Hz, 1H), 2.76–2.59 (m, 2H), 2.31–2.21 (m, 4H), 2.12–2.04 (m, 1H); ^{13}C NMR (100 MHz, $CDCl_3$) δ 155.59, 141.92, 136.88, 129.97, 128.59, 128.44, 128.38,

128.07, 127.95, 127.15, 126.97, 125.93, 120.02, 116.56, 116.25, 60.73, 53.27, 28.86, 26.61, 20.33; HRMS (ESI-TOF) *m/z* Calcd for $C_{23}H_{24}NO$ [M+H]$^+$: 330.1852, found: 333.1847.

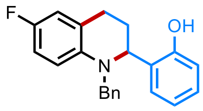

3c

2-(1-Benzyl-6-fluoro-1,2,3,4-tetrahydroquinolin-2-yl)phenol

3c was synthesized following the procedure. After purification by flash column chromatography using PE/EA (15/1) as the eluent, **3c** was obtained in 64% yield (42.8 mg) as a yellow oil. ^{1}H NMR (400 MHz, $CDCl_3$) δ 8.09 (brs, 1H), 7.23 (d, *J* = 6.4 Hz, 3H), 7.18–7.14 (m, 1H), 7.05 (dd, *J* = 7.2, 2.8 Hz, 2H), 6.97 (d, *J* = 7.6 Hz, 1H), 6.91–6.78 (m, 4H), 6.73 (dd, *J* = 8.8, 2.8 Hz, 1H), 4.77 (d, *J* = 16.4 Hz, 1H), 4.48 (dd, *J* = 8.4, 4.0 Hz, 1H), 4.16 (d, *J* = 16.8 Hz, 1H), 2.73–2.59 (m, 2H), 2.31–2.22 (m, 1H), 2.14–2.08 (m, 1H); ^{13}C NMR (100 MHz, $CDCl_3$) δ 156.26 (d, *J* = 238 Hz), 155.27, 140.54, 136.66, 128.54, 128.49, 128.46, 127.88, 127.4 (d, *J* = 7.6 Hz), 127.28, 126.85 (d, *J* = 2.8 Hz), 120.16, 116.81 (d, *J* = 7.0 Hz), 116.53, 115.56 (d, *J* = 22 Hz),113.77 (d, *J* = 22.5 Hz), 60.26, 53.79, 28.23, 26.46; ^{19}F NMR (375 MHz, $CDCl_3$) δ -125.82; HRMS (ESI-TOF) *m/z* Calcd for $C_{22}H_{21}NOF$ [M+H]$^+$: 334.1602, found: 334.1607.

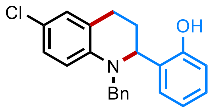

3d

2-(1-Benzyl-6-chloro-1,2,3,4-tetrahydroquinolin-2-yl)phenol

3d was synthesized following the procedure. After purification by flash column chromatography using PE/EA (15/1) as the eluent, **3d** was obtained in 71% yield (49.5 mg) as a yellow solid. ^{1}H NMR (400 MHz, $CDCl_3$) δ 7.28–7.22 (m, 3H), 7.15 (t, *J* = 7.6 Hz, 1H), 7.09–6.96 (m, 6H), 6.85 (t, *J* = 7.6 Hz, 1H), 6.78 (dd, *J* = 12.0, 8.4 Hz, 2H), 4.76 (d, *J* = 16.8 Hz, 1H), 4.63 (dd, *J* = 6.8, 4.4 Hz, 1H), 4.18 (d, *J* = 16.8 Hz, 1H), 2.64 (td, *J* = 8.0, 7.2, 3.6 Hz, 2H), 2.30–2.22 (m, 1H), 2.17–2.09 (m, 1H); ^{13}C NMR (100 MHz, $CDCl_3$) δ 154.42, 143.24, 136.85, 128.77, 128.57, 128.47, 128.43, 127.37, 127.30, 127.18, 127.11, 126.27, 122.67, 120.37, 116.29, 115.32, 59.28, 53.02, 27.82, 25.62; HRMS (ESI-TOF) *m/z* Calcd for $C_{22}H_{21}NOCl$ [M+H]$^+$: 350.1306, found: 350.1314.

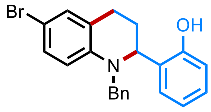

3e

2-(1-Benzyl-6-bromo-1,2,3,4-tetrahydroquinolin-2-yl)phenol

3e was synthesized following the procedure. After purification by flash column chromatography using PE/EA (15/1) as the eluent, **3e** was obtained in 75% yield (59.2 mg) as a yellow solid. ^{1}H NMR (400 MHz, $CDCl_3$) δ 7.29–7.21 (m, 3H), 7.18–7.12 (m, 3H), 7.10–7.08 (m, 2H), 6.98 (dd, *J* = 7.6, 1.6 Hz, 1H), 6.88–6.84 (m, 2H), 6.81–6.79 (m, 1H), 6.71 (d, *J* = 8.8 Hz, 1H), 4.75 (d, *J* = 16.8 Hz, 1H), 4.65 (dd, *J* = 6.8, 4.4 Hz, 1H), 4.19 (d, *J* = 16.8 Hz, 1H),

2.71–2.60 (m, 2H), 2.29–2.22 (m, 1H), 2.17–2.09 (m, 1H); ^{13}C NMR (100 MHz, $CDCl_3$) δ 154.33, 143.77, 136.86, 131.61, 130.03, 128.60, 128.49, 128.45, 127.41, 127.24, 127.19, 126.65, 120.43, 116.29, 115.60, 109.86, 59.19, 52.91, 27.77, 25.52; HRMS (ESI-TOF) *m/z* Calcd for $C_{22}H_{21}NOBr$ $[M+H]^+$: 394.0801, found: 394.0808.

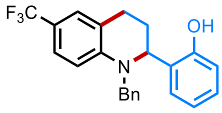

3f

2-(1-Benzyl-6-(trifluoromethyl)-1,2,3,4-tetrahydroquinolin-2-yl)phenol

3f was synthesized following the procedure. After purification by flash column chromatography using PE/EA (15/1) as the eluent, **3f** was obtained in 73% yield (56.2 mg) as a yellow solid. ^{1}H NMR (400 MHz, $CDCl_3$) δ 7.31–7.22 (m, 5H), 7.15 (d, *J* = 7.6 Hz, 3H), 7.01–6.98 (m, 1H), 6.87 (t, *J* = 7.6 Hz, 1H), 6.76 (d, *J* = 8.0 Hz, 1H), 6.70 (d, *J* = 8.6 Hz, 1H), 5.70 (brs, 1H), 4.93–4.90 (m, 1H), 4.76 (d, *J* = 17.2 Hz, 1H), 4.26 (d, *J* = 17.2 Hz, 1H), 2.72–2.62 (m, 2H), 2.32–2.27 (m 1H), 2.22–2.16 (m, 1H); ^{13}C NMR (100 MHz, $CDCl_3$) δ 153.31, 147.63, 137.04, 128.75, 128.41, 128.24, 128.09, 127.14, 126.52, 125.88 (q, *J* = 3.8 Hz), 125.04 (q, *J* = 270.4 Hz),124.65 (q, *J* = 3.8 Hz), 122.82, 120.66, 118.14 (q, *J* = 32.6 Hz), 115.90, 111.20, 57.77, 52.73, 26.79, 24.49; ^{19}F NMR (375 MHz, $CDCl_3$) δ -60.87; HRMS (ESI-TOF) *m/z* Calcd for $C_{23}H_{21}NOF_3$ $[M+H]^+$: 384.1570, found: 384.1575.

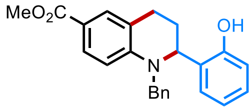

3g

Methyl-1-benzyl-2-(2-hydroxyphenyl)-1,2,3,4-tetrahydroquinoline-6-carboxylate

3g was synthesized following the procedure. After purification by flash column chromatography using PE/EA (10/1) as the eluent, **3g** was obtained in 61% yield (45.2 mg) as a yellow solid. ^{1}H NMR (400 MHz, $CDCl_3$) δ 7.75–7.70 (m, 2H), 7.32–7.28 (m, 2H), 7.24–7.22 (m, 1H), 7.17–7.14 (m, 3H), 6.99 (dd, *J* = 7.6, 1.6 Hz, 1H), 6.89–6.85 (m, 1H), 6.80–6.78 (m, 1H), 6.65 (d, *J* = 8.4 Hz, 1H), 5.71 (brs, 1H), 4.97 (t, *J* = 4.4 Hz, 1H), 4.80 (d, *J* = 17.2 Hz, 1H), 4.26 (d, *J* = 17.2 Hz, 1H), 3.84 (s, 3H), 2.72–2.61 (m, 2H), 2.33–2.27 (m, 1H), 2.23–2.15 (m, 1H); ^{13}C NMR (100 MHz, $CDCl_3$) δ 167.66, 153.25, 149.23, 137.09, 130.59, 129.75, 128.75, 128.36, 128.17, 128.11, 127.10, 126.40, 122.01, 120.55, 117.37, 115.81, 110.61, 57.53, 52.73, 51.59, 26.61, 24.15; HRMS (ESI-TOF) *m/z* Calcd for $C_{24}H_{24}NO_3$ $[M+H]^+$: 374.1751, found: 374.1756.

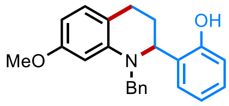

3h

2-(1-Benzyl-7-methoxy-1,2,3,4-tetrahydroquinolin-2-yl)phenol

3h was synthesized following the procedure. After purification by flash column chromatography using PE/EA (10/1) as the eluent, **3h** was obtained in 43% yield (29.7 mg) as a yellow oil. ^{1}H NMR (400 MHz, $CDCl_3$) δ 7.35 (brs, 1H), 7.27–7.19 (m, 3H), 7.18–7.13 (m, 1H), 7.12–7.10 (m, 2H), 7.00–6.97 (m, 1H), 6.92 (dd, *J* = 8.4, 1.2 Hz,

1H), 6.86–6.80 (m, 2H), 6.48 (d, J = 2.4 Hz, 1H), 6.33 (dd, J = 8.4, 2.4 Hz, 1H), 4.80 (d, J = 16.8 Hz, 1H), 4.59–4.56 (m, 1H), 4.17 (d, J = 16.8 Hz, 1H), 3.72 (s, 3H), 2.67–2.60 (m, 2H), 2.29–2.20 (m, 1H), 2.15–2.08 (m, 1H); ^{13}C NMR (100 MHz, $CDCl_3$) δ 159.19, 154.76, 145.53, 137.17, 129.67, 128.58, 128.49, 128.37, 127.52, 127.07, 120.23, 117.69, 116.33, 103.21, 101.14, 59.68, 55.16, 53.07, 28.55, 25.19; HRMS (ESI-TOF) *m/z* Calcd for $C_{23}H_{24}NO_2$ $[M+H]^+$: 346.1802, found: 346.1804.

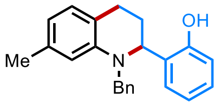

3i

2-(1-Benzyl-7-methyl-1,2,3,4-tetrahydroquinolin-2-yl)phenol

3i was synthesized following the procedure. After purification by flash column chromatography using PE/EA (10/1) as the eluent, **3i** was obtained in 75% yield (49.6 mg) as a yellow solid. ^1H NMR (400 MHz, $CDCl_3$) δ 7.88 (brs, 1H), 7.27–7.22 (m, 3H), 7.19–7.14 (m, 1H), 7.07 (dd, J = 7.2, 2.4 Hz, 2H), 6.96 (dd, J = 7.6, 1.6 Hz, 1H), 6.91 (d, J = 7.6 Hz, 1H), 6.86–6.81 (m, 3H), 6.60 (d, J = 7.6 Hz, 1H), 4.90 (d, J = 16.4 Hz, 1H), 4.46 (dd, J = 8.8, 4.4 Hz, 1H), 4.14 (d, J = 16.4 Hz, 1H), 2.74–2.59 (m, 2H), 2.30–2.21 (m, 4H), 2.13–2.06 (m, 1H); ^{13}C NMR (100 MHz, $CDCl_3$) δ 155.15, 144.33, 137.07, 136.94, 129.12, 128.55, 128.41, 128.35, 127.76, 127.23, 127.08, 122.53, 120.06, 119.61, 116.40, 115.90, 59.99, 52.96, 28.62, 25.87, 21.56; HRMS (ESI-TOF) *m/z* Calcd for $C_{23}H_{24}NO$ $[M+H]^+$: 330.1852, found: 330.1850.

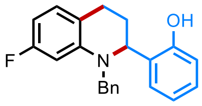

3j

2-(1-Benzyl-7-fluoro-1,2,3,4-tetrahydroquinolin-2-yl)phenol

3j was synthesized following the procedure. After purification by flash column chromatography using PE/EA (15/1) as the eluent, **3j** was obtained in 70% yield (46.5 mg) as a yellow oil. ^1H NMR (400 MHz, $CDCl_3$) δ 8.13 (brs, 1H), 7.28–7.23 (m, 3H), 7.17 (t, J = 7.6 Hz, 1H), 7.09–7.04 (m, 2H), 6.99–6.96 (m, 1H), 6.92–6.79 (m, 4H), 6.76–6.72 (m, 1H), 4.78 (d, J = 16.4 Hz, 1H), 4.50–4.47 (m, 1H), 4.17 (dd, J = 16.4, 2.4 Hz, 1H), 2.76–2.60 (m, 2H), 2.32–2.23 (m, 1H), 2.15–2.09 (m, 1H); ^{13}C NMR (100 MHz, $CDCl_3$) δ 156.38 (d, J = 238 Hz), 155.40, 140.56, 136.65, 128.59, 128.50, 127.98, 127.59 (d, J = 7.2 Hz), 127.32, 126.78 (J = 2.2 Hz), 120.18, 117.18 (d, J = 7.0 Hz), 116.63, 115.61 (d, J = 22 Hz), 115.57 (d, J = 22 Hz), 113.93, 113.71, 60.49, 53.84, 28.38, 26.62; ^{19}F NMR (375 MHz, $CDCl_3$) δ -125.80; HRMS (ESI-TOF) *m/z* Calcd for $C_{22}H_{21}NOF$ $[M+H]^+$: 334.1602, found: 334.1604.

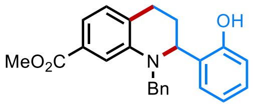

3k

Methyl-1-benzyl-2-(2-hydroxyphenyl)-1,2,3,4-tetrahydroquinoline-7-carboxylate

3k was synthesized following the procedure. After purification by flash column chromatography using PE/EA (10/1) as the eluent, **3k** was obtained in 68% yield (51.0 mg) as a yellow solid. ^{1}H NMR (400 MHz, $CDCl_3$) δ 7.61 (brs, 1H), 7.40 (dd, J = 7.6, 1.6 Hz, 1H), 7.25–7.22 (m, 3H), 7.19–7.15 (m, 1H), 7.11–7.05 (m, 4H), 6.98 (dd, J = 7.6, 1.6 Hz, 1H), 6.88–6.82 (m, 2H), 4.94 (d, J = 16.8 Hz, 1H), 4.67–4.64 (m, 1H), 4.19 (d, J = 16.8 Hz, 1H), 3.86 (s, 3H), 2.79–2.65 (m, 2H), 2.33–2.25 (m, 1H), 2.18–2.10 (m, 1H); ^{13}C NMR (100 MHz, $CDCl_3$) δ 167.51, 154.56, 144.77, 136.86, 129.90, 129.28, 128.54, 128.49, 128.46, 127.55, 127.34, 127.22, 120.34, 119.24, 116.36, 114.90, 59.03, 52.65, 52.01, 27.67, 26.02; HRMS (ESI-TOF) *m/z* Calcd for $C_{24}H_{24}NO_3$ $[M+H]^+$: 374.1751, found: 374.1756.

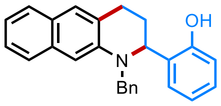

3l

2-(1-Benzyl-1,2,3,4-tetrahydrobenzo[g]quinolin-2-yl)phenol

3l was synthesized following the procedure. After purification by flash column chromatography using PE/EA (15/1) as the eluent, **3l** was obtained in 60% yield (44.0 mg) as a yellow solid. ^{1}H NMR (400 MHz, $CDCl_3$) δ 7.63 (d, J = 8.0 Hz, 1H), 7.54 (d, J = 8.0 Hz, 1H), 7.48 (s, 1H), 7.31–7.21 (m, 5H), 7.19–7.14 (m, 3H), 7.08 (s, 1H), 7.03 (dd, J = 7.6, 1.6 Hz, 1H), 6.85 (td, J = 7.6, 1.2 Hz, 1H), 6.80 (d, J = 8.0 Hz, 1H), 6.60 (brs, 1H), 4.98 (d, J = 16.8 Hz, 1H), 4.81 (t, J = 5.6 Hz, 1H), 4.28 (d, J = 16.8 Hz, 1H), 2.88 (t, J = 6.0 Hz, 2H), 2.36–2.20 (m, 2H); ^{13}C NMR (100 MHz, $CDCl_3$) δ 154.28, 143.56, 137.23, 134.06, 128.50, 128.59, 128.37, 127.87, 127.33, 127.19, 127.13, 127.00, 126.94, 126.75, 125.93, 125.49, 122.48, 120.44, 116.24, 107.47, 59.06, 52.99, 28.32, 26.00; HRMS (ESI-TOF) *m/z* Calcd for $C_{26}H_{24}NO$ $[M+H]^+$: 366.1852, found: 366.1852.

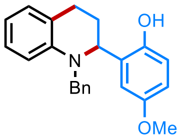

3m

2-(1-Benzyl-1,2,3,4-tetrahydroquinolin-2-yl)-4-methoxyphenol

3m was synthesized following the procedure. After purification by flash column chromatography using PE/EA (15/1) as the eluent, **3m** was obtained in 62% yield (43.0 mg) as a yellow solid. ^{1}H NMR (400 MHz, $CDCl_3$) δ 7.26–7.20 (m, 3H), 7.11–7.06 (m, 3H), 7.01 (dd, J = 7.2, 1.6 Hz, 1H), 6.91 (d, J = 8.0 Hz, 1H), 6.76–6.69 (m, 3H), 6.58 (t, J = 2.8 Hz, 1H), 4.85 (d, J = 16.8 Hz, 1H), 4.55–4.51 (m, 1H), 4.18 (dd, J = 16.8, 2.0 Hz, 1H), 3.70 (s, 3H), 2.76–2.63 (m, 2H), 2.32–2.23 (m, 1H), 2.16–2.09 (m, 1H); ^{13}C NMR (100 MHz, $CDCl_3$) δ 153.22, 148.74, 144.52, 137.16, 129.17, 128.44, 127.55, 127.38, 127.04, 125.01, 118.37, 116.88, 114.72, 114.39, 112.99, 59.95, 55.68, 52.94, 28.38, 26.06.; HRMS (ESI-TOF) *m/z* Calcd for $C_{23}H_{24}NO_2$ $[M+H]^+$: 346.1802, found: 346.1807.

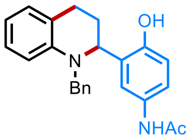

3o

N-(3-(1-Benzyl-1,2,3,4-tetrahydroquinolin-2-yl)-4-hydroxyphenyl)acetamide

3o was synthesized following the procedure. After purification by flash column chromatography using PE/EA (10/1) as the eluent, **3o** was obtained in 81% yield (52.8 mg) as a white solid. ^{1}H NMR (400 MHz, acetone-d_6) δ 9.10 (brs, 1H), 8.43 (brs, 1H), 7.73 (dd, J = 8.8, 2.8 Hz, 1H), 7.32–7.19 (m, 5H), 6.96–6.92 (m, 3H), 6.83 (dt, J = 8.8, 2.8 Hz, 1H), 6.54–6.48 (m, 2H), 5.06 (dd, J = 5.2, 2.4 Hz, 1H), 4.67 (d, J = 17.8 Hz, 1H), 4.19 (d, J = 17.8 Hz, 1H), 2.64–2.55 (m, 2H), 2.33–2.27 (m, 1H), 2.19–2.10 (m, 1H), 1.95 (s, 3H); ^{13}C NMR (100 MHz, acetone-d_6) δ 168.60, 151.19, 146.01, 139.62, 132.54, 130.51, 129.40, 129.37, 128.06, 127.40, 127.05, 122.60, 120.45, 120.00, 116.24, 116.09, 110.97, 57.07, 53.21, 27.18, 24.52, 23.94; HRMS (ESI-TOF) *m/z* Calcd for $C_{24}H_{25}N_2O_2$ [M+H]$^+$: 373.1911, found: 393.1915.

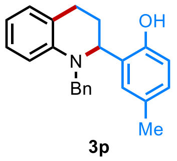

3p

2-(1-Benzyl-1,2,3,4-tetrahydroquinolin-2-yl)-4-methylphenol

3p was synthesized following the procedure. After purification by flash column chromatography using PE/EA (15/1) as the eluent, **3p** was obtained in 88% yield (57.8 mg) as a yellow oil. ^{1}H NMR (400 MHz, $CDCl_3$) δ 7.49 (brs, 1H), 7.23–7.18 (m, 3H), 7.12–7.06 (m, 3H), 7.03–7.00 (m, 1H), 6.98–6.95 (m, 2H), 6.79–6.71 (m, 3H), 4.87 (d, J = 16.8 Hz, 1H), 4.52–4.48 (m, 1H), 4.19 (dd, J = 16.8, 2.8 Hz, 1H), 2.79–2.64 (m, 2H), 2.32–2.23 (m, 4H), 2.15–2.07 (m, 1H); ^{13}C NMR (100 MHz, $CDCl_3$) δ 152.81, 144.50, 137.03, 129.31, 129.22, 129.04, 128.90, 128.44, 127.70, 127.38, 127.05, 125.49, 118.62, 116.30, 115.23, 60.33, 52.71, 28.85, 26.39, 20.59; HRMS (ESI-TOF) *m/z* Calcd for $C_{23}H_{24}NO$ [M+H]$^+$: 330.1852, found: 330.1852.

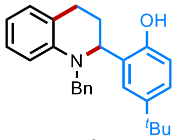

3q

2-(1-Benzyl-1,2,3,4-tetrahydroquinolin-2-yl)-4-(tert-butyl)phenol

3q was synthesized following the procedure. After purification by flash column chromatography using PE/EA (15/1) as the eluent, **3q** was obtained in 51% yield (38.0 mg) as a yellow oil. ^{1}H NMR (400 MHz, $CDCl_3$) δ 7.96 (brs, 1H), 7.24–7.18 (m, 4H), 7.15–7.11 (m, 1H), 7.06–7.02 (m, 4H), 6.94 (d, J = 2.4 Hz, 1H), 6.81–6.77 (m, 2H), 4.91 (d, J = 16.4 Hz, 1H), 4.38 (dd, J = 9.2, 4.0 Hz, 1H), 4.15 (d, J = 16.4 Hz, 1H), 2.83–2.75 (m, 1H), 2.71–2.65 (m, 1H), 2.34–2.25 (m, 1H), 2.15–2.08 (m, 1H), 1.26 (s, 9H); ^{13}C NMR (100 MHz, $CDCl_3$) δ 153.05, 144.60, 142.81, 136.96, 129.36, 128.36, 128.09, 127.39, 127.18, 126.07, 126.00, 125.72, 125.17, 119.09, 116.03, 115.96, 61.26, 52.92, 34.00, 31.52, 29.10, 26.89; HRMS (ESI-TOF) *m/z* Calcd for $C_{26}H_{30}NO$ [M+H]$^+$: 372.2322, found: 372.2330.

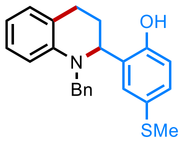

3r

2-(1-Benzyl-1,2,3,4-tetrahydroquinolin-2-yl)-4-(methylthio)phenol

3r was synthesized following the procedure. After purification by flash column chromatography using PE/EA (15/1) as the eluent, **3r** was obtained in 36% yield (26.1 mg) as a brown oil. ^{1}H NMR (400 MHz, $CDCl_3$) δ 7.99 (brs, 1H), 7.24–7.21 (m, 2H), 7.16–7.10 (m, 3H), 7.09–7.05 (m, 2H), 7.03–6.99 (m, 2H), 6.97 (d, *J* = 2.4 Hz, 1H), 6.80–6.77 (m, 2H), 4.88 (d, *J* = 16.4 Hz, 1H), 4.45 (dd, *J* = 8.8, 4.4 Hz, 1H), 4.16 (d, *J* = 16.4 Hz, 1H), 2.79–2.63 (m, 2H), 2.40 (s, 3H), 2.31–2.21 (m, 1H), 2.15–2.07 (m, 1H); ^{13}C NMR (100 MHz, $CDCl_3$) δ 153.94, 144.29, 136.71, 129.31, 129.12, 129.04, 128.47, 128.03, 127.92, 127.83, 127.44, 127.22, 125.72, 119.15, 117.34, 115.79, 60.46, 53.13, 28.76, 26.44, 18.03; HRMS (ESI-TOF) *m/z* Calcd for $C_{23}H_{22}NOS$ $[M+H]^+$: 362.1573, found: 362.1579.

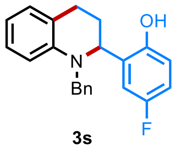

3s

2-(1-Benzyl-1,2,3,4-tetrahydroquinolin-2-yl)-4-fluorophenol

3s was synthesized following the procedure. After purification by flash column chromatography using PE/EA (20/1-15/1) as the eluent, **3s** was obtained in 44% yield (29.4 mg) as a brown oil. ^{1}H NMR (400 MHz, $CDCl_3$) δ 7.52 (brs, 1H), 7.26–7.23 (m, 3H), 7.15–7.07 (m, 3H), 7.03 (dd, *J* = 7.2, 2.0 Hz, 1H), 6.97–6.95 (m, 1H), 6.88–6.83 (m, 1H), 6.80–6.70 (m, 3H), 4.88 (d, *J* = 16.4 Hz, 1H), 4.52–4.47 (m, 1H), 4.18–4.13 (m, 1H), 2.78–2.63 (m, 2H), 2.31–2.20 (m, 1H), 2.17–2.08 (m, 1H); ^{13}C NMR (100 MHz, $CDCl_3$) δ 156.75 (d, *J* = 238 Hz), 150.92, 144.28, 136.80 (d, *J* = 3.6 Hz), 129.31, 128.55, 127.65, 127.51, 127.25, 125.20, 118.89, 117.21 (d, *J* = 7.8 Hz), 115.24 (d, *J* = 7.0 Hz), 114.88 (d, *J* = 21.2 Hz), 114.65 (d, *J* = 20.7 Hz), 59.83, 53.24, 28.24, 26.04; ^{19}F NMR (375 MHz, $CDCl_3$) δ -124.08 (td, *J* = 8.5, 4.5 Hz); HRMS (ESI-TOF) *m/z* Calcd for $C_{22}H_{21}NOF$ $[M+H]^+$: 334.1602, found: 334.1609.

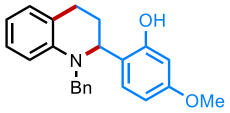

3t

2-(1-Benzyl-1,2,3,4-tetrahydroquinolin-2-yl)-5-methoxyphenol

3t was synthesized following the procedure. After purification by flash column chromatography using PE/EA (15/1) as the eluent, **3t** was obtained in 59% yield (40.6 mg) as a yellow solid. ^{1}H NMR (400 MHz, $CDCl_3$) δ 8.07 (brs, 1H), 7.24–7.21 (m, 3H), 7.14–7.10 (m, 1H), 7.08–7.05 (m, 2H), 7.03–7.00 (m, 2H), 6.88–6.85 (m, 1H), 6.78 (td, *J* = 7.2, 1.2 Hz, 1H), 6.43–6.40 (m, 2H), 4.88 (d, *J* = 16.4 Hz, 1H), 4.44–4.40 (m, 1H), 4.19 (d, *J* = 16.4 Hz, 1H), 3.77 (s, 3H), 2.80–2.64 (m, 2H), 2.32–2.21 (m, 1H), 2.13–2.05 (m, 1H); ^{13}C NMR (100 MHz, $CDCl_3$) δ 160.06, 156.42, 144.52, 136.90, 129.33, 129.27, 128.42, 127.91, 127.36, 127.14, 125.86, 119.26, 119.03, 115.93, 105.92, 102.21,

60.12, 55.25, 52.85, 29.03, 26.57; HRMS (ESI-TOF) *m/z* Calcd for $C_{23}H_{24}NO_2$ $[M+H]^+$: 346.1802, found: 346.1800.

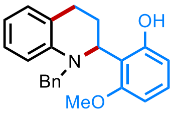

3u

2-(1-Benzyl-1,2,3,4-tetrahydroquinolin-2-yl)-3-methoxyphenol

3u was synthesized following the procedure. After purification by flash column chromatography using PE/EA (15/1) as the eluent, **3u** was obtained in 36% yield (25.2 mg) as a yellow oil. ^{1}H NMR (400 MHz, $CDCl_3$) δ 9.53 (brs, 1H), 7.20–7.19 (m, 3H), 7.16–7.11 (m, 3H), 7.02–7.00 (m, 3H), 6.84–6.80 (m, 1H), 6.53–6.51 (m, 1H), 6.43 (d, *J* = 8.4 Hz, 1H), 4.92–4.84 (m, 2H), 4.16 (d, *J* = 16.0 Hz, 1H), 3.73 (s, 3H), 2.80–2.61 (m, 2H), 2.20–2.07 (m, 2H); ^{13}C NMR (100 MHz, $CDCl_3$) δ 157.88, 157.60, 144.10, 136.21, 129.41, 128.71, 128.53, 128.15, 127.72, 127.20, 120.04, 117.84, 114.15, 109.95, 102.06, 55.48, 54.74, 53.15, 28.15, 27.69; HRMS (ESI-TOF) *m/z* Calcd for $C_{23}H_{24}NO_2$ $[M+H]^+$: 346.1802, found: 346.1798.

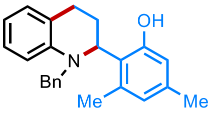

3v

2-(1-Benzyl-1,2,3,4-tetrahydroquinolin-2-yl)-3,5-dimethylphenol

3v was synthesized following the procedure. After purification by flash column chromatography using PE/EA (15/1) as the eluent, **3v** was obtained in 35% yield (23.7 mg) as a yellow oil. ^{1}H NMR (400 MHz, $CDCl_3$) δ 9.39 (brs, 1H), 7.22–7.15 (m, 5H), 7.04 (d, *J* = 7.2 Hz, 1H), 6.97–6.93 (m, 2H), 6.87–6.83 (m, 1H), 6.58 (d, *J* = 1.6 Hz, 1H), 6.50 (d, *J* = 1.6 Hz, 1H), 5.03 (d, *J* = 16.0 Hz, 1H), 4.41 (dd, *J* = 11.6, 3.6 Hz, 1H), 4.08 (d, *J* = 16.0 Hz, 1H), 2.86–2.78 (m, 1H), 2.73–2.70 (m, 1H), 2.27–2.17 (m, 4H), 2.03–1.97 (m, 1H), 1.93 (s, 3H); ^{13}C NMR (100 MHz, $CDCl_3$) δ 156.76, 144.50, 138.15, 136.62, 135.93, 129.66, 128.44, 128.38, 127.44, 127.37, 127.16, 123.12, 120.84, 120.15, 117.76, 115.63, 57.25, 52.63, 28.08, 27.83, 21.00, 19.11; HRMS (ESI-TOF) *m/z* Calcd for $C_{24}H_{26}NO$ $[M+H]^+$: 344.2009, found: 344.2008.

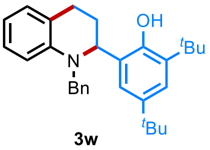

3w

2-(1-Benzyl-1,2,3,4-tetrahydroquinolin-2-yl)-4,6-di-tert-butylphenol

3w was synthesized following the procedure. After purification by flash column chromatography using PE/EA (50/1) as the eluent, **3w** was obtained in 54% yield (46.4 mg) as a yellow oil. ^{1}H NMR (400 MHz, $CDCl_3$) δ 8.76 (brs, 1H), 7.24–7.23 (m, 1H), 7.21–7.18 (m, 3H), 7.16–7.11 (m, 2H), 7.03–6.99 (m, 3H), 6.83–6.79 (m, 1H), 6.77–6.76 (m, 1H), 4.92 (d, *J* = 16.0 Hz, 1H), 4.24 (dd, *J* = 11.2, 3.6 Hz, 1H), 4.12 (d, *J* = 16.0 Hz, 1H), 2.87–2.79 (m, 1H), 2.72–2.66 (m, 1H), 2.43–2.33 (m, 1H), 2.11–2.04 (m, 1H), 1.42 (s, 9H), 1.29 (s, 9H); ^{13}C NMR (100 MHz,

CDCl$_3$) δ 152.67, 144.59, 141.25, 136.87, 136.14, 129.48, 128.51, 128.27, 127.32, 127.24, 126.88, 125.45, 123.76, 122.82, 119.85, 117.38, 63.25, 53.11, 34.96, 34.20, 31.67, 29.68, 29.25, 27.71; HRMS (ESI-TOF) *m/z* Calcd for $C_{30}H_{38}NO$ [M+H]$^+$: 428.2948, found: 428.2952.

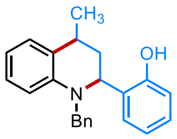

3x

1-Benzyl-4-methyl-1,2,3,4-tetrahydroquinolin-2-yl)phenol

3x was synthesized following the procedure. After purification by preparative thin-layer chromatography using PE/EA (20/1) as the eluent, **3x** was obtained in 36% total yield as a yellow oil with 1/1 dr. For one isomer (**3x'**), 18% yield:^{1}H NMR (400 MHz, CDCl$_3$) δ 8.78 (brs, 1H), 7.22–7.16 (m, 7H), 6.99–6.84 (m, 6H), 4.99 (d, *J* = 16.0 Hz, 1H), 4.38 (dd, *J* = 10.8, 5.2 Hz, 1H), 4.11 (d, *J* = 16.0 Hz, 1H), 2.86 (dd, *J* = 11.6, 6.0 Hz, 1H), 2.12–2.01 (m, 2H), 1.29 (d, *J* = 6.8 Hz, 3H); ^{13}C NMR (100 MHz, CDCl$_3$) δ 155.61, 143.75, 136.48, 130.75, 128.59, 128.46, 128.42, 128.36, 128.05, 127.30, 127.28, 127.07, 120.14, 119.07, 116.64, 115.84, 56.55, 52.85, 35.85, 29.63, 22.16; HRMS (ESI-TOF) *m/z* Calcd for $C_{23}H_{24}NO$ [M+H]$^+$: 330.1852, found: 330.1857. For another isomer (**3x''**), 18% yield : ^{1}H NMR (400 MHz, CDCl$_3$) δ 8.01 (brs, 1H), 7.24–7.13 (m, 5H), 7.10–7.04 (m, 4H), 6.98 (dd, *J* = 7.5, 1.6 Hz, 1H), 6.88–6.81 (m, 3H), 4.95 (d, *J* = 16.0 Hz, 1H), 4.50 (dd, *J* = 9.6, 4.4 Hz, 1H), 4.16 (d, *J* = 16.0 Hz, 1H), 2.90–2.82 (m, 1H), 2.36–2.29 (m, 1H), 1.85 (dt, *J* = 13.6, 4.8 Hz, 1H), 1.13 (d, *J* = 7.2 Hz, 3H); ^{13}C NMR (100 MHz, CDCl$_3$) δ 155.61, 143.75, 136.48, 130.76, 128.59, 128.46, 128.42, 128.36, 128.05, 127.31, 127.28, 127.07, 120.14, 119.07, 116.64, 115.84, 56.55, 52.85, 35.85, 29.63, 22.16; HRMS (ESI-TOF) *m/z* Calcd for $C_{23}H_{24}NO$ [M+H]$^+$: 330.1852, found: 330.1857.

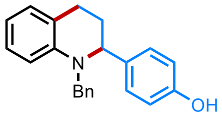

3y

4-(1-Benzyl-1,2,3,4-tetrahydroquinolin-2-yl)phenol

3y was synthesized following the procedure. After purification by flash column chromatography using PE/EA (15/1) as the eluent, **3y** was obtained in 88% yield (55.3 mg) as a yellow oil. ^{1}H NMR (400 MHz, CDCl$_3$) δ 7.32–7.27 (m, 2H), 7.21 (t, *J* = 7.9 Hz, 3H), 7.06–7.00 (m, 4H), 6.76–6.73 (m, 2H), 6.61 (q, *J* = 7.4, 5.4 Hz, 1H), 6.54 (d, *J* = 8.2 Hz, 1H), 4.78–4.74 (m, 1H), 4.70–4.61 (m, 2H), 4.22 (d, *J* = 17.5 Hz, 1H), 2.69–2.58 (m, 2H), 2.30–2.21 (m, 1H), 2.06–2.00 (m, 1H); ^{13}C NMR (100 MHz, CDCl$_3$) δ 154.54, 145.15, 138.57, 135.96, 128.77, 128.57, 127.89, 127.34, 126.65, 126.24, 122.05, 115.51, 115.21, 110.29, 60.60, 52.60, 29.52, 23.47; HRMS (ESI-TOF) *m/z* Calcd for $C_{22}H_{22}NO$ [M+H]$^+$: 316.1696, found: 316.1691.

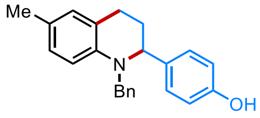

3z

4-(1-Benzyl-6-methyl-1,2,3,4-tetrahydroquinolin-2-yl)phenol

3z was synthesized following the procedure. After purification by preparative thin-layer using PE/EA (15/1) as the eluent, **3z** was obtained in 77% yield (50.9 mg) as a yellow foam. ^{1}H NMR (400 MHz, $CDCl_3$) δ 7.29–7.26 (m, 2H), 7.22–7.19 (m, 3H), 7.06–7.02 (m, 2H), 6.82 (d, *J* = 8.0 Hz, 2H), 6.75–6.72 (m, 2H), 6.45 (d, *J* = 8.0 Hz, 1H), 4.77 (brs, 1H), 4.63 (d, *J* = 17.6 Hz, 1H), 4.58 (t, *J* = 4.0 Hz, 1H), 4.20 (d, *J* = 17.6 Hz, 1H), 2.66–2.55 (m, 2H), 2.27–2.22 (m, 4H), 2.05–1.99 (m, 1H); ^{13}C NMR (100 MHz, $CDCl_3$) δ 154.54, 142.94, 138.85, 136.12, 129.53, 128.54, 127.90, 127.74, 126.58, 126.26, 124.50, 122.03, 115.18, 110.30, 60.60, 52.71, 29.73, 23.52, 20.17; HRMS (ESI-TOF) *m/z* Calcd for $C_{23}H_{24}NO$ $[M+H]^+$: 330.1852, found: 330.1855.

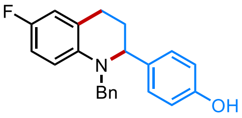

3aa

4-(1-Benzyl-6-fluoro-1,2,3,4-tetrahydroquinolin-2-yl)phenol

3aa was synthesized following the procedure. After purification by preparative thin-layer using PE/EA (15/1) as the eluent, **3aa** was obtained in 49% yield (32.6 mg) as a white foam. ^{1}H NMR (400 MHz, $CDCl_3$) δ 7.30 (t, *J* = 7.0 Hz, 2H), 7.24–7.18 (m, 3H), 7.04 (dd, *J* = 8.5, 4.5 Hz, 2H), 6.83–6.69 (m, 4H), 6.42–6.40 (m, 1H), 4.84–4.72 (m, 1H), 4.62–4.58 (m, 2H), 4.23 (dd, *J* = 17.4, 4.9 Hz, 1H), 2.68–2.57 (m, 2H), 2.29–2.21 (m, 1H), 2.09–2.01 (m, 1H); ^{13}C NMR (100 MHz, $CDCl_3$) δ 154.52 (*J* = 238 Hz), 154.48, 141.49, 138.51, 136.02, 128.63, 127.89, 126.75, 126.22, 123.41 (d, *J* = 6.5 Hz), 115.26 (d, *J* = 2.1 Hz), 115.25 (*J* = 22 Hz), 113.29 (d, *J* = 22 Hz), 110.79 (d, *J* = 7.4 Hz), 60.70, 53.16, 29.58, 23.82; ^{19}F NMR (375 MHz, $CDCl_3$) δ -130.69; HRMS (ESI-TOF) *m/z* Calcd for $C_{22}H_{21}NOF$ $[M+H]^+$: 334.1602, found: 334.1606.

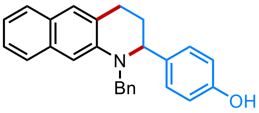

3ab

4-(1-Benzyl-1,2,3,4-tetrahydrobenzo[g]quinolin-2-yl)phenol

3ab was synthesized following the procedure. After purification by preparative thin-layer using PE/EA (15/1) as the eluent, **3ab** was obtained in 72% yield (52.9 mg) as a yellow foam. ^{1}H NMR (400 MHz, $CDCl_3$) δ 7.59 (d, *J* = 8.0 Hz, 1H), 7.46 (d, *J* = 8.0 Hz, 2H), 7.30–7.27 (m, 2H), 7.24–7.21 (m, 4H), 7.13–7.10 (m, 1H), 7.03 (d, *J* = 8.4 Hz, 2H), 6.79 (s, 1H), 6.71 (d, *J* = 8.2 Hz, 2H), 4.82 (d, *J* = 17.2 Hz, 2H), 4.67 (t, *J* = 4.2 Hz, 1H), 4.26 (d, *J* = 17.2 Hz, 1H), 2.85–2.76 (m, 2H), 2.36–2.27 (m, 1H), 2.08–2.03 (m, 1H); ^{13}C NMR (100 MHz, $CDCl_3$) δ 154.52, 143.99, 138.08, 135.69, 134.35, 128.66, 127.98, 126.87, 126.77, 126.72, 126.41, 126.42, 125.80, 125.56, 125.36, 121.60, 115.25, 103.62, 60.57, 52.92, 29.81, 23.91; HRMS (ESI-TOF) *m/z* Calcd for $C_{26}H_{24}NO$ $[M+H]^+$: 366.1852, found: 366.1853.

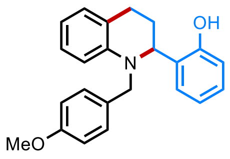

5a

2-(1-(4-Methoxybenzyl)-1,2,3,4-tetrahydroquinolin-2-yl)phenol

5a was synthesized following the procedure. After purification by flash column chromatography using PE/EA (10/1) as the eluent, **5a** was obtained in 74% yield (51.1 mg) as a yellow oil. ^{1}H NMR (400 MHz, $CDCl_3$) δ 8.11 (brs, 1H), 7.19–7.11 (m, 2H), 7.05–6.95 (m, 5H), 6.87–6.74 (m, 5H), 4.83 (d, *J* = 16.0 Hz, 1H), 4.47–4.44 (m, 1H), 4.09 (dd, *J* = 16.0, 2.8 Hz, 1H), 3.75 (s, 3H), 2.77–2.61 (m, 2H), 2.32–2.20 (m, 1H), 2.12–2.04 (m, 1H); ^{13}C NMR (100 MHz, $CDCl_3$) δ 158.72, 155.38, 144.43, 129.34, 129.17, 128.75, 128.58, 128.42, 127.34, 127.09, 125.90, 120.08, 118.99, 116.50, 116.01, 113.78, 60.20, 55.15, 52.50, 28.66, 26.53; HRMS (ESI-TOF) *m/z* Calcd for $C_{23}H_{24}NO_2$ $[M+H]^+$: 346.1802, found: 346.1810.

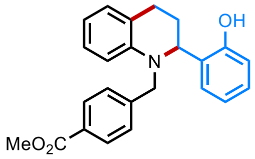

5b

Methyl-4-((2-(2-hydroxyphenyl)-3,4-dihydroquinolin-1(2H)-yl)methyl)benzoate

5b was synthesized following the procedure. After purification by flash column chromatography using PE/EA (10/1) as the eluent, **5b** was obtained in 74% yield (55.0 mg) as a yellow solid. ^{1}H NMR (400 MHz, $CDCl_3$) δ 7.92 (dd, *J* = 8.3, 2.1 Hz, 2H), 7.36 (brs, 1H), 7.20–7.12 (m, 3H), 7.09–7.05 (m, 1H), 7.03–6.98 (m, 2H), 6.85–6.80 (m, 2H), 6.77–6.71 (m, 2H), 4.82 (d, *J* = 17.2 Hz, 1H), 4.69–4.65 (m, 1H), 4.25 (dd, *J* = 17.2, 2.0 Hz, 1H), 3.88 (s, 3H), 2.75–2.63 (m, 2H), 2.34–2.25 (m, 1H), 2.20–2.12 (m, 1H); ^{13}C NMR (100 MHz, $CDCl_3$) δ 167.08, 154.44, 144.44, 143.23, 129.82, 129.23, 128.80, 128.38, 127.69, 127.40, 127.23, 124.41, 120.21, 118.05, 116.17, 113.55, 59.29, 52.89, 52.09, 27.95, 25.53; HRMS (ESI-TOF) *m/z* Calcd for $C_{24}H_{24}NO_3$ $[M+H]^+$: 374.1751, found: 374.1755.

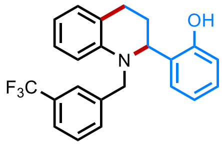

5c

2-(1-(3-(Trifluoromethyl)benzyl)-1,2,3,4-tetrahydroquinolin-2-yl)phenol

5c was synthesized following the procedure. After purification by flash column chromatography using PE/EA (25/1) as the eluent, **5c** was obtained in 64% yield (48.8 mg) as a yellow oil.^{1}H NMR (400 MHz, $CDCl_3$) δ 7.47 (d, *J* = 7.6 Hz, 1H), 7.36–7.21 (m, 4H), 7.17–7.08 (m, 2H), 7.04–6.98 (m, 2H), 6.86 (d, *J* = 8.0 Hz, 2H), 6.80–6.74 (m, 2H), 4.84 (d, *J* = 16.8 Hz, 1H), 4.56 (dd, *J* = 7.6, 4.4 Hz, 1H), 4.24 (dd, *J* = 16.8, 3.6 Hz, 1H), 2.77–2.64 (m, 2H), 2.34–2.25 (m, 1H), 2.19–2.11 (m, 1H); ^{13}C NMR (100 MHz, $CDCl_3$) δ 154.54, 144.30, 138.43, 130.73 (q, *J* = 32.2

Hz),130.68, 129.39, 128.98, 128.55, 128.45, 127.48, 127.30, 124.95, 124.64 (J = 272 Hz), 124.40 (q, J = 3.8 Hz), 123.97 (q, J = 3.8 Hz), 120.40, 118.62, 116.34, 114.25, 59.80, 52.88, 28.20, 25.85; ^{19}F NMR (375 MHz, $CDCl_3$) δ -62.63; HRMS (ESI-TOF) *m/z* Calcd for $C_{23}H_{21}NOF_3$ $[M+H]^+$: 384.1570, found: 384.1571.

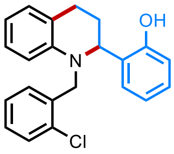

5d

2-(1-(2-Chlorobenzyl)-1,2,3,4-tetrahydroquinolin-2-yl)phenol

5d was synthesized following the procedure. After purification by flash column chromatography using PE/EA (15/1) as the eluent, **5d** was obtained in 75% yield (52.7 mg) as a yellow oil. ^{1}H NMR (400 MHz, $CDCl_3$) δ 7.34 (dt, J = 7.2, 1.2 Hz, 1H), 7.25–7.09 (m, 5H), 7.06–7.01 (m, 3H), 6.83 (t, J = 7.6 Hz, 1H), 6.74 (d, J = 8.0 Hz, 1H), 6.69 (t, J = 7.2 Hz, 1H), 6.54 (d, J = 8.4 Hz, 1H), 4.86–4.83 (m, 1H), 4.76 (d, J = 18.0 Hz, 1H), 4.30 (dd, J = 18.0, 1.6 Hz, 1H), 2.76–2.66 (m, 2H), 2.35–2.17 (m, 2H); ^{13}C NMR (100 MHz, $CDCl_3$) δ 153.66, 144.48, 134.71, 133.20, 129.65, 128.95, 128.43, 128.27, 128.21, 127.99, 127.69, 127.46, 126.80, 123.36, 120.44, 117.21, 115.94, 112.09, 58.32, 51.01, 27.42, 24.67; HRMS (ESI-TOF) *m/z* Calcd for $C_{22}H_{21}NOCl$ $[M+H]^+$: 350.1306, found: 350.1312.

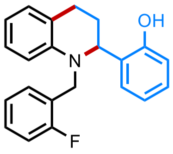

5e

2-(1-(2-Fluorobenzyl)-1,2,3,4-tetrahydroquinolin-2-yl)phenol

5e was synthesized following the procedure. After purification by flash column chromatography using PE/EA (15/1) as the eluent, **5e** was obtained in 63% yield (41.8 mg) as a yellow oil. ^{1}H NMR (400 MHz, $CDCl_3$) δ 7.22–6.98 (m, 9H), 6.86–6.72 (m, 4H), 4.76 (d, J = 17.2 Hz, 1H), 4.72–4.68 (m, 1H), 4.35 (d, J = 17.2 Hz, 1H), 2.78–2.65 (m, 2H), 2.34–2.26 (m, 1H), 2.23–2.14 (m, 1H); ^{13}C NMR (100 MHz, $CDCl_3$) δ 161.02 (d, J = 245.8 Hz), 154.46, 144.39, 129.09, 128.95 (d, J = 4.5 Hz), 128.58 (d, J = 8.2 Hz), 128.52, 128.36, 127.59, 127.39, 124.70, 124.18 (d, J = 14.7 Hz), 123.98 (d, J = 3.5 Hz), 120.27, 118.26, 116.22, 115.34 (d, J = 21.4 Hz), 114.11, 59.62, 47.26, 28.00, 25.54;; ^{19}F NMR (375 MHz, $CDCl_3$) δ -117.09; HRMS (ESI-TOF) *m/z* Calcd for $C_{22}H_{21}NOF$ $[M+H]^+$: 334.1602, found: 334.1611.

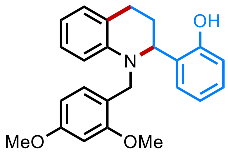

5f

2-(1-(2,4-Dimethoxybenzyl)-1,2,3,4-tetrahydroquinolin-2-yl)phenol

5f was synthesized following the procedure. After purification by flash column chromatography using PE/EA (15/1) as the eluent, **5f** was obtained in 68% yield (50.9 mg) as a yellow oil. ^{1}H NMR (400 MHz, $CDCl_3$) δ 7.94 (brs, 1H), 7.11–7.04 (m, 2H), 7.03–6.93 (m, 3H), 6.87 (d, *J* = 8.4 Hz, 1H), 6.82–6.78 (m, 1H), 6.74–6.70 (m, 2H), 6.44–6.41 (m, 1H), 6.36–6.32 (m, 1H), 4.69–4.64 (m, 1H), 4.57 (dd, *J* = 16.8, 2.8 Hz, 1H), 4.25 (dd, *J* = 16.8, 2.8 Hz, 1H), 3.75 (s, 3H), 3.70 (s, 3H), 2.74–2.67 (m, 2H), 2.33–2.24 (m, 1H), 2.22–2.13 (m, 1H); ^{13}C NMR (100 MHz, $CDCl_3$) δ 159.93, 158.56, 155.02, 144.93, 128.89, 128.84, 128.37, 128.04, 127.60, 127.19, 124.95, 119.79, 118.28, 117.24, 116.17, 115.36, 103.61, 98.39, 58.93, 55.26, 54.97, 49.22, 27.20, 25.32; HRMS (ESI-TOF) *m/z* Calcd for $C_{24}H_{25}NO_3Na$ [M+Na]$^+$: 398.1727, found: 398.1734.

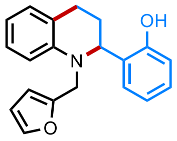

5g

2-(1-(Furan-2-ylmethyl)-1,2,3,4-tetrahydroquinolin-2-yl)phenol

5g was synthesized following the procedure. After purification by flash column chromatography using PE/EA (15/1) as the eluent, **5g** was obtained in 66% yield (40.5 mg) as a yellow oil. ^{1}H NMR (400 MHz, $CDCl_3$) δ 8.41 (brs, 1H), 7.28–7.26 (m, 1H), 7.20–7.06 (m, 4H), 7.01–6.99 (m, 1H), 6.89–6.79 (m, 3H), 6.22–6.19 (m, 1H), 5.98–5.96 (m, 1H), 4.70 (d, *J* = 16.8 Hz, 1H), 4.48–4.43 (m, 1H), 4.21 (dd, *J* = 16.8, 1.6 Hz, 1H), 2.86–2.78 (m, 1H), 2.71–2.65 (m, 1H), 2.36–2.26 (m, 1H), 2.12–2.03 (m, 1H); ^{13}C NMR (100 MHz, $CDCl_3$) δ 155.71, 150.77, 143.91, 142.02, 129.33, 128.71, 128.64, 127.10, 126.70, 126.32, 120.09, 119.91, 116.80, 116.73, 110.05, 108.73, 62.17, 46.45, 28.97, 26.93; HRMS (ESI-TOF) *m/z* Calcd for $C_{20}H_{20}NO_2$ [M+H]$^+$: 306.1589, found: 306.1496.

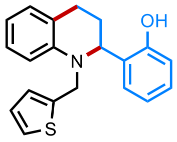

5h

2-(1-(Thiophen-2-ylmethyl)-1,2,3,4-tetrahydroquinolin-2-yl)phenol

5h was synthesized following the procedure. After purification by flash column chromatography using PE/EA (15/1) as the eluent, **5h** was obtained in 74% yield (47.6 mg) as a yellow oil. ^{1}H NMR (400 MHz, $CDCl_3$) δ 8.58 (brs, 1H), 7.22–7.16 (m, 3H), 7.10 (dd, *J* = 5.2, 1.2 Hz, 1H), 7.05–7.01 (m, 2H), 6.89–6.83 (m, 4H), 6.74–6.72 (m, 1H), 4.99 (d, *J* = 16.8 Hz, 1H), 4.38–4.34 (m, 2H), 2.80–2.72 (m, 1H), 2.67–2.61 (m, 1H), 2.35–2.25 (m, 1H), 2.09–2.02 (m, 1H); ^{13}C NMR (100 MHz, $CDCl_3$) δ 155.81, 143.40, 137.76, 129.60, 128.71, 128.68, 127.34, 127.25, 127.18, 126.46, 126.19, 125.24, 120.23, 120.11, 117.09, 116.77, 61.09, 47.94, 28.97, 27.08; HRMS (ESI-TOF) *m/z* Calcd for $C_{20}H_{20}NOS$ [M+H]$^+$: 322.1260, found: 322.1269.

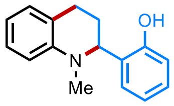

5i

2-(1-Methyl-1,2,3,4-tetrahydroquinolin-2-yl)phenol

5i was synthesized following the procedure, stirred at 80 °C for 24 h. After purification by flash column chromatography using PE/EA (15/1) as the eluent, **5i** was obtained in 74% yield (35.6 mg) as a yellow oil. ^{1}H NMR (400 MHz, $CDCl_3$) δ 8.97 (brs, 1H), 7.18 (t, J = 8.4 Hz, 2H), 7.06–7.04 (m, 2H), 6.99 (d, J = 8.3 Hz, 1H), 6.87–6.84 (m, 3H), 4.11 (dd, J = 10.4, 3.6 Hz, 1H), 3.01–2.94 (m, 1H), 2.90 (s, 3H), 2.80–2.71 (m, 1H), 2.38–2.28 (m, 1H), 2.13–2.08 (m, 1H); ^{13}C NMR (100 MHz, $CDCl_3$) δ 155.76, 146.00, 129.13, 128.56, 128.35, 127.25, 127.00, 125.83, 120.14, 119.90, 116.77, 116.38, 65.72, 39.32, 29.66, 27.15; HRMS (ESI-TOF) *m/z* Calcd for $C_{16}H_{18}NO$ $[M+H]^+$: 240.1383, found: 240.1385.

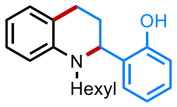

5j

2-(1-Hexyl-1,2,3,4-tetrahydroquinolin-2-yl)phenol

5j was synthesized following the procedure. After purification by flash column chromatography using PE/EA (15/1) as the eluent, **5j** was obtained in 56% yield (34.5 mg) as a yellow oil. ^{1}H NMR (400 MHz, $CDCl_3$) δ 8.63 (brs, 1H), 7.19–7.12 (m, 2H), 7.06–7.00 (m, 2H), 6.94 (d, J = 8.4 Hz, 1H), 6.86–6.77 (m, 3H), 4.36 (dd, J = 8.4, 5.2 Hz, 1H), 3.56–3.48 (m, 1H), 3.08–3.00 (m, 1H), 2.93–2.85 (m, 1H), 2.70 (dt, J = 16.0, 4.4 Hz, 1H), 2.35–2.20 (m, 1H), 2.12–2.05 (m, 1H), 1.51–1.47 (m, 1H), 1.26–1.16 (m, 7H), 0.84–0.80 (m, 3H); ^{13}C NMR (100 MHz, $CDCl_3$) δ 155.78, 144.15, 129.34, 128.50, 128.33, 127.24, 127.20, 126.06, 119.91, 119.32, 116.67, 116.17, 62.18, 49.73, 31.42, 29.29, 27.13, 26.75, 23.96, 22.49, 13.93; HRMS (ESI-TOF) *m/z* Calcd for $C_{21}H_{28}NO$ $[M+H]^+$: 310.2165, found: 310.2173.

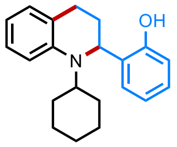

5k

2-(1-Cyclohexyl-1,2,3,4-tetrahydroquinolin-2-yl)phenol

5k was synthesized following the procedure. After purification by flash column chromatography using PE/EA (15/1) as the eluent, **5k** was obtained in 63% yield (39.0 mg) as a yellow oil. ^{1}H NMR (400 MHz, $CDCl_3$) δ 9.26 (brs, 1H), 7.17–6.99 (m, 5H), 6.82–6.74 (m, 2H), 6.75 (d, J = 8.0 Hz, 1H), 4.62 (dd, J = 7.2, 5.2 Hz, 1H), 3.52 (tt, J = 11.6, 3.6 Hz, 1H), 2.73–2.58 (m, 2H), 2.17–1.98 (m, 2H), 1.84–1.69 (m, 4H), 1.61–1.54 (m, 2H), 1.35–1.16 (m, 3H), 1.10–0.99 (m, 1H); ^{13}C NMR (100 MHz, $CDCl_3$) δ 155.84, 144.19, 128.97, 128.95, 127.84, 127.80, 127.20, 119.87, 119.63, 118.51, 116.81, 63.24, 58.26, 31.39, 30.63, 28.85, 26.51, 26.37, 26.18, 25.76; HRMS (ESI-TOF) *m/z* Calcd for $C_{21}H_{26}NO$ $[M+H]^+$: 308.2009, found: 308.2015.

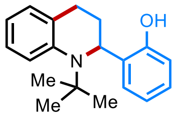

5l

2-(1-(*Tert*-butyl)-1,2,3,4-tetrahydroquinolin-2-yl)phenol

5l was synthesized following the procedure. After purification by flash column chromatography using PE/EA (15/1) as the eluent, **5l** was obtained in 40% yield (22.9 mg) as a yellow oil. ^{1}H NMR (400 MHz, $CDCl_3$) δ 12.09 (brs, 1H), 7.18–7.10 (m, 2H), 7.07–6.98 (m, 4H), 6.76–6.72 (m, 2H), 4.72 (dd, *J* = 10.4, 5.6 Hz, 1H), 2.81–2.73 (m, 1H), 2.77–2.61 (m, 1H), 2.43–2.36 (m, 1H), 1.87–1.77 (m, 1H), 1.24 (s, 9H); ^{13}C NMR (100 MHz, $CDCl_3$) δ 157.13, 140.86, 137.74, 129.00, 128.27, 127.89, 127.62, 126.77, 125.84, 124.69, 118.88, 117.11, 60.59, 60.14, 34.02, 28.63, 27.83; HRMS (ESI-TOF) *m/z* Calcd for $C_{19}H_{24}NO$ [M+H]$^+$: 282.1852, found: 282.1863.

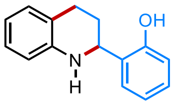

5m

2-(1,2,3,4-Tetrahydroquinolin-2-yl)phenol

5m was synthesized following the procedure. After purification by preparative thin-layer chromatography using PE/EA (10/1) as the eluent, **5m** was obtained in 59% yield (26.5 mg) as a yellow oil. ^{1}H NMR (400 MHz, $CDCl_3$) δ 9.04 (brs, 1H), 7.20 (td, *J* = 7.6, 1.6 Hz, 1H), 7.07 (t, *J* = 7.2 Hz, 3H), 6.90–6.82 (m, 3H), 6.70–6.68 (m, 1H), 4.37 (dd, *J* = 11.6, 2.8 Hz, 1H), 4.27 (brs, 1H), 3.07–2.98 (m, 1H), 2.88–2.82 (m, 1H), 2.40–2.30 (m, 1H), 2.11–2.05 (m, 1H); ^{13}C NMR (100 MHz, $CDCl_3$) δ 156.32, 142.57, 129.72, 129.04, 128.20, 126.83, 126.71, 123.50, 120.76, 119.81, 117.31, 117.27, 57.86, 28.60, 26.67; HRMS (ESI-TOF) *m/z* Calcd for $C_{15}H_{16}NO$ [M+H]$^+$: 226.1226, found: 226.1230.

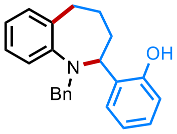

7a

2-(1-Benzyl-2,3,4,5-tetrahydro-1*H*-benzo[*b*]azepin-2-yl)phenol

7a was synthesized following the procedure. After purification by flash column chromatography using PE/EA (50/1) as the eluent, **7a** was obtained in 56% yield (37.2 mg) as a yellow oil. ^{1}H NMR (400 MHz, acetone-*d*6) δ 8.93 (brs, 1H), 7.59 (dd, *J* = 7.2, 1.6 Hz, 1H), 7.20–7.02 (m, 8H), 6.98–6.96 (m, 1H), 6.90–6.85 (m, 3H), 4.65 (dd, *J* = 6.8, 3.6 Hz, 1H), 4.25 (q, *J* = 15.2 Hz, 2H), 3.12–3.05 (m, 1H), 2.81–2.74 (m, 1H), 1.96–1.87 (m, 1H), 1.77–1.69 (m, 1H), 1.65–1.58 (m, 2H); ^{13}C NMR (100 MHz, acetone-*d*6) δ 156.18, 147.43, 139.80, 136.81, 130.11, 129.71, 128.87, 128.85, 128.71, 128.44, 127.53, 127.32, 123.46, 122.71, 120.25, 116.46, 60.96, 55.69, 31.54, 30.56, 21.99; HRMS (ESI-TOF) *m/z* Calcd for $C_{23}H_{24}NO$ [M+H]$^+$: 330.1852, found: 330.1859.

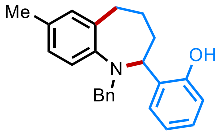

7b

2-(1-Benzyl-7-methyl-2,3,4,5-tetrahydro-1*H*-benzo[*b*]azepin-2-yl)phenol

7b was synthesized following the procedure. After purification by flash column chromatography using PE/EA (100/1) as the eluent, **7b** was obtained in 54% yield (37.4 mg) as a yellow oil. ^{1}H NMR (400 MHz, acetone-d_6) δ 9.17 (brs, 1H), 7.55 (dd, *J* = 7.6, 1.6 Hz, 1H), 7.17 (d, *J* = 4.4 Hz, 4H), 7.11 (td, *J* = 7.2, 6.3, 2.9 Hz, 2H), 6.89–6.86 (m, 3H), 6.84 (d, *J* = 1.2 Hz, 2H), 4.58 (dd, *J* = 6.8, 3.6 Hz, 1H), 4.20 (q, *J* = 14.8 Hz, 2H), 3.08–3.01 (m, 1H), 2.76–2.69 (m, 1H), 2.19 (s, 3H), 1.95–1.85 (m, 1H), 1.78–1.54 (m, 3H); ^{13}C NMR (100 MHz, acetone-d_6) δ 156.45, 144.54, 139.78, 137.02, 132.92, 130.61, 129.93, 128.99, 128.85, 128.70, 128.47, 127.96, 127.33, 123.05, 120.20, 116.51, 61.50, 55.66, 31.58, 30.63, 22.20, 20.73; HRMS (ESI-TOF) *m/z* Calcd for $C_{24}H_{26}NO$ $[M+H]^+$: 344.2009, found: 344.2010.

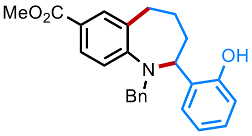

7c

Methyl-1-benzyl-2-(2-hydroxyphenyl)-2,3,4,5-tetrahydro-1*H*-benzo[*b*]azepine-7-carboxylate

7c was synthesized following the procedure. After purification by flash column chromatography using PE/EA (50/1) as the eluent, **7c** was obtained in 38% yield (29.6 mg) as a yellow oil. ^{1}H NMR (400 MHz, acetone-d_6) δ 8.59 (brs, 1H), 7.71–7.69 (m, 2H), 7.63 (dd, *J* = 7.6, 1.7 Hz, 1H), 7.27–7.19 (m, 4H), 7.15–7.09 (m, 2H), 7.03–6.98 (m, 1H), 6.93–6.88 (m, 2H), 4.85 (dd, *J* = 6.8, 3.2 Hz, 1H), 4.35 (q, *J* = 16.0 Hz, 2H), 3.80 (s, 3H), 3.18–3.11 (m, 1H), 2.86–2.79 (m, 1H), 2.12–2.07 (m, 1H), 1.84–1.76 (m, 1H), 1.70–1.54 (m, 2H); ^{13}C NMR (100 MHz, acetone-d_6) δ 156.47, 144.56, 139.78, 137.05, 132.95, 130.62, 129.94, 129.00, 128.85, 128.71, 128.48, 127.97, 127.34, 123.10, 120.21, 116.52, 61.54, 55.68, 31.60, 30.66, 22.24, 20.74; HRMS (ESI-TOF) *m/z* Calcd for $C_{25}H_{26}NO_3$ $[M+H]^+$: 388.1907, found: 388.1907.

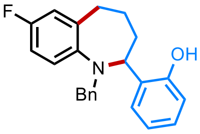

7d

2-(1-Benzyl-7-fluoro-2,3,4,5-tetrahydro-1*H*-benzo[*b*]azepin-2-yl)phenol

7d was synthesized following the procedure. After purification by flash column chromatography using PE/EA (50/1) as the eluent, **7d** was obtained in 58% yield (40.2 mg) as a yellow oil. ^{1}H NMR (400 MHz, acetone-d_6) δ 8.89 (brs, 1H), 7.59 (dd, *J* = 7.6, 1.6 Hz, 1H), 7.22–7.16 (m, 4H), 7.13–7.09 (m, 2H), 6.97–6.85 (m, 4H), 6.78 (td, *J* = 8.4, 3.2 Hz, 1H), 4.63 (dd, *J* = 6.8, 3.2 Hz, 1H), 4.25–4.16 (m, 2H), 3.14–3.07 (m, 1H), 2.84–2.77 (m, 1H), 1.92–1.84 (m, 1H), 1.75–1.60 (m, 3H); ^{13}C NMR (100 MHz, acetone-d_6) δ 159.48 (d, *J* = 240 Hz), 156.12, 143.47 (d, *J* = 2.6 Hz), 139.70, 139.50 (d, *J* = 7.6 Hz), 129.93, 129.09, 128.91, 128.54 (d, *J* = 9.0 Hz), 128.18, 127.39, 124.04, 120.27, 116.46, 116.17 (d, *J* = 21.9 Hz), 113.23 (d, *J* = 21.8 Hz), 60.72, 55.72, 31.44, 30.46, 21.64; ^{19}F NMR (375 MHz, acetone-d_6) δ -120.04; HRMS (ESI-TOF) *m/z* Calcd for $C_{23}H_{23}NOF$ $[M+H]^+$: 348.1759, found: 348.1763.

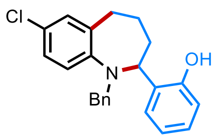

7e

2-(1-Benzyl-7-chloro-2,3,4,5-tetrahydro-1*H*-benzo[*b*]azepin-2-yl)phenol

7e was synthesized following the procedure. After purification by flash column chromatography using PE/EA (50/1) as the eluent, **7e** was obtained in 59% yield (43.0 mg) as a yellow solid. ^{1}H NMR (400 MHz, acetone-d_6) δ 8.70 (brs, 1H), 7.63 (dd, *J* = 7.6, 1.6 Hz, 1H), 7.24–7.17 (m, 4H), 7.13–7.10 (m, 3H), 7.04 (dd, *J* = 8.4, 2.4 Hz, 1H), 6.96–6.86 (m, 3H), 4.70 (dd, *J* = 6.8, 3.6 Hz, 1H), 4.28–4.19 (m, 2H), 3.14–3.07 (m, 1H), 2.84–2.76 (m, 1H), 1.97–1.88 (m, 1H), 1.75–1.67 (m, 1H), 1.64–1.57 (m, 2H); ^{13}C NMR (100 MHz, acetone-d_6) δ 155.81, 146.60, 139.66, 138.72, 130.01, 129.24, 128.97, 128.70, 128.59, 128.48, 127.40, 127.32, 127.10, 123.61, 120.33, 116.40, 60.37, 55.62, 31.25, 30.27, 21.39; HRMS (ESI-TOF) *m/z* Calcd for $C_{23}H_{23}NOCl$ $[M+H]^+$: 364.1463, found: 364.1462.

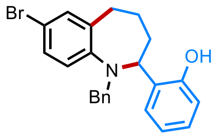

7f

2-(1-Benzyl-7-bromo-2,3,4,5-tetrahydro-1*H*-benzo[*b*]azepin-2-yl)phenol

7f was synthesized following the procedure, stirred at 80 °C for 36 h. After purification by flash column chromatography using PE/EA (50/1) as the eluent, **7f** was obtained in 56% yield (45.6 mg) as a yellow oil. ^{1}H NMR (400 MHz, acetone-d_6) δ 8.66 (brs, 1H), 7.63 (dd, *J* = 7.6, 1.6 Hz, 1H), 7.25–7.16 (m, 6H), 7.14–7.09 (m, 2H), 6.94–6.85 (m, 3H), 4.71 (dd, *J* = 6.8, 3.6 Hz, 1H), 4.28–4.20 (m, 2H), 3.15–3.07 (m, 1H), 2.84–2.76 (m, 1H), 1.98–1.90 (m,,1H), 1.76–1.68 (m, 1H), 1.65–1.56 (m, 2H); ^{13}C NMR (100 MHz, acetone-d_6) δ 155.79, 147.12, 139.65, 139.05, 132.07, 130.11, 130.01, 128.98, 128.66, 128.57, 128.47, 127.39, 123.95, 120.32, 116.39, 114.96, 60.27, 55.54, 31.17, 30.17, 21.31; HRMS (ESI-TOF) *m/z* Calcd for $C_{23}H_{23}NOBr$ $[M+H]^+$: 408.0958, found: 408.0962.

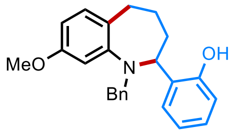

7g

2-(1-Benzyl-8-methoxy-2,3,4,5-tetrahydro-1*H*-benzo[*b*]azepin-2-yl)phenol

7g was synthesized following the procedure. After purification by flash column chromatography using PE/EA (50/1) as the eluent, **7g** was obtained in 53% yield (37.9 mg) as a yellow oil. ^{1}H NMR (400 MHz, acetone-d_6) δ 8.87 (brs, 1H), 7.63 (d, *J* = 7.2 Hz, 1H), 7.26–7.18 (m, 4H), 7.14–7.09 (m, 2H), 6.96 (dd, *J* = 8.0, 1.6 Hz, 1H), 6.90–6.85 (m, 2H), 6.55 (d, *J* = 2.4 Hz, 1H), 6.45 (dt, *J* = 8.0, 2.4 Hz, 1H), 4.65 (dd, *J* = 6.4, 3.6 Hz, 1H), 4.24 (q, *J* = 15.2 Hz, 2H), 3.63 (s, 3H), 3.07–3.00 (m, 1H), 2.74–2.68 (m, 1H), 1.98–1.89 (m, 1H), 1.79–1.70 (m, 1H), 1.63–1.53 (m, 2H); ^{13}C NMR (100 MHz, acetone-d_6) δ 159.84, 156.12, 148.43, 139.91, 130.09, 129.95, 129.11,

128.93, 128.79, 128.75, 128.44, 127.34, 120.26, 116.43, 109.47, 107.69, 61.11, 55.72, 55.22, 30.66, 30.49, 22.12; HRMS (ESI-TOF) *m/z* Calcd for $C_{24}H_{26}NO_2$ $[M+H]^+$: 360.1958, found: 360.1962.

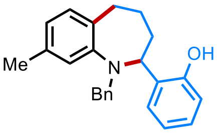

7h

2-(1-Benzyl-8-methyl-2,3,4,5-tetrahydro-1*H*-benzo[*b*]azepin-2-yl)phenol

7h was synthesized following the procedure. After purification by flash column chromatography using PE/EA (50/1) as the eluent, **7h** was obtained in 54% yield (37.4 mg) as a yellow oil. ^{1}H NMR (400 MHz, acetone-d_6) δ 9.05 (brs, 1H), 7.56 (dd, *J* = 7.6, 1.6 Hz, 1H), 7.20–7.16 (m, 4H), 7.14–7.09 (m, 2H), 6.94 (d, *J* = 7.6 Hz, 1H), 6.89–6.82 (m, 3H), 6.71 (dd, *J* = 7.2, 1.6 Hz, 1H), 4.62 (dd, *J* = 7.0, 3.6 Hz, 1H), 4.29–4.16 (m, 2H), 3.03–2.98 (m, 1H), 2.74–2.68 (m, 1H), 2.16 (s, 3H), 1.96–1.88 (m, 1H), 1.78–1.70 (m, 1H), 1.64–1.60 (m, 2H); ^{13}C NMR (100 MHz, acetone-d_6) δ 156.35, 147.27, 139.74, 136.80, 133.85, 130.13, 129.64, 129.02, 128.86, 128.75, 128.46, 127.38, 124.28, 123.95, 120.24, 116.51, 61.18, 55.82, 31.15, 30.70, 22.45, 21.28; HRMS (ESI-TOF) *m/z* Calcd for $C_{24}H_{26}NO$ $[M+H]^+$: 344.2009, found: 344.2013.

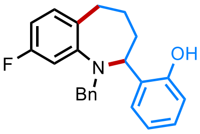

7i

2-(1-Benzyl-8-fluoro-2,3,4,5-tetrahydro-1*H*-benzo[*b*]azepin-2-yl)phenol

7i was synthesized following the procedure. After purification by flash column chromatography using PE/EA (50/1) as the eluent, **7i** was obtained in 52% yield (35.8 mg) as a yellow oil. ^{1}H NMR (400 MHz, acetone-d_6) δ 8.88 (brs, 1H), 7.59 (dd, *J* = 7.6, 1.6 Hz, 1H), 7.22–7.16 (m, 4H), 7.13–7.09 (m, 2H), 6.95 (dd, *J* = 8.8, 5.2 Hz, 1H), 6.91–6.85 (m, 3H), 6.78 (td, *J* = 8.8, 3.2 Hz, 1H), 4.63 (dd, *J* = 6.8, 3.2 Hz, 1H), 4.26–4.16 (m, 2H), 3.14–3.07 (m, 1H), 2.84–2.77 (m, 1H), 1.93–1.84 (m, 1H), 1.75–1.60 (m, 3H); ^{13}C NMR (100 MHz, acetone-d_6) δ 159.49 (d, *J* = 240 Hz), 156.13, 143.48 (d, *J* = 2.5 Hz), 139.71, 139.48 (d, *J* = 7.0 Hz), 129.94, 129.10, 128.92, 128.90, 128.55 (d, *J* = 9.4 Hz), 128.19, 127.40, 124.09, 116.47, 116.17 (d, *J* = 22.1 Hz), 113.24 (d, *J* = 21.8 Hz), 60.73, 55.74, 31.45, 30.47, 21.66; ^{19}F NMR (375 MHz, acetone-*d*6) δ -120.05; HRMS (ESI-TOF) *m/z* Calcd for $C_{23}H_{23}NOF$ $[M+H]^+$: 348.1758, found: 348.1761.

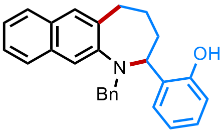

7j

2-(1-Benzyl-2,3,4,5-tetrahydro-1H-naphtho[2,3-*b*]azepin-2-yl)phenol

7j was synthesized following the procedure. After purification by flash column chromatography using PE/EA (50/1) as the eluent, **7j** was obtained in 47% yield (35.8 mg) as a yellow oil. ^{1}H NMR (400 MHz, acetone-d_6) δ 8.70 (brs, 1H), 7.76 (d, *J* = 7.6 Hz, 1H), 7.69 (d, *J* = 6.8,, 1H), 7.63–7.61 (m, 1H), 7.58 (s, 1H), 7.39 (s, 1H), 7.33–7.24 (m, 4H),

7.18–7.11 (m, 3H), 7.07–7.04 (m, 1H), 6.93–6.88 (m, 2H), 4.75 (dd, J = 6.4, 3.2 Hz, 1H), 4.44–4.36 (m, 2H), 3.38–3.31 (m, 1H), 3.01–2.95 (m, 1H), 1.99–1.92 (m, 1H), 1.79–1.66 (m, 3H); ^{13}C NMR (100 MHz, acetone-d_6) δ 155.87, 146.55, 140.03, 137.67, 134.37, 131.15, 129.99, 128.89, 128.70, 128.64, 128.44, 127.56, 127.47, 127.42, 127.21, 125.78, 124.72, 120.31, 118.68, 116.42, 61.62, 55.88, 31.63, 30.59, 22.10; HRMS (ESI-TOF) *m/z* Calcd for $C_{27}H_{26}NO$ $[M+H]^+$: 380.2009, found: 380.2002.

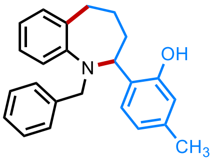

7k

2-(1-Benzyl-2,3,4,5-tetrahydro-1*H*-benzo[*b*]azepin-2-yl)-5-methylphenol

7k was synthesized following the procedure. After purification by flash column chromatography using PE/EA (50/1) as the eluent, **7k** was obtained in 49% yield (33.6 mg) as a yellow oil. ^{1}H NMR (400 MHz, acetone-d_6) δ 8.83 (brs, 1H), 7.43 (d, J = 7.6 Hz, 1H), 7.21–7.16 (m, 4H), 7.13–7.02 (m, 3H), 6.95 (dd, J = 8.0, 1.2 Hz, 1H), 6.88 (td, J = 7.2, 1.2 Hz, 1H), 6.73–6.68 (m, 2H), 4.60 (dd, J = 6.8, 3.2 Hz, 1H), 4.24 (q, J = 15.2 Hz, 2H), 3.11–3.04 (m, 1H), 2.81–2.74 (m, 1H), 2.25 (s, 3H), 1.93–1.86 (m, 1H), 1.76–1.57 (m, 3H); ^{13}C NMR (100 MHz, acetone-d_6) δ 156.12, 147.46, 139.87, 138.12, 136.93, 129.75, 128.89, 128.64, 127.53, 127.33, 126.99, 123.51, 122.80, 121.05, 121.07, 117.15, 61.02, 55.61, 31.63, 30.70, 22.05, 21.13; HRMS (ESI-TOF) *m/z* Calcd for $C_{24}H_{26}NO$ $[M+H]^+$: 344.2009, found: 344.2015.

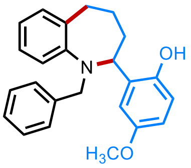

7l

2-(1-Benzyl-2,3,4,5-tetrahydro-1*H*-benzo[*b*]azepin-2-yl)-4-methoxyphenol

7l was synthesized following the procedure. After purification by flash column chromatography using PE/EA (50/1) as the eluent, **7l** was obtained in 41% yield (29.8 mg) as a yellow oil. ^{1}H NMR (400 MHz, acetone-d_6) δ 8.33 (brs, 1H), 7.24–7.17 (m, 5H), 7.13–7.03 (m, 3H), 6.99 (dd, J = 8.0, 1.2 Hz, 1H), 6.89–6.85 (m, 1H), 6.82 (d, J = 8.8 Hz, 1H), 6.68 (dd, J = 8.8, 3.2 Hz, 1H), 4.64 (dd, J = 6.8, 3.6 Hz, 1H), 4.31–4.22 (m, 2H), 3.68 (s, 3H), 3.13–3.06 (m, 1H), 2.83–2.77 (m, 1H), 1.96–1.84 (m, 1H), 1.75–1.57 (m, 3H); ^{13}C NMR (100 MHz, acetone-d_6) δ 153.95, 149.72, 147.48, 140.00, 136.66, 131.12, 129.67, 128.90, 128.74, 127.53, 127.30, 123.31, 122.37, 117.01, 113.95, 113.56, 60.80, 55.68, 31.56, 30.46, 21.79; HRMS (ESI-TOF) *m/z* Calcd for $C_{24}H_{26}NO_2$ $[M+H]^+$: 360.1958, found: 360.1956.

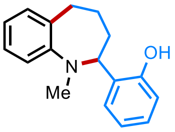

7m

2-(1-Methyl-2,3,4,5-tetrahydro-1*H*-benzo[*b*]azepin-2-yl)phenol

7m was synthesized following the procedure, stirred at 80 °C for 36 h. After purification by flash column chromatography using PE/EA (50/1) as the eluent, **7m** was obtained in 52% yield (26.4 mg) as a yellow oil. ^{1}H NMR (400 MHz, acetone-d_6) δ 9.47 (brs, 1H), 7.48 (d, *J* = 7.6 Hz, 1H), 7.23 (td, *J* = 7.6, 1.6 Hz, 1H), 7.14–7.06 (m, 3H), 6.96 (t, *J* = 7.2 Hz, 1H), 6.89–6.84 (m, 2H), 4.26 (t, *J* = 4.4 Hz, 1H), 3.20–3.12 (m, 1H), 2.73 (s, 3H), 2.68–2.62 (m, 1H), 1.96–1.88 (m, 1H), 1.73–1.64 (m, 1H), 1.57–1.42 (m, 2H); ^{13}C NMR (100 MHz, acetone-d_6) δ 156.80, 149.74, 135.38, 129.67, 129.09, 128.44, 128.06, 123.36, 120.59, 120.11, 119.74, 116.54, 64.76, 40.12, 31.14, 30.55, 20.31; HRMS (ESI-TOF) *m/z* Calcd for $C_{17}H_{20}NO$ [M+H]$^+$: 254.1539, found: 254.1547.

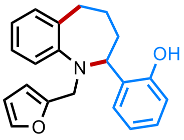

7n

2-(1-(Furan-2-ylmethyl)-2,3,4,5-tetrahydro-1*H*-benzo[*b*]azepin-2-yl)phenol

7n was synthesized following the procedure, stirred at 80 °C for 36 h. After purification by flash column chromatography using PE/EA (50/1) as the eluent, **7n** was obtained in 56% yield (35.6 mg) as a yellow oil. ^{1}H NMR (400 MHz, acetone-d_6) δ 9.01 (brs, 1H), 7.54 (dd, *J* = 7.6, 1.6 Hz, 1H), 7.37 (dd, *J* = 2.0, 0.8 Hz, 1H), 7.15–7.06 (m, 4H), 6.97–6.91 (m, 1H), 6.89–6.88 (m, 2H), 6.24 (dd, *J* = 3.2, 2.0 Hz, 1H), 6.04 (d, *J* = 3.2 Hz, 1H), 4.65 (dd, *J* = 7.2, 3.2 Hz, 1H), 4.22 (s, 2H), 2.95–2.90 (m, 1H), 2.74–2.67 (m, 1H), 1.96–1.88 (m, 1H), 1.76–1.68 (m, 1H), 1.66–1.60 (m, 2H); ^{13}C NMR (100 MHz, acetone-d_6) δ 156.39, 153.31, 147.57, 142.49, 136.85, 129.81, 129.72, 128.78, 128.50, 127.67, 123.85, 122.99, 120.25, 116.53, 110.95, 108.94, 60.81, 48.78, 31.24, 30.48, 22.49; HRMS (ESI-TOF) *m/z* Calcd for $C_{21}H_{20}NO_2$ [M+H]$^+$: 318.1489, found: 318.1483.

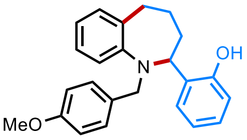

7o

2-(1-(4-Methoxybenzyl)-2,3,4,5-tetrahydro-1*H*-benzo[*b*]azepin-2-yl)phenol

7o was synthesized following the procedure. After purification by flash column chromatography using PE/EA (50/1) as the eluent, **7o** was obtained in 47% yield (34.0 mg) as a yellow oil. ^{1}H NMR (400 MHz, acetone-d_6) δ 9.04 (brs, 1H), 7.56–7.53 (m, 1H), 7.13–7.04 (m, 5H), 7.00–6.97 (m, 1H), 6.91–6.85 (m, 3H), 6.77–6.73 (m, 2H), 4.62 (dd, *J* = 7.2, 3.2 Hz, 1H), 4.23–4.11 (m, 2H), 3.70 (s, 3H), 3.07–3.00 (m, 1H), 2.80–2.73 (m, 1H), 1.95–1.86 (m, 1H), 1.77–1.69 (m, 1H), 1.67–1.60 (m, 2H); ^{13}C NMR (100 MHz, acetone-d_6) δ 159.43, 156.32, 147.47, 137.03, 131.49, 130.10, 129.76, 128.73, 128.45, 127.54, 123.61, 123.26, 120.24, 116.51, 114.26, 61.05, 55.32, 55.18, 31.60, 30.70, 22.32; HRMS (ESI-TOF) *m/z* Calcd for $C_{24}H_{25}NO_2Na$ [M+Na]$^+$: 382.1778, found: 382.1774.

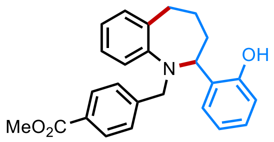

7p

Methyl-4-((2-(2-hydroxyphenyl)-2,3,4,5-tetrahydro-1*H*-benzo[*b*]azepin-1-yl)methyl)benzoate

7p was synthesized following the procedure. After purification by flash column chromatography using PE/EA (50/1) as the eluent, **7p** was obtained in 54% yield (42.1 mg) as a yellow solid. ^{1}H NMR (400 MHz, acetone-d_6) δ 8.74 (brs, 1H), 7.84 (d, *J* = 8.0 Hz, 2H), 7.60 (dd, *J* = 7.6, 1.6 Hz, 1H), 7.35 (d, *J* = 8.0 Hz, 2H), 7.13–7.02 (m, 3H), 6.96 (d, *J* = 7.6 Hz, 1H), 6.91–6.85 (m, 3H), 4.68 (dd, *J* = 7.0, 3.6 Hz, 1H), 4.31 (s, 2H), 3.82 (s, 3H), 3.16–3.08 (m, 1H), 2.92–2.80 (m, 1H), 1.95–1.86 (m, 1H), 1.74–1.59 (m, 3H); ^{13}C NMR (100 MHz, acetone-d_6) δ 167.07, 155.96, 147.37, 145.80, 136.76, 130.10, 130.02, 129.71, 129.44, 128.95, 128.62, 128.49, 127.57, 123.44, 122.18, 120.32, 116.44, 60.43, 55.39, 52.15, 31.51, 30.59, 21.78; HRMS (ESI-TOF) *m/z* Calcd for $C_{25}H_{26}NO_3$ [M+H]$^+$: 388.1907, found: 388.1902.

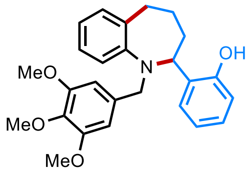

7q

2-(1-(3,4,5-Trimethoxybenzyl)-2,3,4,5-tetrahydro-1*H*-benzo[*b*]azepin-2-yl)phenol

7q was synthesized following the procedure. After purification by flash column chromatography using PE/EA (50/1) as the eluent, **7q** was obtained in 68% yield (57.3 mg) as a yellow solid. ^{1}H NMR (400 MHz, acetone-d_6) δ 8.89 (brs, 1H), 7.49 (dd, *J* = 7.6, 1.6 Hz, 1H), 7.13–7.08 (m, 3H), 7.02 (dd, *J* = 8.4, 1.2 Hz, 1H), 6.94–6.85 (m, 3H), 6.46 (s, 2H), 4.68 (dd, *J* = 8.0, 3.2 Hz, 1H), 4.22–4.10 (m, 2H), 3.68 (s, 6H), 3.64 (s, 3H), 3.04–2.97 (m, 1H), 2.89–2.83 (m, 1H), 1.93–1.86 (m, 1H), 1.78–1.64 (m, 3H); ^{13}C NMR (100 MHz, acetone-d_6) δ 156.16, 154.06, 147.93, 137.79, 137.02, 135.21, 130.47, 129.80, 128.71, 128.47, 127.69, 123.66, 123.23, 120.30, 116.49, 106.29, 60.41, 56.19, 56.14, 56.03, 31.69, 31.00, 22.69; HRMS (ESI-TOF) *m/z* Calcd for $C_{26}H_{29}NO_4Na$ [M+Na]$^+$: 442.1989, found: 442.1986.

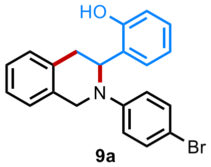

9a

2-(2-(4-Bromophenyl)-1,2,3,4-tetrahydroisoquinolin-3-yl)phenol

9a was synthesized following the procedure, using $Pd_2(dba)_3$ (4.6 mg, 2.5 mol%), **L8** (7.2 mg, 5.0 mol%). After purification by flash column chromatography using PE/EA (20/1) as the eluent, **9a** was obtained in 39% yield (29.7 mg) as a yellow solid. ^{1}H NMR (400 MHz, $CDCl_3$) δ 9.14 (brs, 1H), 7.33–7.30 (m, 2H), 7.22–7.08 (m, 5H), 7.00–6.95 (m, 3H), 6.84 (d, *J* = 8.0 Hz, 1H), 6.73 (t, *J* = 7.6 Hz, 1H), 4.94 (t, *J* = 5.2 Hz, 1H), 4.31 (q, *J* = 16.4 Hz,

2H), 3.26–3.09 (m, 2H); ^{13}C NMR (100 MHz, $CDCl_3$) δ 156.35, 148.39, 133.25, 132.94, 132.13, 128.96, 128.59, 127.50, 127.05, 126.66, 125.87, 124.97, 122.17, 119.89, 116.56, 115.68, 60.20, 50.35, 29.43; HRMS (ESI-TOF) *m/z* Calcd for $C_{21}H_{19}NOBr$ $[M+H]^+$: 380.0645, found: 380.0643.

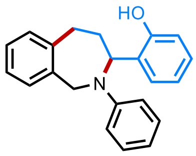

9b

2-(2-Phenyl-2,3,4,5-tetrahydro-1H-benzo[c]azepin-3-yl)phenol

9b was synthesized following the procedure, using $Pd_2(dba)_3$ (4.6 mg, 2.5 mol%), **L8** (7.2 mg, 5.0 mol%). After purification by flash column chromatography using PE/EA (15/1) as the eluent, **9b** was obtained in 39% yield (24.9 mg) as a yellow oil. ^{1}H NMR (400 MHz, acetone-d_6) δ 8.74 (brs, 1H), 7.44 (d, J = 7.2 Hz, 1H), 7.22–7.09 (m, 5H), 7.04–7.00 (m, 2H), 6.95 (d, J = 8.0 Hz, 1H), 6.81 (t, J = 7.6 Hz, 1H), 6.63 (d, J = 8.0 Hz, 2H), 6.52 (t, J = 7.2 Hz, 1H), 5.20 (d, J = 17.2 Hz, 1H), 5.07 (dd, J = 11.2, 4.8 Hz, 1H), 4.61 (d, J = 17.2 Hz, 1H), 3.13 (dd, J = 16.8, 6.8 Hz, 1H), 3.00–2.96 (m, 1H), 2.89–2.83 (m, 1H), 2.26–2.14 (m, 1H); ^{13}C NMR (100 MHz, acetone-d_6) δ 155.47, 150.65, 140.13, 139.92, 130.97, 129.73, 129.66, 128.93, 128.65, 127.65, 126.78, 126.63, 120.64, 116.95, 116.44, 112.64, 61.15, 50.62, 34.76, 32.29; HRMS (ESI-TOF) *m/z* Calcd for $C_{22}H_{22}NO$ $[M+H]^+$: 316.1696, found: 316.1698.

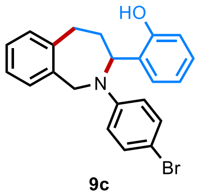

9c

2-(2-(4-Bromophenyl)-2,3,4,5-tetrahydro-1H-benzo[c]azepin-3-yl)phenol

9c was synthesized following the procedure, using $Pd_2(dba)_3$ (4.6 mg, 2.5 mol%), **L8** (7.2 mg, 5.0 mol%). After purification by flash column chromatography using PE/EA (15/1) as the eluent, **9c** was obtained in 46% yield (36.6 mg) as a white solid. ^{1}H NMR (400 MHz, acetone-d_6) δ 8.77 (brs, 1H), 7.43 (dd, J = 7.0, 1.6 Hz, 1H), 7.21–7.10 (m, 7H), 6.98–6.96 (m, 1H), 6.82 (t, J = 7.2 Hz, 1H), 6.56 (d, J = 9.2 Hz, 2H), 5.21 (d, J = 17.2 Hz, 1H), 5.03 (dd, J = 11.6, 4.8 Hz, 1H), 4.57 (d, J = 11.6 Hz, 1H), 3.13 (dd, J = 16.8, 6.8 Hz, 1H), 2.96–2.92 (m, 1H), 2.89–2.80 (m, 1H), 2.28–2.17 (m, 1H); ^{13}C NMR (100 MHz, acetone-d_6) δ 155.38, 149.76, 139.96, 139.19, 132.26, 131.03, 129.11, 128.94, 128.82, 127.78, 126.66, 126.59, 120.70, 116.45, 114.33, 108.09, 61.23, 50.45, 34.69, 32.12; HRMS (ESI-TOF) *m/z* Calcd for $C_{22}H_{21}NOBr$ $[M+H]^+$: 394.0801, found: 394.0805.

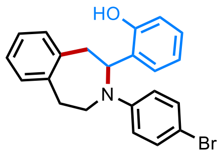

9e

2-(3-(4-Bromophenyl)-2,3,4,5-tetrahydro-1H-benzo[d]azepin-2-yl)phenol

9e was synthesized following the procedure. After purification by preparative thin-layer chromatography using PE/EA (10/1) as the eluent, **9e** was obtained in 33% yield (26.4 mg) as a yellow solid. ^{1}H NMR (400 MHz, $CDCl_3$) δ 8.94 (brs, 1H), 7.27 (d, *J* = 8.8 Hz, 2H), 7.18–7.14 (m, 3H), 7.11–7.02 (m, 3H), 6.95 (d, *J* = 8.8 Hz, 2H), 6.77–6.72 (m, 2H), 4.74 (d, *J* = 9.6 Hz, 1H), 3.82–3.68 (m, 2H), 3.40 (dt, *J* = 14.4, 5.2 Hz, 1H), 3.19–3.14 (m, 3H); ^{13}C NMR (100 MHz, $CDCl_3$) δ 155.12, 148.69, 139.89, 138.08, 132.07, 130.49, 129.22, 128.41, 127.77, 127.50, 126.92, 126.54, 122.11, 120.03, 116.72, 63.35, 52.61, 40.26, 34.29; HRMS (ESI-TOF) *m/z* Calcd for $C_{22}H_{21}NOBr$ $[M+H]^+$: 394.0801, found: 394.0799.

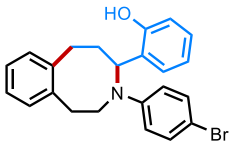

9f

2-(3-(4-Bromophenyl)-1,2,3,4,5,6-hexahydrobenzo[*d*]azocin-4-yl)phenol

9f was synthesized following the procedure. After purification by preparative thin-layer chromatography using PE/EA (10/1) as the eluent, **9f** was obtained in 19% yield (15.8 mg) as a yellow oil. ^{1}H NMR (400 MHz, $CDCl_3$) δ 7.55 (brs, 1H), 7.26–7.23 (m, 2H), 7.21–7.11 (m, 6H), 6.87 (td, *J* = 7.6, 1.2 Hz, 1H), 7.75–7.70 (m, 3H), 4.32–4.19 (m, 2H), 3.59–3.51 (m, 2H), 2.96–2.88 (m, 1H), 2.72–2.63 (m, 2H), 2.58–2.50 (m, 1H), 2.37–2.28 (m, 1H); ^{13}C NMR (100 MHz, $CDCl_3$) δ 156.46, 144.49, 139.72, 139.49, 132.00, 129.61, 129.20, 128.68, 127.36, 126.90, 125.70, 120.54, 116.71, 116.54, 110.87, 59.68, 53.80, 32.01, 31.93, 28.69; HRMS (ESI-TOF) *m/z* Calcd for $C_{23}H_{23}NOBr$ $[M+H]^+$: 408.0958, found: 408.0959.

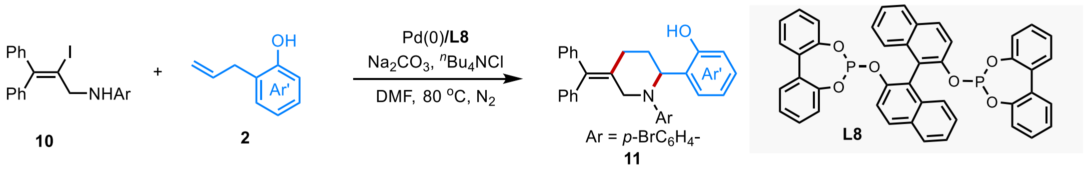

$Pd_2(dba)_3$ (2.8 mg, 1.5 mol%), **L8** (4.3 mg, 3.0 mol%), Na_2CO_3 (53.0 mg, 0.5 mmol) and ^{n}Bu_4NCl (111.2 mg, 0.4 mmol) were added to a 10 mL vial in dry box. The tube was sealed using a cap with PTFE cap liner and moved outside of the glovebox. DMF (3.0 mL) was added followed by addition of aniline derivatives **10** (0.4 mmol), alkene **2** (0.2 mmol). The reaction mixture was stirred at 80 °C for 18 h. After cooling to room temperature, the reaction mixture was diluted with ethyl acetate, and the resulted solution was washed with brine for three times. The organic phase was concentrated, and the residue was then purified by preparative thin-layer chromatography.

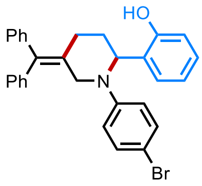

11a

2-(1-(4-Bromophenyl)-5-(diphenylmethylene)piperidin-2-yl)phenol

11a was synthesized following the procedure. After purification by preparative thin-layer chromatography using PE/EA (10/1) as the eluent, **11a** was obtained in 97% yield (96.4 mg) as a yellow solid. ^{1}H NMR (400 MHz, $CDCl_3$) δ 7.31–7.22 (m, 8H), 7.20–7.16 (m, 4H), 7.11–7.07 (m, 1H), 6.98 (dd, J = 7.6, 1.6 Hz, 1H), 6.87–6.80 (m, 2H), 6.36–6.32 (m, 2H), 4.78 (dd, J = 11.6, 2.0 Hz, 1H), 4.49 (brs, 1H), 4.00 (d, J = 12.4 Hz, 1H), 3.79 (d, J = 12.4 Hz, 1H), 2.80–2.67 (m, 2H), 2.26–2.14 (m, 1H), 2.06–2.00 (m, 1H); ^{13}C NMR (100 MHz, $CDCl_3$) δ 154.54, 147.04, 146.09, 141.03, 132.96, 131.67, 129.57, 129.01, 128.96, 128.24, 128.21, 127.50, 127.36, 127.28, 121.83, 120.42, 116.64, 114.53, 108.61, 77.56, 43.19, 26.68, 25.30; HRMS (ESI-TOF) *m/z* Calcd for $C_{30}H_{27}NOBr$ [M+H]$^+$: 496.1271, found: 496.1274.

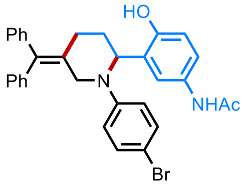

11b

_N_-(3-(1-(4-Bromophenyl)-5-(diphenylmethylene)piperidin-2-yl)-4-hydroxyphenyl)acetamide

11b was synthesized following the procedure. After purification by preparative thin-layer chromatography using PE/EA (10/1) as the eluent, **11b** was obtained in 70% yield (77.7 mg) as a yellow solid. ^{1}H NMR (400 MHz, $CDCl_3$) δ 7.29–7.22 (m, 10H), 7.17 (td, J = 6.8, 1.6 Hz, 4H), 7.06 (dd, J = 8.8, 2.4 Hz, 1H), 6.79 (d, J = 8.8 Hz, 1H), 6.33 (d, J = 8.8 Hz, 2H), 4.73 (dd, J = 11.6, 2.0 Hz, 1H), 4.42 (brs, 1H), 3.99 (d, J = 12.4 Hz, 1H), 3.77 (d, J = 12.4 Hz, 1H), 2.75–2.63 (m, 2H), 2.21–2.09 (m, 4H), 2.03–1.97 (m, 1H); ^{13}C NMR (100 MHz, $CDCl_3$) δ 168.21, 151.48, 147.01, 146.19, 141.02, 140.97, 132.89, 131.67, 128.97, 128.92, 128.24, 128.21, 127.50, 127.39, 122.15, 121.93, 119.99, 116.74, 114.52, 108.62, 77.62, 43.16, 26.57, 25.41, 24.26; HRMS (ESI-TOF) *m/z* Calcd for $C_{32}H_{29}N_2O_2BrNa$ [M+Na]$^+$: 575.1305, found: 575.1306.

Unsuccessful Substrates with Respect to 2-Iodoaniline Derivatives

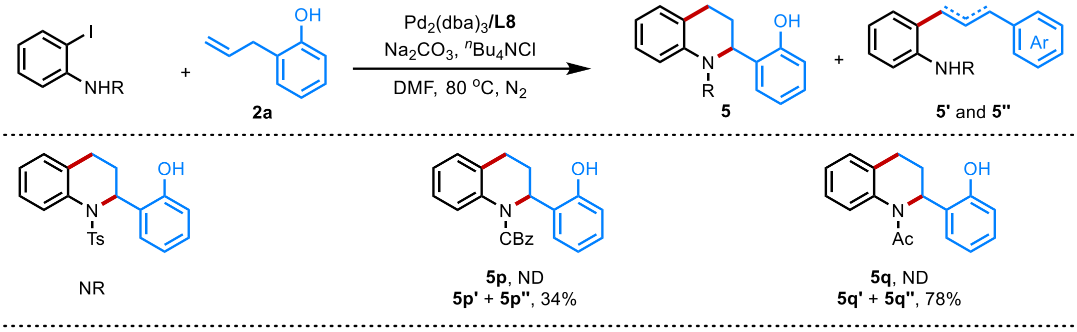

The yield was determined by the analysis of the crude ^{1}H NMR using dibromomethane as the internal standard.

Substrates with Respect to *ortho*-Iodide Benzylamines

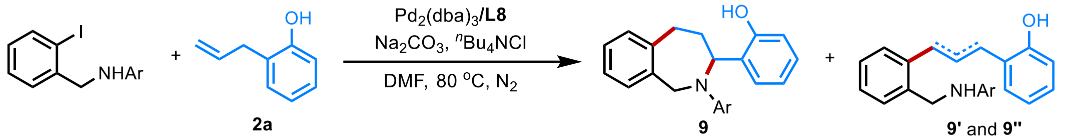

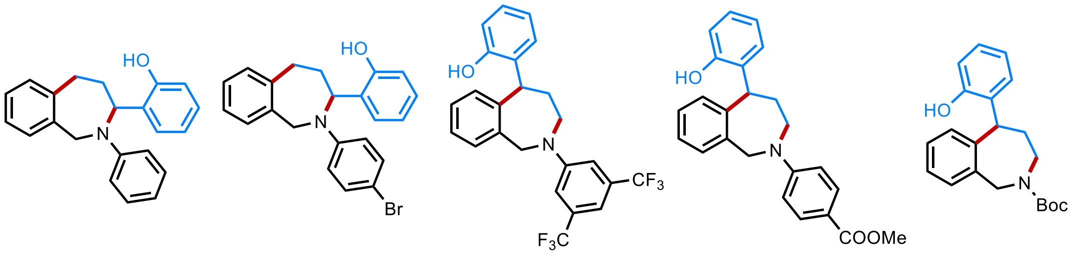

**9b**, 39%	**9c**, 46%	**9h**, 66%	**9i**, ND	**9d**, ND
**9b'** + **9b''**, 26%	**9c'** + **9c''**, 30%	**9h'** + **9h''**, 34%	**9i'** + **9i''**, 61%	**9d'** + **9d''**, 96%

The yield of **9'** and **9''** was determined by the analysis of the crude ^{1}H NMR using dibromomethane as the internal standard.

Unsuccessful Substrates with Respect to *ortho*-Iodide Phenylethanamines

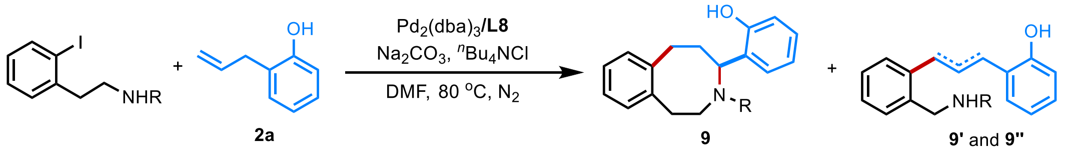

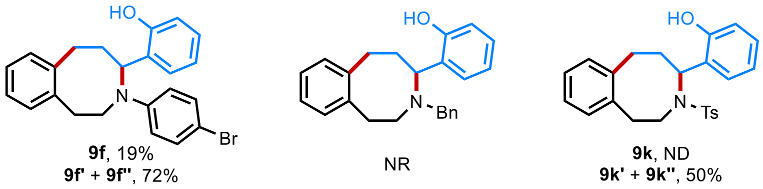

**9f**, 19%	NR	**9k**, ND
**9f'** + **9f''**, 72%		**9k'** + **9k''**, 50%

The yield of **9'** and **9''** was determined by analysis of the crude ^{1}H NMR using dibromomethane as the internal standard.
NR, no reaction; ND, not detected

Unsuccessful Substrates with Respect to Alkenes

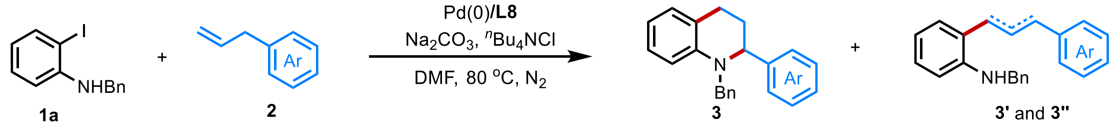

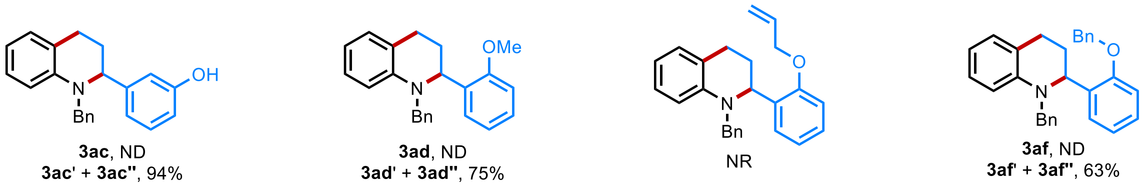

**3ac**, ND	**3ad**, ND	NR	**3af**, ND
**3ac'** + **3ac''**, 94%	**3ad'** + **3ad''**, 75%		**3af'** + **3af''**, 63%

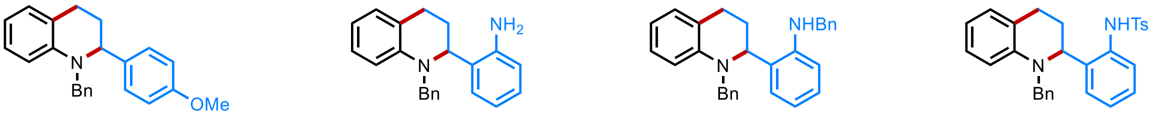

**3ag**, ND	**3ah**, ND	**3ai**, ND	**3ak**, ND
**3ag'** + **3ag''**, 88%	**3ah'** + **3ah''**, 38%	**3ai'** + **3ai''**,65%	**3ak'** + **3ak''**,17%

The yield was determined by the analysis of the crude ^{1}H NMR using dibromomethane as the internal standard.

2.4 Gram-Scale Reaction and Synthetic Application

2.4.1 Gram-Scale Reaction

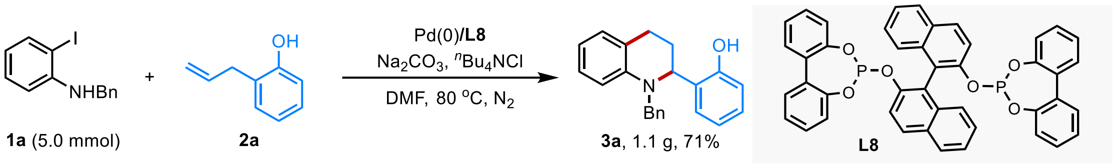

$Pd_2(dba)_3$ (68.7 mg, 0.075 mmol), **L8** (107.2 mg, 0.15 mmol), Na_2CO_3 (1.3 g, 12.5 mmol) and ^{n}Bu_4NCl (2.8 g, 10.0 mmol) were added to a 350 mL Schlenk flask in dry box. The bottle was moved outside of the glovebox. DMF (75.0 mL) was added followed by the addition of aniline derivatives **1a** (3.1 g, 10 mmol), alkene **2** (670.9 mg, 5.0 mmol). The mixture was stirred at 80 °C for 48 h. After cooling to room temperature, the mixture was diluted with ethyl acetate, and the resulted solution was washed with brine for three times. The organic phase was concentrated, and the residue was then purified by flash column chromatography using PE/EA (15/1) as the eluent, **3a** was obtained in 71% yield (1.1 g) as a yellow oil.

2.4.2 Synthetic Application

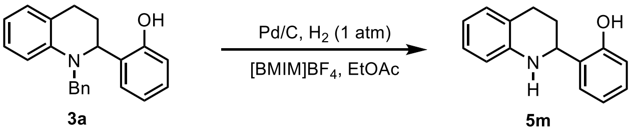

3a (31.5 mg, 0.1 mmol), 10% Pd/C (53.2 mg, 0.05 mmol), [bmim]BF_4 (1.0 mL) and EA (1.0 mL) were added to a 50 mL round bottle. The bottle was back-filled with hydrogen three times, and stirred under H_2 atmosphere at 30 °C overnight. Upon completion, the mixture was filtered through a pad of celite using ethyl acetate as the eluent, and the filtrate was concentrated. After purification by flash column chromatography on silica gel (PE/EA = 15/1), **5m** was obtained in 77% yield (19.7 mg) as a yellow oil.

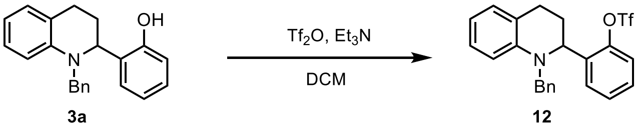

3a (652.9 mg, 2.1 mmol) was added to a 100 mL Schlenk flask under N_2, followed by the injection of DCM (20 mL) and NEt_3 (0.6 mL, 4.1 mmol) Tf_2O was added dropwise at 0 °C, and then the mixture was warmed to room temperature gradually. Upon completion, the mixture was quenched with H_2O at 0 °C and extracted with DCM for three times. The combined organic layers were washed with brine, dried over Na_2SO_4, filtered, and concentrated under vacuum. After purification by flash column chromatography on silica gel (PE/EA = 20/1), **12** was obtained in 82% yield (774.8 mg) as a white solid. ^{1}H NMR (400 MHz, $CDCl_3$) δ 7.37–7.28 (m, 6H), 7.26–7.21 (m, 3H), 7.07–7.00 (m, 2H), 6.66 (td, J = 7.2, 1.2 Hz, 1H), 6.60 (d, J = 8.0 Hz, 1H), 5.04 (t, J = 4.0 Hz, 1H), 4.66 (d, J = 17.2 Hz, 1H), 4.17 (d, J = 17.2 Hz, 1H), 2.71–2.53 (m, 2H), 2.38–2.29 (m, 1H), 2.17–2.11 (m, 1H); ^{13}C NMR (100 MHz, $CDCl_3$) δ 146.73, 144.79, 137.90, 136.29, 129.54, 128.95, 128.66, 128.25, 127.57, 126.90, 126.26, 121.51,

121.44, 118.42 (q, J = 320 Hz), 116.28, 110.76, 56.25, 53.16, 27.16, 23.38; ^{19}F NMR (375 MHz, $CDCl_3$) δ -73.83–-73.87 (m); HRMS (ESI-TOF) m/z Calcd for $C_{23}H_{21}NO_3F_3S$ $[M+H]^+$: 448.1189, found: 448.1192.

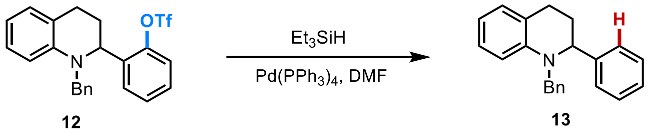

12 (44.7 mg, 0.1 mmol) and $Pd(PPh_3)_4$ (5.8 mg, 0.005 mmol) were added to a 10 mL vial in dry box. The tube was sealed using a cap with PTFE cap liner and moved outside of the glovebox. DMF (1.0 mL) was added followed by addition of Et_3SiH (63.7 μL, 0.4 mmol). The mixture was stirred at 110 °C for 14 h. After cooling to room temperature, the mixture was diluted with EA, and the resulted solution was washed with brine for three times. The organic phase was concentrated. After purification by preparative thin-layer chromatography using PE/EA (15/1) as the eluent, **13**[17] was obtained in 99% yield (29.6 mg) as a white solid. ^{1}H NMR (400 MHz, $CDCl_3$) δ 7.32–7.27 (m, 5H), 7.23–7.17 (m, 5H), 7.01 (dd, J = 10.8, 7.2 Hz, 2H), 6.61 (t, J = 7.2 Hz, 1H), 6.55 (d, J = 8.0 Hz, 1H), 4.71 (d, J = 17.2 Hz, 2H), 4.23 (d, J = 17.2 Hz, 1H), 2.68–2.57 (m, 2H), 2.34–2.25 (m, 1H), 2.10–2.05 (m, 1H).

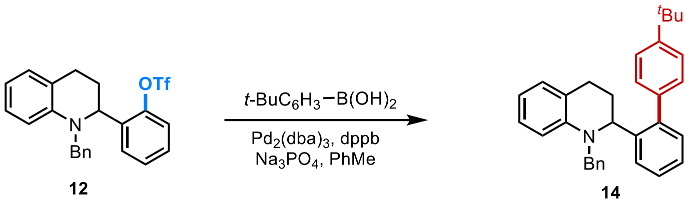

12 (44.7 mg, 0.1 mmol) and $Pd_2(dba)_3$ (4.6 mg, 0.005 mmol), dppb (8.5 mg, 0.02 mmol), Na_3PO_4 (49.2 mg, 0.3 mmol) and ^{t}Bu-C_6H_3-$B(OH)_2$ (35.6 mg, 0,2 mmol) were added to a 10 mL vial in dry box. The tube was sealed using a cap with PTFE cap liner and moved outside of the glovebox. Toluene (1.0 mL) was added and mixture was then stirred at 110 °C for 14 h. After cooling to room temperature, the mixture was filtered through a pad of celite and concentrated. After purification by preparative thin-layer chromatography using PE/DCM (5/1) as the eluent, **14** was obtained in 71% yield (30.7 mg) as a white solid. ^{1}H NMR (400 MHz, $CDCl_3$) δ 7.35–7.24 (m, 7H), 7.22–7.15 (m, 4H), 7.10 (dd, J = 8.4, 2.4 Hz, 2H), 7.00 (td, J = 7.6, 2.4 Hz, 2H), 6.60 (td, J = 7.2, 2.4 Hz, 1H), 6.53 (dd, J = 8.0, 2.4 Hz, 1H), 4.89 (q, J = 3.6 Hz, 1H), 4.63 (dd, J = 17.2, 2.4 Hz, 1H), 4.24 (dd, J = 17.2, 2.4 Hz, 1H), 2.71–2.53 (m, 2H), 2.04–1.95 (m, 1H), 1.88–1.81 (m, 1H), 1.33 (s, 9H); ^{13}C NMR (100 MHz, $CDCl_3$) δ 149.72, 145.69, 141.24, 141.10, 138.81, 137.92, 130.65, 128.76, 128.54, 128.50, 127.27, 127.20, 127.05, 126.64, 126.60, 126.44, 125.12, 122.25, 115.63, 110.73, 57.81, 53.19, 34.48, 31.35, 28.32, 23.81; HRMS (ESI-TOF) m/z Calcd for $C_{32}H_{34}N$ $[M+H]^+$: 432.2686, found: 432.2690.

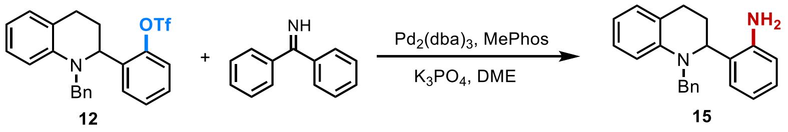

12 (44.7 mg, 0.1 mmol) and $Pd_2(dba)_3$ (9.1 mg, 0.01 mmol), MePhos (14.6 mg, 0.04 mmol) and K_3PO_4 (31.8 mg, 1.15 mmol) were added to a 10 mL vial in dry box. The tube was sealed using a cap with PTFE cap liner and moved outside of the glovebox. DME (1.0 mL) was added followed by addition of diphenylmethanimine (21.0 µL, 0.13 mmol). The mixture was stirred at 110 °C for 14 h. After cooling to room temperature, quenched with 3.0 M HCl (aq.), and then neutralized with NaOH (aq.). The mixture was extracted with EA for three times, and the combined organic layers were washed with brine, dried over Na_2SO_4, filtered and concentrated under vacuum. After purification by preparative thin-layer chromatography using PE/EA (15/1) as the eluent, **15** was obtained in 60% yield (18.8 mg) as a white solid. ^{1}H NMR (400 MHz, $CDCl_3$) δ 7.31–7.27 (m, 2H), 7.24–7.18 (m, 3H), 7.10–7.00 (m, 4H), 6.74–6.70 (m, 1H), 6.68–6.61 (m, 3H), 4.72 (d, *J* = 17.2 Hz, 1H), 4.65 (t, *J* = 4.8 Hz, 1H), 4.20 (d, *J* = 17.2 Hz, 1H), 3.70 (brs, 2H), 2.71–2.67 (m, 2H), 2.29–2.14 (m, 2H); ^{13}C NMR (100 MHz, $CDCl_3$) δ 145.14, 143.53, 138.31, 128.75, 128.56, 128.21, 127.86, 127.43, 126.87, 126.70, 126.51, 122.37, 118.54, 116.43, 116.03, 110.92, 58.07, 52.11, 25.96, 24.62; HRMS (ESI-TOF) *m/z* Calcd for $C_{22}H_{23}N_2$ $[M+H]^+$: 315.1856, found: 315.1863.

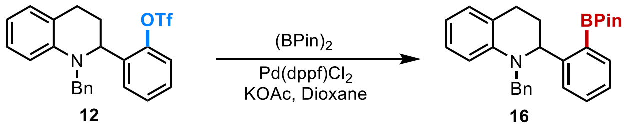

12 (44.7 mg, 0.1 mmol) and $Pd(dppf)Cl_2$ (43.7 mg, 0.005 mmol), KOAc (29.4 mg, 0.3 mmol) and $(Bpin)_2$ (50.8 mg, 0,2 mmol) were added to a 10 mL vial in dry box. The tube was sealed using a cap with PTFE cap liner and moved outside of the glovebox. Dioxane (1.0 mL) was added then mixture was stirred at 110 °C for 14 h. After cooling to room temperature, the mixture was filtered through a pad of celite and concentrated. After purification by preparative thin-layer chromatography using PE/EA (25/1) as the eluent, **16** was obtained in 56% yield (23.8 mg) as a colorless oil. ^{1}H NMR (400 MHz, $CDCl_3$) δ 7.81 (d, *J* = 7.2 Hz, 1H), 7.33–7.19 (m, 8H), 7.04–6.99 (m, 2H), 6.61–6.54 (m, 2H), 5.42 (t, *J* = 4.0 Hz, 1H), 4.65 (d, *J* = 17.2 Hz, 1H), 4.20 (d, *J* = 17.2 Hz, 1H), 2.62–2.55 (m, 2H), 2.31–2.22 (m, 1H), 2.10–2.04 (m, 1H), 1.28 (s, 6H), 1.24 (s, 6H); ^{13}C NMR (100 MHz, $CDCl_3$) δ 150.62, 145.59, 138.96, 136.29, 130.68, 128.75, 128.45, 127.21, 126.48, 126.40, 126.04, 122.31, 115.21, 110.24, 83.58, 59.71, 53.03, 29.60, 24.83, 24.62, 23.59; HRMS (ESI-TOF) *m/z* Calcd for $C_{28}H_{33}NBO_2$ $[M+H]^+$: 426.2599, found: 426.2603.

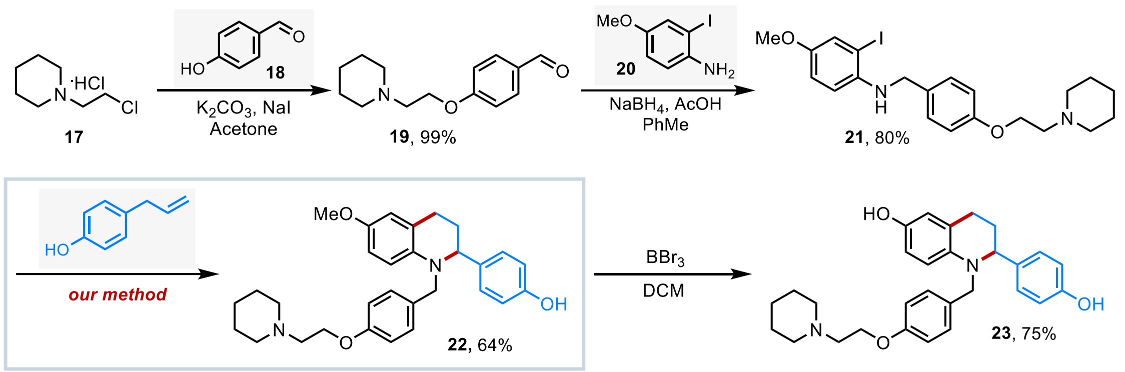

18 (1.2 g, 10.0 mmol), **17** (2.8 g, 15 mmol), K_2CO_3 (4.1 g, 30.0 mmol) and NaI (37.5 mg, 0,25 mmol) were added to a 250 mL round bottle. Acetone (50 mL) was added, and the reaction mixture was heated to reflux overnight. After cooling to room temperature, the mixture was filtered through a pad of celite and concentrated under vacuum. After purification by column chromatography on silica gel using PE/EA (1/1) as the eluent, **19** was obtained in 99% yield (2.6 g) as a light-yellow liquid. ^{1}H NMR (400 MHz, $CDCl_3$) δ 9.88 (s, 1H), 7.83 (d, J = 8.8 Hz, 1H), 7.01 (d, J = 8.8 Hz, 2H), 4.19 (t, J = 6.0 Hz, 2H), 2.80 (t, J = 6.0 Hz, 2H), 2.53–2.50 (m, 4H), 1.64–1.59 (m, 4H), 1.48–1.44 (m, 2H).

20 (1.1 g, 4.3 mmol) and toluene (50 mL) were added to a dried three necked bottle under N_2. **19** (1.7 g, 7.4 mmol) and AcOH (10 mL) were added and the reaction mixture was heated to reflux with a Dean-Stark apparatus for 4 h. Then $NaBH_4$ (325.3 mg, 8.6 mmol) was added portion-wise at 0 ºC. The mixture was warmed up to room temperature and stirred overnight. The reaction was quenched with saturated $NaHCO_3$ (aq.) carefully, and was extracted with EA for three times. The combined organic layers were washed with brine, dried over Na_2SO_4, filtered and concentrated under vacuum. After purification by column chromatography on silica gel using DCM/CH_3OH (20/1) as the eluent, **21** was obtained in 80% yield (1.6 g) as a brown oil. ^{1}H NMR (400 MHz, $CDCl_3$) δ 7.29–7.26 (m, 3H), 6.99–6.87 (m, 2H), 6.80 (dd, J = 8.8, 2.8 Hz, 1H), 6.50 (d, J = 8.8 Hz, 1H), 4.26 (s, 2H), 4.18 (brs, 1H), 4.10 (t, J = 6.0 Hz, 2H), 3.72 (s, 3H), 2.77 (t, J = 6.0 Hz, 2H), 2.51 (br, 4H), 1.64–1.58 (m, 4H), 1.48 (br, 2H).

$Pd_2(dba)_3$ (13.7 mg, 0.015 mmol), Na_2CO_3 (265.0 g, 2.5 mmol) and ^{n}Bu$_4$NCl (555.8 mg, 2.0 mmol) were added to a 100 mL Schlenk flask in dry box. The bottle was moved outside of the glovebox. DMF (15.0 mL) was added followed by addition of aniline derivatives **21** (466.4 mg, 1.0 mmol), 4-allylphenol (134.2 mg, 1.0 mmol). The mixture was stirred at 80 °C for 36 h. After cooling to room temperature, the mixture was diluted with EA, and the resulted solution was washed with brine for three times. The combined organic layers were concentrated, and the residue was then purified by flash column chromatography on silica gel using DCM/CH_3OH (30/1) as the eluent, **22** was obtained in 64% yield (300.9 mg) as a brown solid. ^{1}H NMR (400 MHz, $CDCl_3$) δ 7.78 (brs, 1H), 7.02 (d, J = 8.2 Hz, 2H), 6.96 (d, J = 8.0 Hz, 2H), 6.70 (d, J = 8.0 Hz, 2H), 6.66–6.59 (m, 4H), 6.42 (d, J = 8.8 Hz, 1H), 4.53 (t, J = 4.0 Hz, 1H), 4.46 (d, J = 17.2 Hz, 1H), 4.13–4.04 (m, 3H), 3.72 (s, 3H), 2.80 (t, J = 6.0 Hz, 2H), 2.68–2.54 (m, 6H), 2.25–2.18 (m, 1H), 2.03–1.97 (m, 1H), 1.69–1.63 (m, 4H), 1.48–1.44 (m, 2H); ^{13}C NMR (100 MHz, $CDCl_3$) δ

157.33, 155.44, 150.26, 139.83, 135.16, 131.08, 127.83, 127.29, 123.41, 115.54, 114.97, 114.50, 112.39, 111.01, 65.02, 60.67, 57.81, 55.71, 54.89, 52.61, 29.93, 25.24, 24.08, 23.90; HRMS (ESI-TOF) *m/z* Calcd for $C_{30}H_{37}N_2O_3$ $[M+H]^+$: 473.2799, found: 473.2796.

22 (59.4 mg, 0.13 mmol) and DCM (20.0 mL) were added to a dried Schlenk flask under N_2. The solution of BBr_3 (0.38 mmol, in 2.0 mL DCM) was added dropwise at 0 ^oC. Then the mixture was warmed to room temperature. The reaction was quenched with saturated $NaHCO_3$ (aq.) carefully, extracted with DCM for three times. The organic layers were combined and washed with brine, dried over Na_2SO_4, filtered and concentrated under vacuum. After purification by preparative thin-layer chromatography using DCM/CH_3OH (15/1) as the eluent, **23** was obtained in 75% yield (44.7 mg) as a yellow solid. ^1H NMR (400 MHz, CD_3OD) δ 7.08 (d, *J* = 8.4 Hz, 2H), 6.97 (d, *J* = 8.0 Hz, 2H), 6.82 (d, *J* = 8.0 Hz, 2H), 6.69 (d, *J* = 8.0 Hz, 2H), 6.46–6.37 (m, 3H), 4.59–4.43 (m, 2H), 4.09–4.01 (m, 3H), 3.35–3.44 (m, 2H), 2.78 (t, *J* = 5.6 Hz, 2H), 2.62–2.51 (m, 6H), 2.17–2.14 (m, 1H), 1.99–1.96 (m, 1H), 1.66–1.60 (m, 4H), 1.49–1.47 (m, 2H); ^{13}C NMR (100 MHz, CD_3OD) δ 158.88, 157.30, 148.45, 140.55, 136.42, 133.05, 128.91, 128.88, 125.05, 116.78, 116.05, 115.53, 114.77, 113.10, 66.18, 62.25, 58.91, 55.85, 53.95, 31.50, 26.29, 25.52, 24.87; HRMS (ESI-TOF) *m/z* Calcd for $C_{29}H_{35}N_2O_3$ $[M+H]^+$: 459.2642, found: 459.2645.

3. Mechanistic Study

3.1 Deuteration Experiments

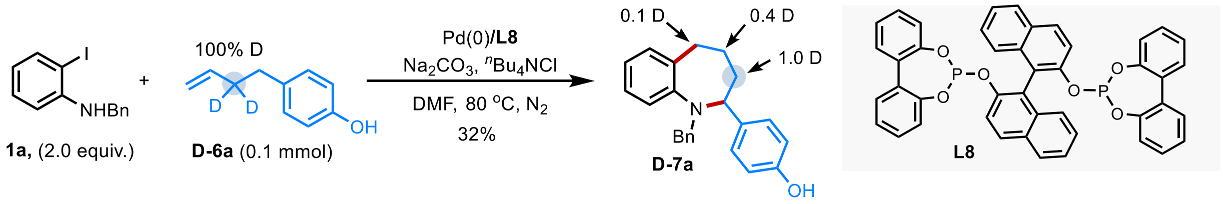

Pd2(dba)3 (1.4 mg, 1.5 mol%), **L8** (2.3 mg, 3.0 mol%), Na_2CO_3 (26.5 mg, 0.25 mmol) and $^{n}Bu_4NCl$ (55.6 mg, 0.2 mmol) were added to a 10 mL vial in dry box. The tube was sealed using a cap with PTFE cap liner and moved outside of the glovebox. DMF (1.0 mL) was added followed by addition of aniline derivatives **1a** (78 μL, 0.2 mmol), alkene **D-6a** (15.0 mg, 0.1 mmol). The reaction mixture was stirred at 80 °C for 18 h. After cooling to room temperature, the reaction mixture was diluted with EA, and the resulted solution was washed with brine for three times. The organic phase was concentrated, and the residue was then purified by preparative thin-layer chromatography using PE/EA(10/1) as the eluent, **D-7a** was obtained in 32% yield (10.7 mg) as a yellow oil.

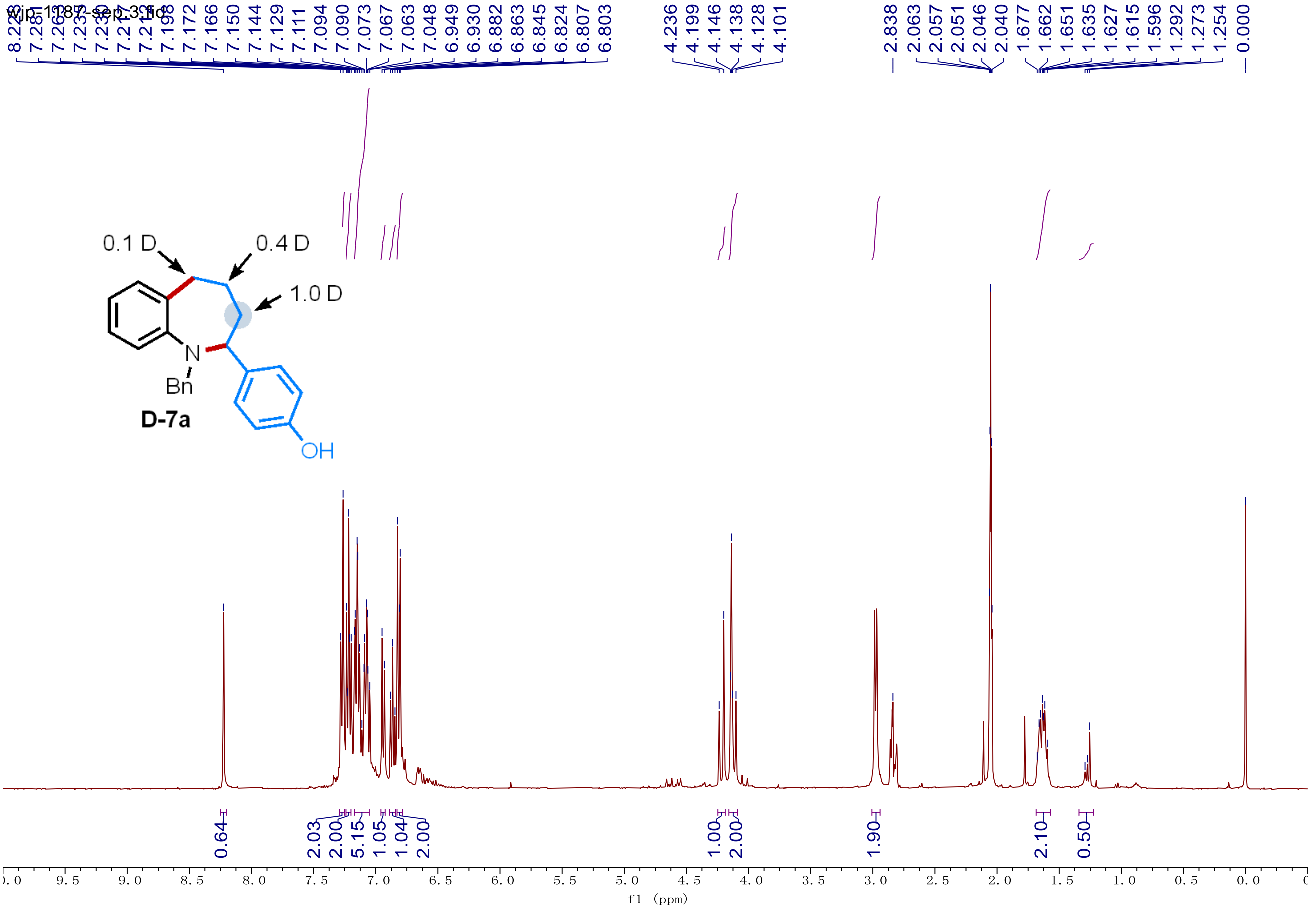

Supplementary Figure 1. ^{1}H NMR (400 MHz, CDCl$_3$) spectrum of D-7a

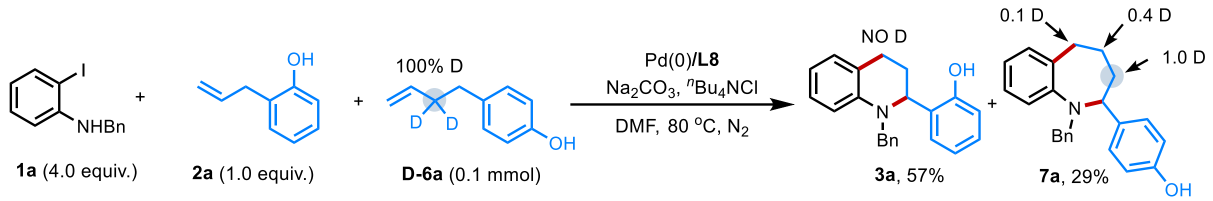

$Pd_2(dba)_3$ (2.8 mg, 1.5 mol%), **L8** (4.3 mg, 3.0 mol%), Na_2CO_3 (53.0 mg, 0.5 mmol) and $^{n}Bu_4NCl$ (111.2 mg, 0.4 mmol) were added to a 10 mL vial in dry box. The tube was sealed using a cap with PTFE cap liner and moved outside of the glovebox. DMF (3.0 mL) was added followed by addition of aniline derivatives **1a** (78 μL, 0.2 mmol), alkene **D-6a** (15.0 mg, 0.1 mmol) and alkene **2a** (13 μL, 0.1 mmol). The reaction mixture was stirred at 80 °C for 18 h. After cooling to room temperature, the reaction mixture was diluted with EA, and the resulted solution was washed with brine for three times. The organic phase was concentrated, and the residue was then purified by preparative thin-layer chromatography using PE/EA (8/1) as the eluent, **3a** was obtained in 57% yield (18.1 mg) as a yellow oil, **7a** was obtained in 29% yield (9.7 mg) as a yellow oil.

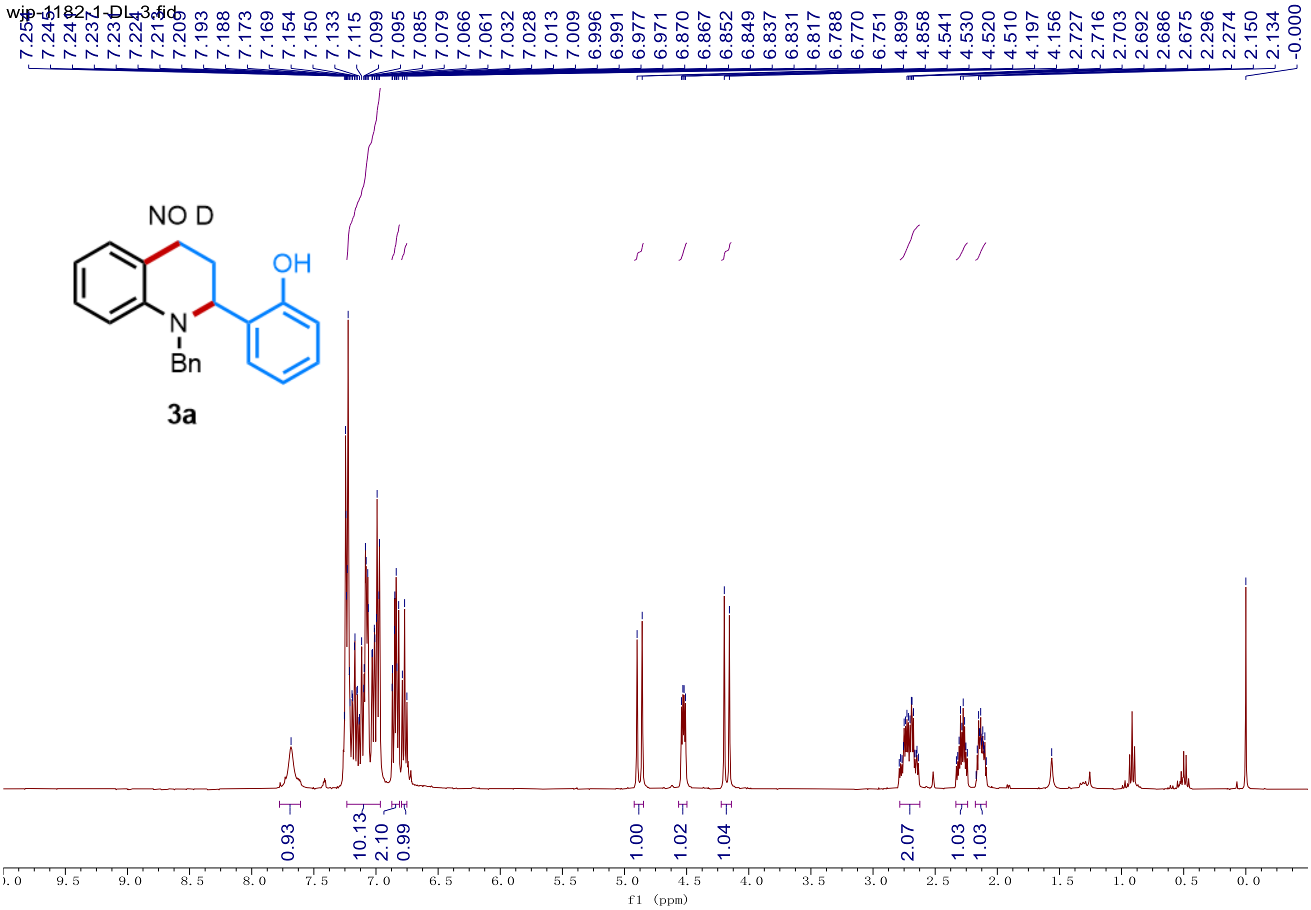

Supplementary Figure 2. ^{1}H NMR (400 MHz, $CDCl_3$) spectrum of 3a.

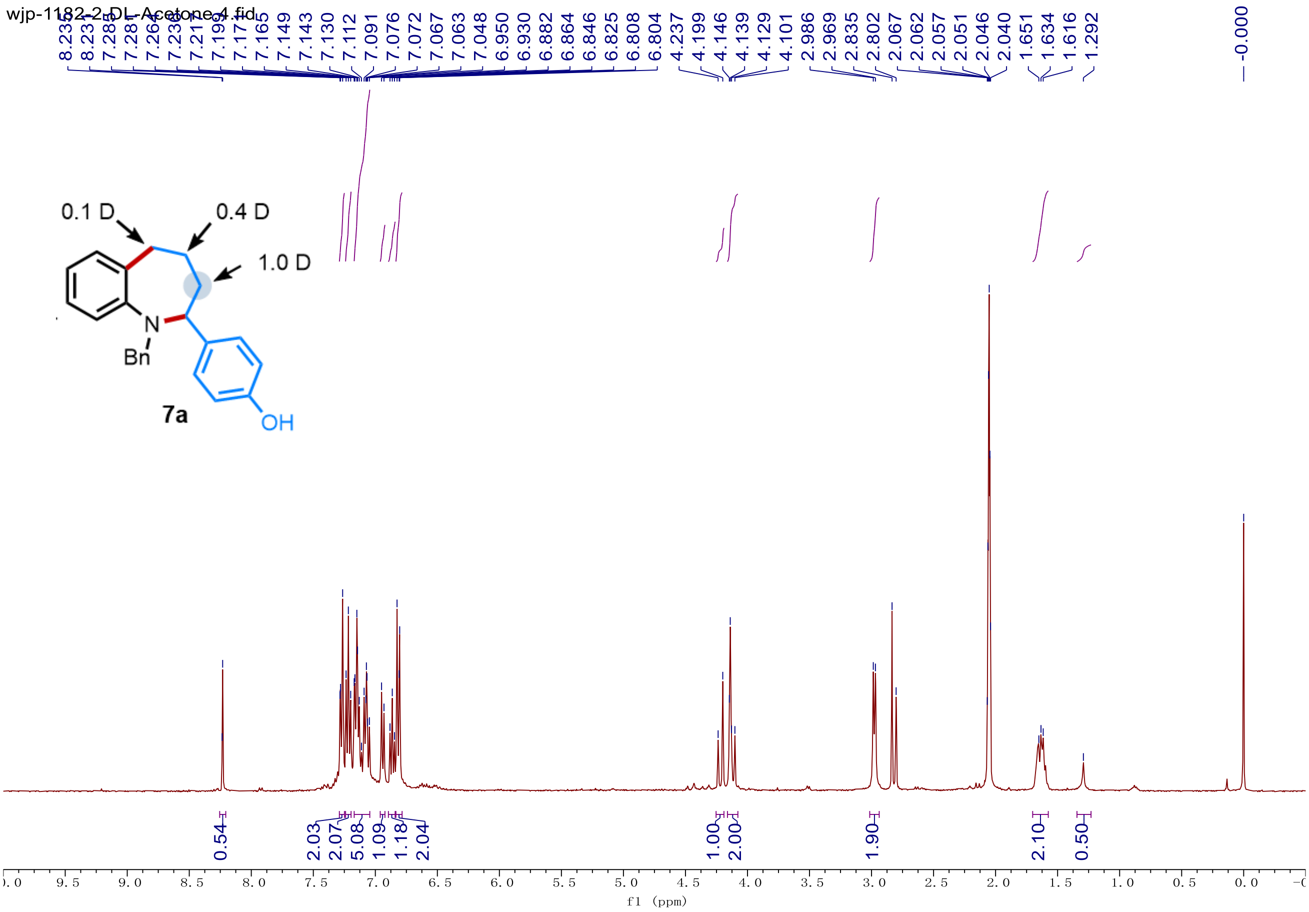

Supplementary Figure 3. ^{1}H NMR (400 MHz, $CDCl_3$) spectrum of 7a.

3.2 Transformation of Isolated Heck-Product

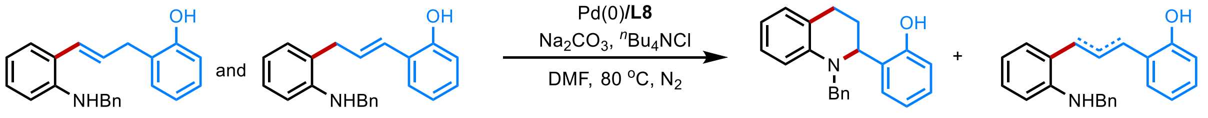

3a' **3a''** **3a** >99%, **3a'/3a''** = 1.25/1.0

mixture of **3a'** and **3a''** (**3a'/3a''** = 1.25/1.0)

Isolated Heck-product **3a’** and **3a’’** (15.8 mg, 0.05 mmol, **3a’/3a’’** = 1/25/1.0), $Pd_2(dba)_3$ (0.7 mg, 1.5 mol%), **L8** (1.1 mg, 3.0 mol%), Na_2CO_3 (13.3 mg, 0.125 mmol) and $^nBu_4NCl$ (27.8 mg, 0.1 mmol) were added to a 10 mL vial in dry box. The tube was sealed using a cap with PTFE cap liner and moved outside of the glovebox. DMF (0.5 mL) was added, and the reaction mixture was stirred at 80 °C for 18 h. After cooling to room temperature, the reaction mixture was diluted with EA, and the resulted solution was washed with brine for three times. The organic phase was concentrated, and the residue was then purified by preparative thin-layer chromatography using PE/EA (15/1) as the eluent, the mixture of **3a’** and **3a’’** was recycled in 99% yield (15.6 mg, **3a’/3a’’** = 1/25/1.0) as a yellow oil.

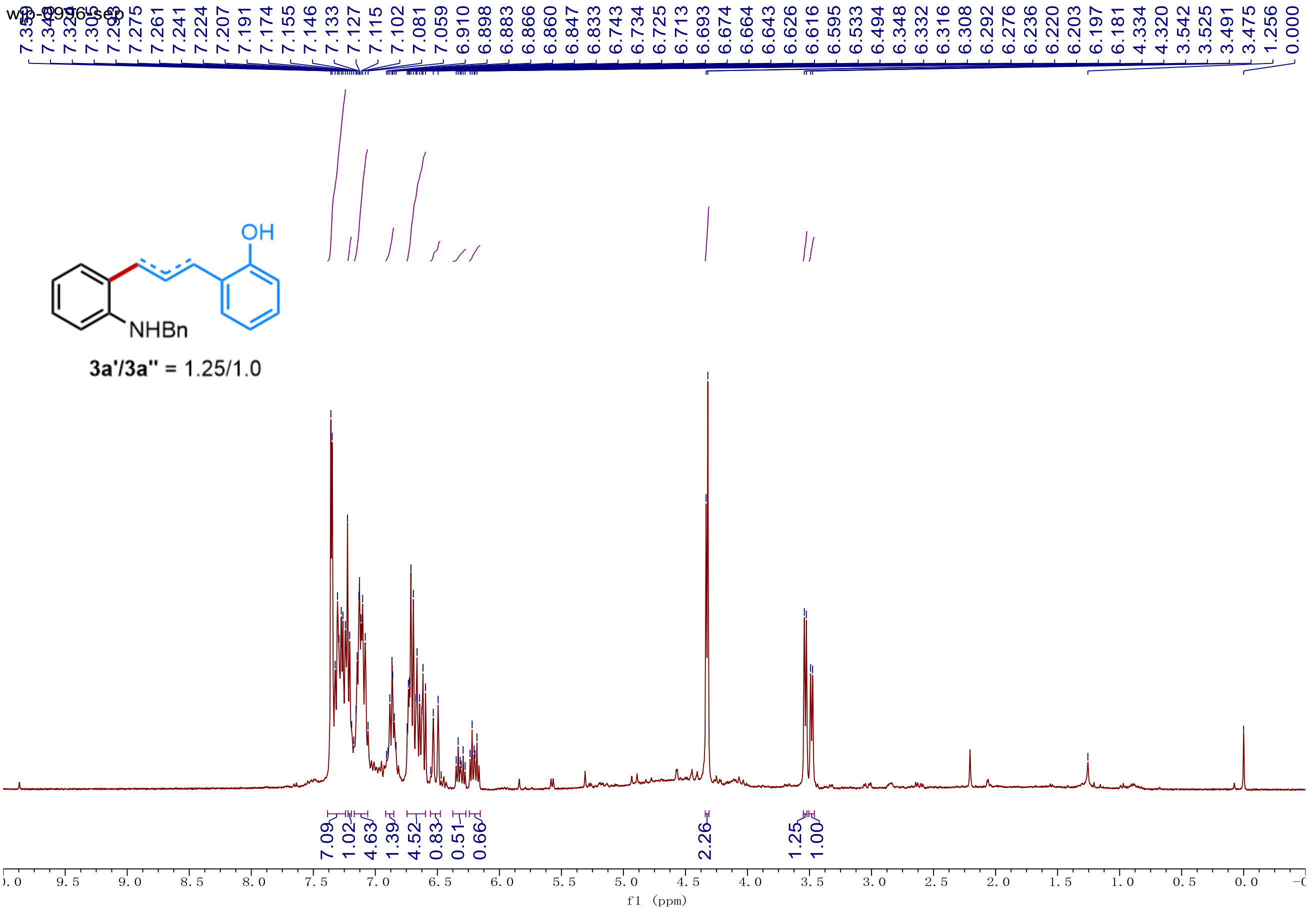

Supplementary Figure 4. ^{1}**H NMR (400 MHz,** $CDCl_3$**) spectrum of 3a’ and 3a’’.**

4. X-Ray Structure

4.1 X-Ray structure of compound 3g (cddc 2161960)

Single crystals of **3g** were obtained by recrystallization from DCM. The molecular structure and X-ray diffractional data/refinement of **3g** were shown below.

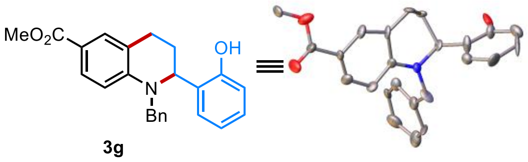

Bond precision:	C-C = 0.0031 A		Wavelength=0.71073
Cell:	a=10.6651(3)	b=12.9187(4)	c=15.5070(5)
	alpha=82.518(1)	beta=71.969(1)	gamma=78.139(1)
Temperature:	293 K		

	Calculated	Reported
Volume	1983.14(11)	1983.14(11)
Space group	P -1	P -1
Hall group	-P 1	-P 1
Moiety formula	C24 H23 N O3	C24 H23 N O3
Sum formula	C24 H23 N O3	C24 H23 N O3
Mr	373.43	373.43
Dx,g cm-3	1.251	1.251
Z	4	4
Mu (mm-1)	0.082	0.082
F000	792.0	792.0
F000'	792.36	
h,k,lmax	12,15,18	12,15,18
Nref	7392	7378
Tmin,Tmax	0.987,0.992	0.688,0.746
Tmin'	0.987	

Correction method= # Reported T Limits: Tmin=0.688 Tmax=0.746 AbsCorr = MULTI-SCAN

Data completeness= 0.998 Theta(max)= 25.497

R(reflections)= 0.0476(5150) wR2(reflections)= 0.1164(7378)

S = 1.045 Npar= 510

4.2 X-Ray structure of compound 7p (cddc 2161962)

Single crystals of **7p** were obtained by recrystallization from DCM. The molecular structure and X-ray diffractional data/refinement of **7p** were shown below.

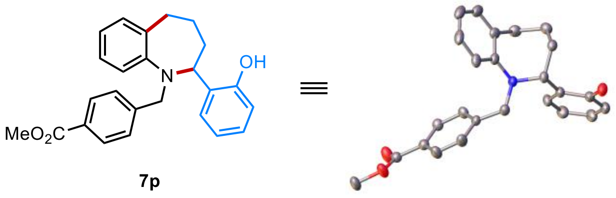

Bond precision: C-C = 0.0052 A Wavelength=0.71073

Cell:	a=22.629(3)	b=8.4039(10)	c=22.647(2)
	alpha=90	beta=108.084(3)	gamma=90
Temperature:	213 K		

	Calculated	Reported
Volume	4094.1(8)	4094.0(8)
Space group	P 21/n	P 21/n
Hall group	-P 2yn	-P 2yn
Moiety formula	C25 H25 N O3	C25 H25 N O3
Sum formula	C25 H25 N O3	C25 H25 N O3
Mr	387.46	387.46
Dx,g cm-3	1.257	1.257
Z	8	8
Mu (mm-1)	0.082	0.082
F000	1648.0	1648.0
F000'	1648.74	
h,k,lmax	27,10,27	27,10,27
Nref	8052	8043
Tmin,Tmax	0.985,0.991	0.618,0.746
Tmin'	0.985	

Correction method= # Reported T Limits: Tmin=0.618 Tmax=0.746 AbsCorr = MULTI-SCAN

Data completeness= 0.999 Theta(max)= 26.000

R(reflections)= 0.0682(6128) wR2(reflections)= 0.1879(8043)

S = 1.063 Npar= 548

5. NMR Spectra

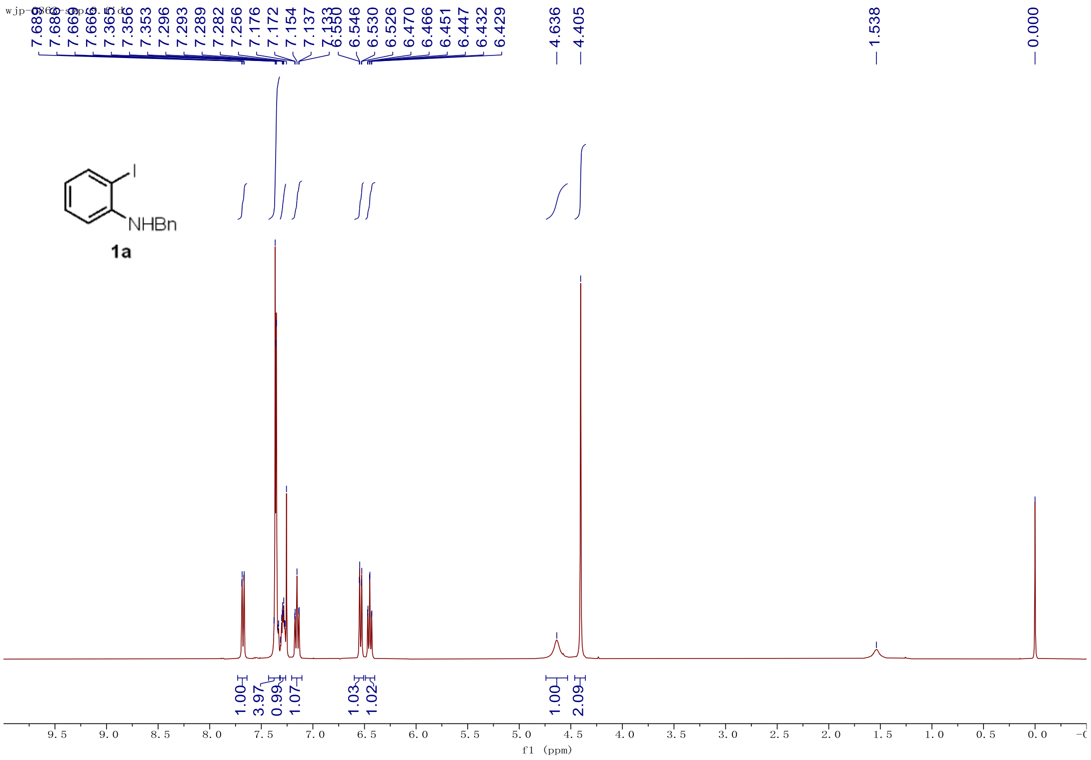

Supplementary Figure 5. ^{1}H NMR (400 MHz, $CDCl_3$) spectrum of 1a.

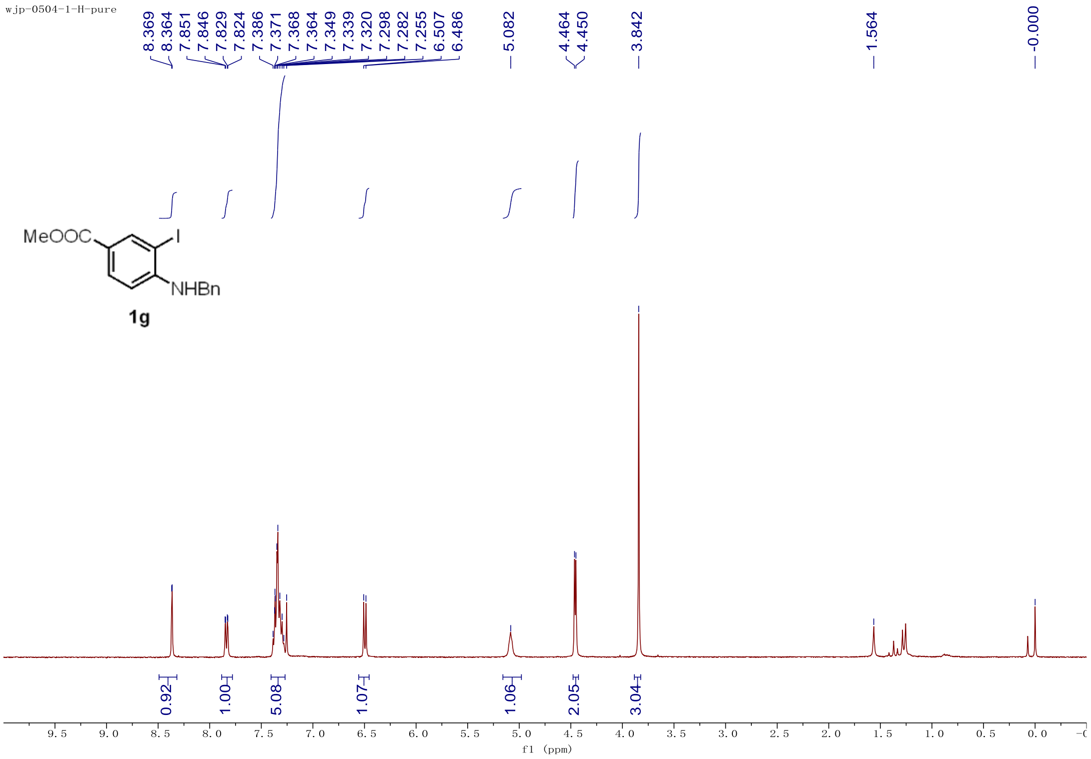

Supplementary Figure 6. ^{1}H NMR (400 MHz, $CDCl_3$) spectrum of 1g.

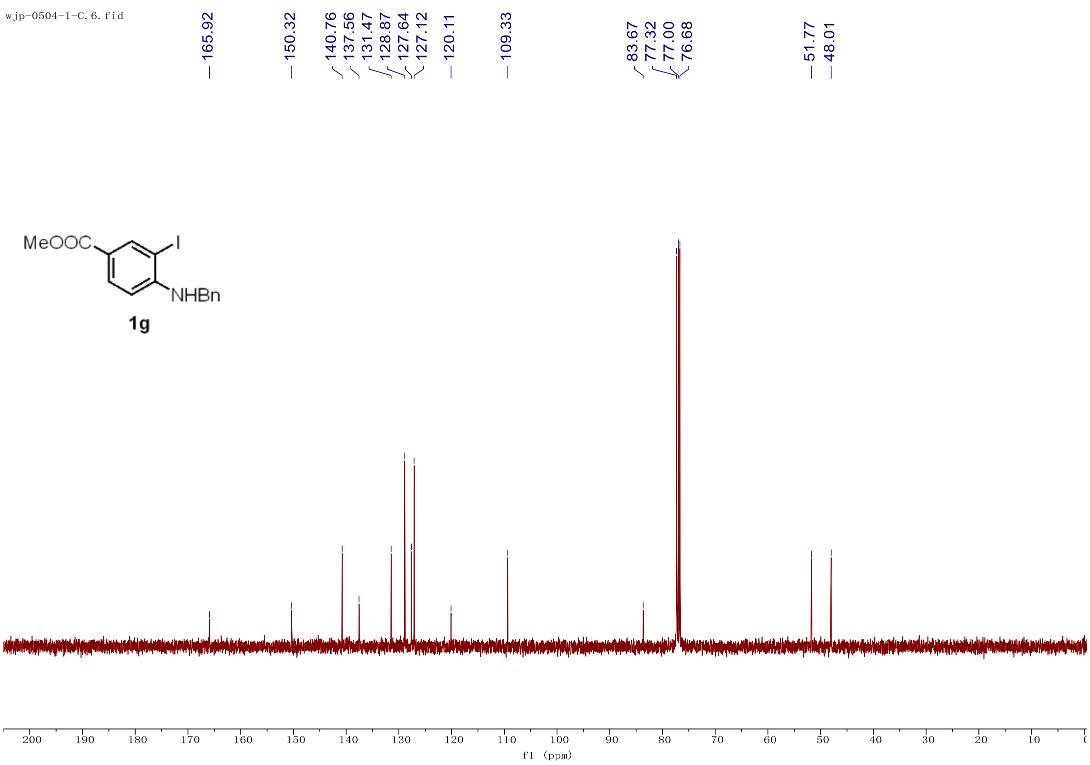

Supplementary Figure 7. ^{13}C NMR (100 MHz, $CDCl_3$) spectrum of 1g.

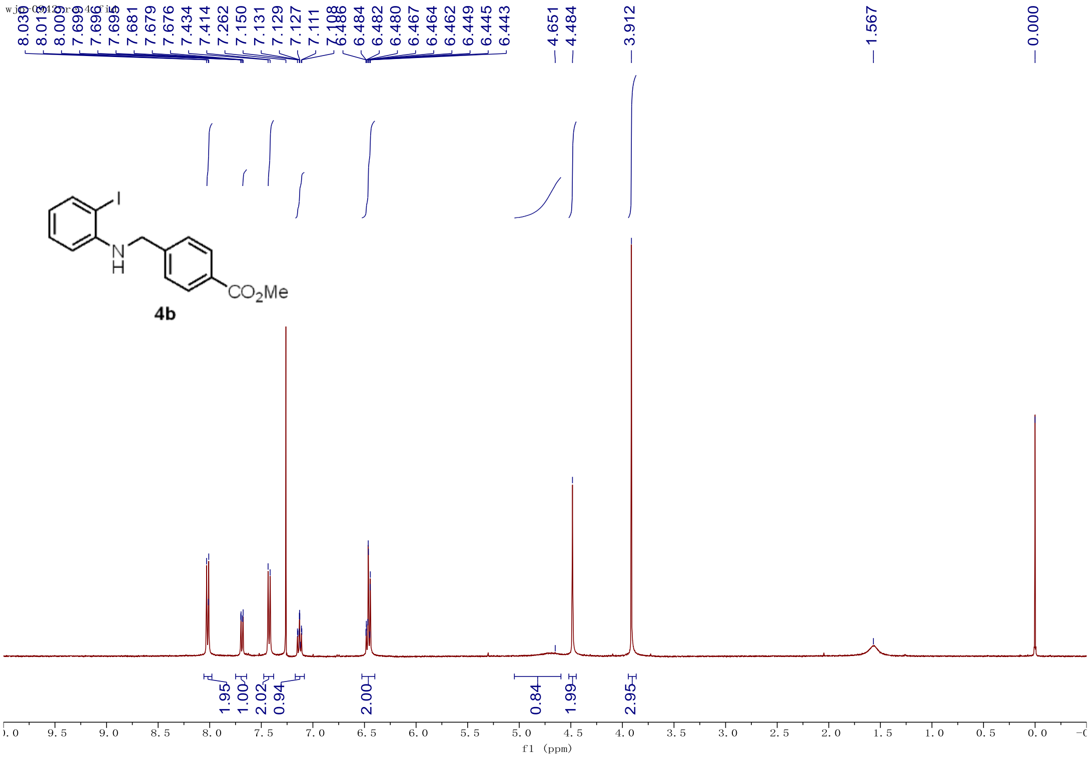

Supplementary Figure 8. ^{1}H NMR (400 MHz, $CDCl_3$) spectrum of 4b

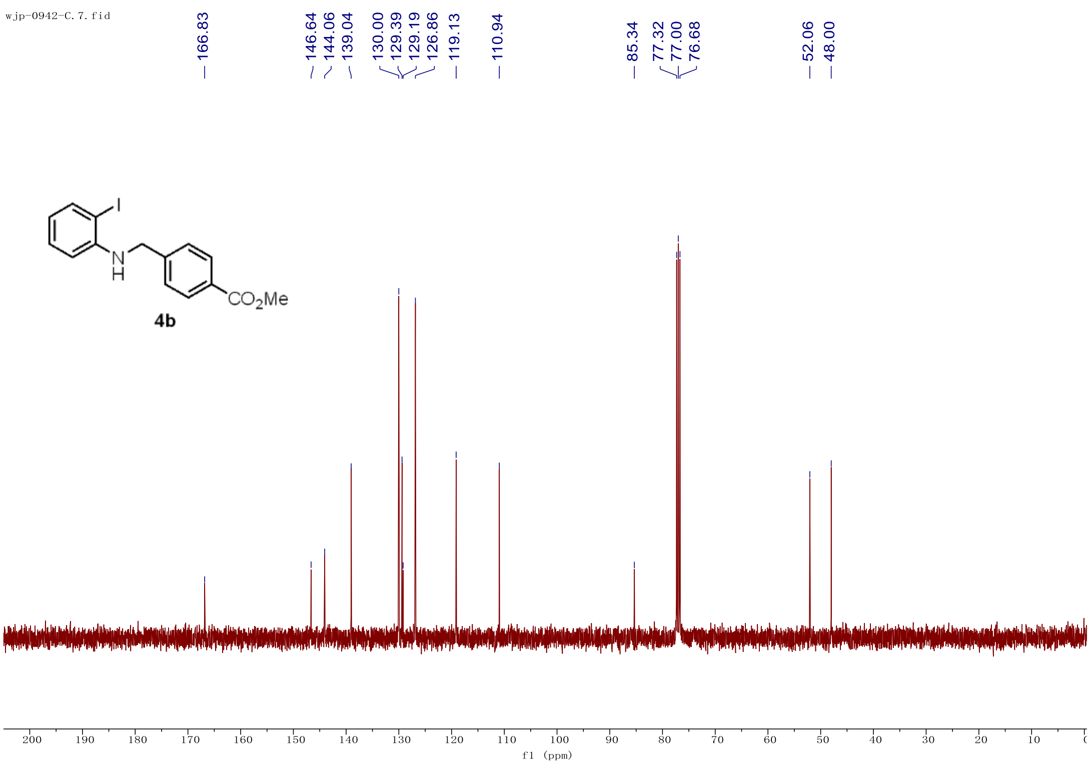

Supplementary Figure 9. ^{13}C NMR (100 MHz, $CDCl_3$) spectrum of 4b.

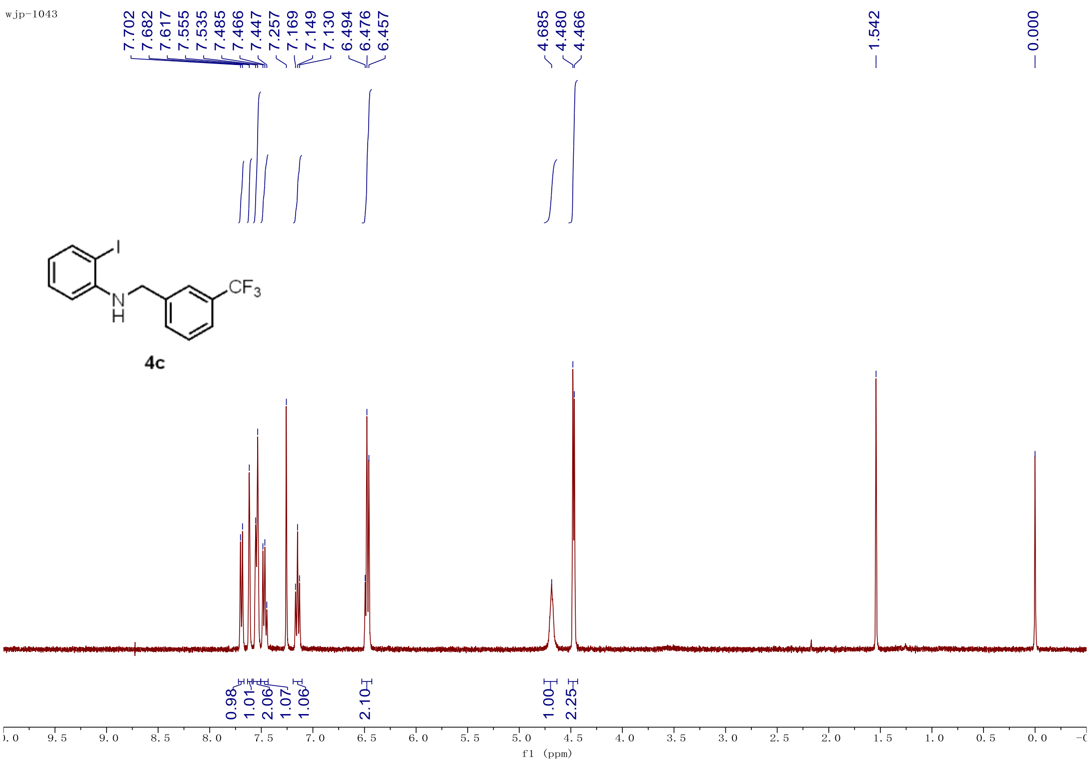

Supplementary Figure 10. ^{1}H NMR (400 MHz, $CDCl_3$) spectrum of 4c.

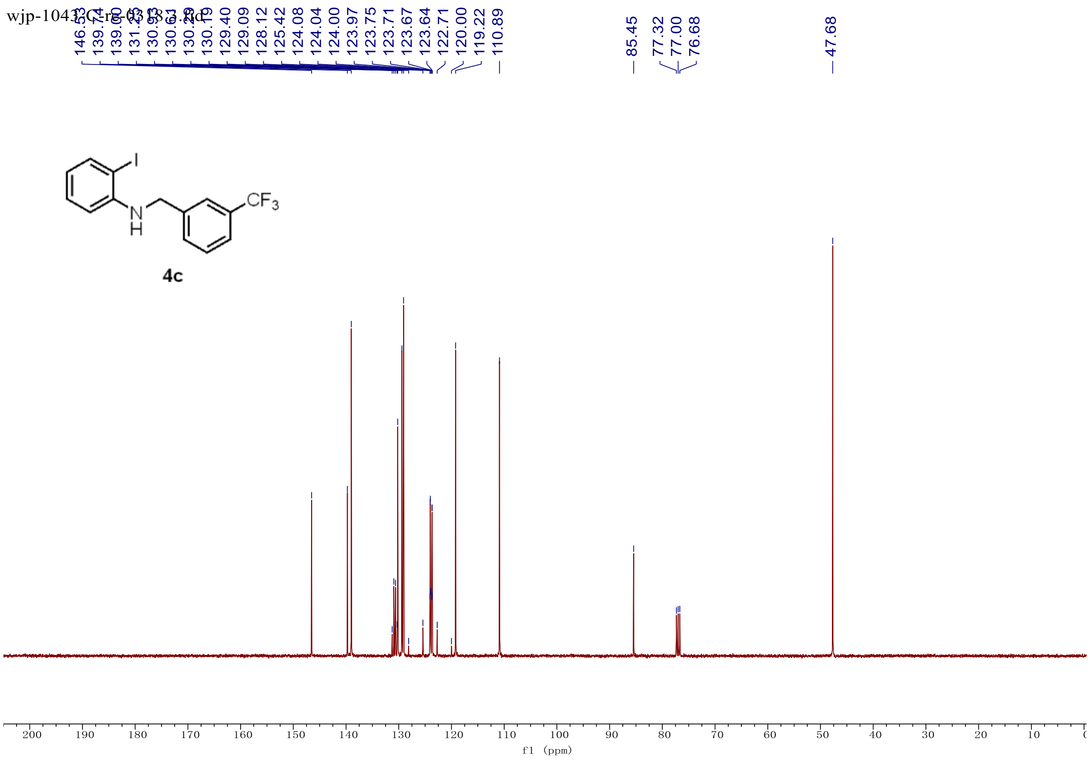

Supplementary Figure 11. ^{13}C NMR (100 MHz, $CDCl_3$) spectrum of 4c.

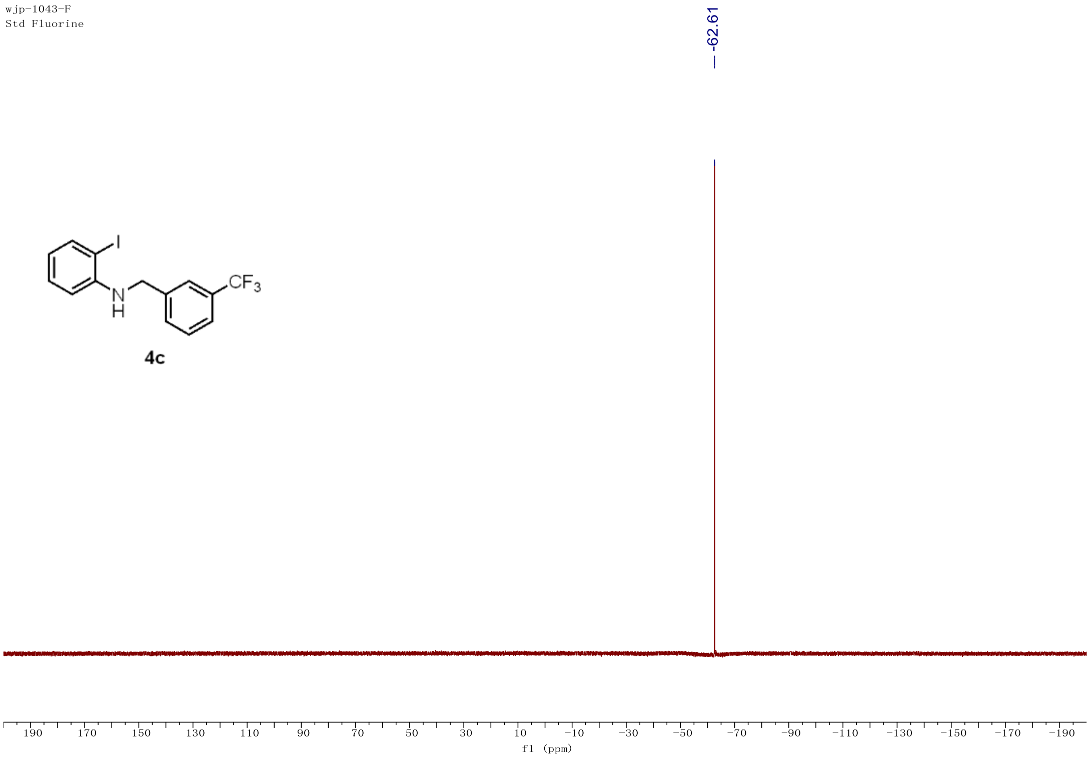

Supplementary Figure 12. ^{19}F NMR (375 MHz, $CDCl_3$) spectrum of 4c.

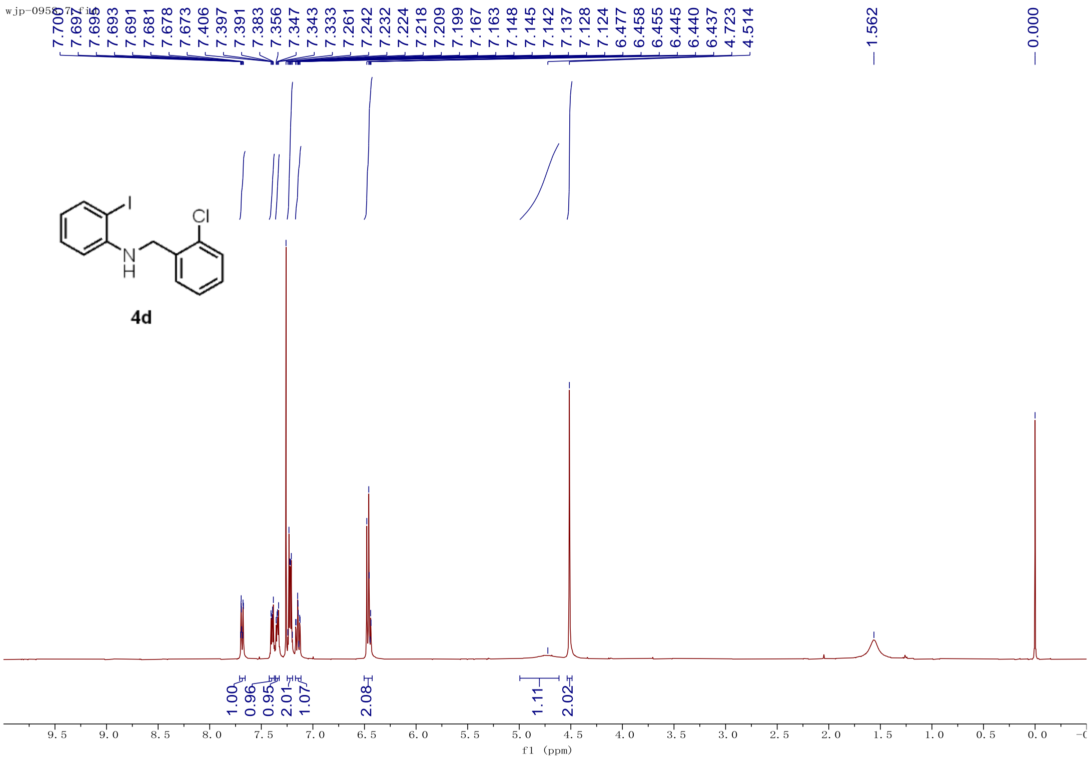

Supplementary Figure 13. ^{1}H NMR (400 MHz, $CDCl_3$) spectrum of 4d.

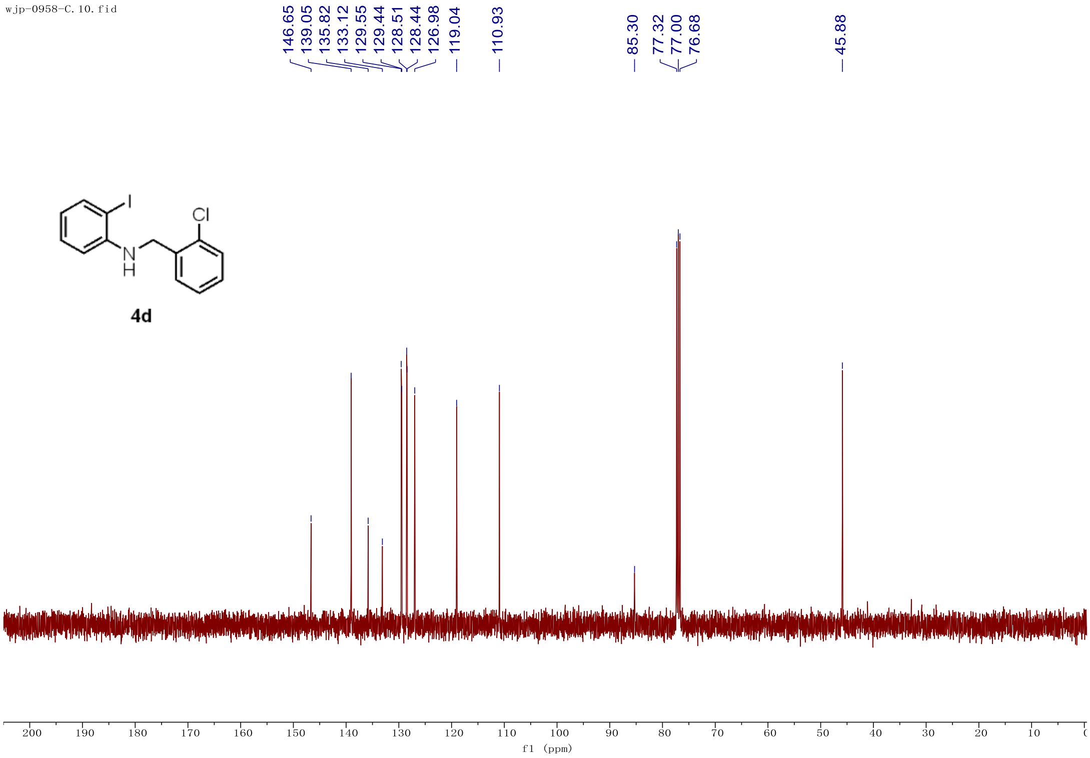

Supplementary Figure 14. ^{13}C NMR (100 MHz, $CDCl_3$) spectrum of 4d.

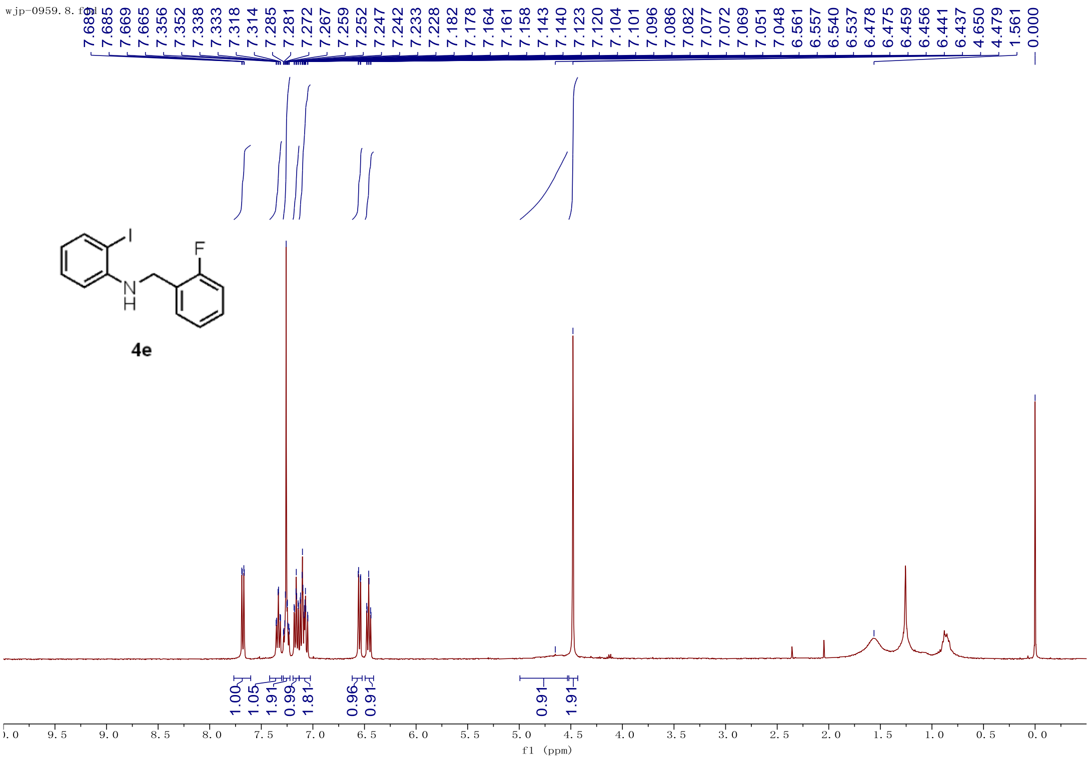

Supplementary Figure 15. ^{1}H NMR (400 MHz, $CDCl_3$) spectrum of 4e.

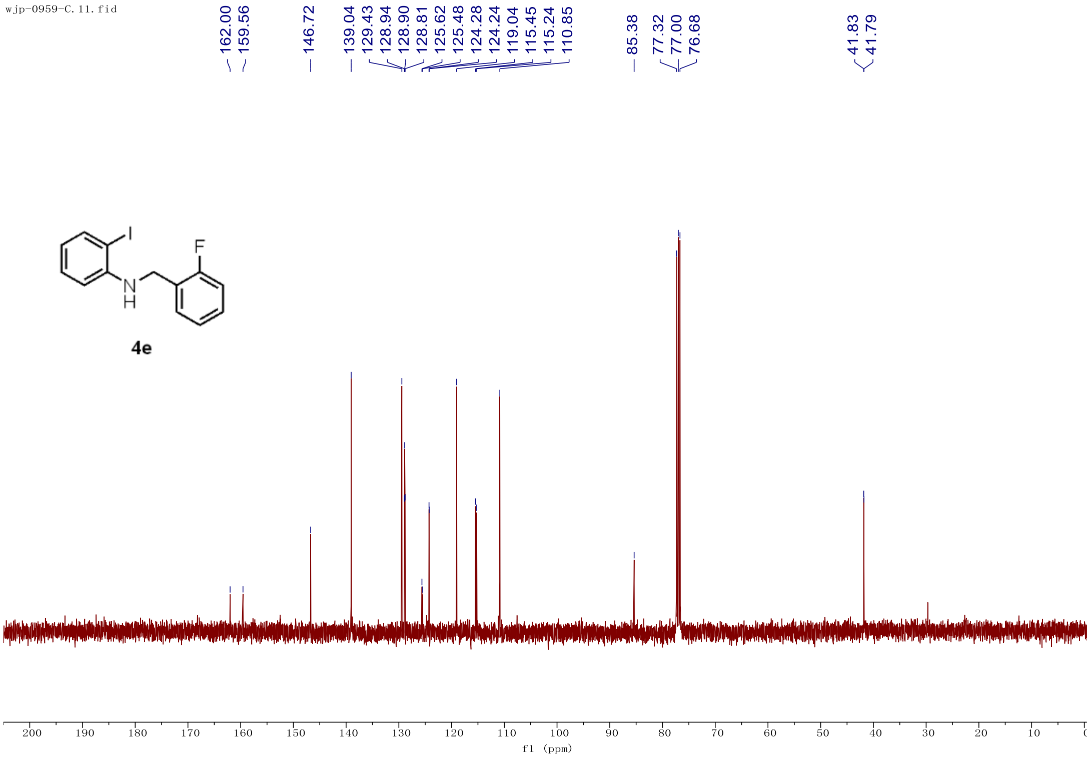

Supplementary Figure 16. ^{13}C NMR (100 MHz, $CDCl_3$) spectrum of 4e.

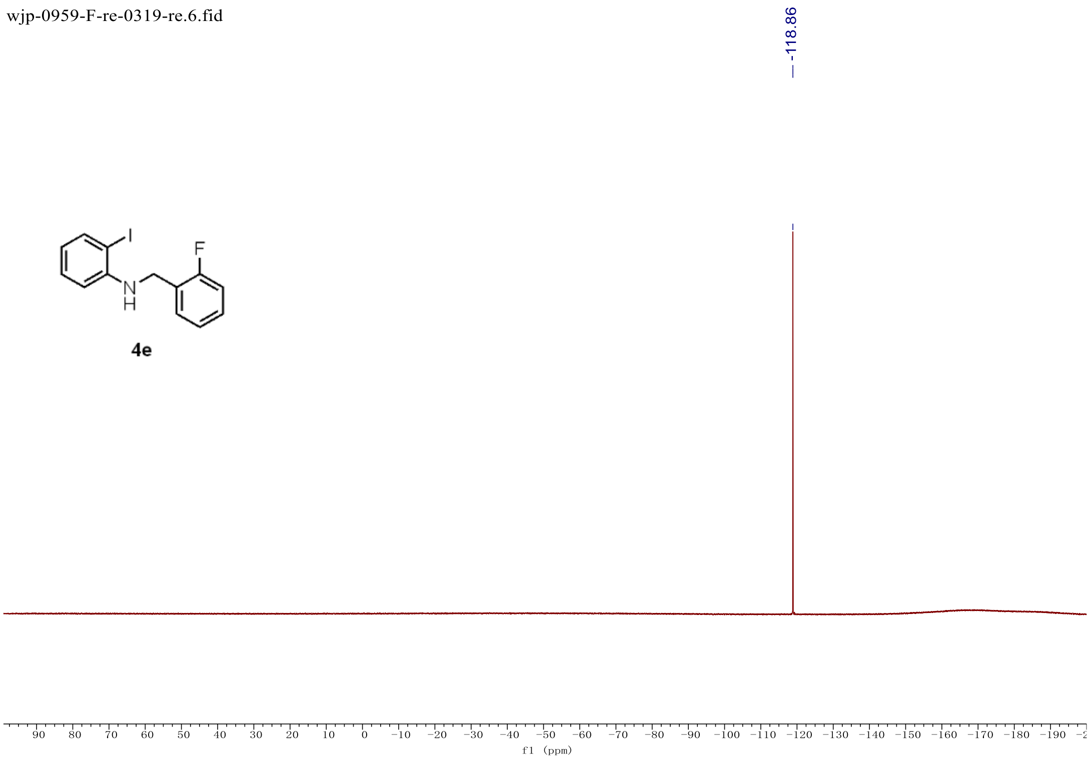

Supplementary Figure 17. ^{19}**F NMR (375 MHz,** $CDCl_3$**) spectrum of 4e.**

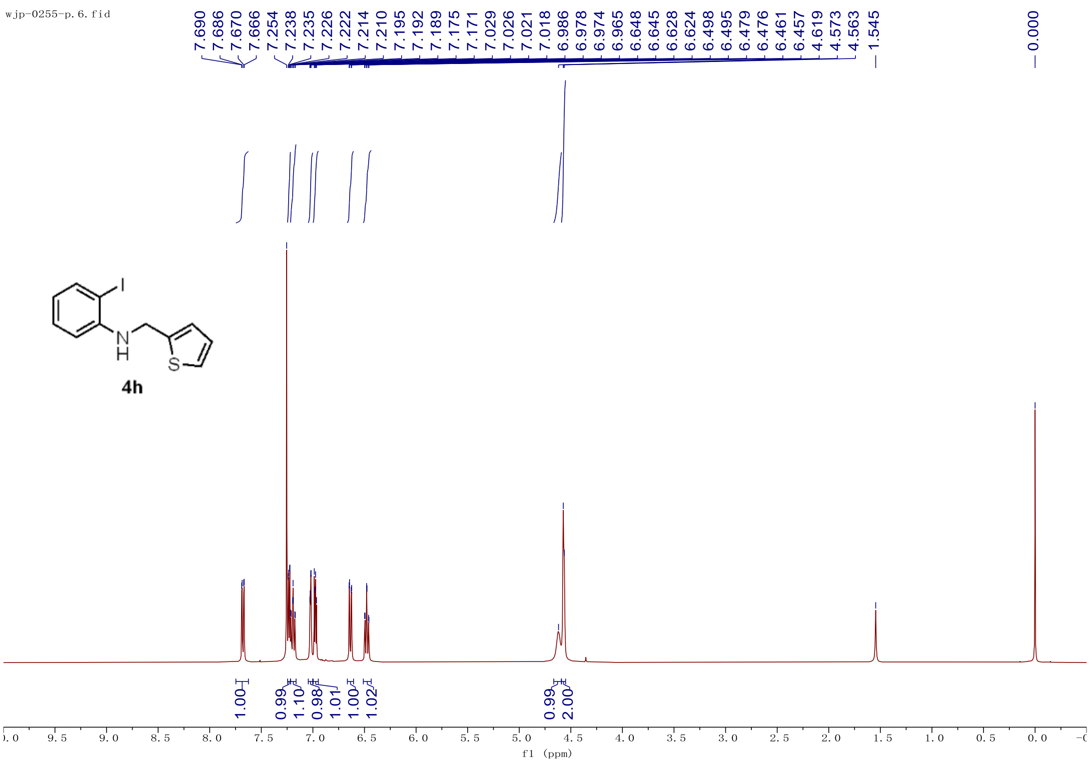

Supplementary Figure 18. ^{1}**H NMR (400 MHz,** $CDCl_3$**) spectrum of 4h.**

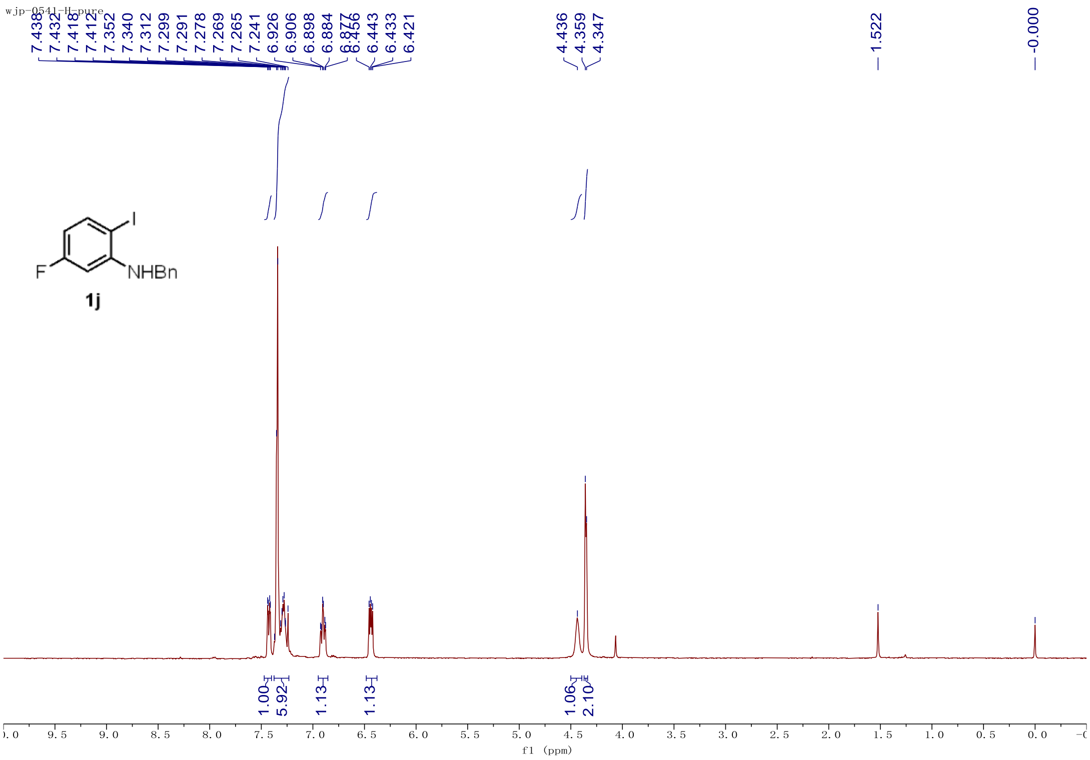

Supplementary Figure 19. ^{1}H NMR (400 MHz, $CDCl_3$) spectrum of 1j.

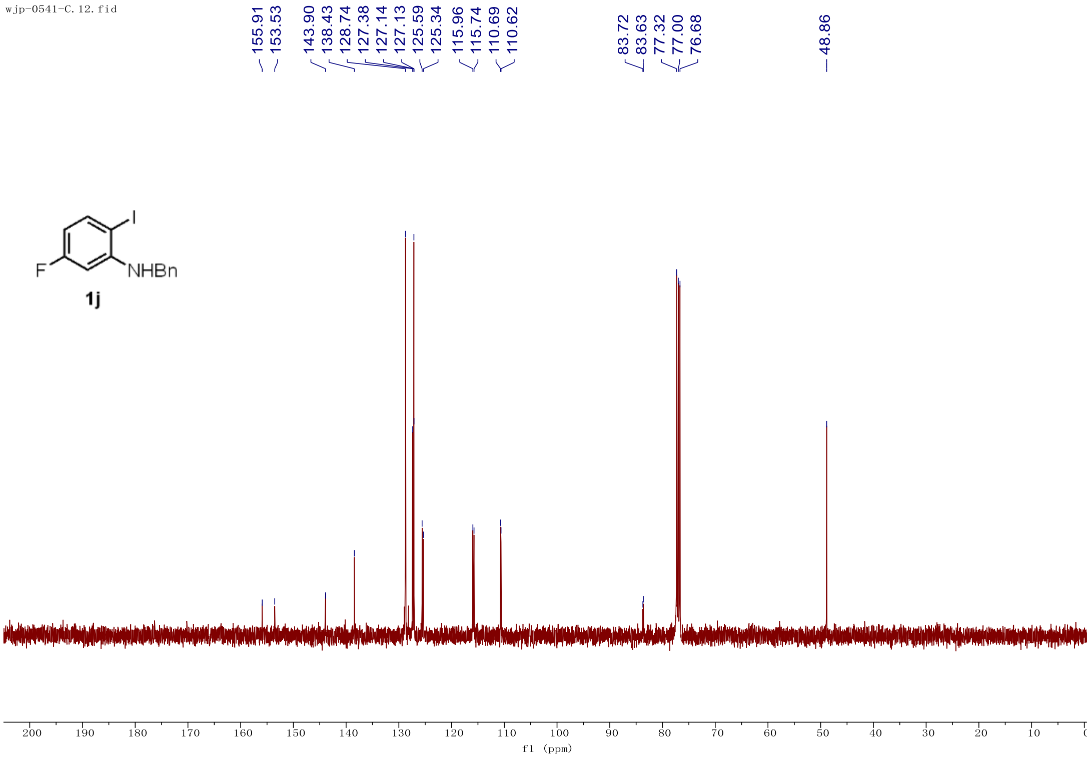

Supplementary Figure 20. ^{13}C NMR (100 MHz, $CDCl_3$) spectrum of 1j.

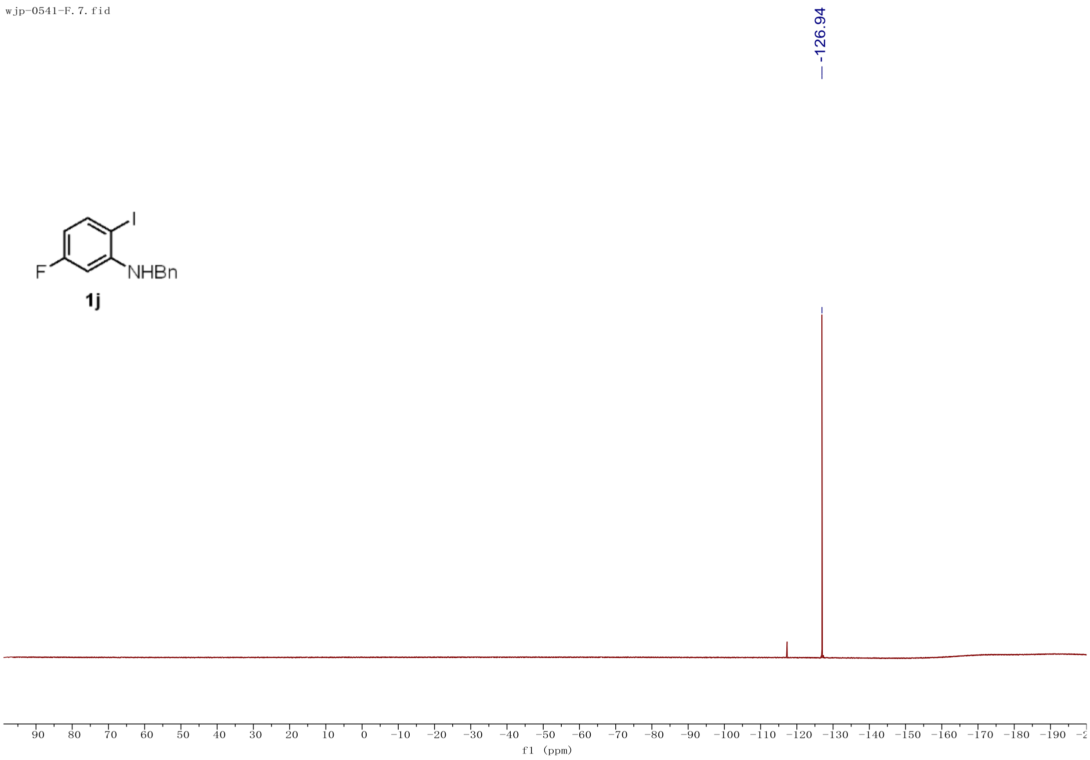

Supplementary Figure 21. ^{19}F NMR (375 MHz, $CDCl_3$) spectrum of 1j.

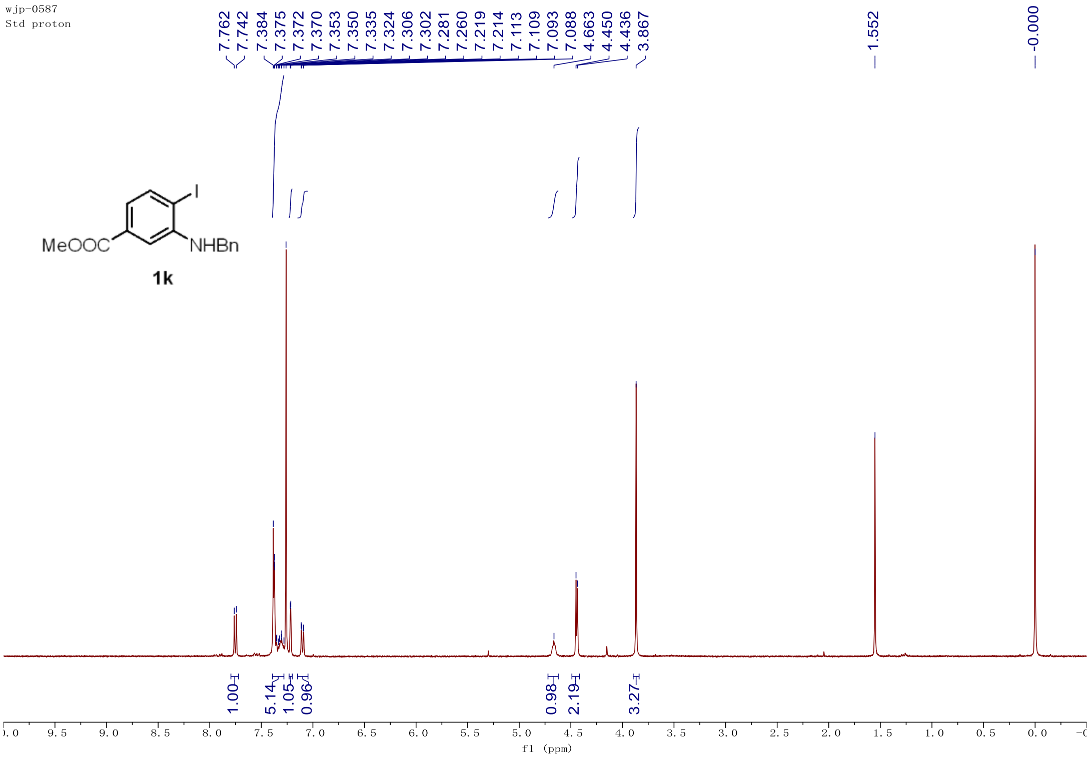

Supplementary Figure 22. ^{1}H NMR (400 MHz, $CDCl_3$) spectrum of 1k.

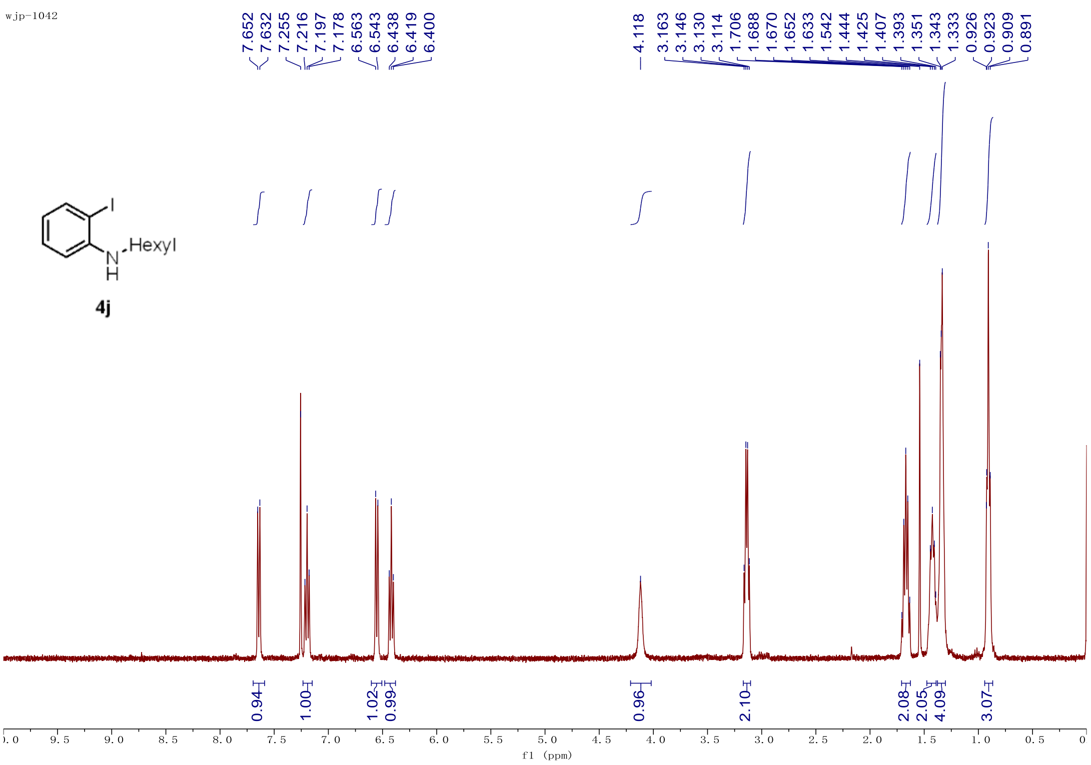

Supplementary Figure 23. ^{1}H NMR (400 MHz, $CDCl_3$) spectrum of 4j.

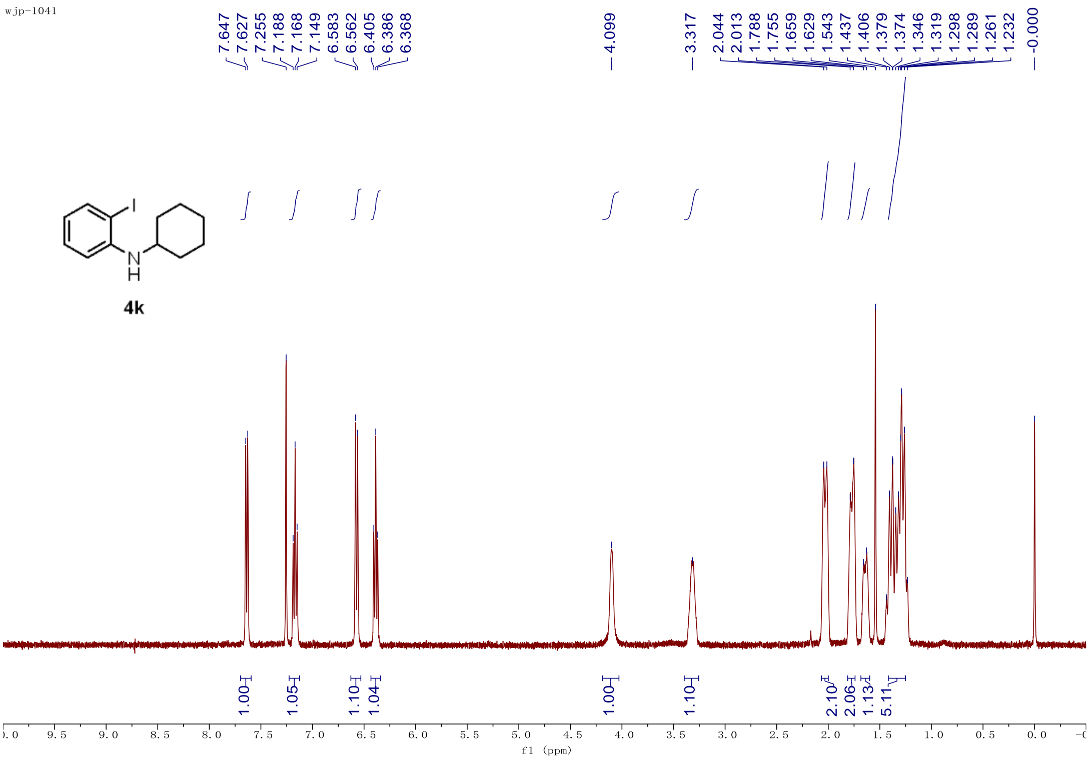

Supplementary Figure 24. ^{1}H NMR (400 MHz, $CDCl_3$) spectrum of 4k.

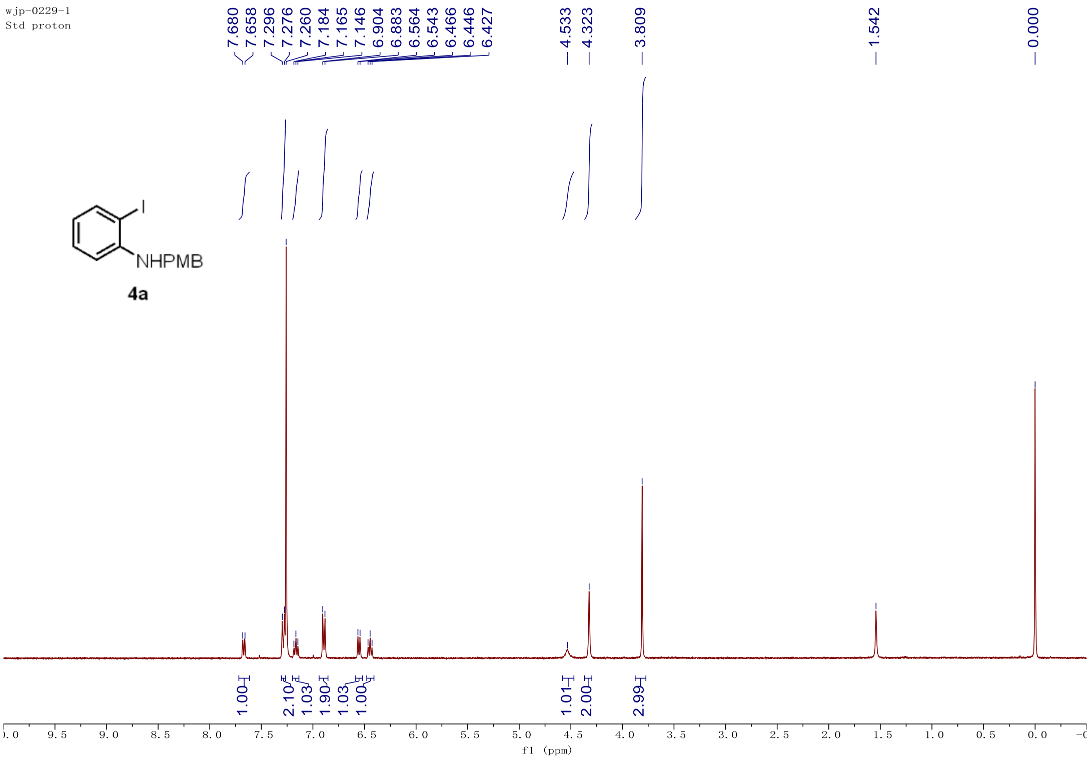

Supplementary Figure 25. ¹H NMR (400 MHz, $CDCl_3$) spectrum of 4a.

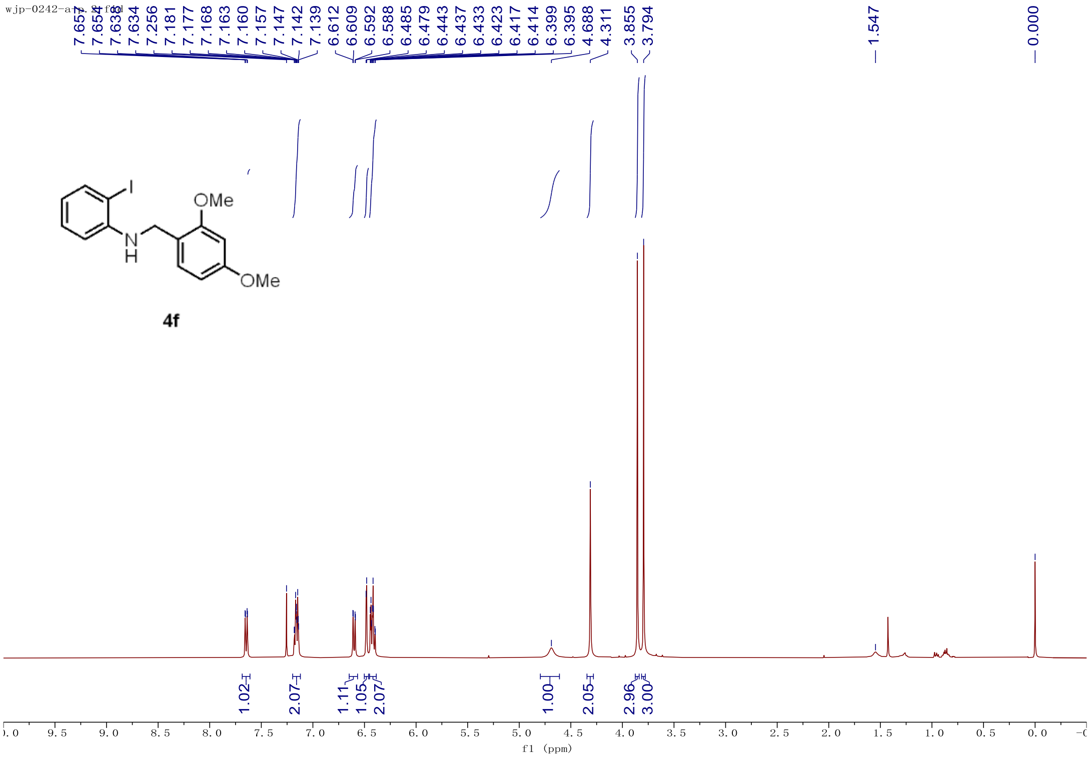

Supplementary Figure 26. ¹H NMR (400 MHz, $CDCl_3$) spectrum of 4f.

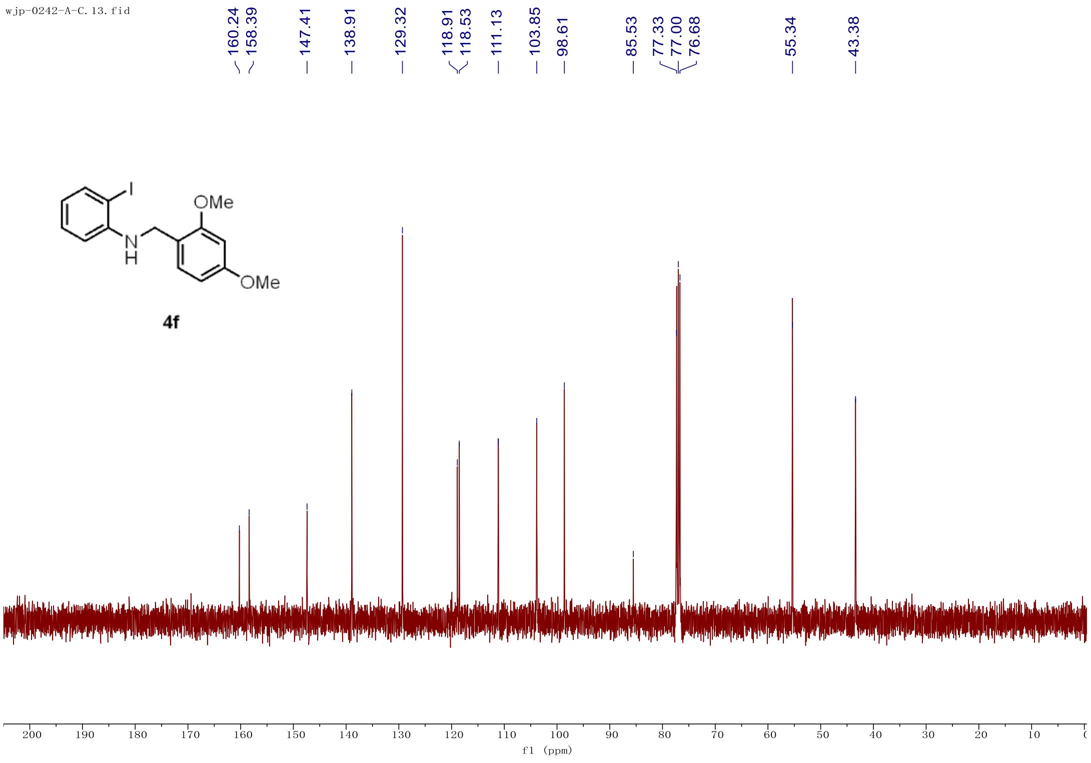

Supplementary Figure 27. ^{13}**C NMR (100 MHz,** $CDCl_3$**) spectrum of 4f.**

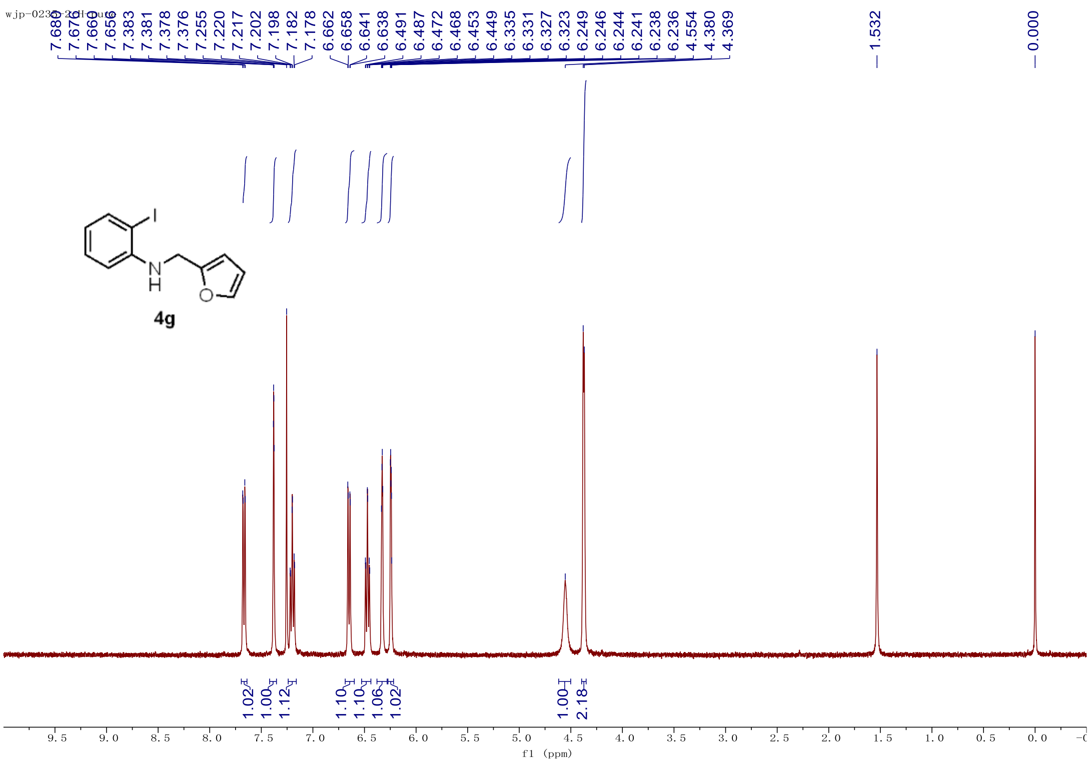

Supplementary Figure 28. ^{1}**H NMR (400 MHz,** $CDCl_3$**) spectrum of 4g.**

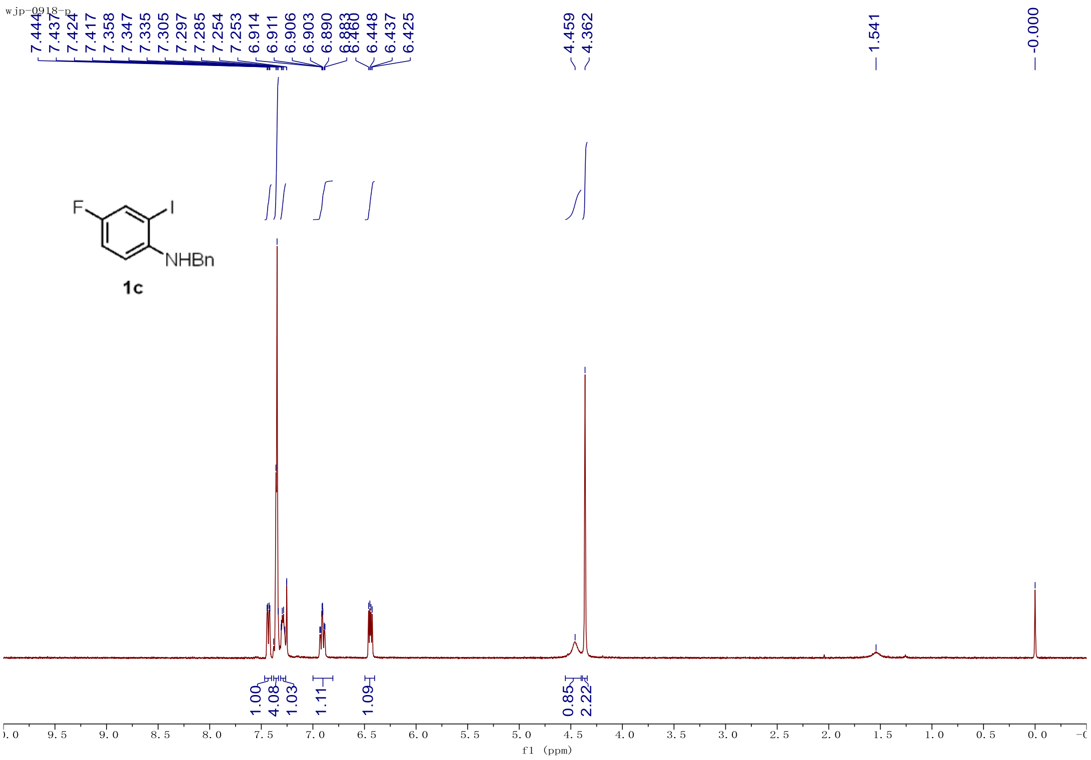

Supplementary Figure 29. ^{1}H NMR (400 MHz, CDCl$_3$) spectrum of 1c.

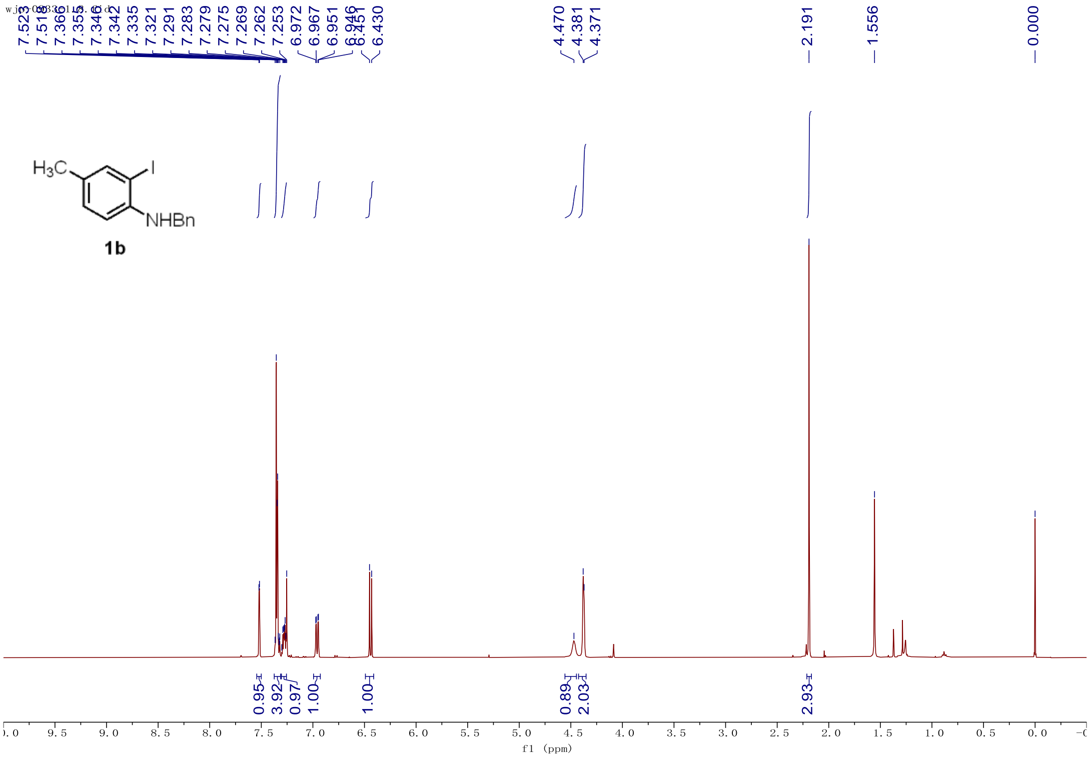

Supplementary Figure 30. ^{1}H NMR (400 MHz, CDCl$_3$) spectrum of 1b.

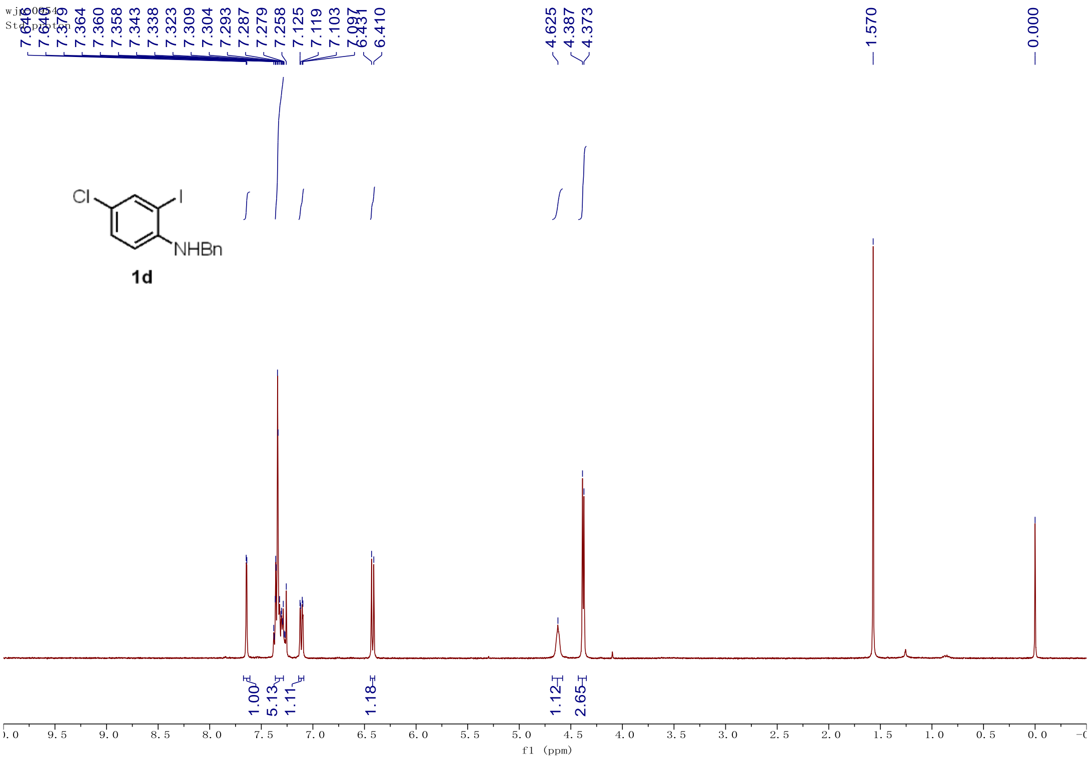

Supplementary Figure 31. ^{1}H NMR (400 MHz, $CDCl_3$) spectrum of 1d.

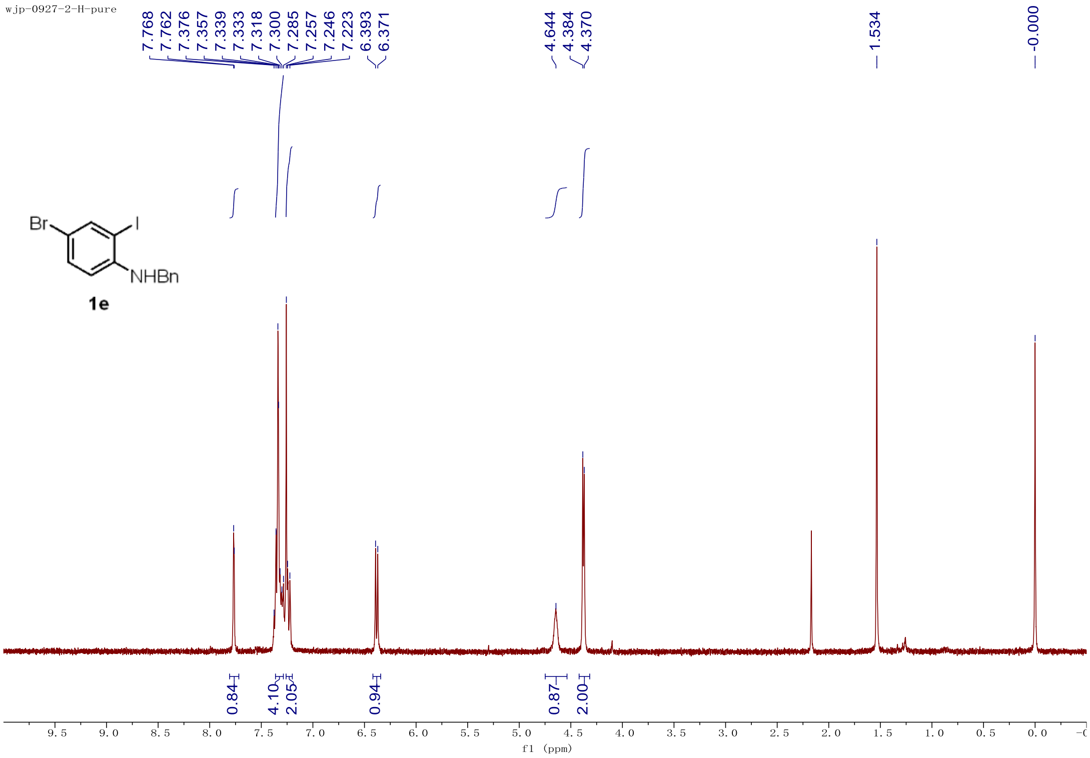

Supplementary Figure 32. ^{1}H NMR (400 MHz, $CDCl_3$) spectrum of 1e.

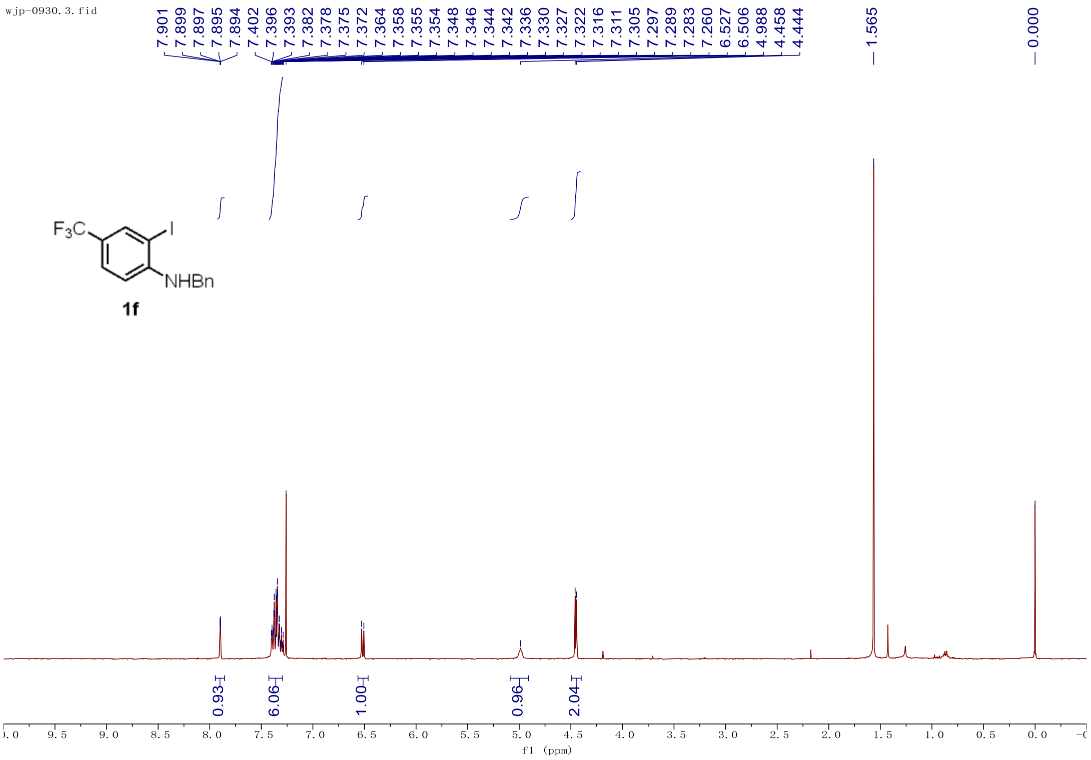

Supplementary Figure 33. ^{1}H NMR (400 MHz, $CDCl_3$) spectrum of 1f.

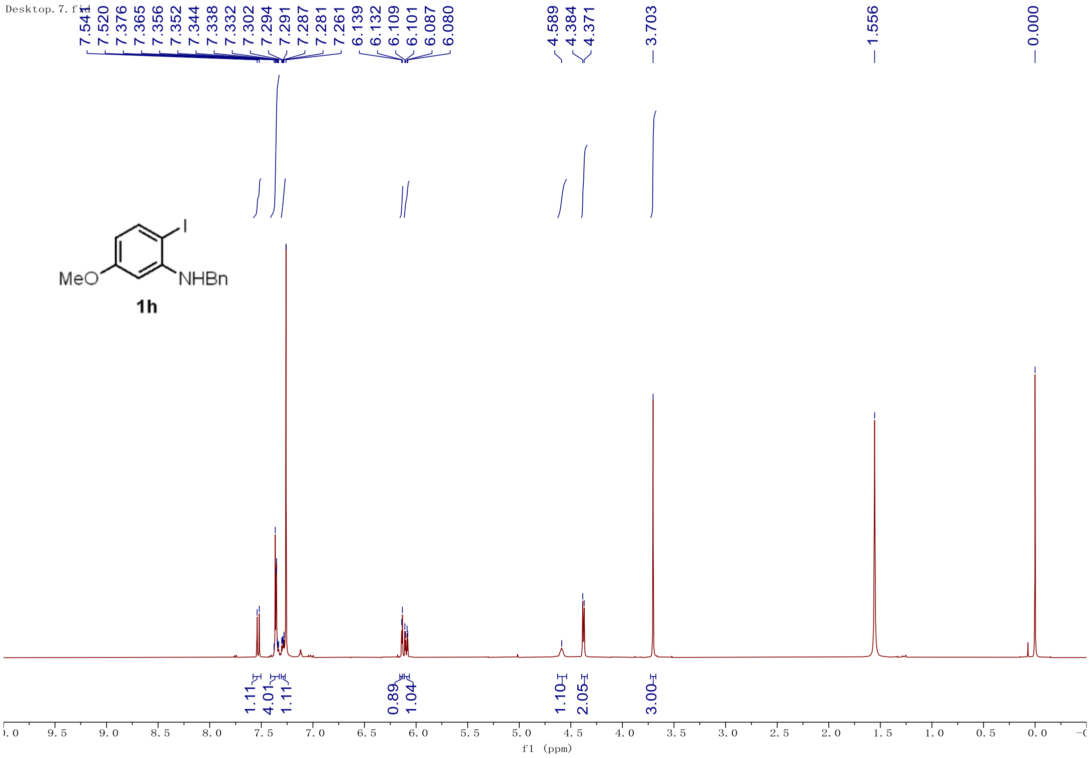

Supplementary Figure 34. ^{1}H NMR (400 MHz, $CDCl_3$) spectrum of 1h.

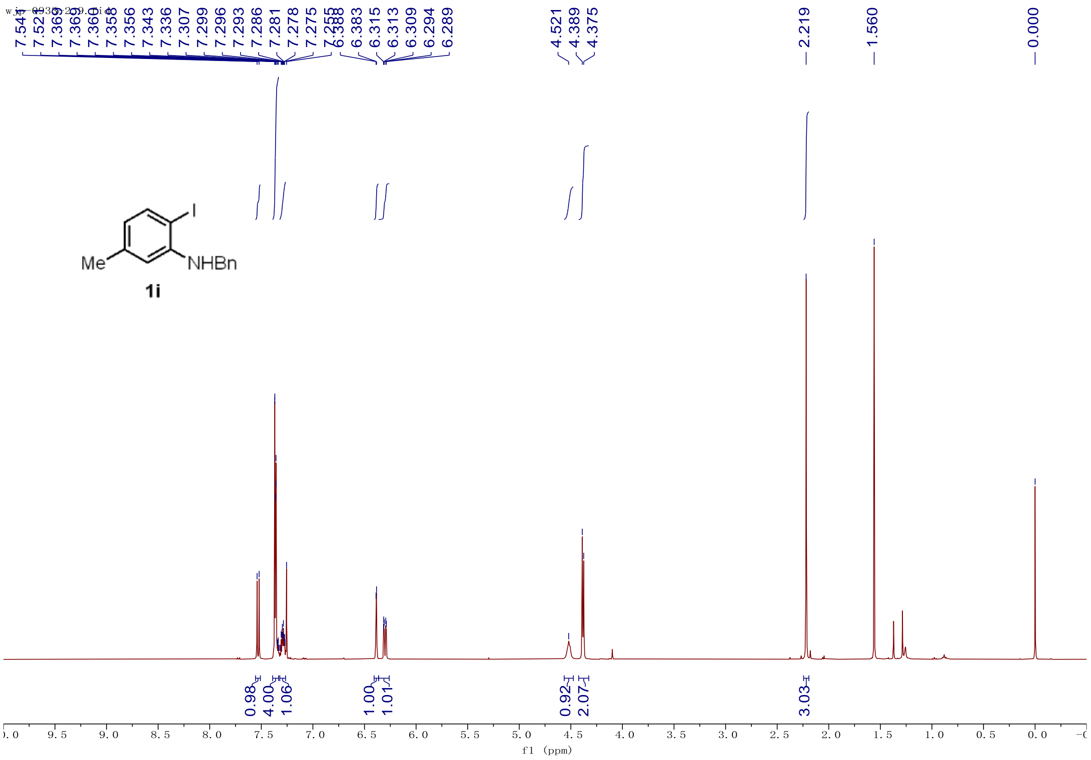

Supplementary Figure 35. ^{1}H NMR (400 MHz, $CDCl_3$) spectrum of 1i.

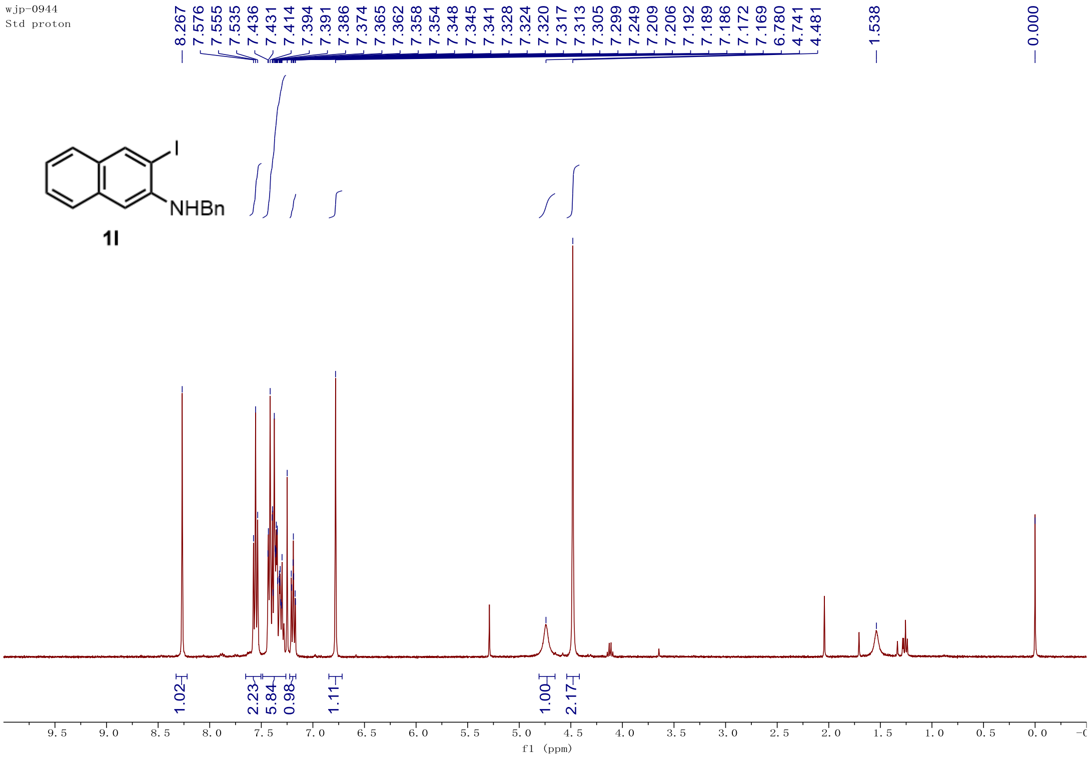

Supplementary Figure 36. ^{1}H NMR (400 MHz, $CDCl_3$) spectrum of 1l.

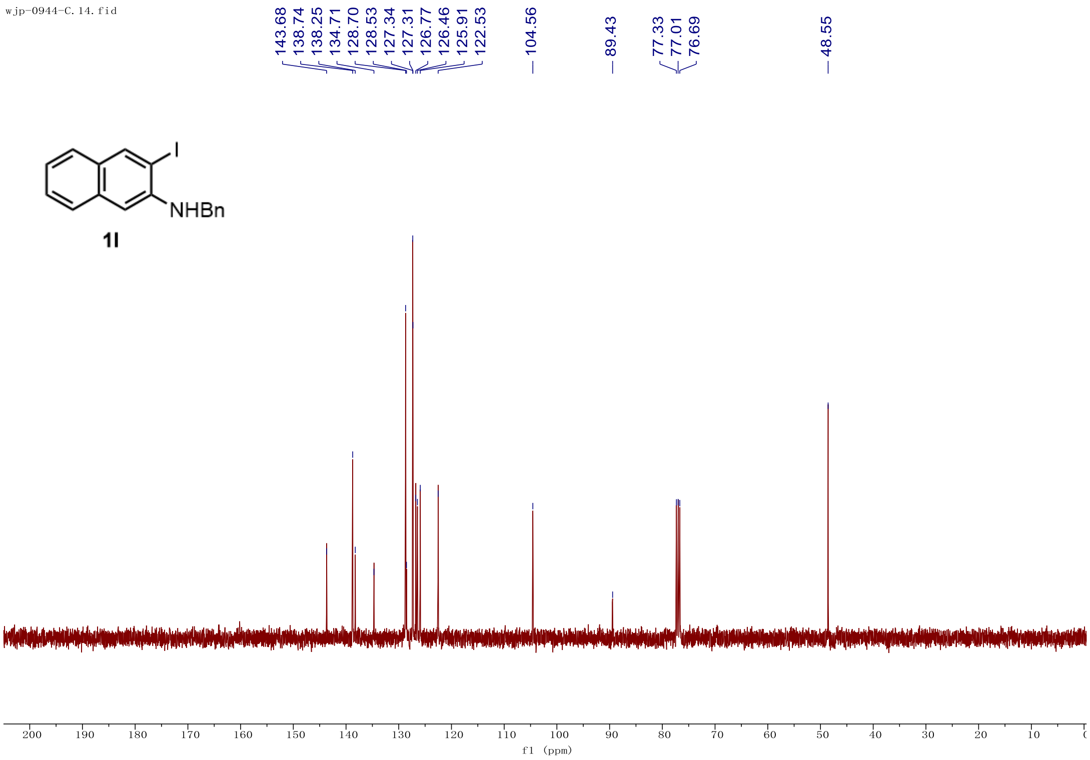

Supplementary Figure 37. **^{13}C NMR (100 MHz, $CDCl_3$) spectrum of 1l.**

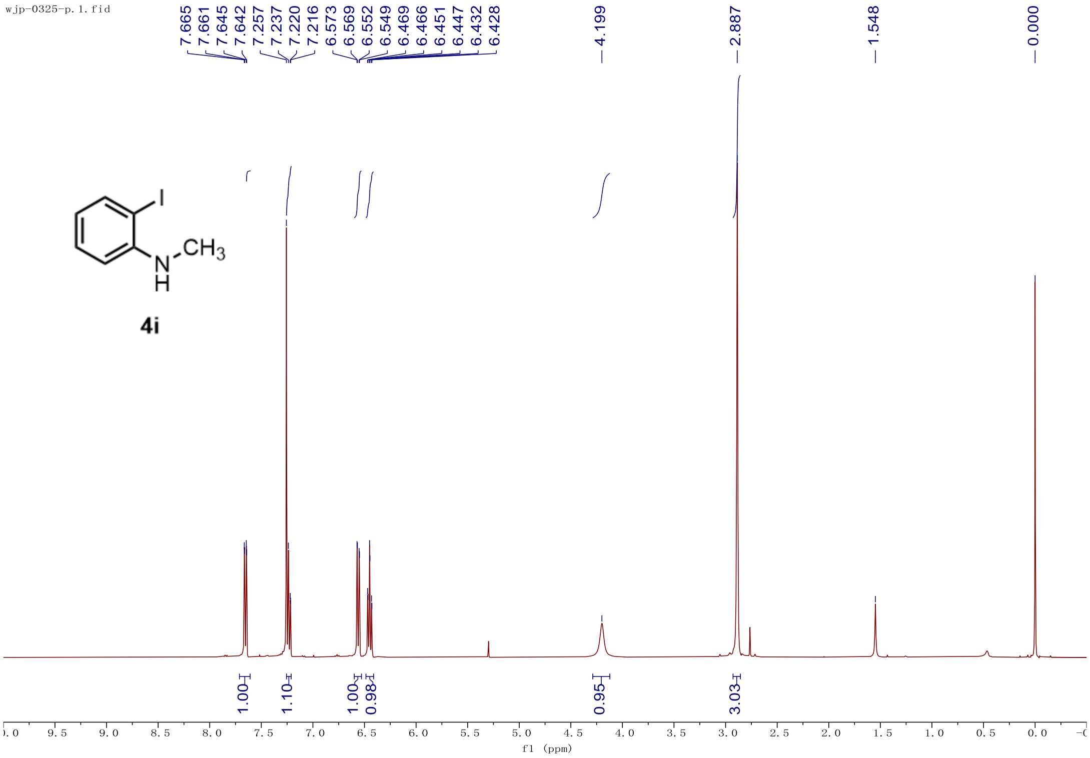

Supplementary Figure 38. **^{1}H NMR (400 MHz, $CDCl_3$) spectrum of 4i.**

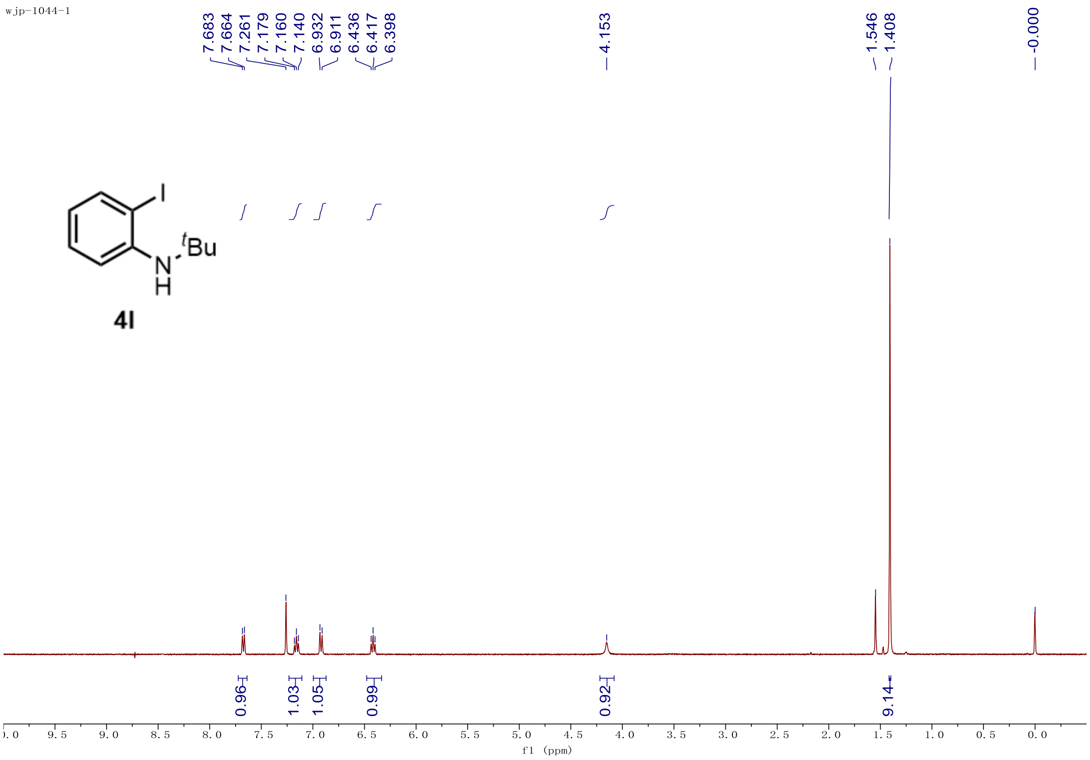

Supplementary Figure 39. ^{1}H NMR (400 MHz, $CDCl_3$) spectrum of 4l.

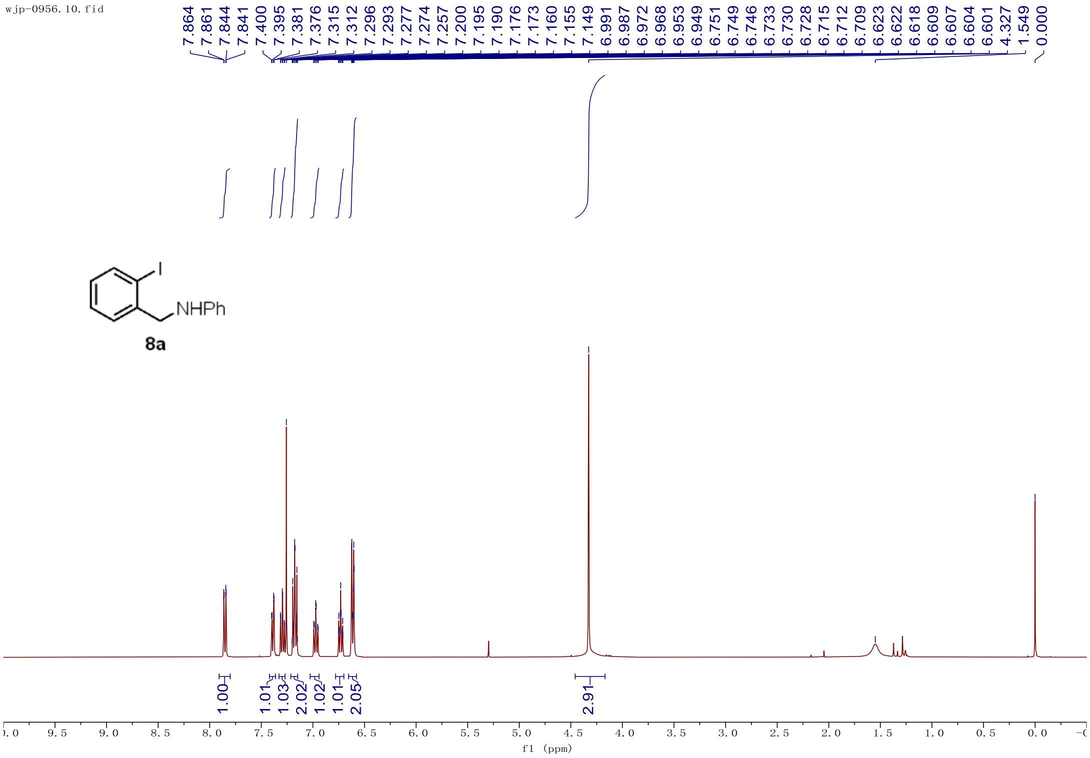

Supplementary Figure 40. ^{1}H NMR (400 MHz, $CDCl_3$) spectrum of 8a.

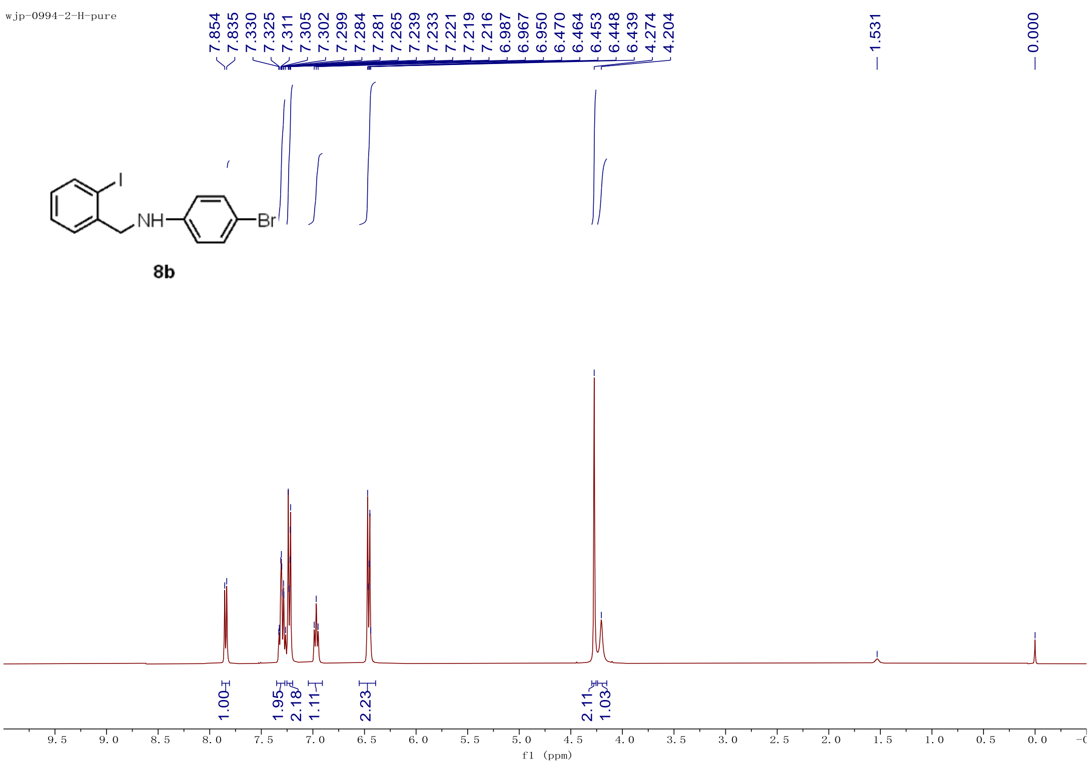

Supplementary Figure 41. ^{1}H NMR (400 MHz, $CDCl_3$) spectrum of 8b.

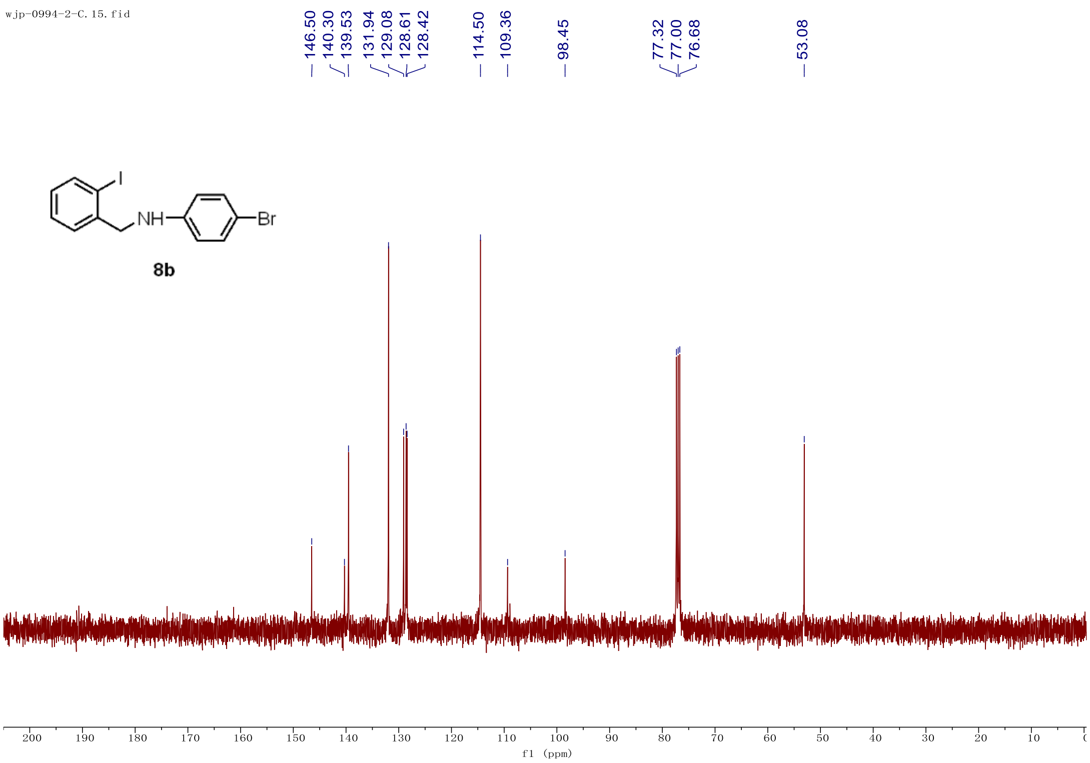

Supplementary Figure 42. ^{13}C NMR (100 MHz, $CDCl_3$) spectrum of 8b.

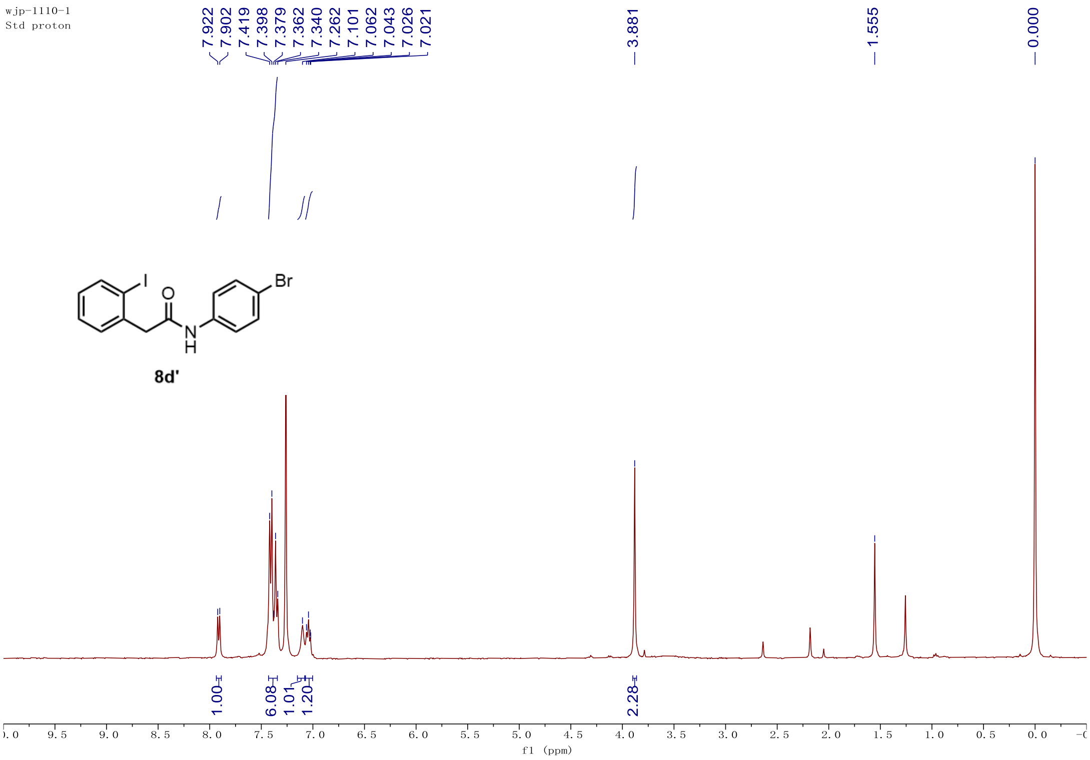

Supplementary Figure 43. ^{1}H NMR (400 MHz, $CDCl_3$) spectrum of 8d'.

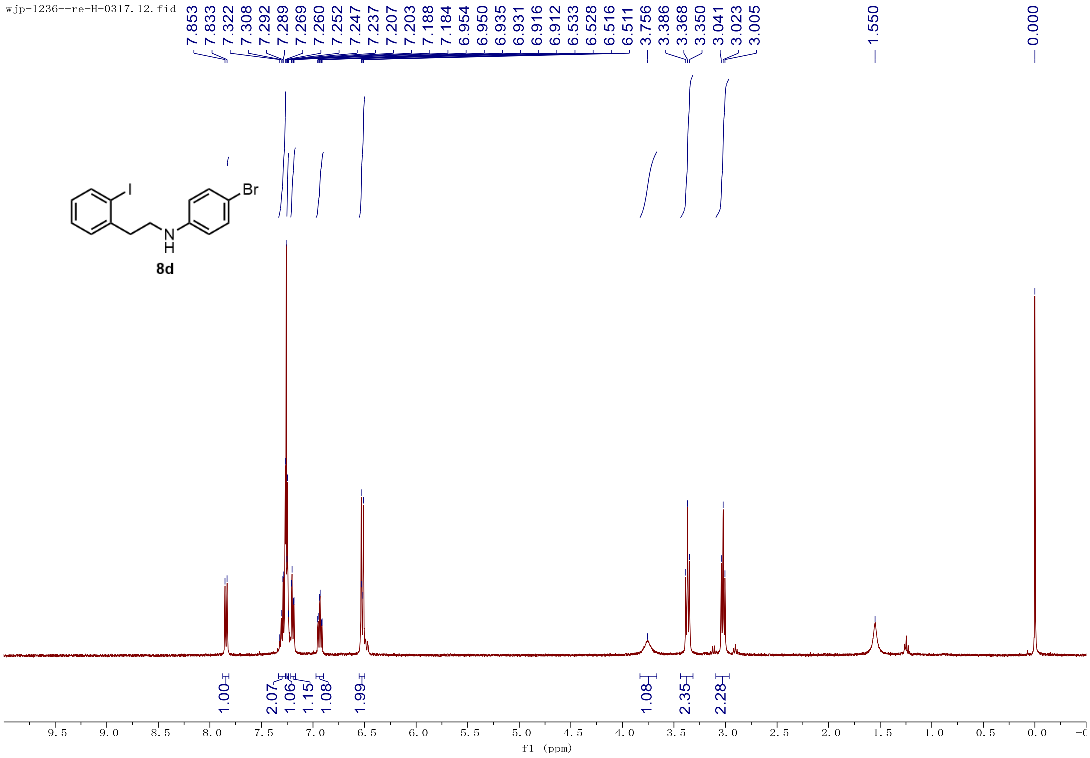

Supplementary Figure 44. ^{1}H NMR (400 MHz, $CDCl_3$) spectrum of 8d.

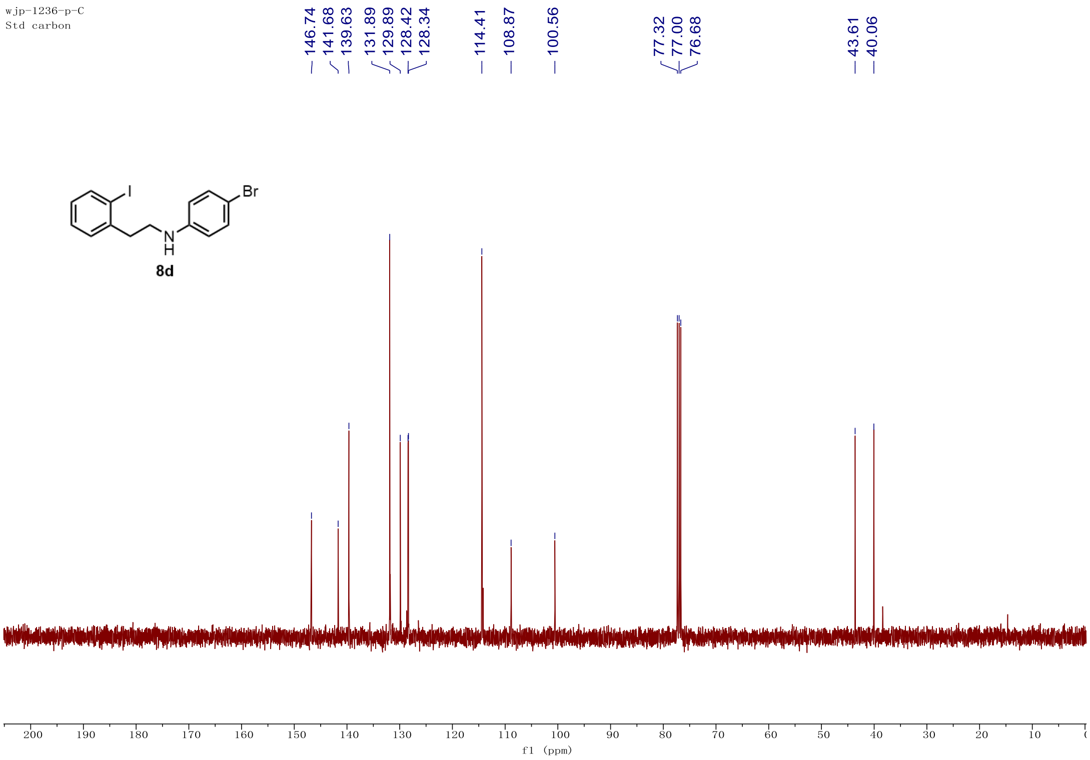

Supplementary Figure 45. ^{13}C NMR (100 MHz, $CDCl_3$) spectrum of 8d.

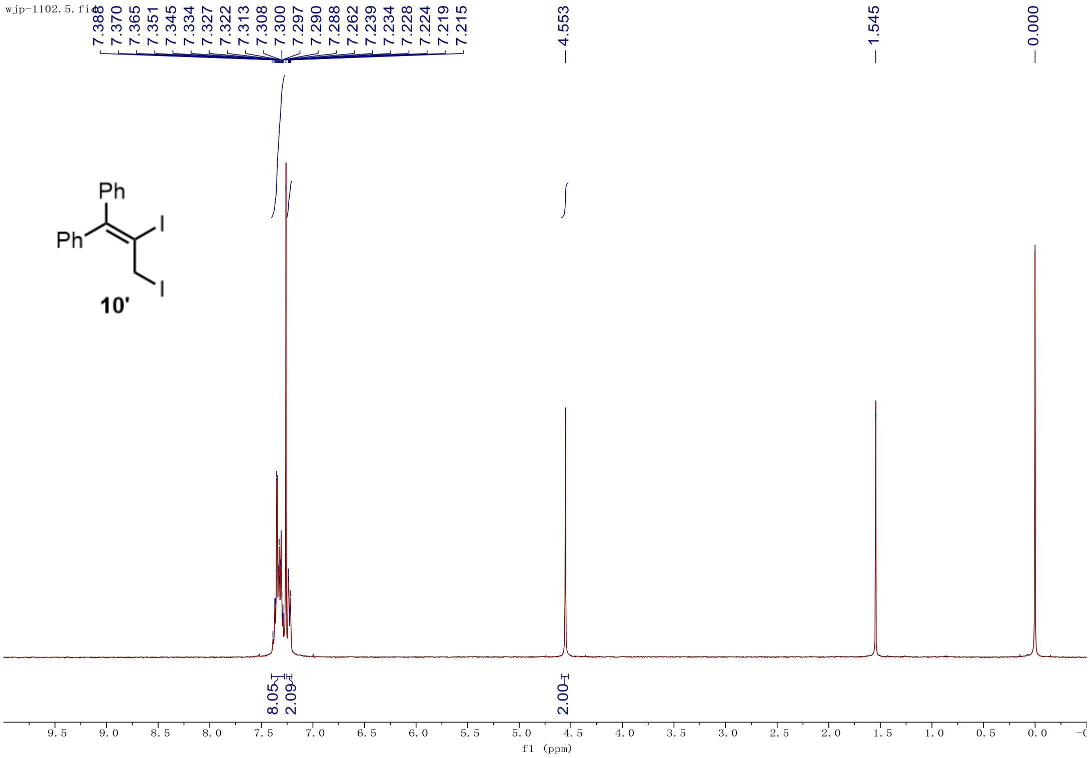

Supplementary Figure 46. ^{1}H NMR (400 MHz, $CDCl_3$) spectrum of 10'.

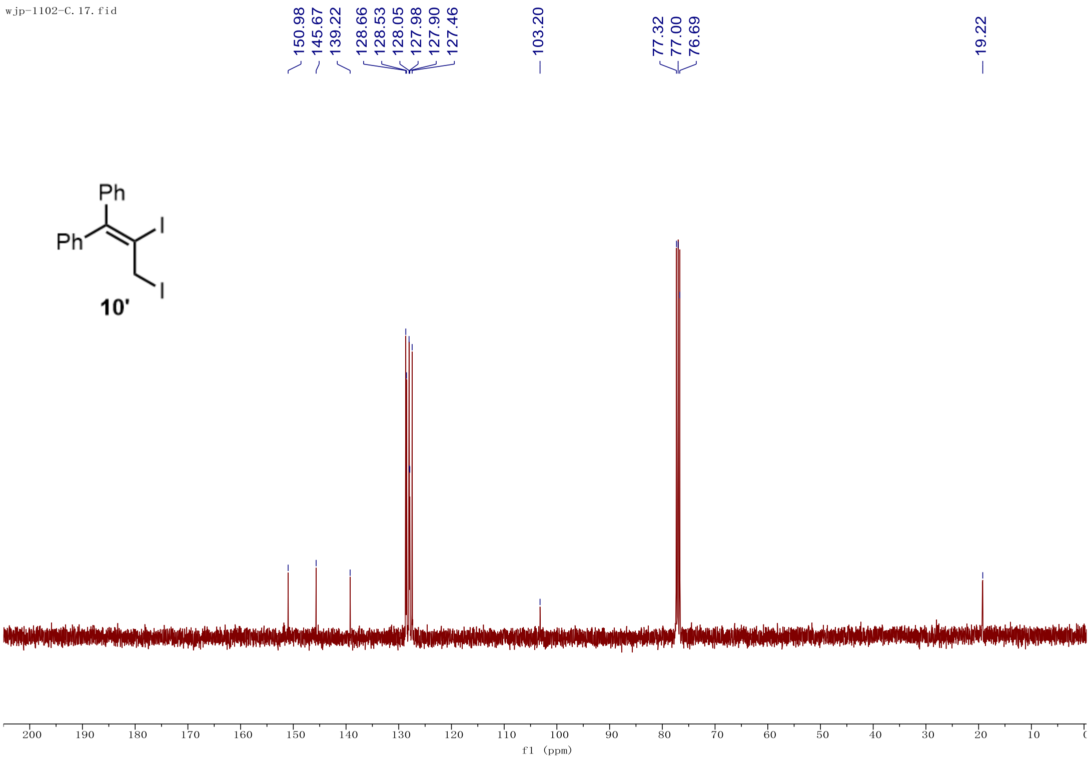

Supplementary Figure 47. ^{13}**C NMR (100 MHz, CDCl**$_3$**) spectrum of 10'.**

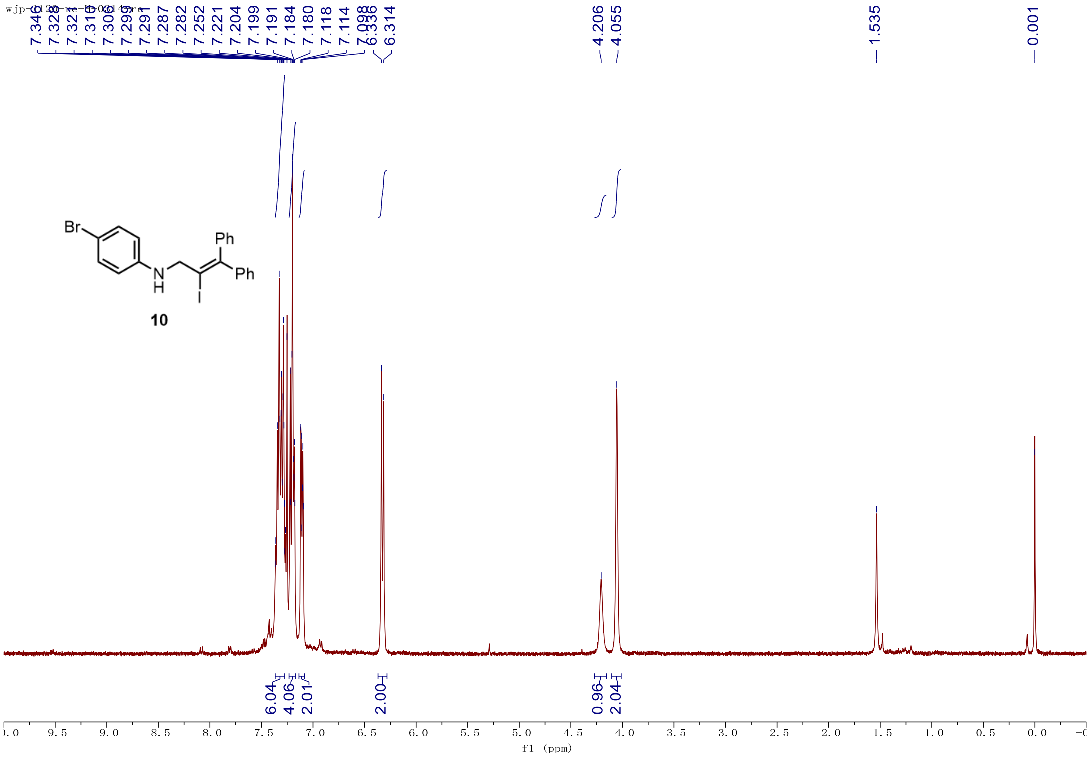

Supplementary Figure 48. ^{1}**H NMR (400 MHz, CDCl**$_3$**) spectrum of 10.**

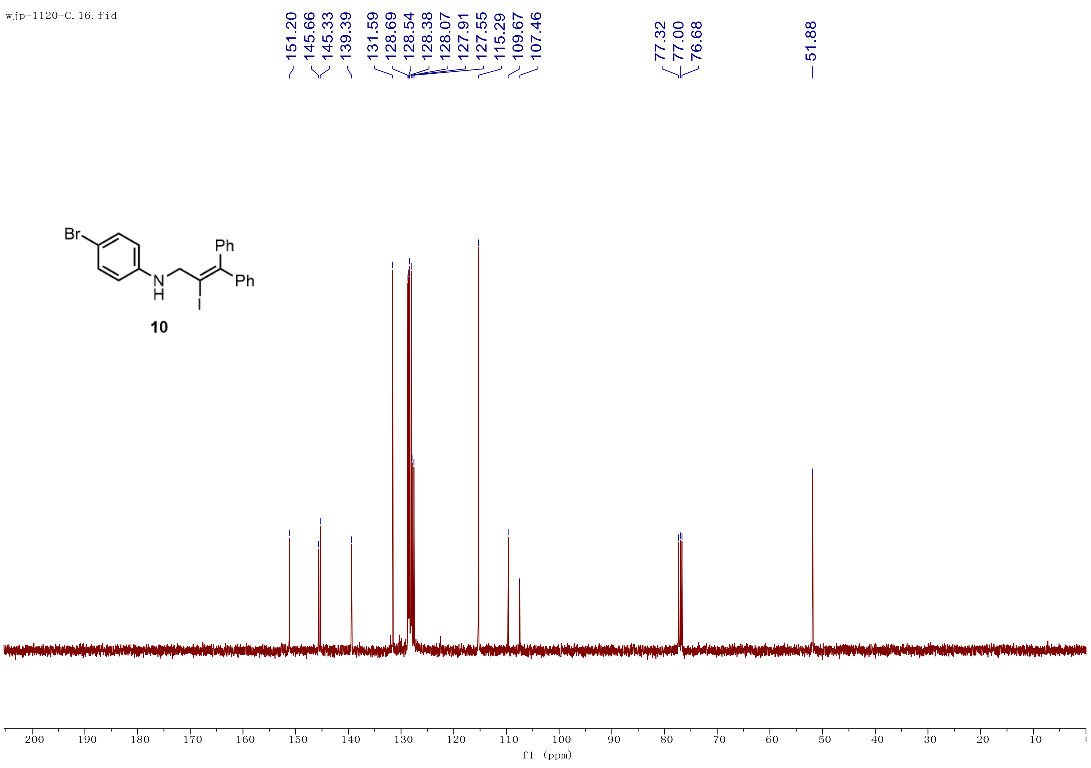

Supplementary Figure 49. ^{13}C NMR (100 MHz, $CDCl_3$) spectrum of 10.

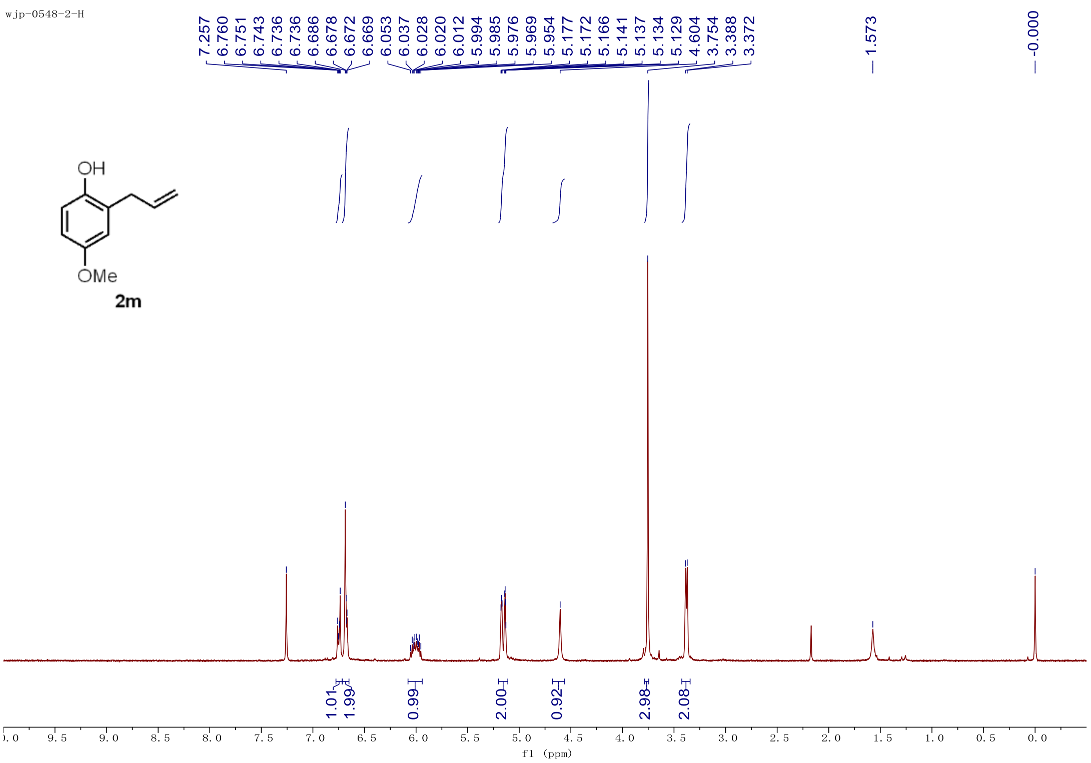

Supplementary Figure 50. ^{1}H NMR (400 MHz, $CDCl_3$) spectrum of 2m.

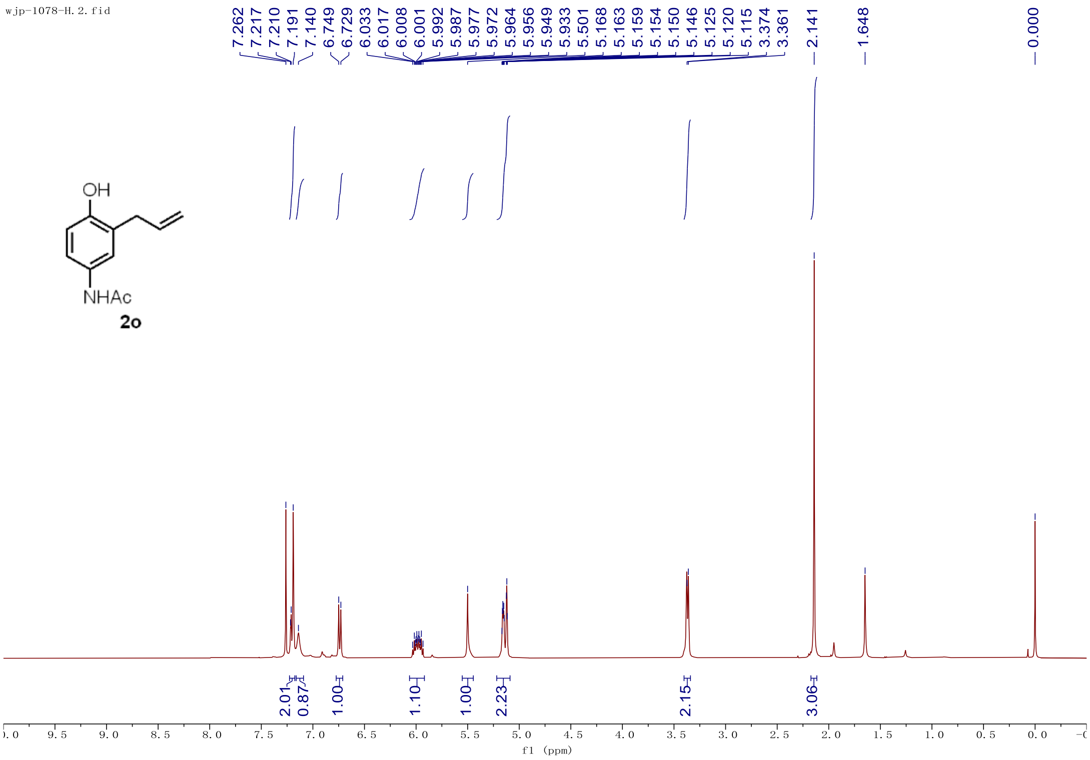

Supplementary Figure 51. ^{1}H NMR (400 MHz, CDCl$_3$) spectrum of 2o.

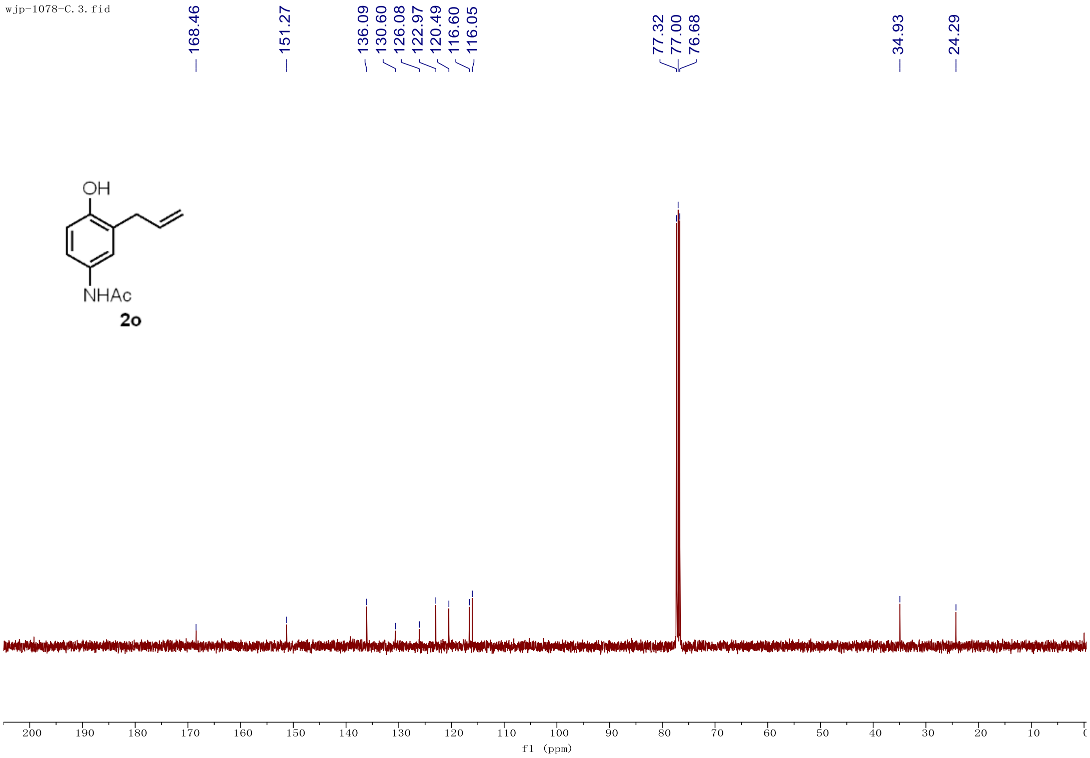

Supplementary Figure 52. ^{13}C NMR (100 MHz, CDCl$_3$) spectrum of 2o.

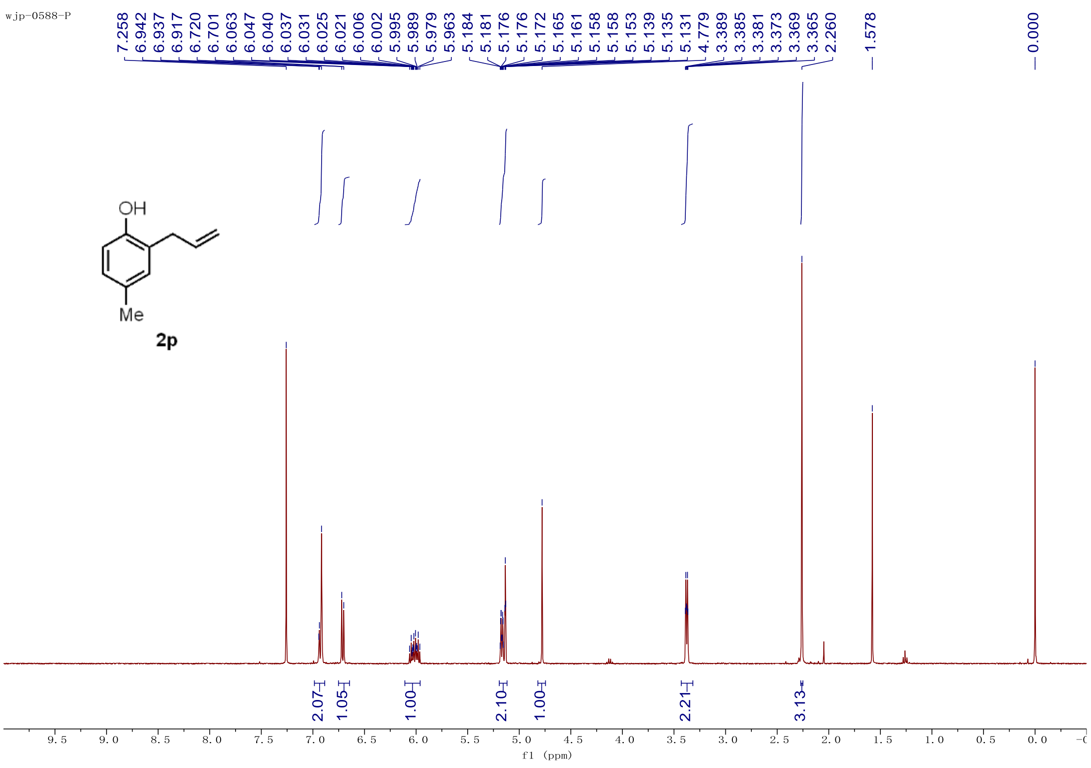

Supplementary Figure 53. ^{1}H NMR (400 MHz, $CDCl_3$) spectrum of 2p.

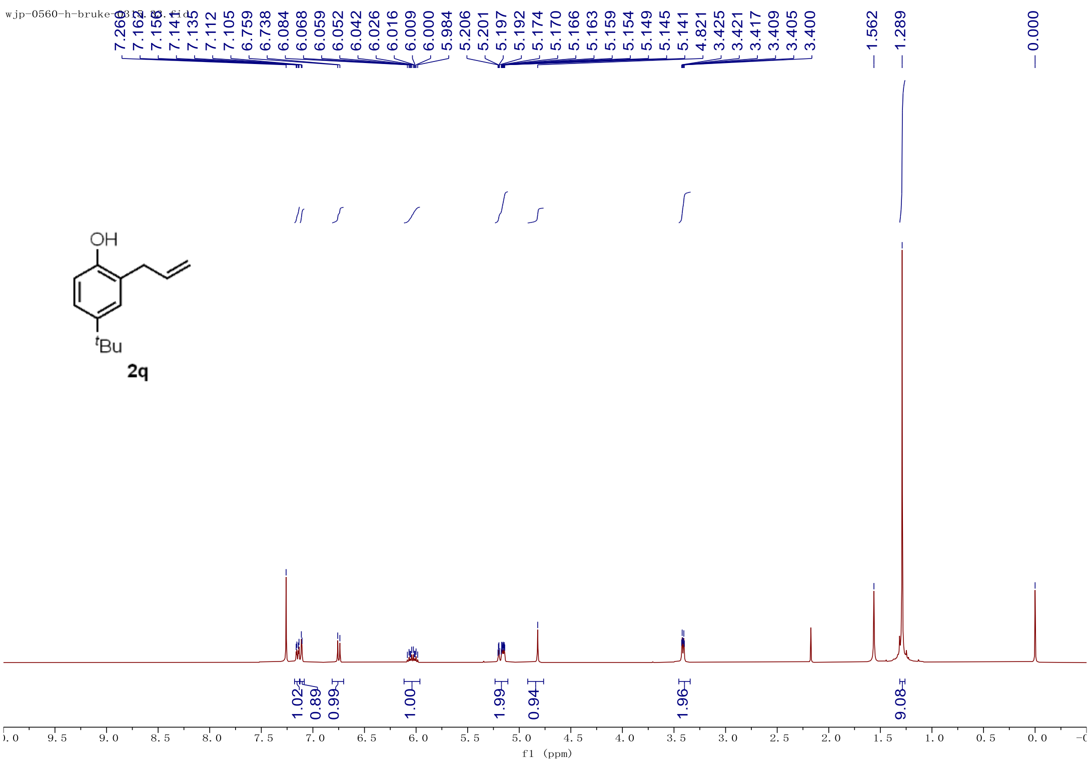

Supplementary Figure 54. ^{1}H NMR (400 MHz, $CDCl_3$) spectrum of 2q.

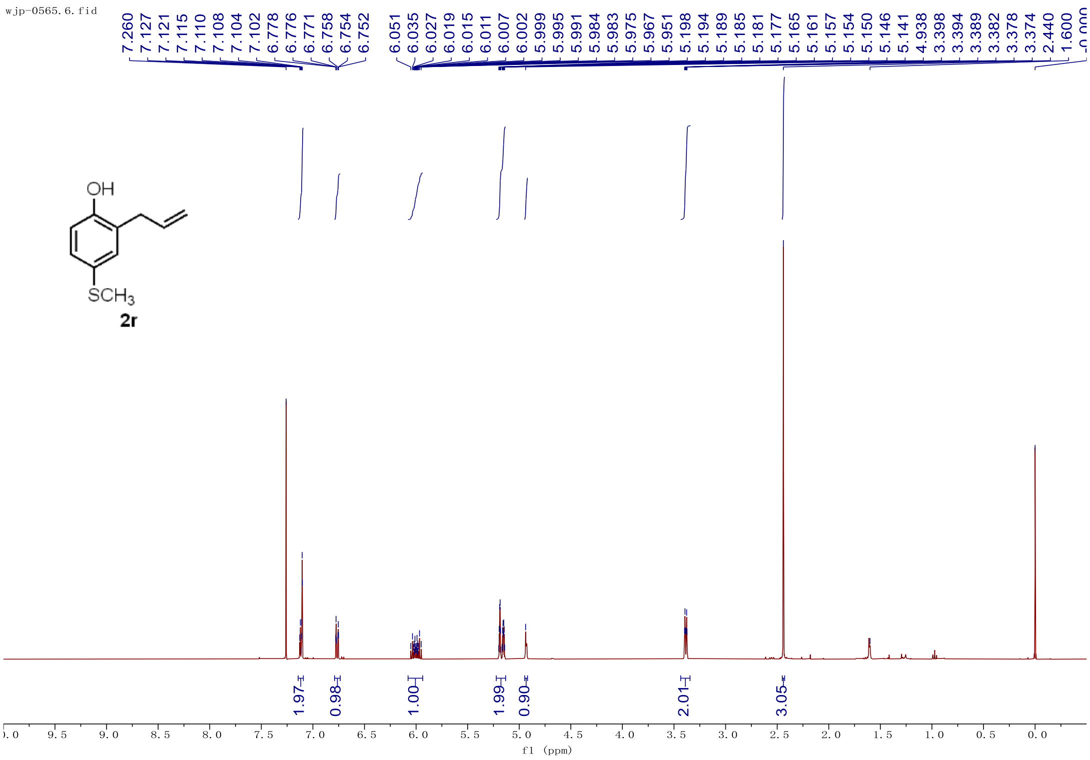

Supplementary Figure 55. ^{1}H NMR (400 MHz, $CDCl_3$) spectrum of 2r.

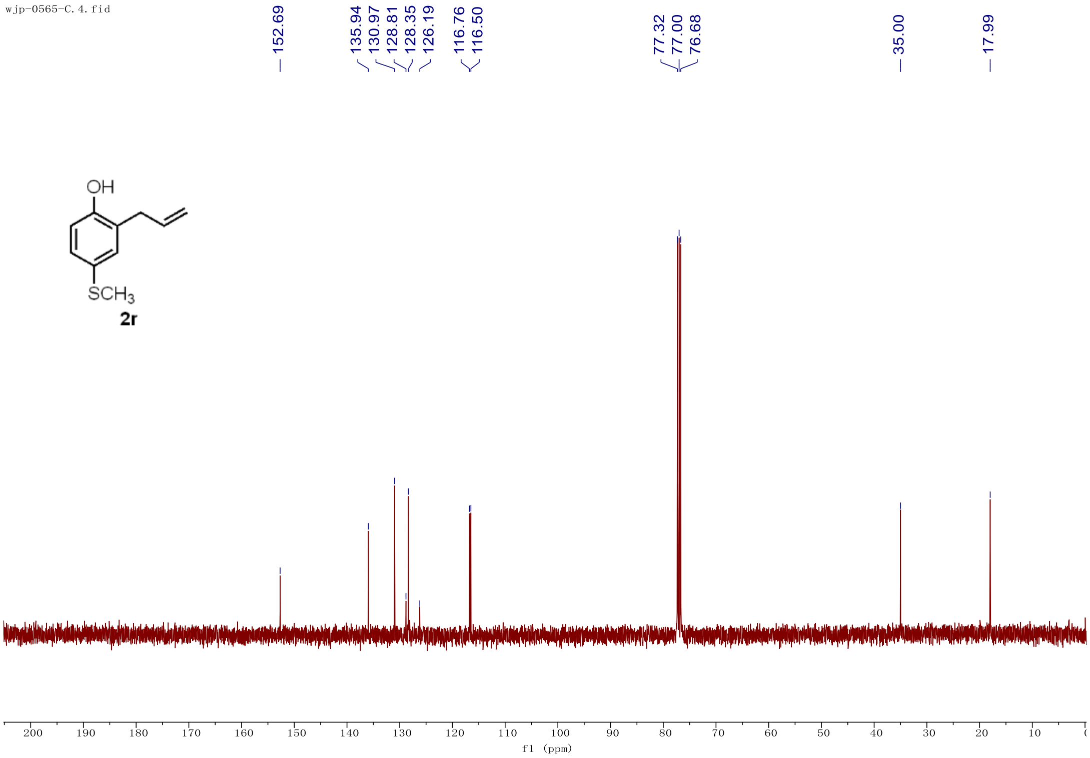

Supplementary Figure 56. ^{13}C NMR (100 MHz, $CDCl_3$) spectrum of 2r.

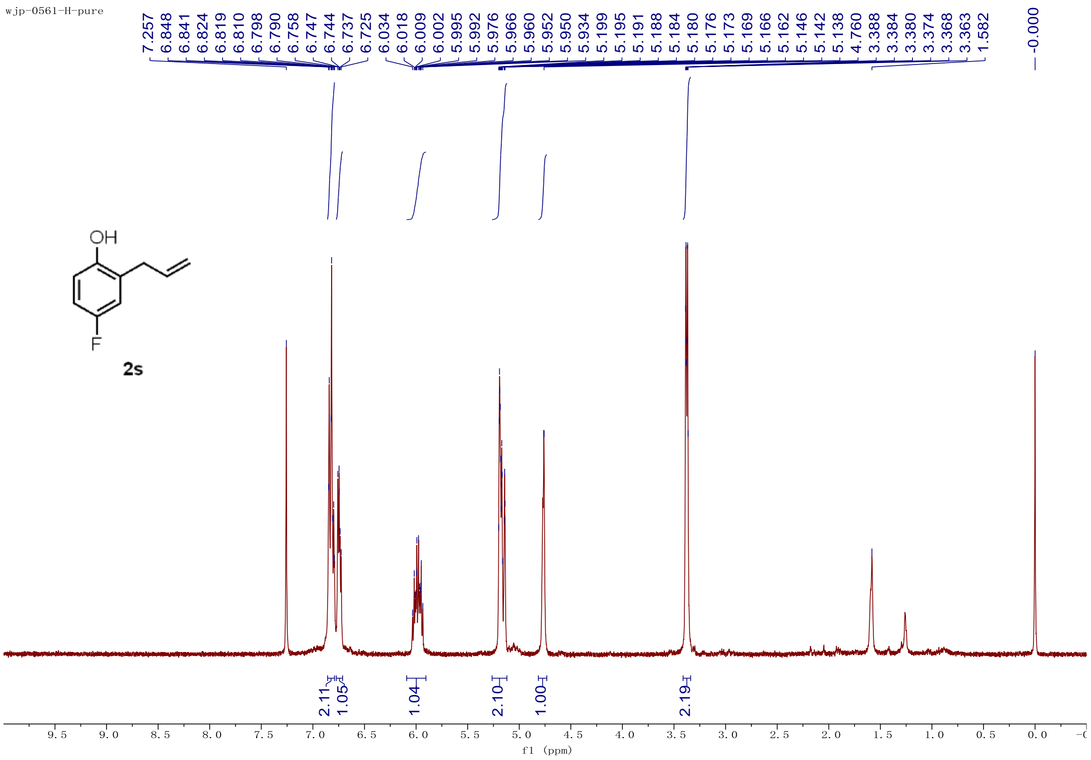

Supplementary Figure 57. ^{1}H NMR (400 MHz, $CDCl_3$) spectrum of 2s.

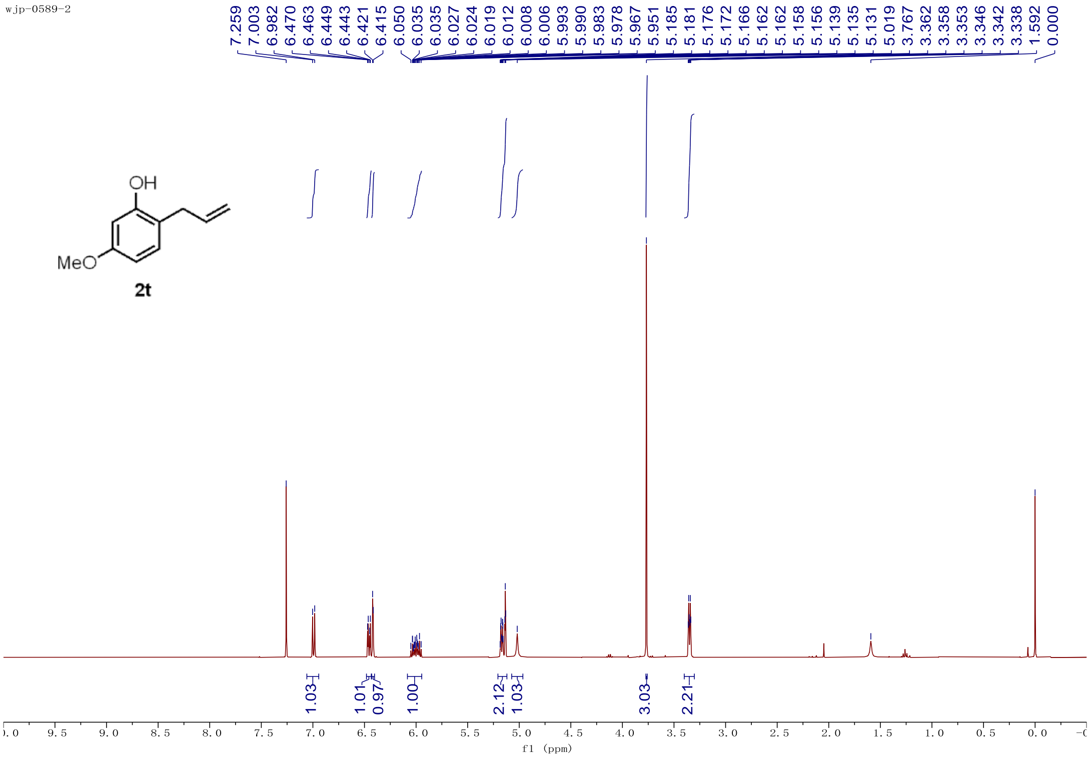

Supplementary Figure 58. ^{1}H NMR (400 MHz, $CDCl_3$) spectrum of 2t.

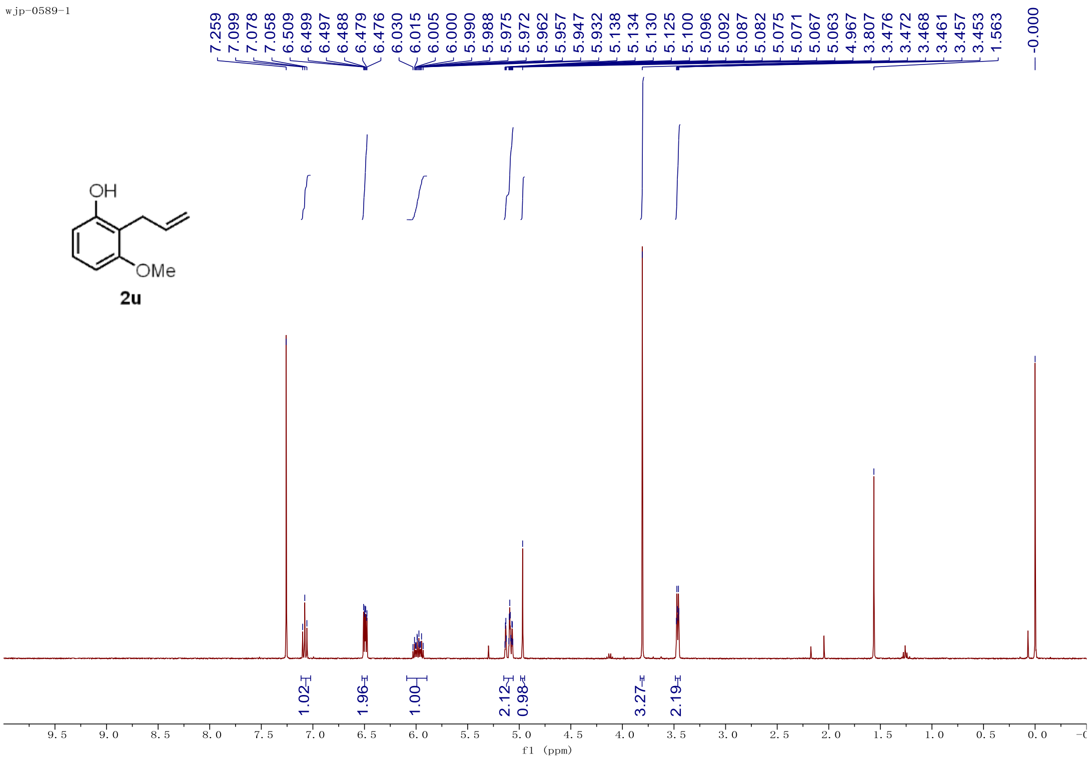

Supplementary Figure 59. ^{1}H NMR (400 MHz, CDCl$_3$) spectrum of 2u.

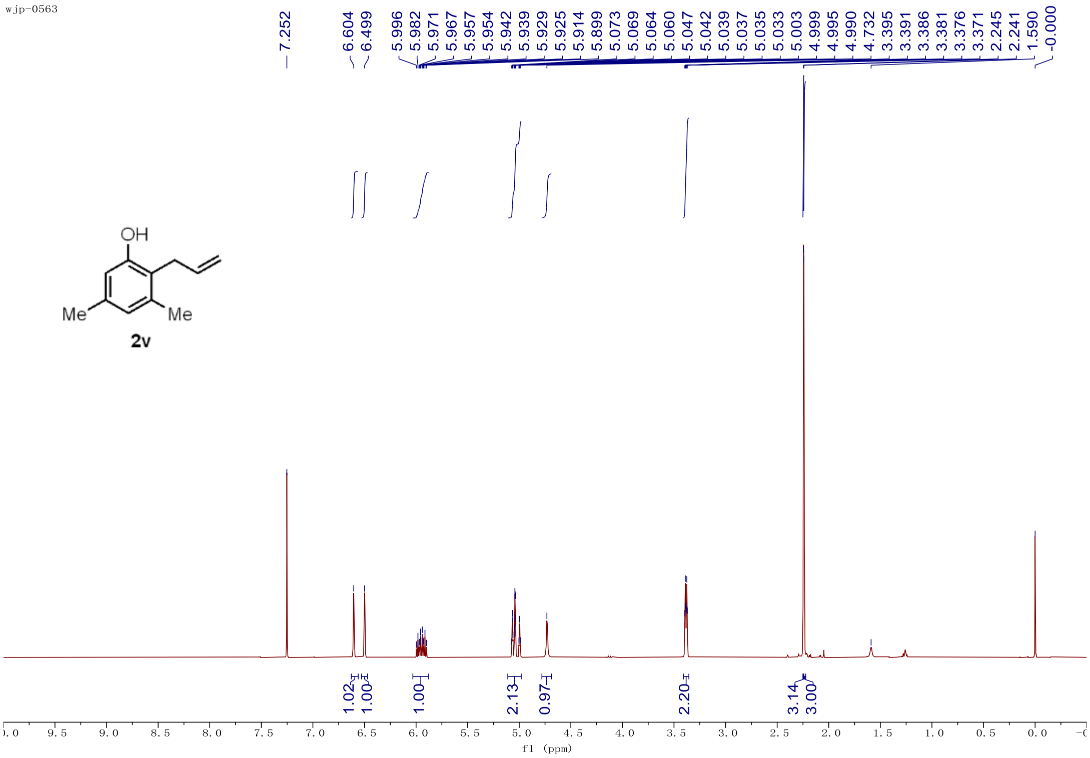

Supplementary Figure 60. ^{1}H NMR (400 MHz, CDCl$_3$) spectrum of 2v.

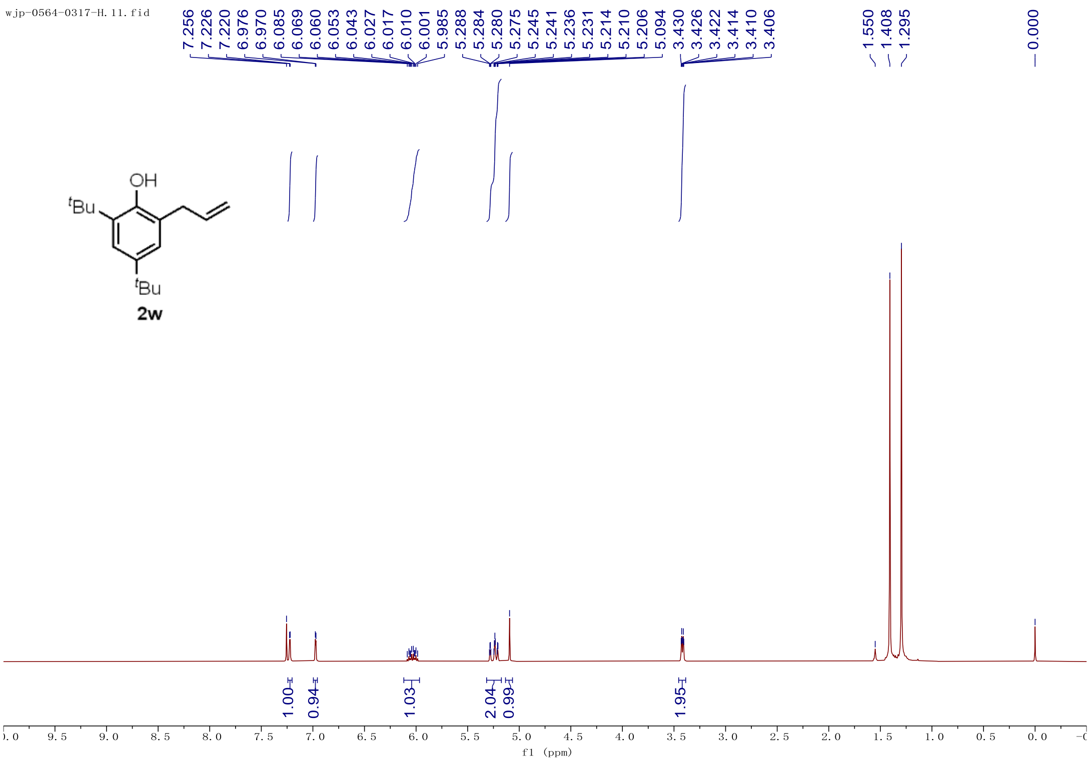

Supplementary Figure 61. ^{1}H NMR (400 MHz, $CDCl_3$) spectrum of 2w.

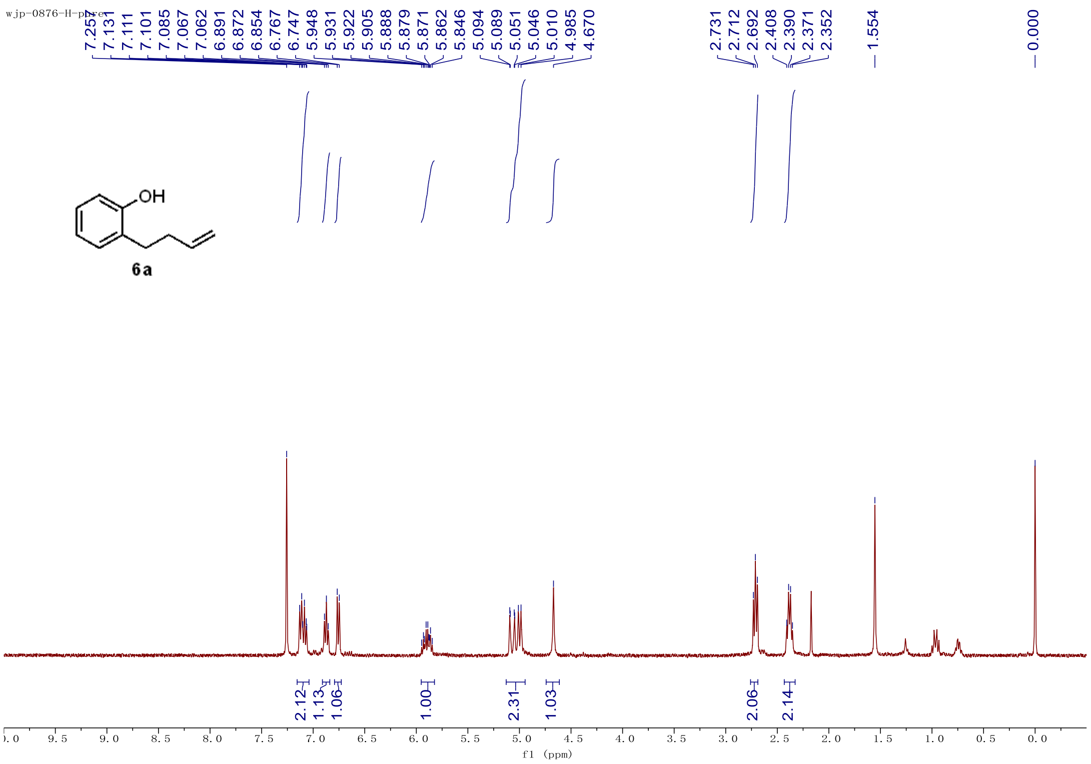

Supplementary Figure 62. ^{1}H NMR (400 MHz, $CDCl_3$) spectrum of 6a.

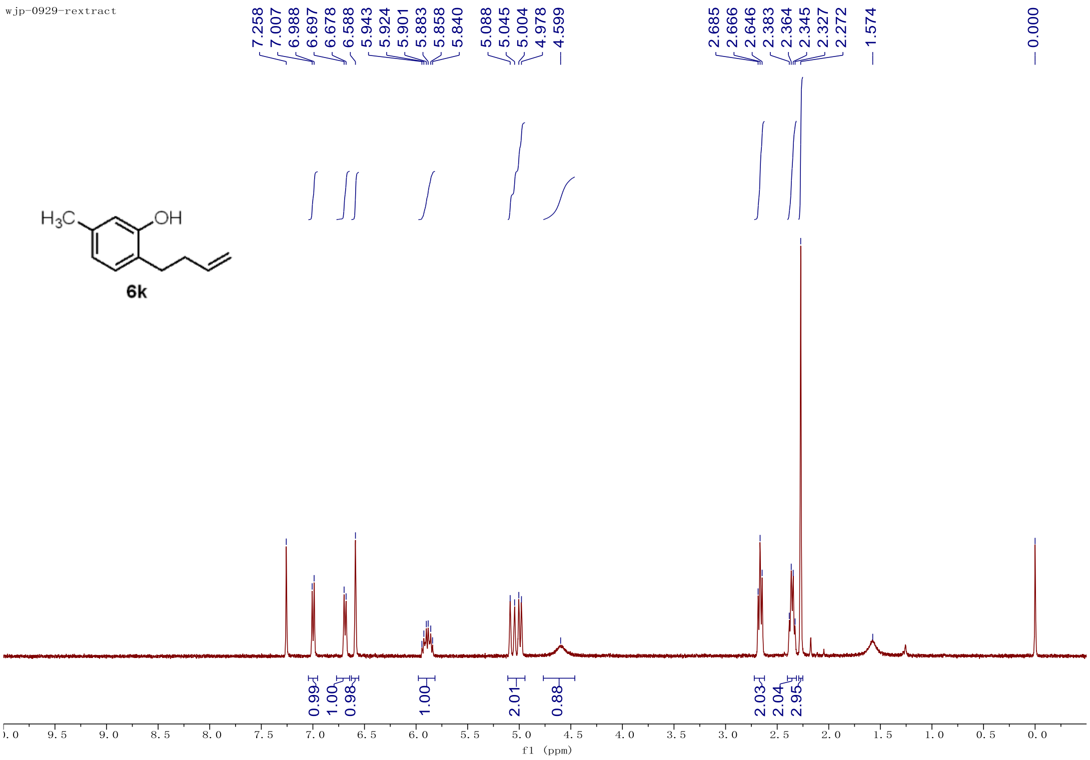

Supplementary Figure 63. ^{1}H NMR (400 MHz, $CDCl_3$) spectrum of 6k.

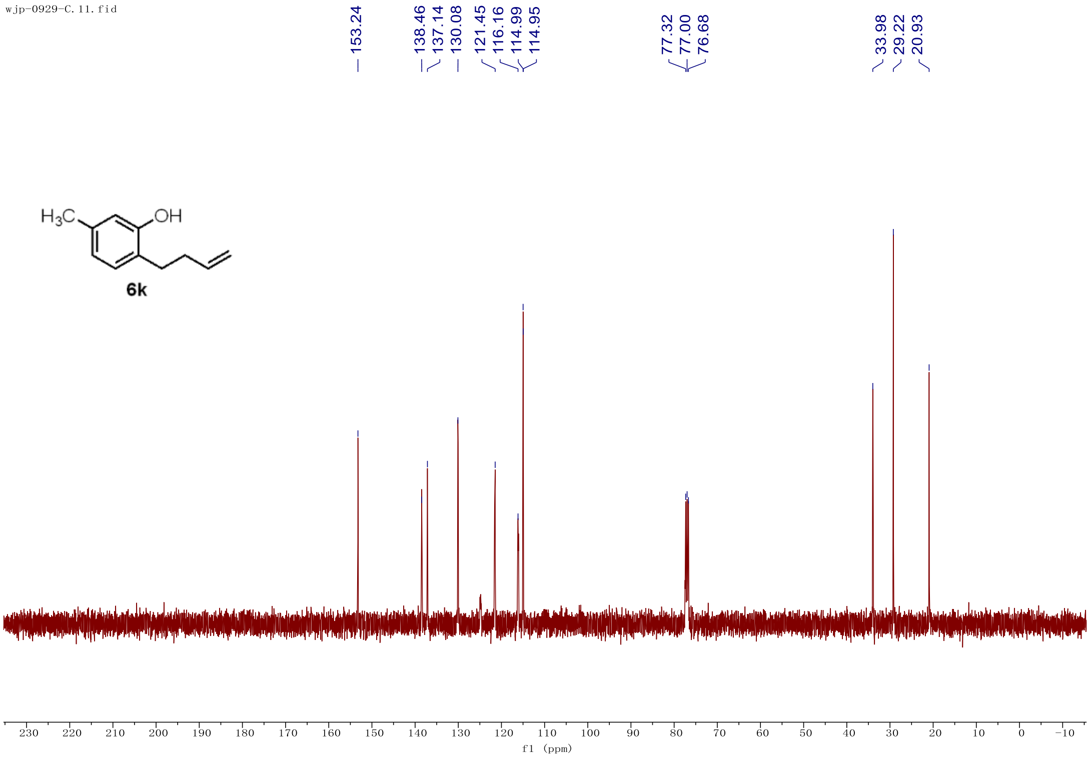

Supplementary Figure 64. ^{13}C NMR (100 MHz, $CDCl_3$) spectrum of 6k.

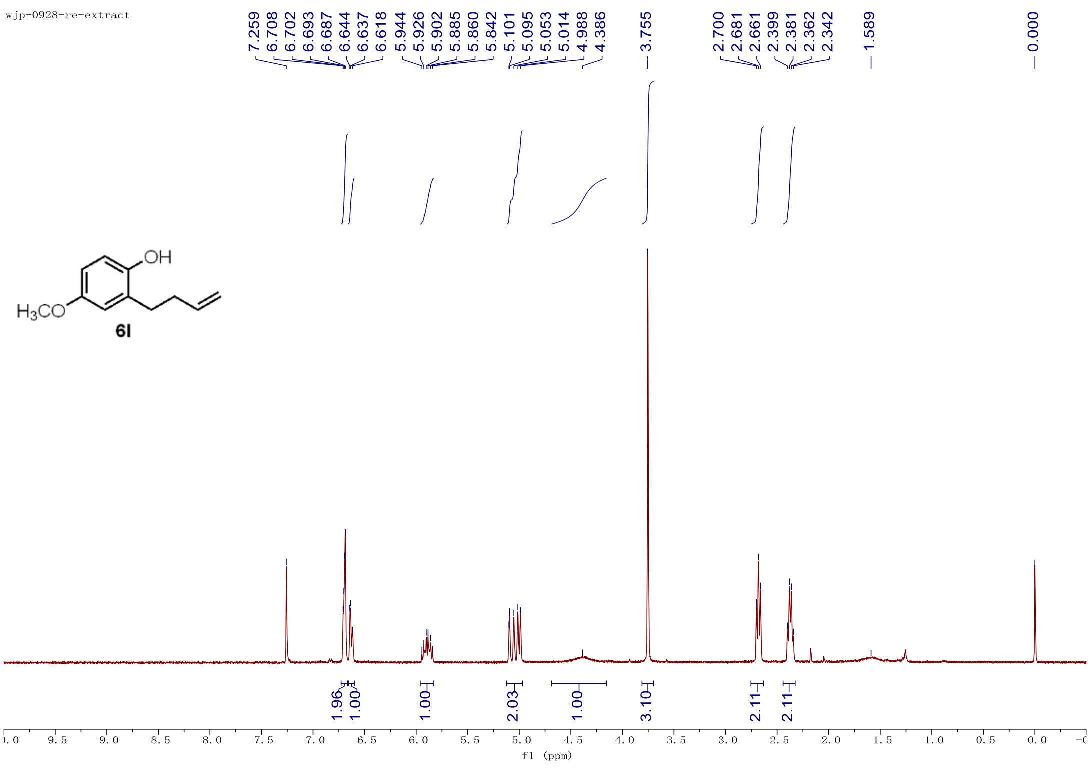

Supplementary Figure 65. ^{1}H NMR (400 MHz, $CDCl_3$) spectrum of 6l.

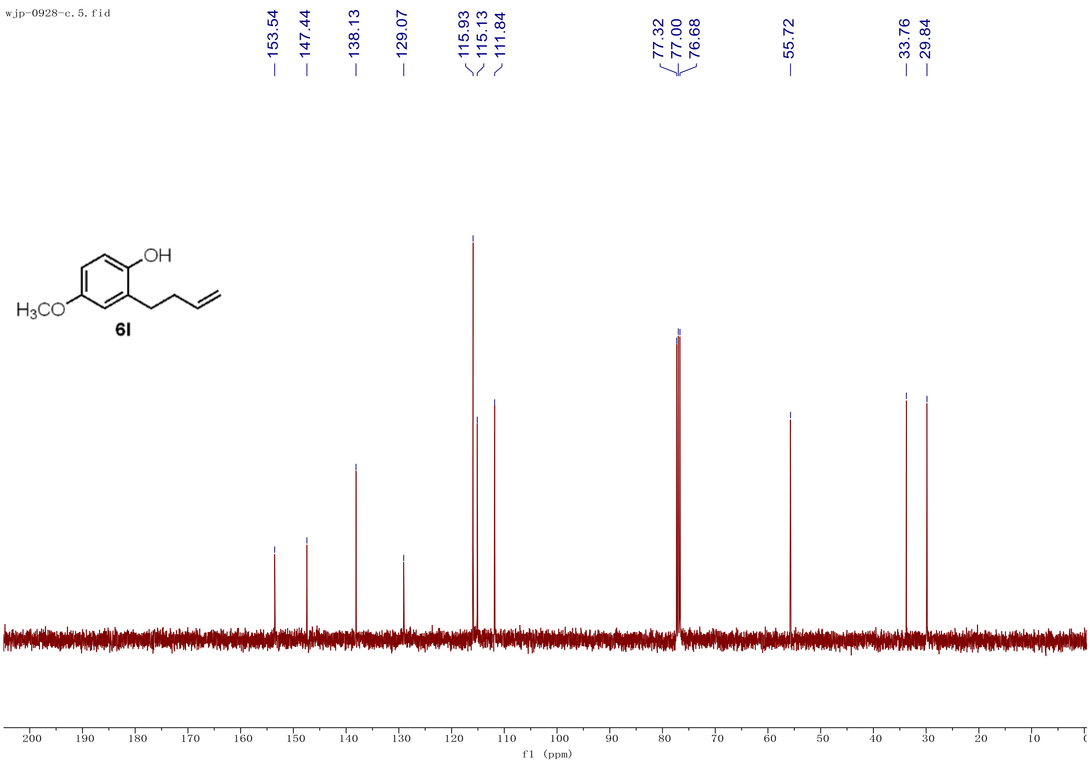

Supplementary Figure 66. ^{13}C NMR (100 MHz, $CDCl_3$) spectrum of 6l.

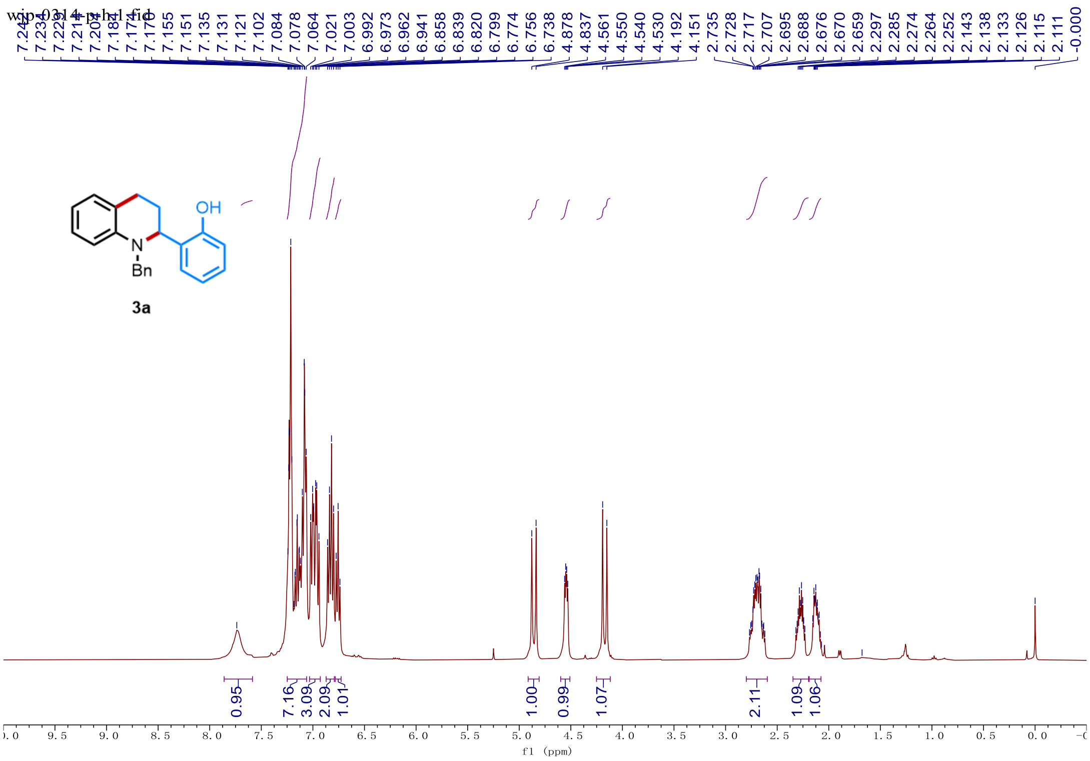

Supplementary Figure 67. ^{1}H NMR (400 MHz, $CDCl_3$) spectrum of 3a.

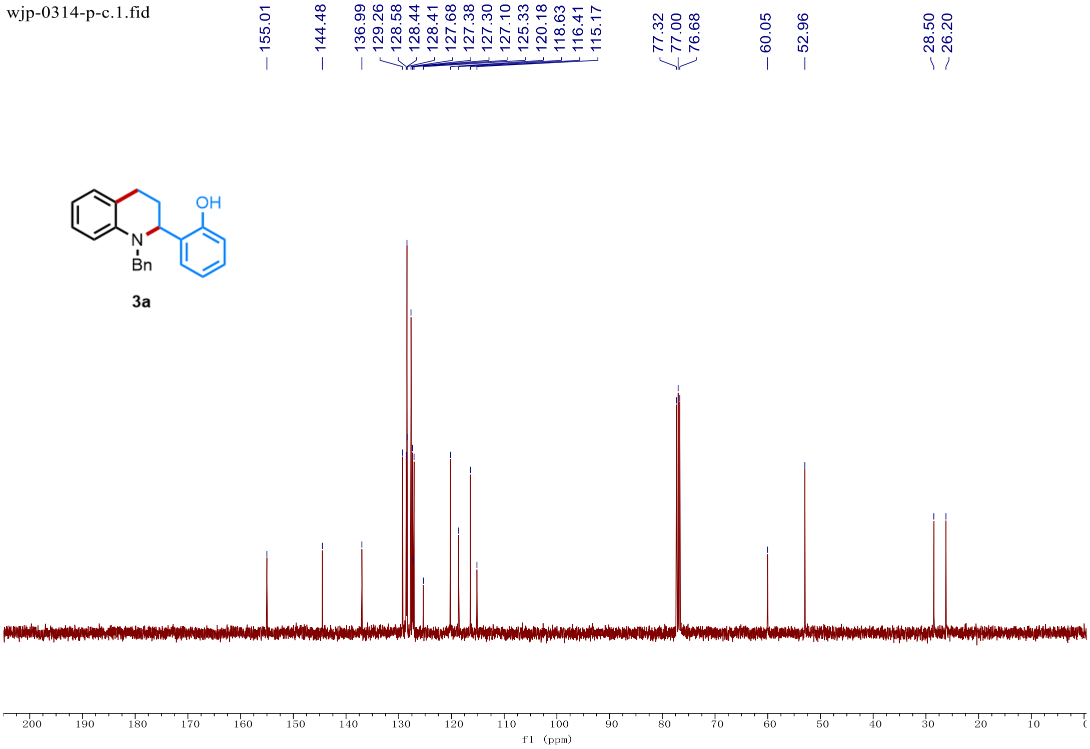

Supplementary Figure 68. ^{13}C NMR (100 MHz, $CDCl_3$) spectrum of 3a.

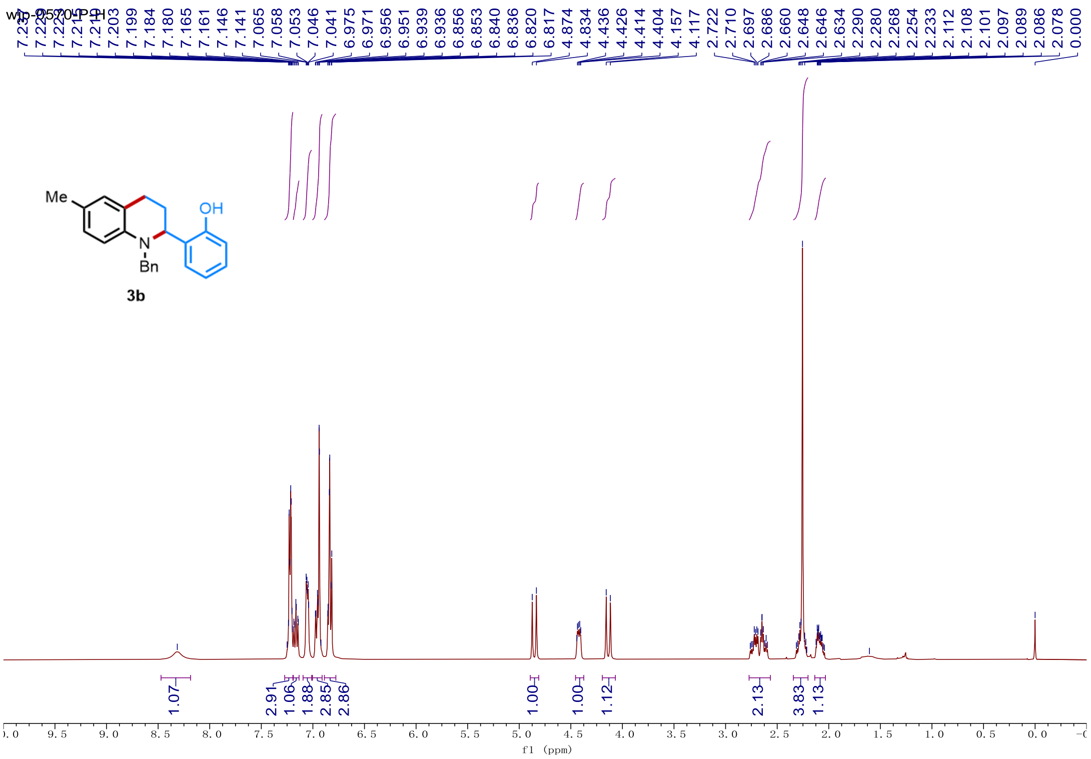

Supplementary Figure 69. ^{1}H NMR (400 MHz, $CDCl_3$) spectrum of 3b.

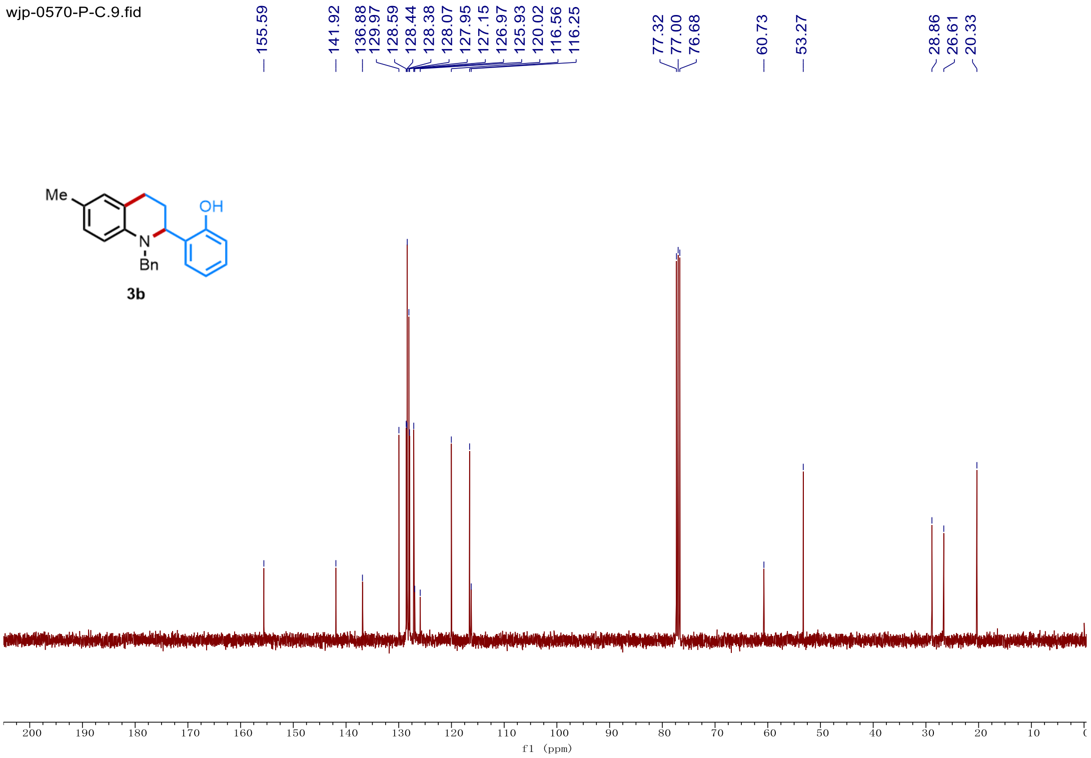

Supplementary Figure 70. ^{13}C NMR (100 MHz, $CDCl_3$) spectrum of 3b.

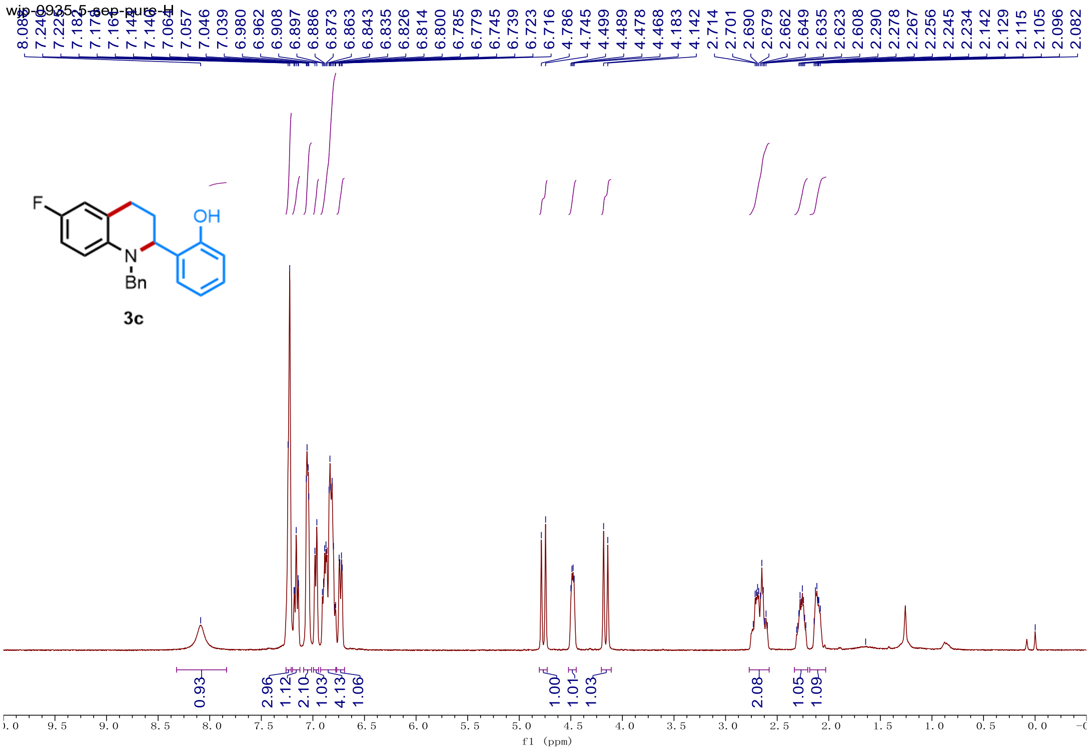

Supplementary Figure 71. ^{1}H NMR (400 MHz, $CDCl_3$) spectrum of 3c.

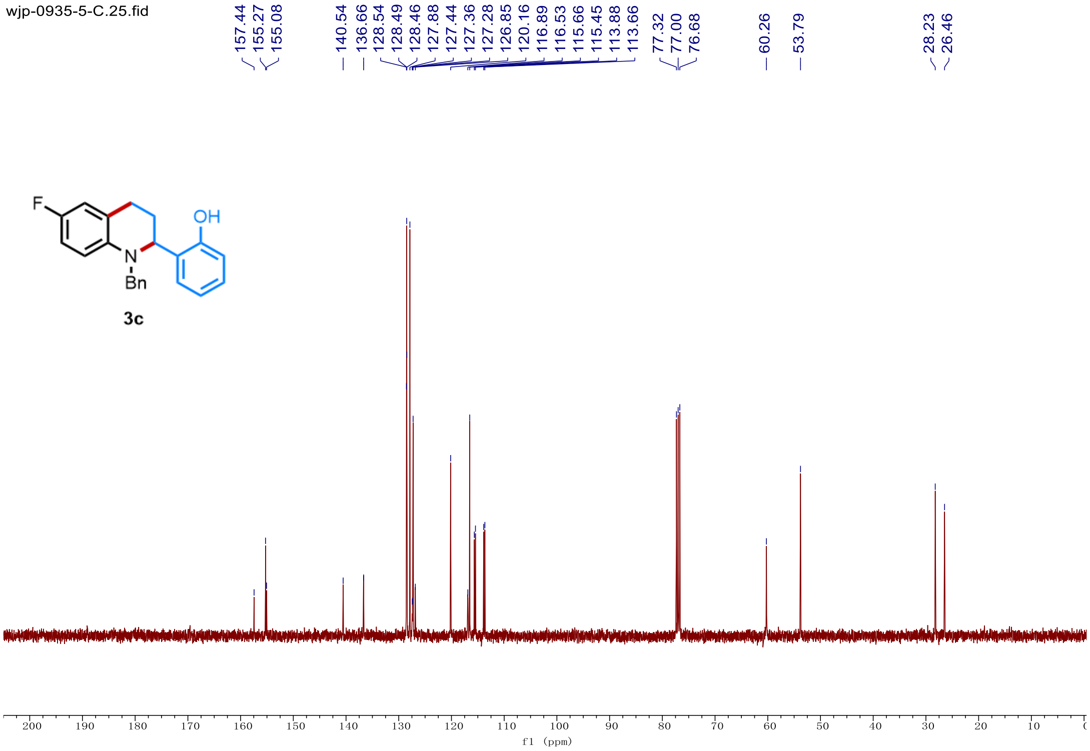

Supplementary Figure 72. ^{13}C NMR (100 MHz, $CDCl_3$) spectrum of 3c.

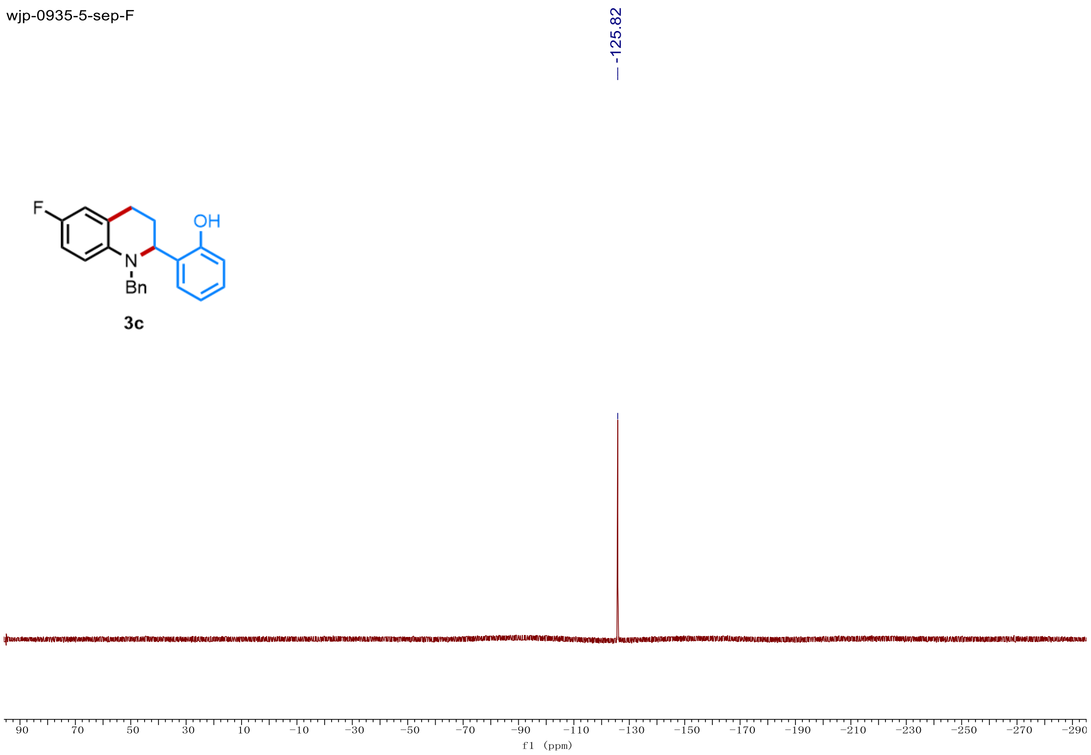

Supplementary Figure 73. ^{19}**F NMR (375 MHz, CDCl$_3$) spectrum of 3c.**

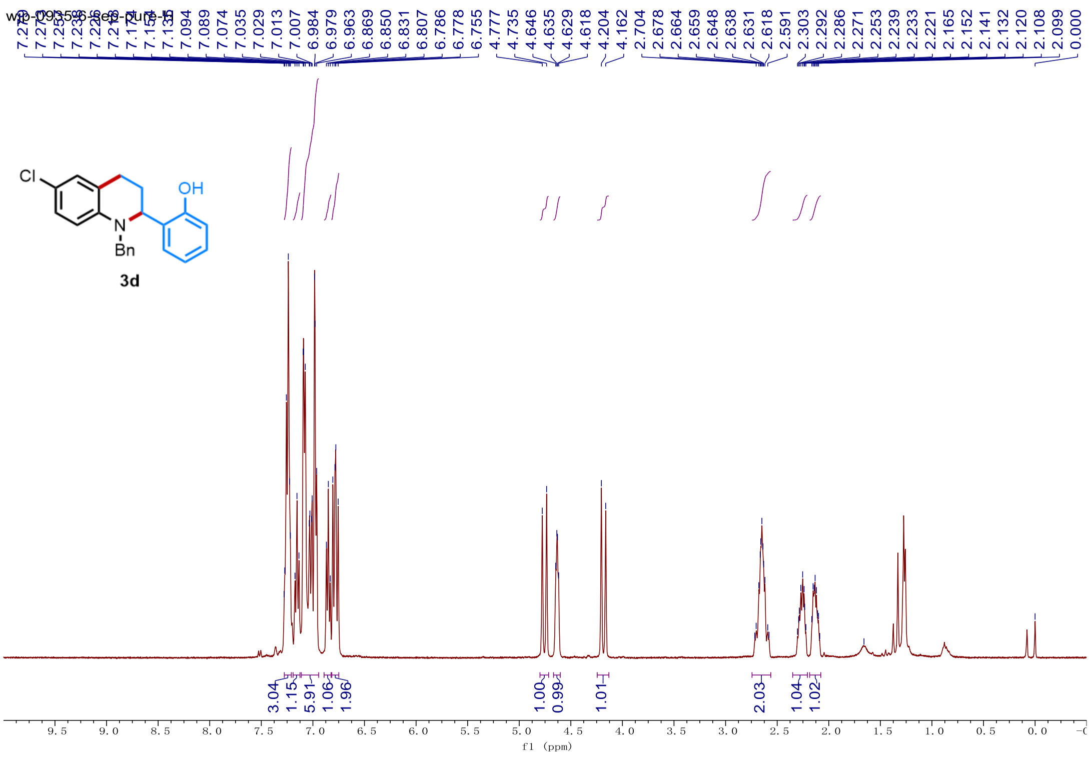

Supplementary Figure 74. ^{1}**H NMR (400 MHz, CDCl$_3$) spectrum of 3d.**

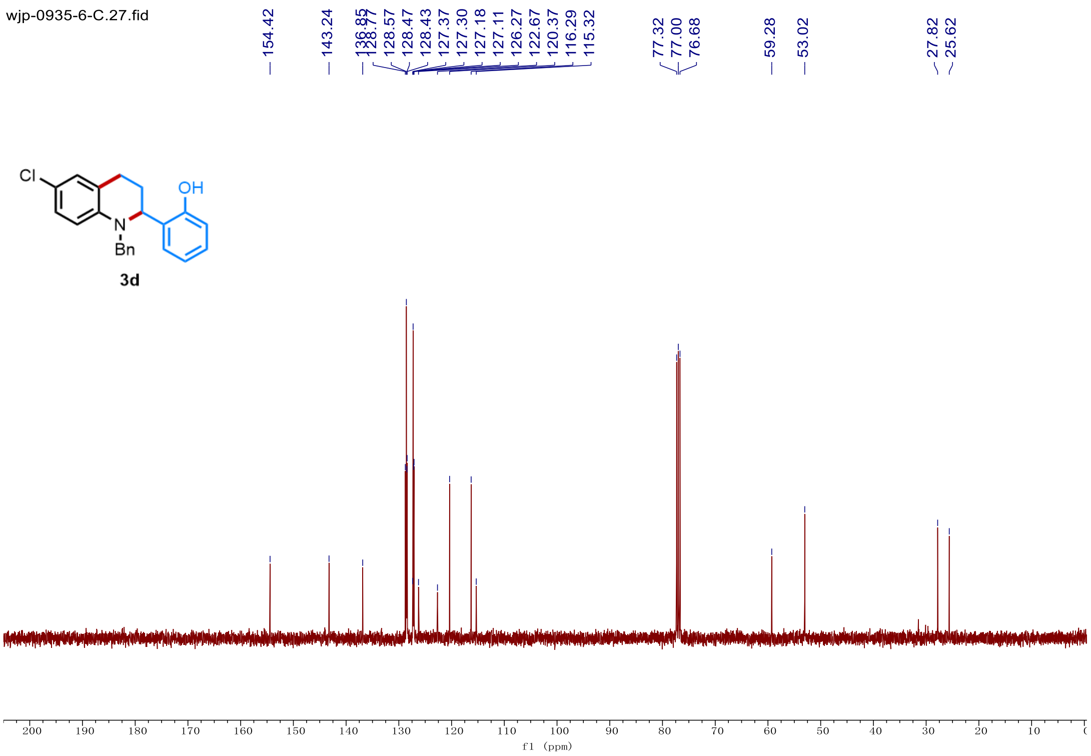

Supplementary Figure 75. ^{13}C NMR (100 MHz, $CDCl_3$) spectrum of 3d.

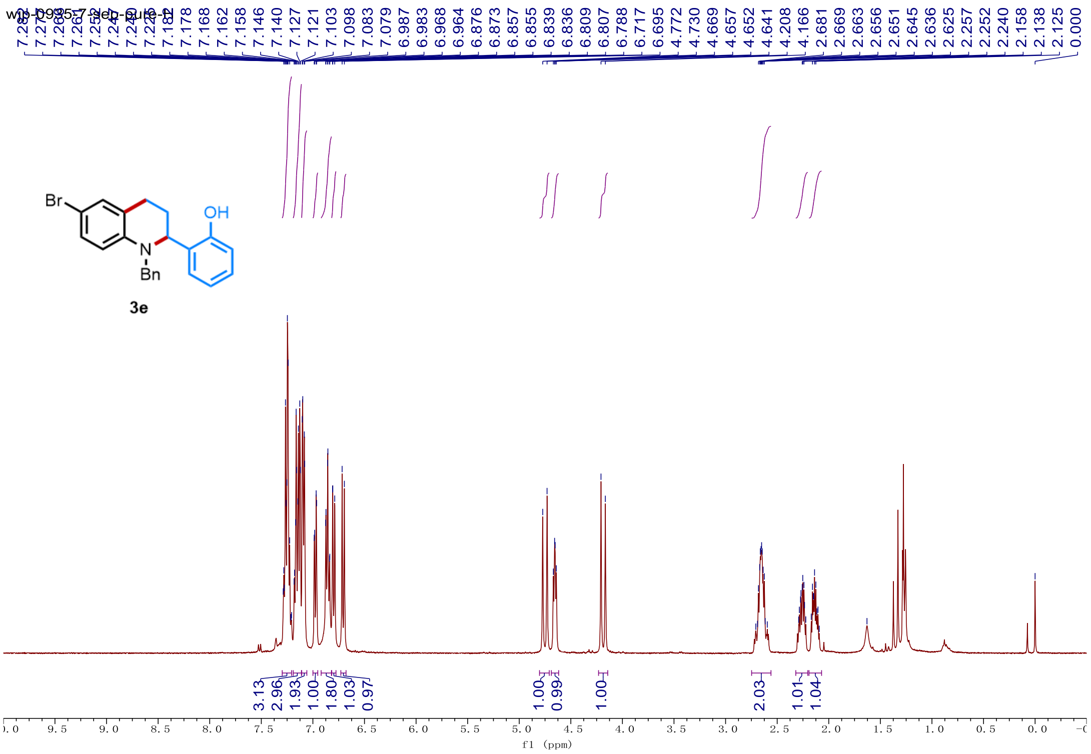

Supplementary Figure 76. ^{1}H NMR (400 MHz, $CDCl_3$) spectrum of 3e.

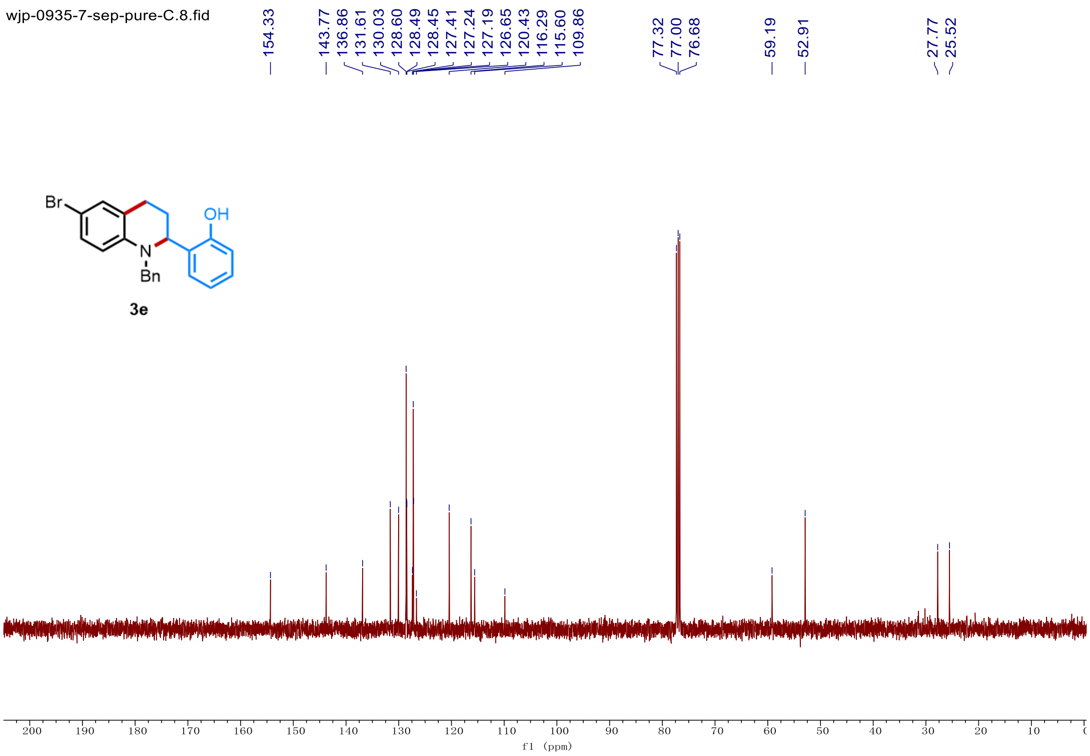

Supplementary Figure 77. ^{13}C NMR (100 MHz, $CDCl_3$) spectrum of 3e.

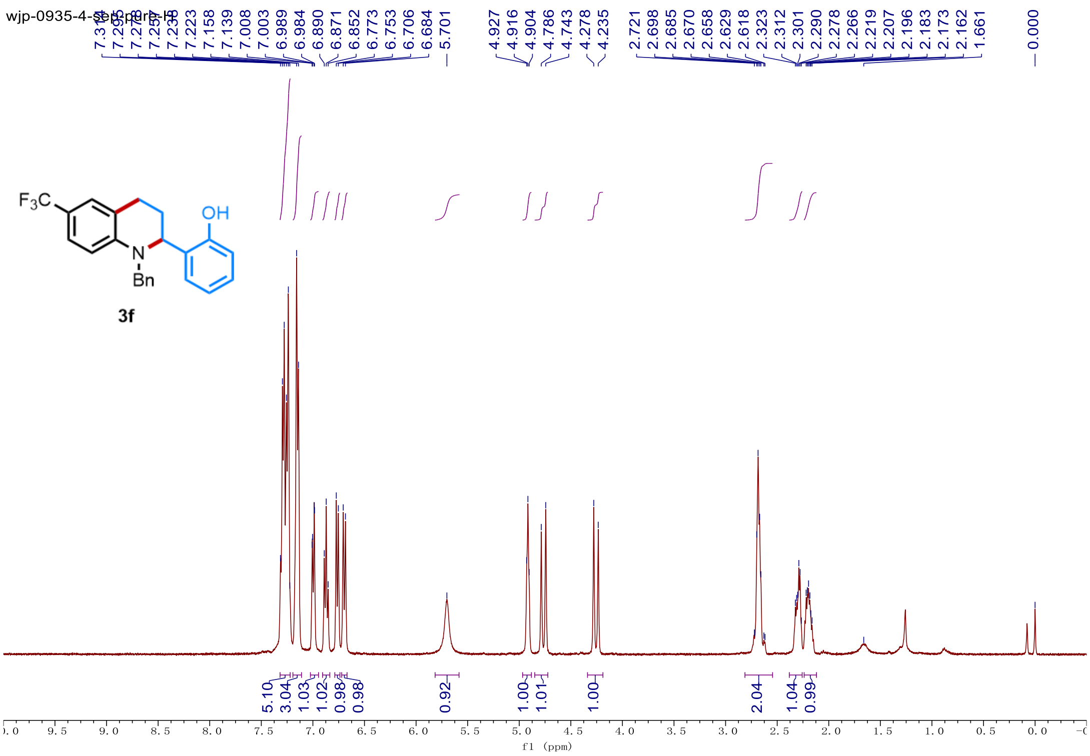

Supplementary Figure 78. ^{1}H NMR (400 MHz, $CDCl_3$) spectrum of 3f.

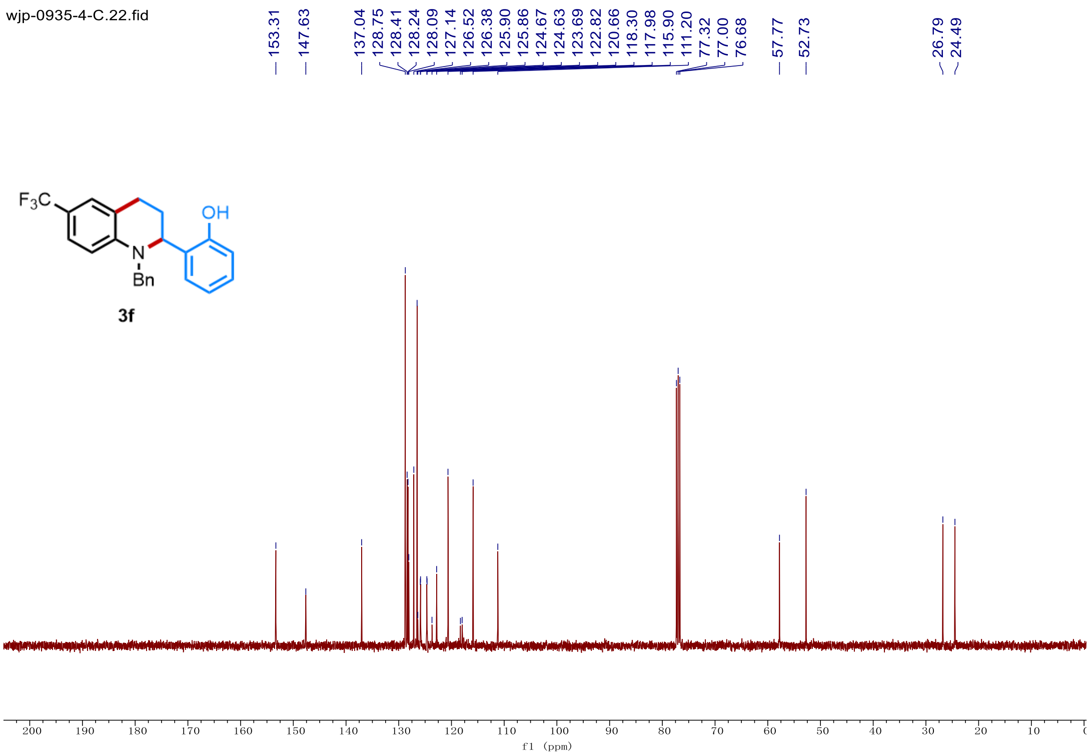

Supplementary Figure 79. ^{13}C NMR (100 MHz, $CDCl_3$) spectrum of 3f.

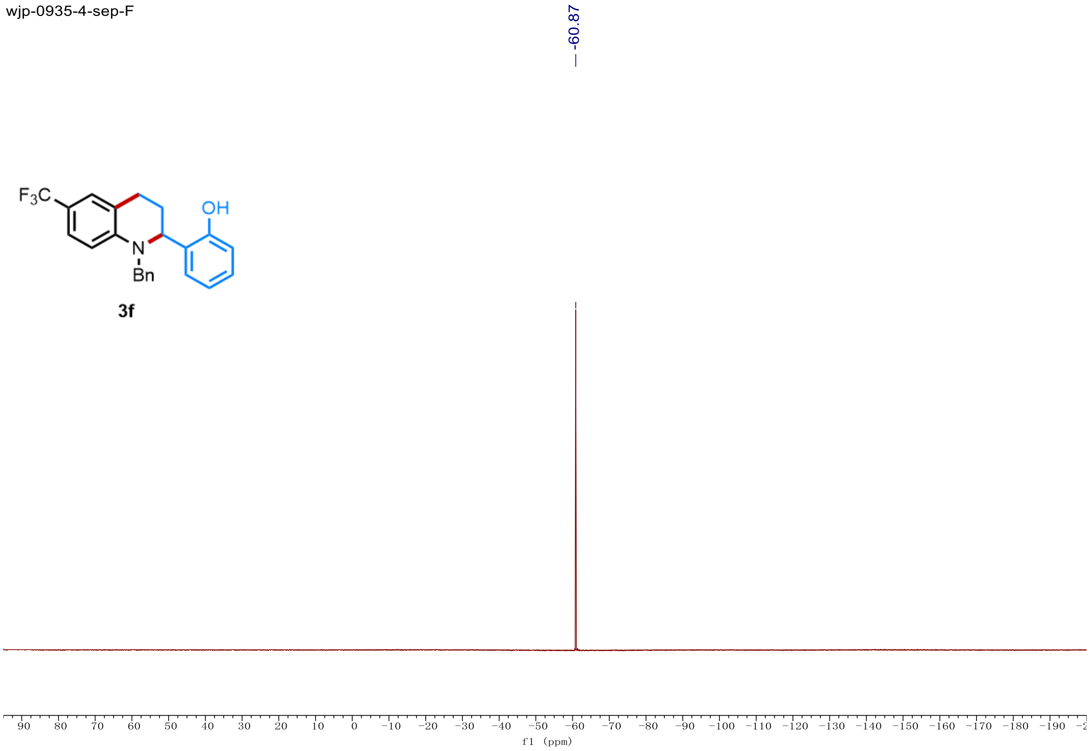

Supplementary Figure 80. ^{19}F NMR (375 MHz, $CDCl_3$) spectrum of 3f.

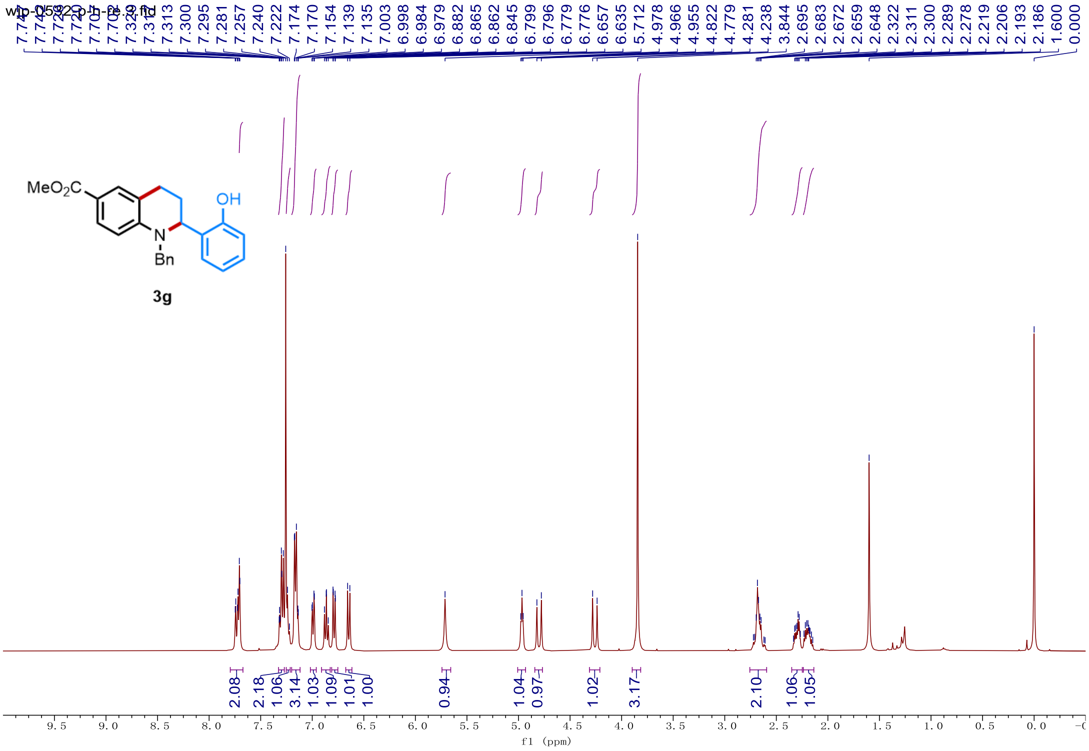

Supplementary Figure 81. ^{1}H NMR (400 MHz, $CDCl_3$) spectrum of 3g.

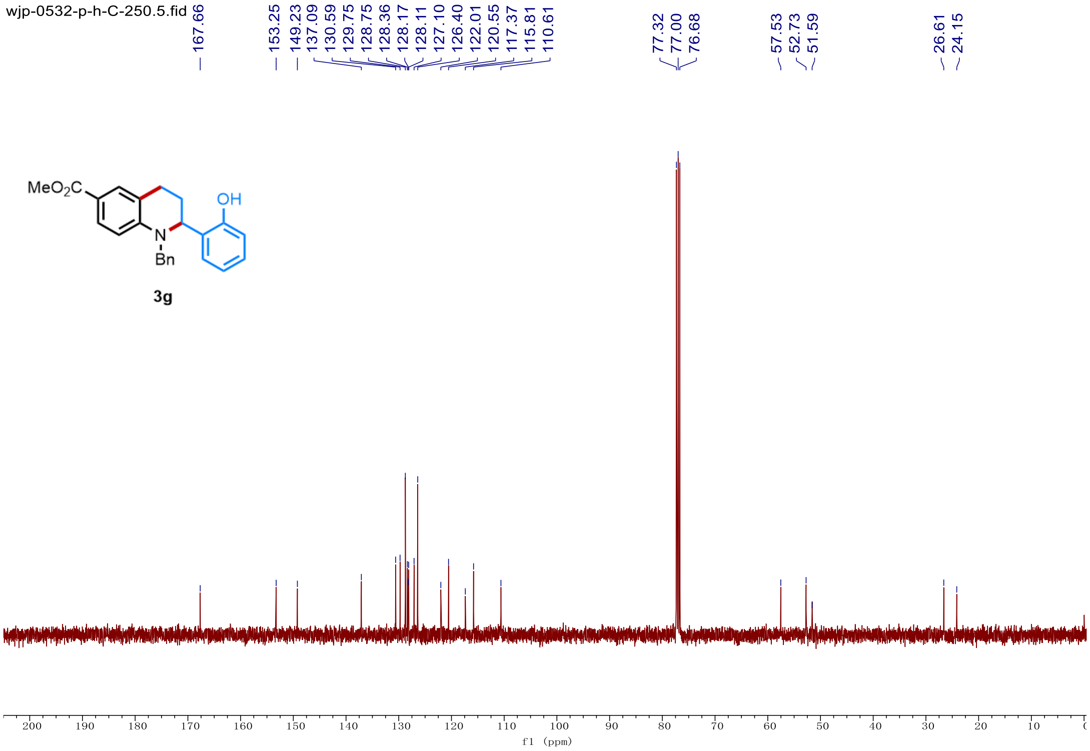

Supplementary Figure 82. ^{13}C NMR (100 MHz, $CDCl_3$) spectrum of 3g.

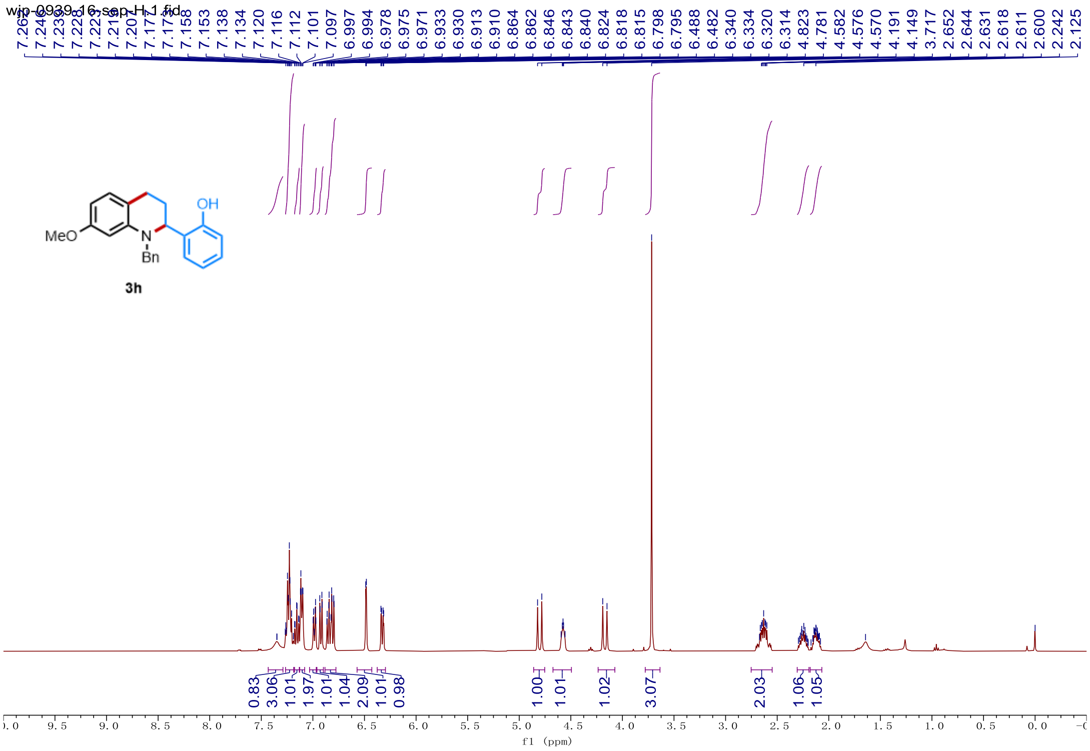

Supplementary Figure 83. ^{1}H NMR (400 MHz, $CDCl_3$) spectrum of 3h.

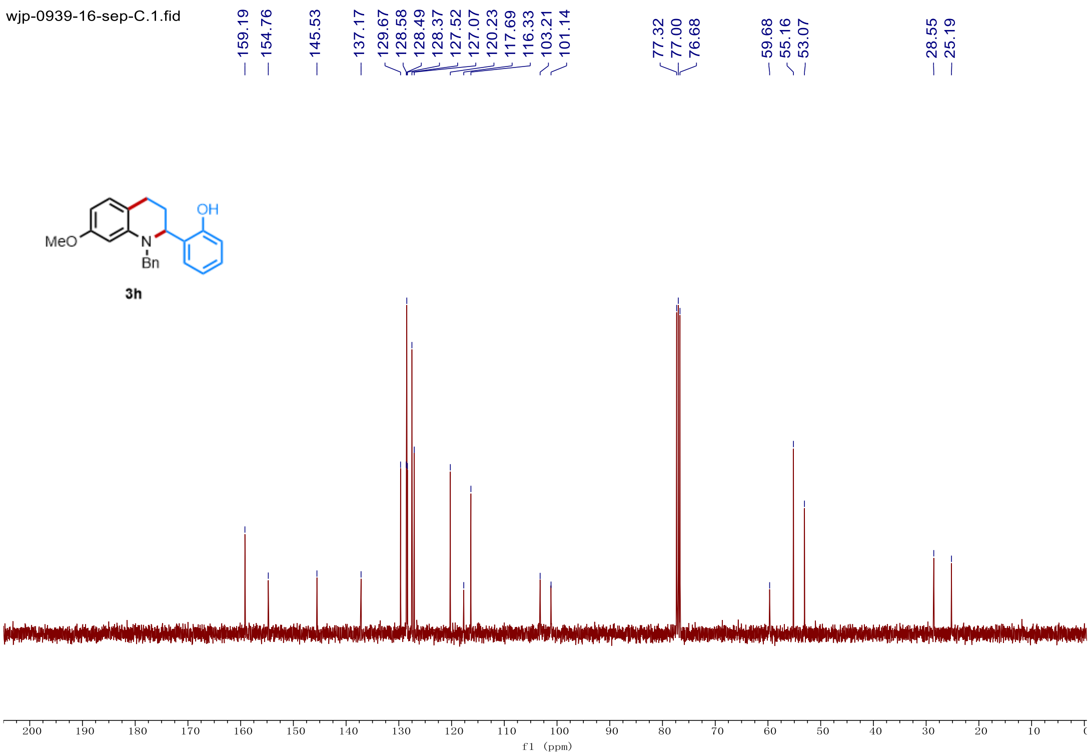

Supplementary Figure 84. ^{13}C NMR (100 MHz, $CDCl_3$) spectrum of 3h.

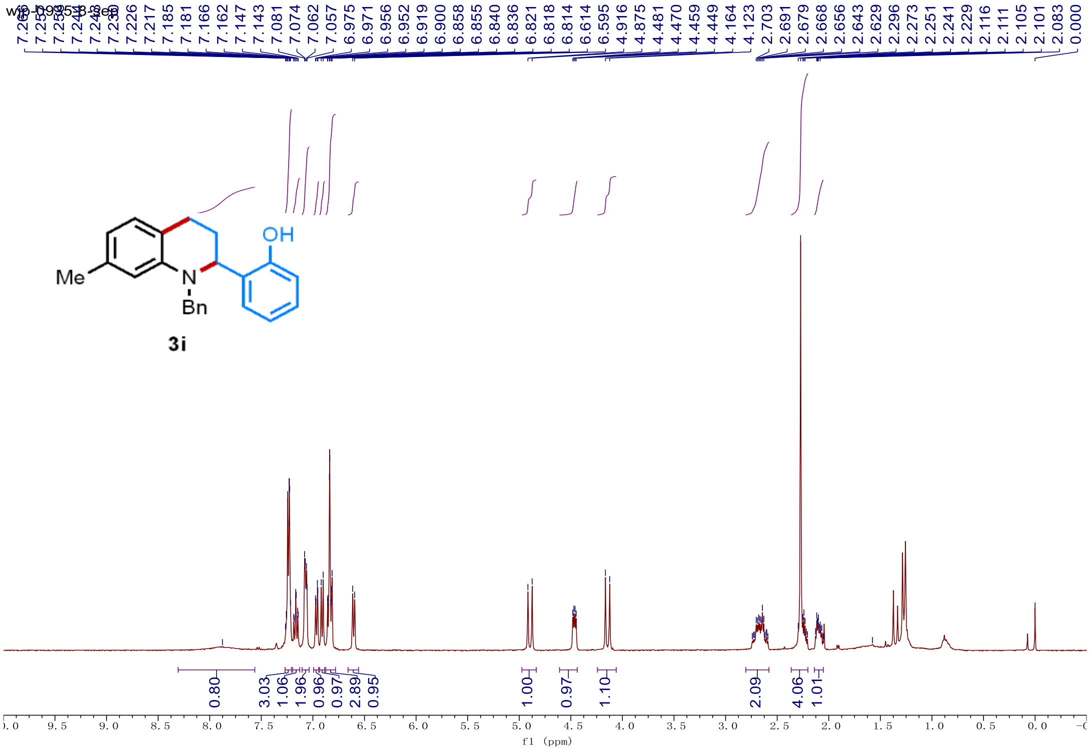

Supplementary Figure 85. **^{1}H NMR (400 MHz, $CDCl_3$) spectrum of 3i.**

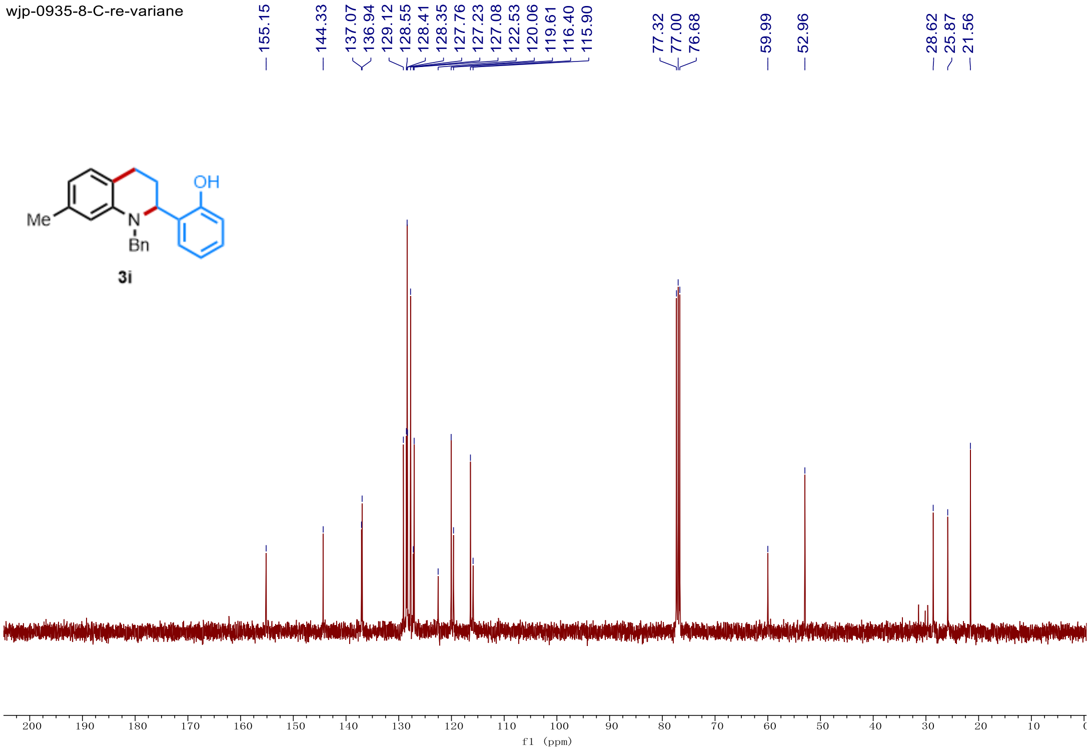

Supplementary Figure 86. **^{13}C NMR (100 MHz, $CDCl_3$) spectrum of 3i.**

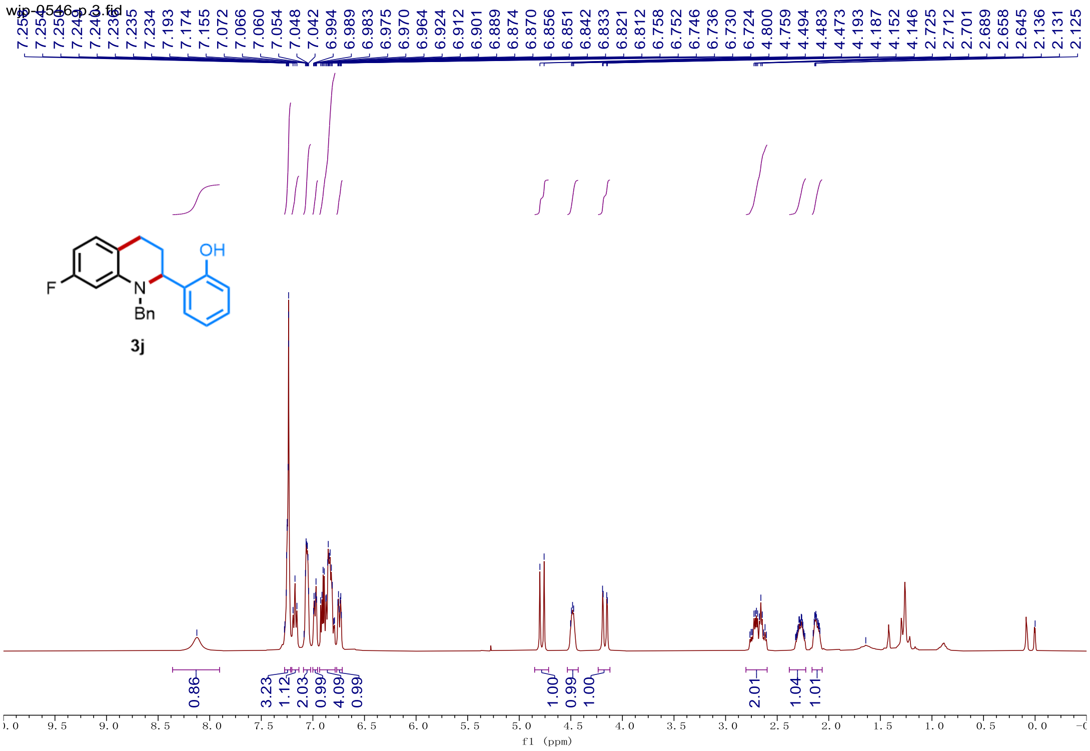

Supplementary Figure 87. ^{1}H NMR (400 MHz, $CDCl_3$) spectrum of 3j.

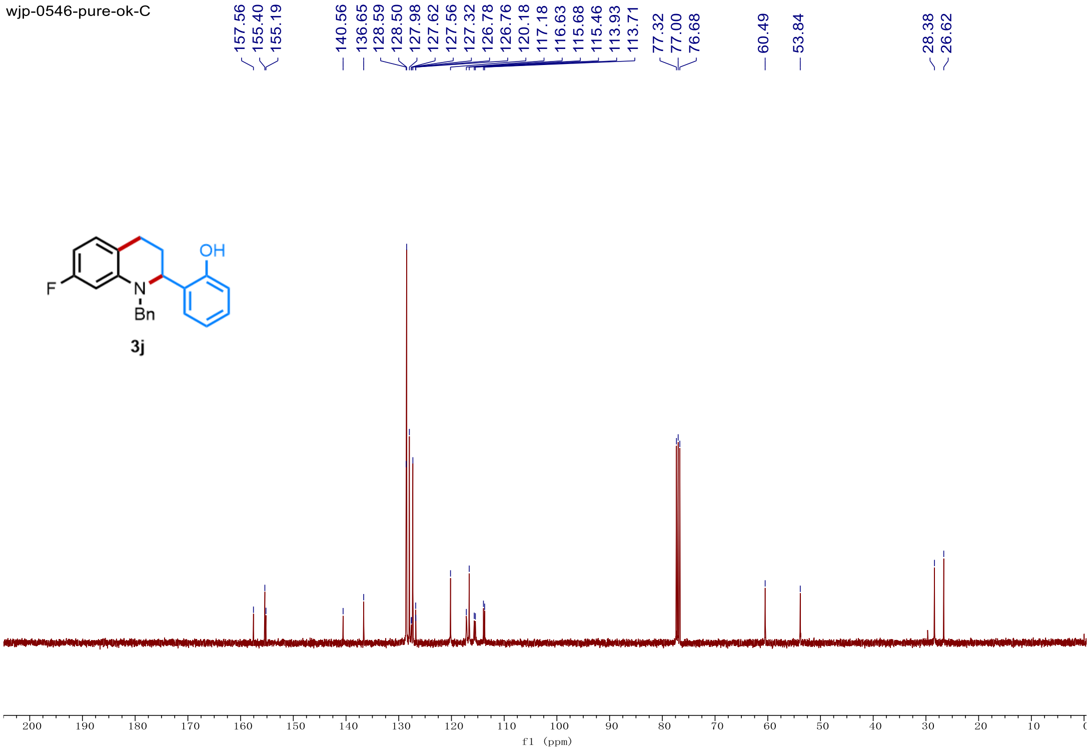

Supplementary Figure 88. ^{13}C NMR (100 MHz, $CDCl_3$) spectrum of 3j.

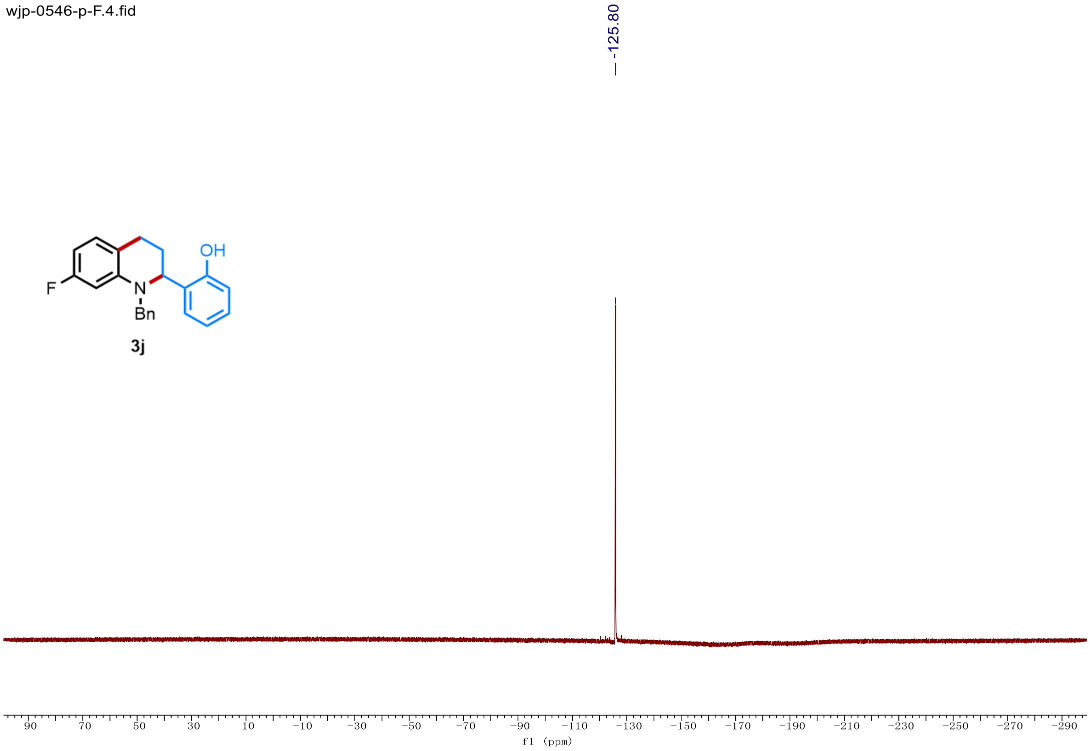

Supplementary Figure 89. ^{19}F NMR (375 MHz, $CDCl_3$) spectrum of 3j.

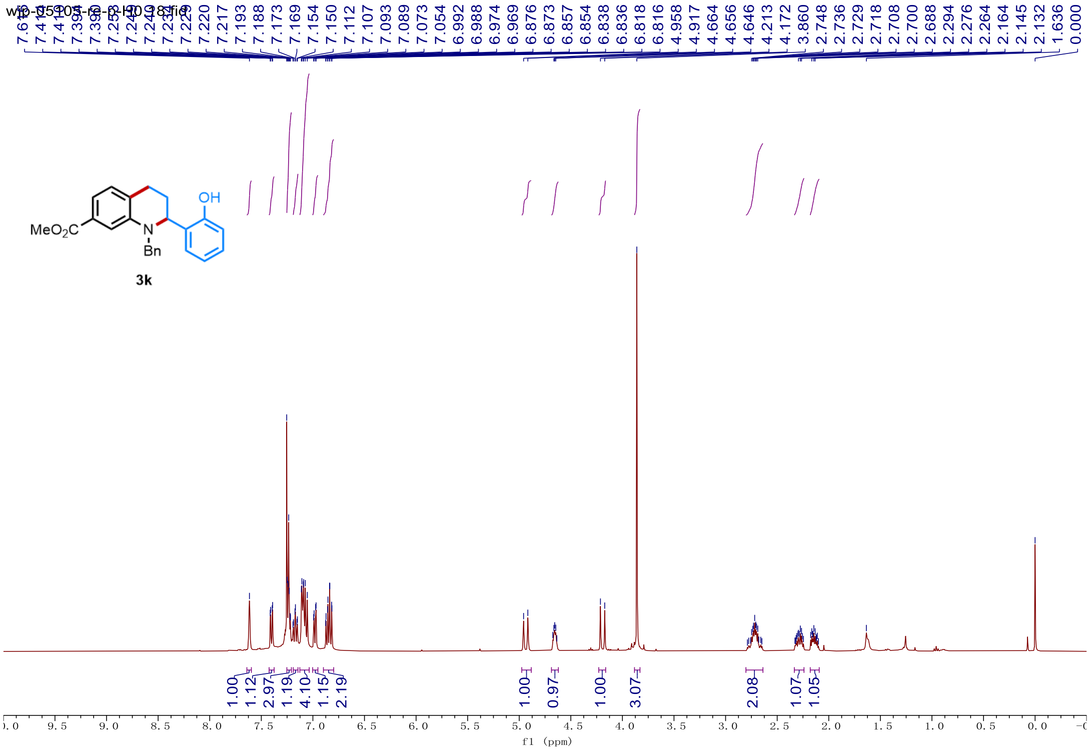

Supplementary Figure 90. ^{1}H NMR (400 MHz, $CDCl_3$) spectrum of 3k.

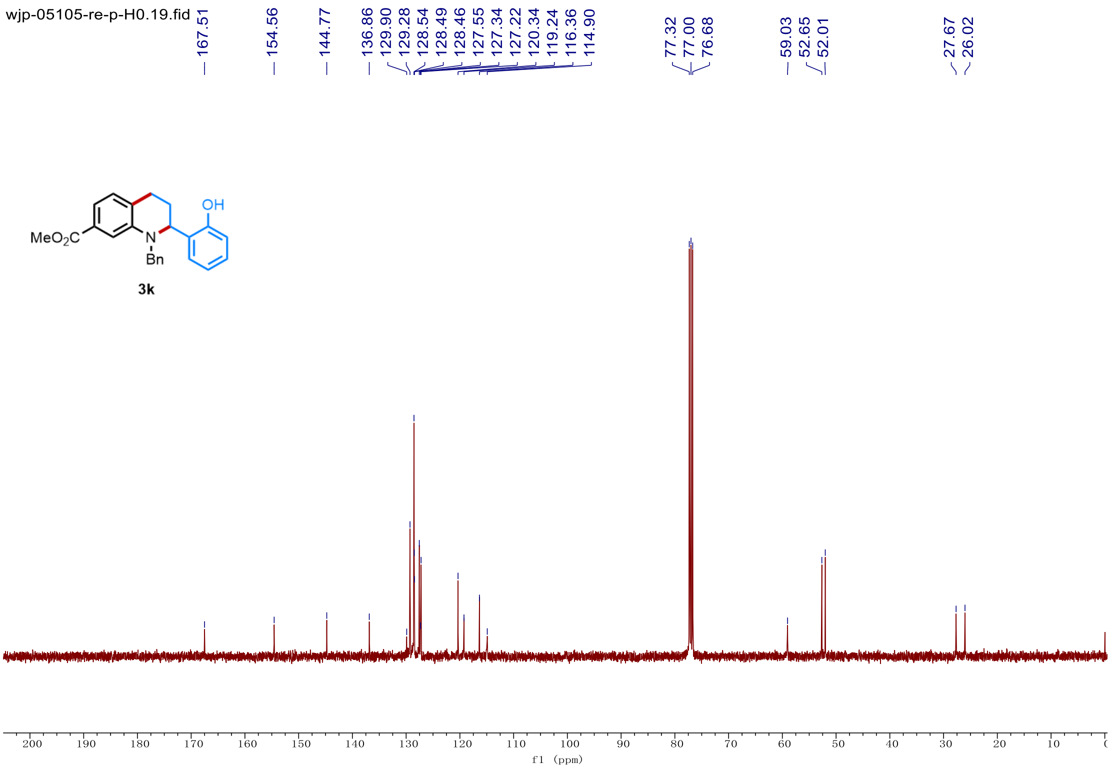

Supplementary Figure 91. ^{13}C NMR (100 MHz, $CDCl_3$) spectrum of 3k.

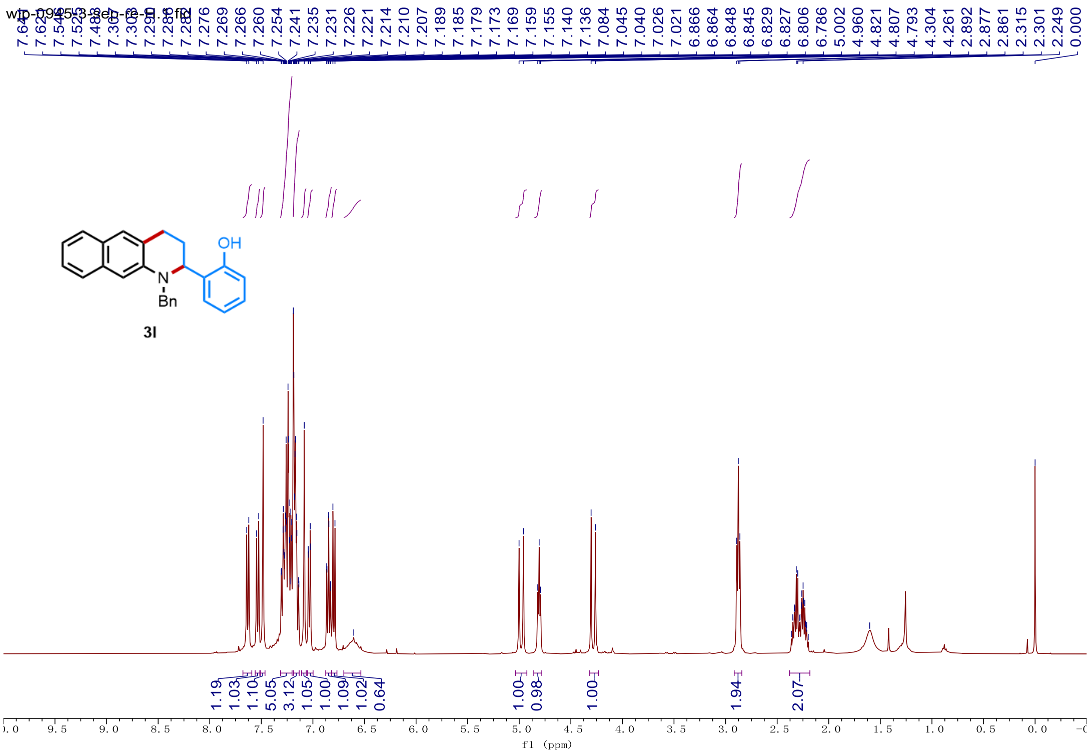

Supplementary Figure 92. ^{1}H NMR (400 MHz, $CDCl_3$) spectrum of 3l.

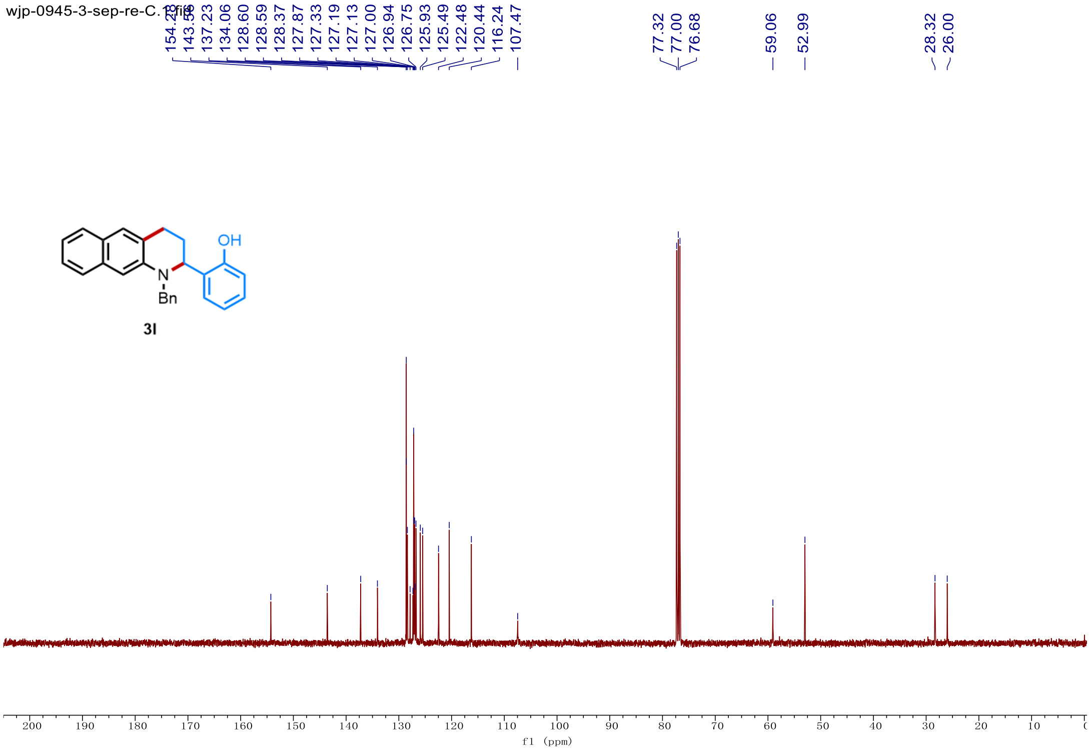

Supplementary Figure 93. ^{13}**C NMR (100 MHz, CDCl$_3$) spectrum of 3l.**

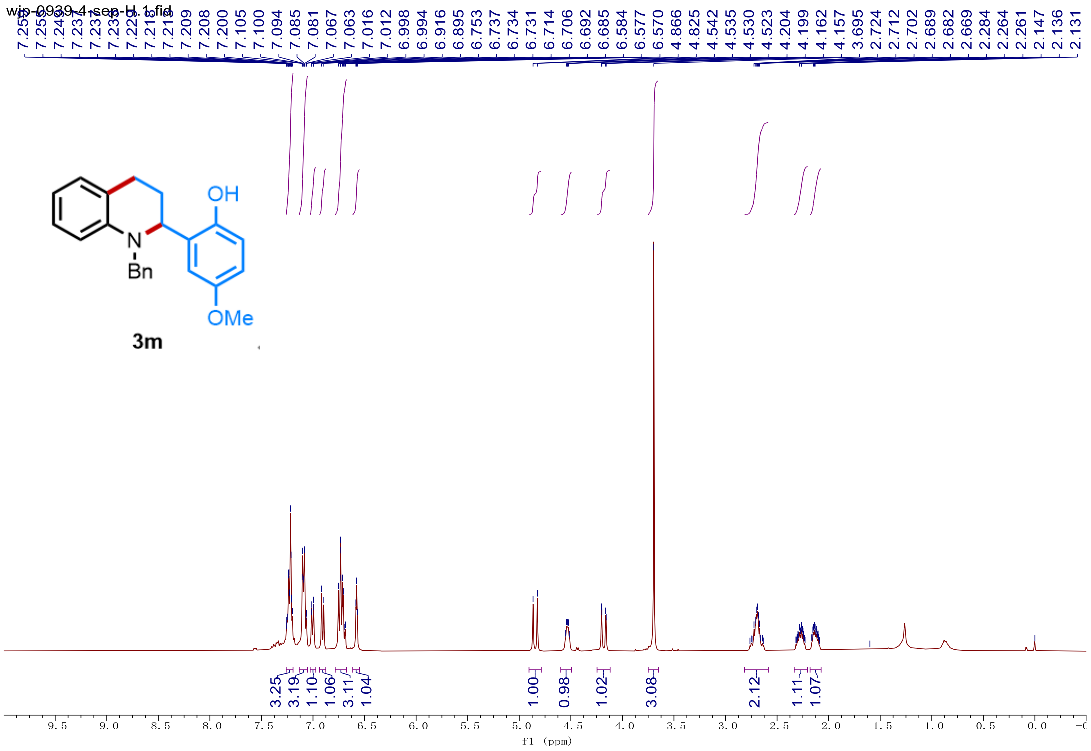

Supplementary Figure 94. ^{1}**H NMR (400 MHz, CDCl$_3$) spectrum of 3m.**

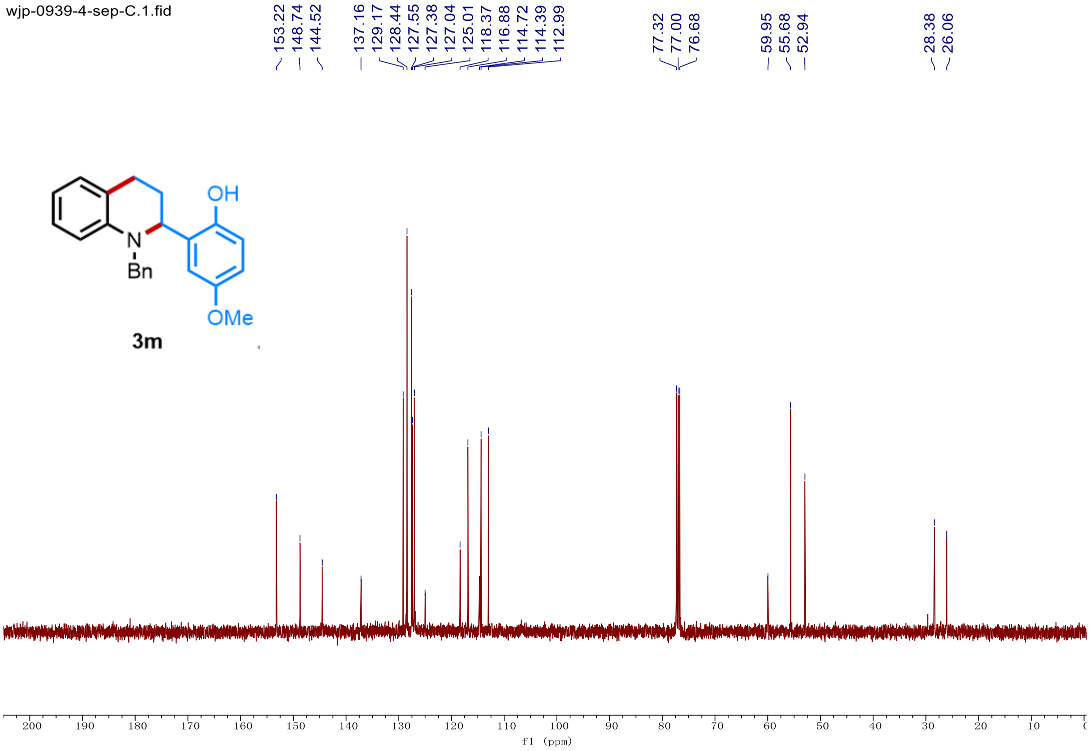

Supplementary Figure 95. ^{13}C NMR (100 MHz, CDCl$_3$) spectrum of 3m.

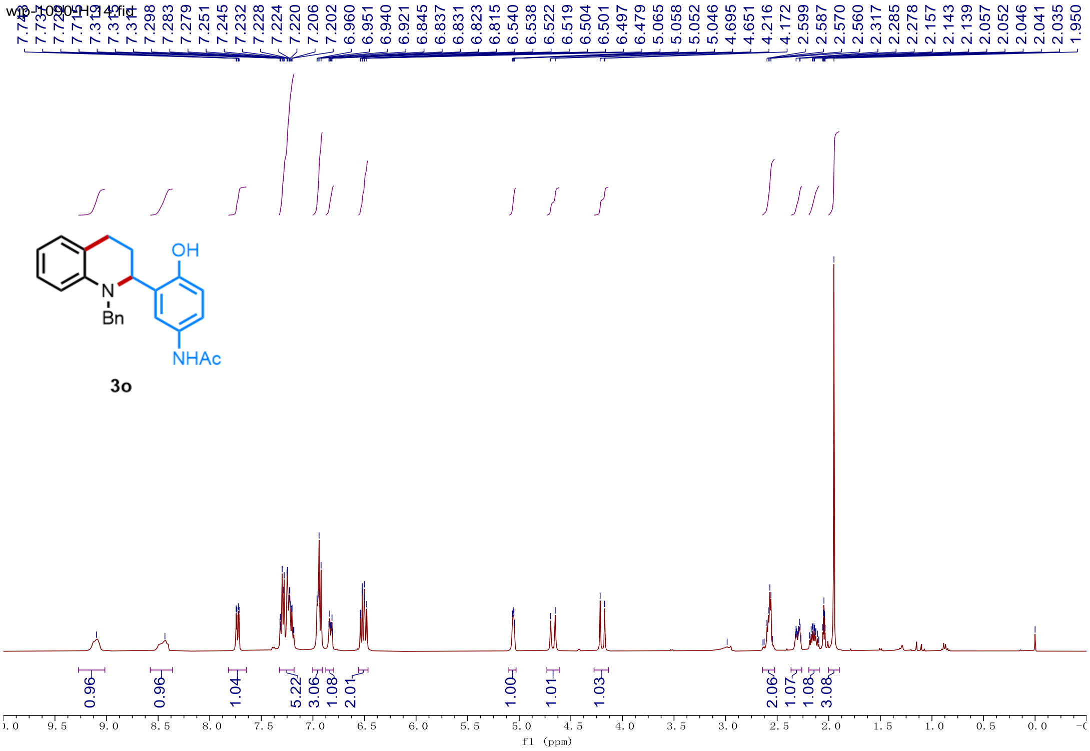

Supplementary Figure 96. ^{1}H NMR (400 MHz, CDCl$_3$) spectrum of 3o.

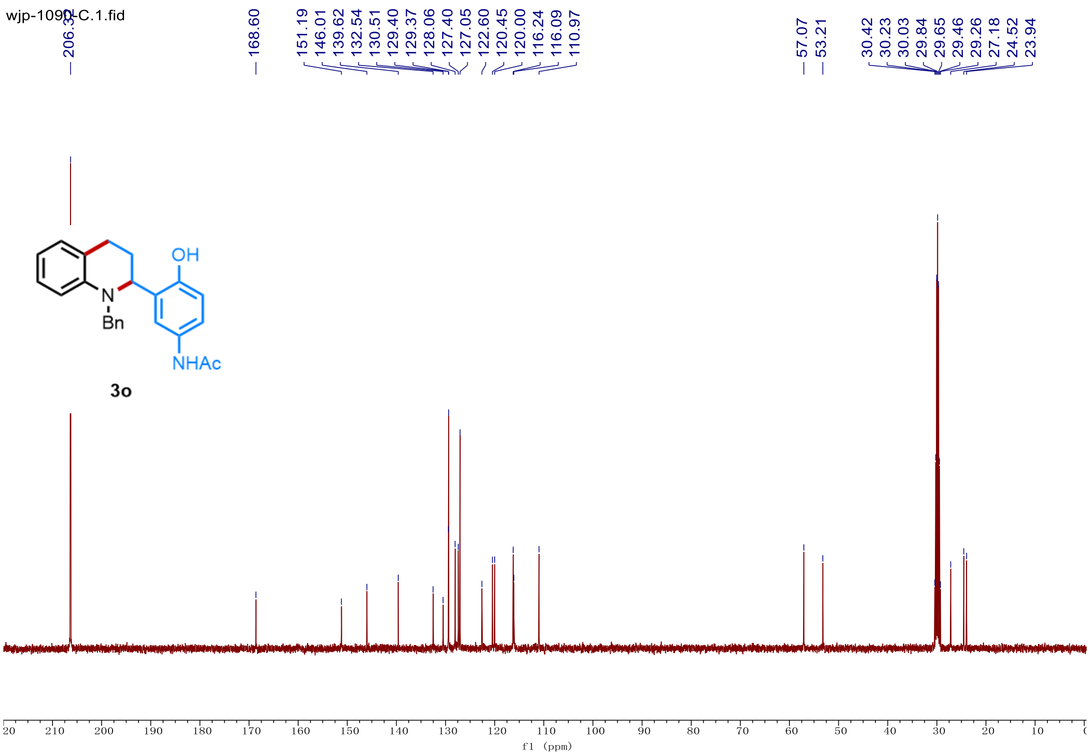

Supplementary Figure 97. ^{13}C NMR (100 MHz, $CDCl_3$) spectrum of 3o.

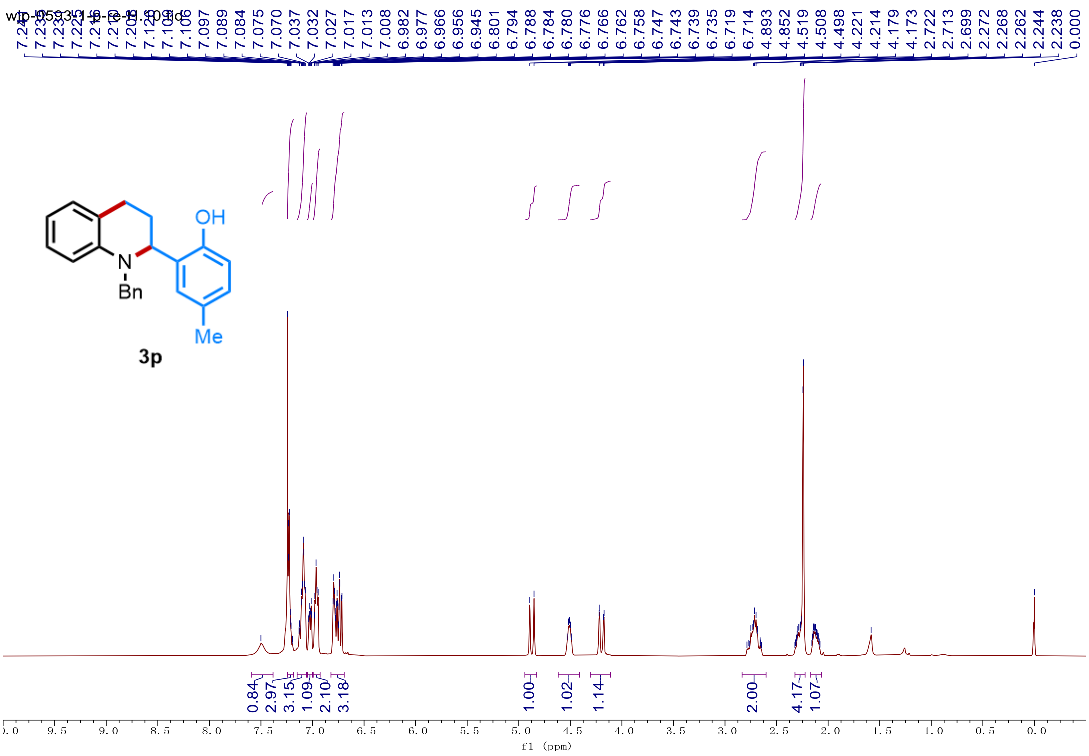

Supplementary Figure 98. ^{1}H NMR (400 MHz, $CDCl_3$) spectrum of 3p.

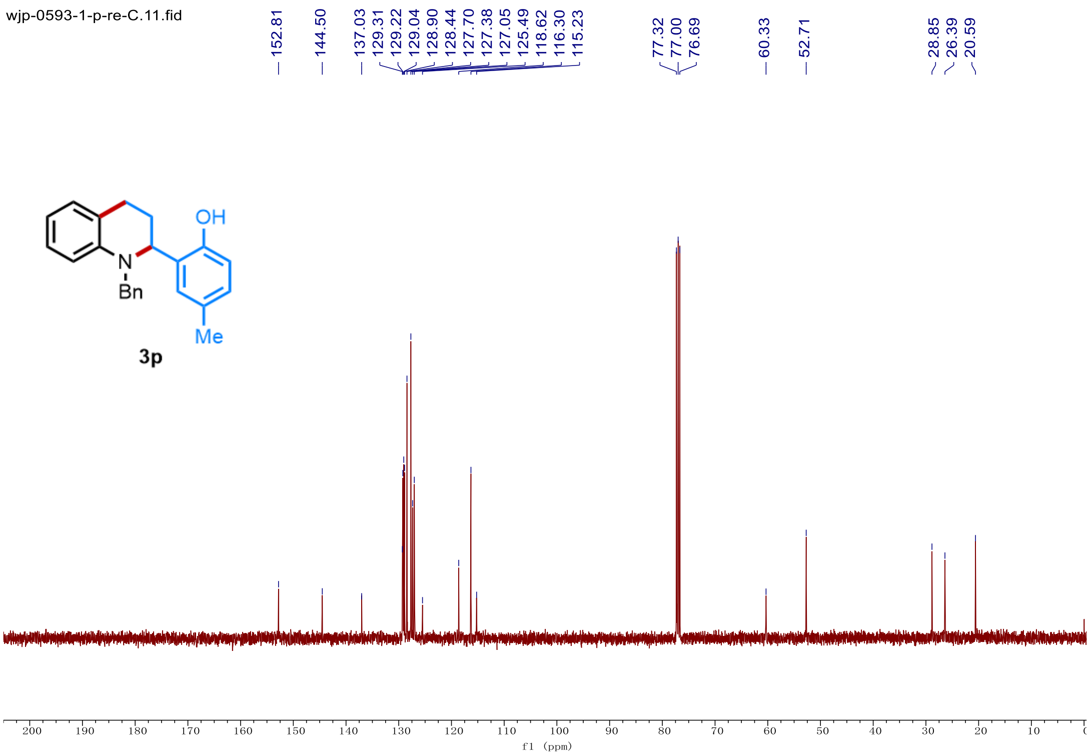

Supplementary Figure 99. ^{13}C NMR (100 MHz, $CDCl_3$) spectrum of 3p.

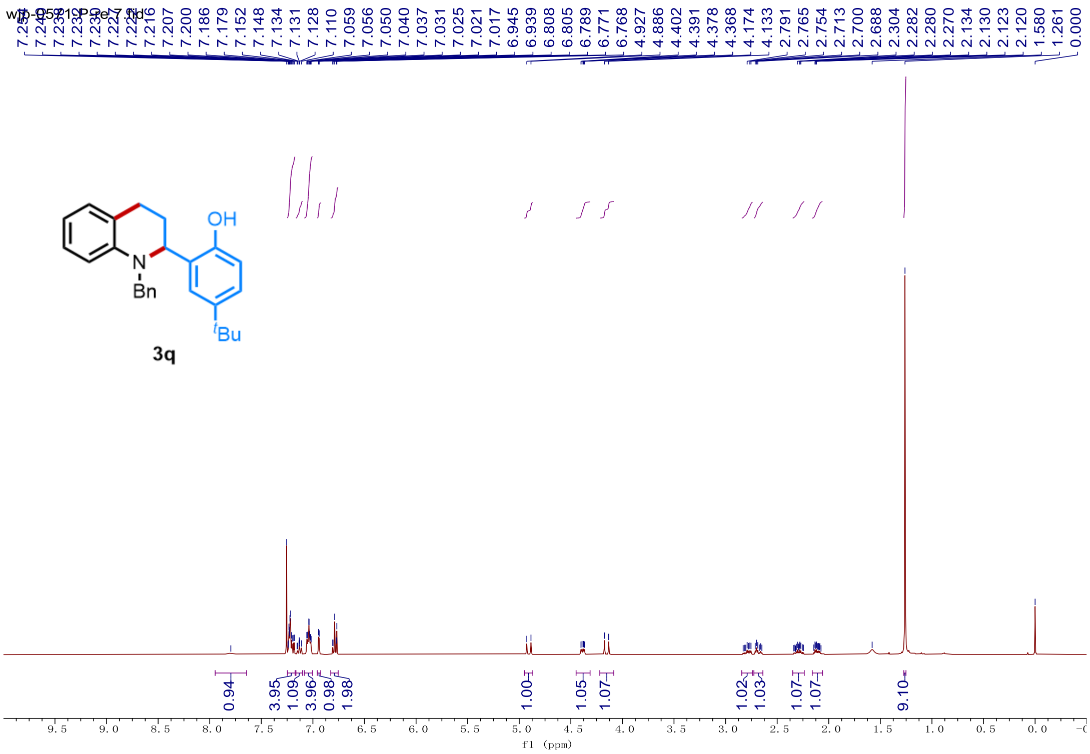

Supplementary Figure 100. ^{1}H NMR (400 MHz, $CDCl_3$) spectrum of 3q.

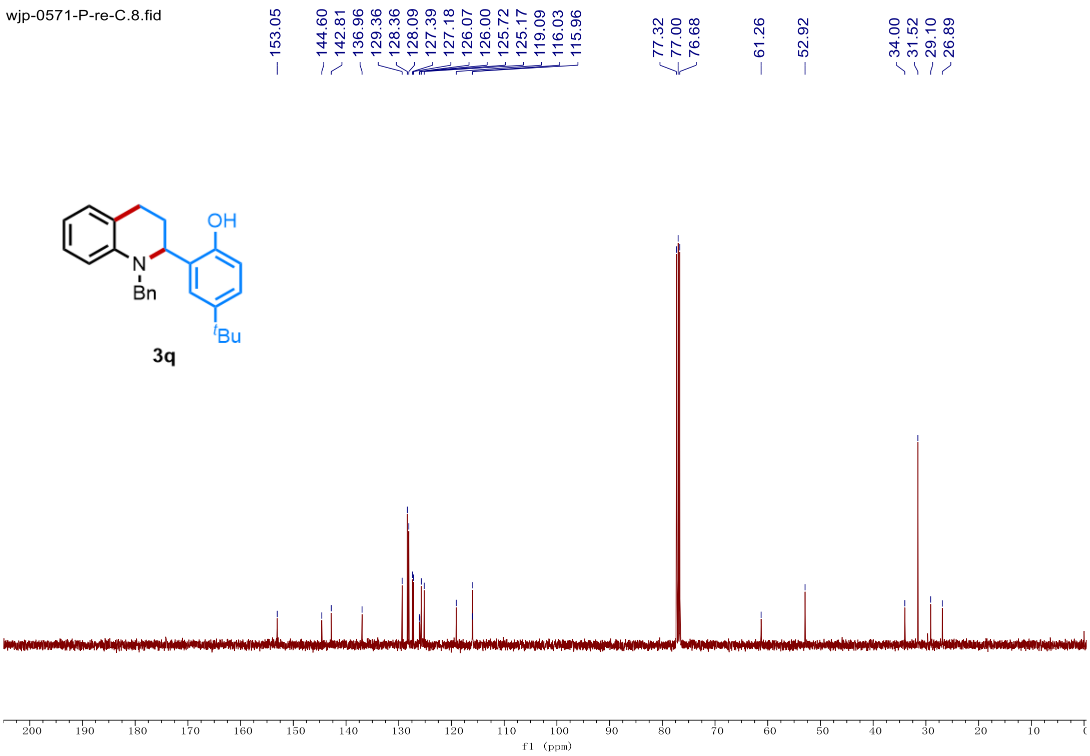

Supplementary Figure 101. ^{13}**C NMR (100 MHz, CDCl$_3$) spectrum of 3q.**

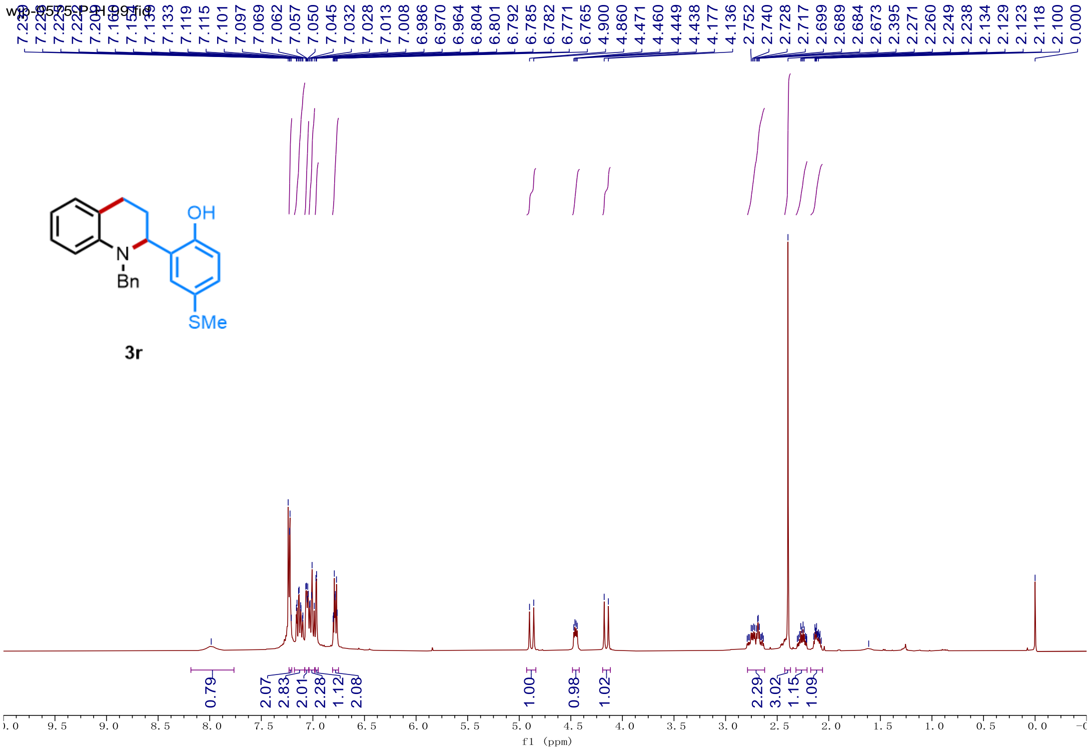

Supplementary Figure 102. ^{1}**H NMR (400 MHz, CDCl$_3$) spectrum of 3r.**

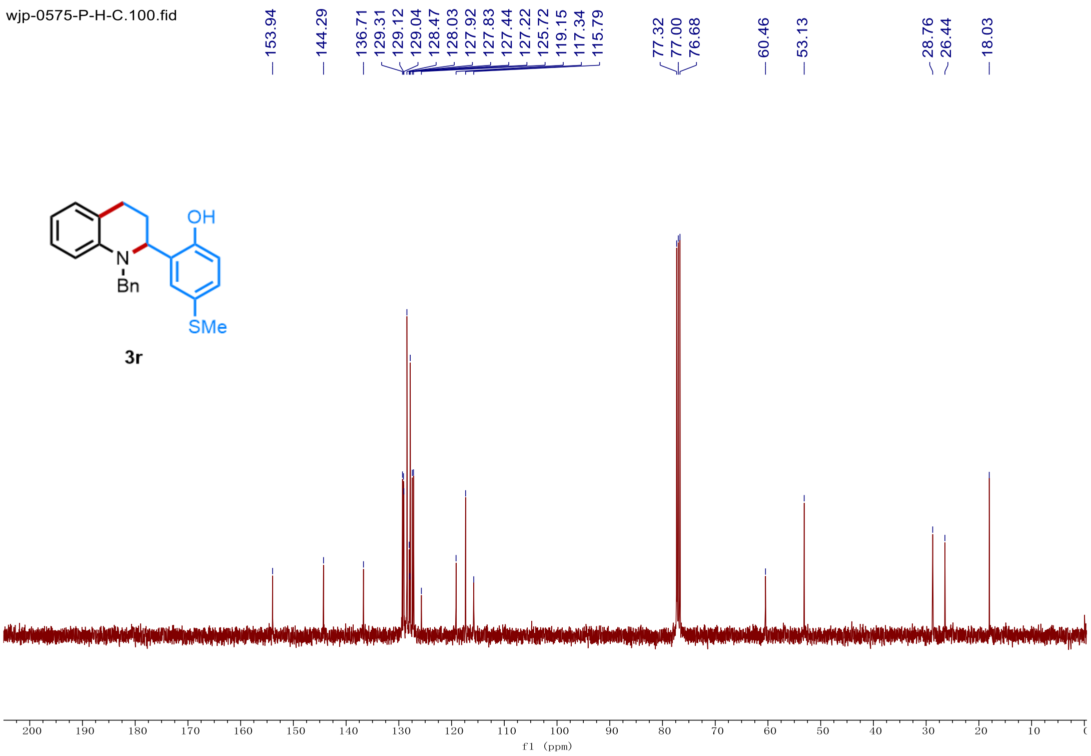

Supplementary Figure 103. ^{13}C NMR (100 MHz, $CDCl_3$) spectrum of 3r.

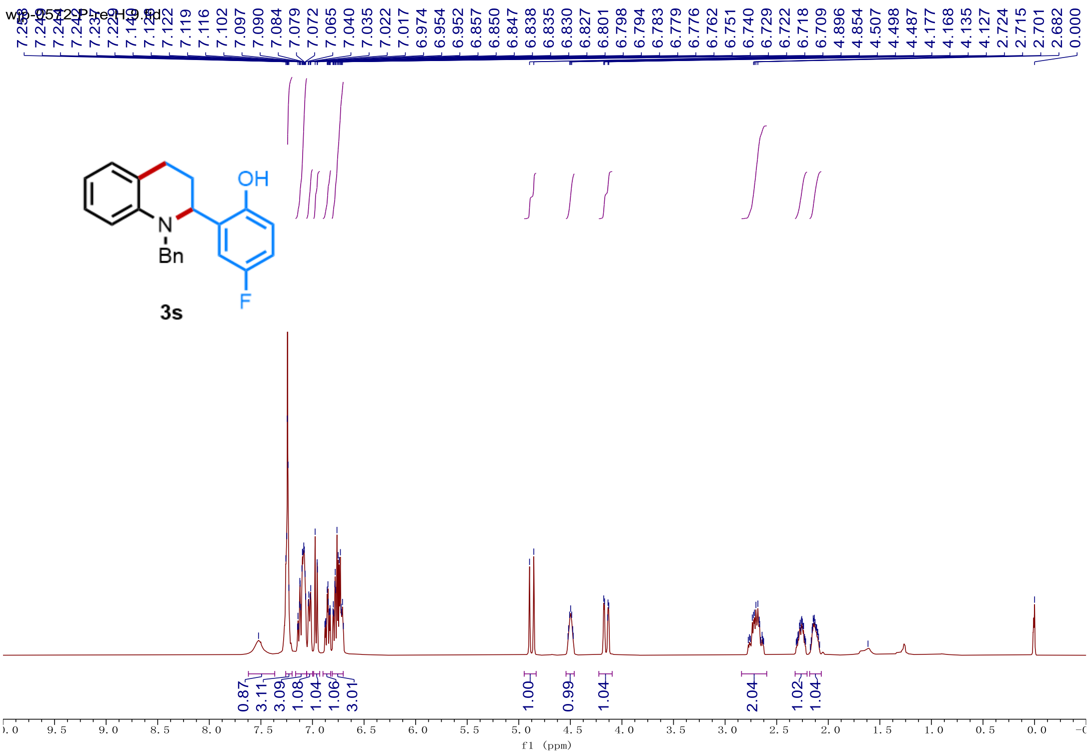

Supplementary Figure 104. ^{1}H NMR (400 MHz, $CDCl_3$) spectrum of 3s.

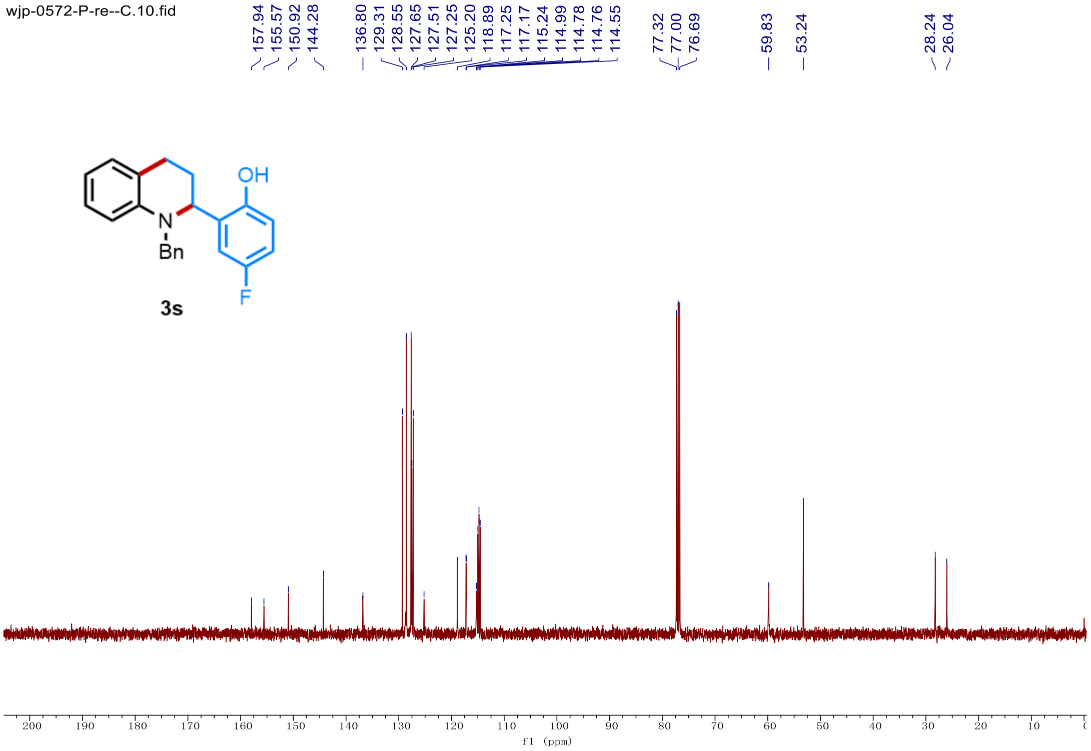

Supplementary Figure 105. ^{13}C **NMR (100 MHz,** $CDCl_3$**) spectrum of 3s.**

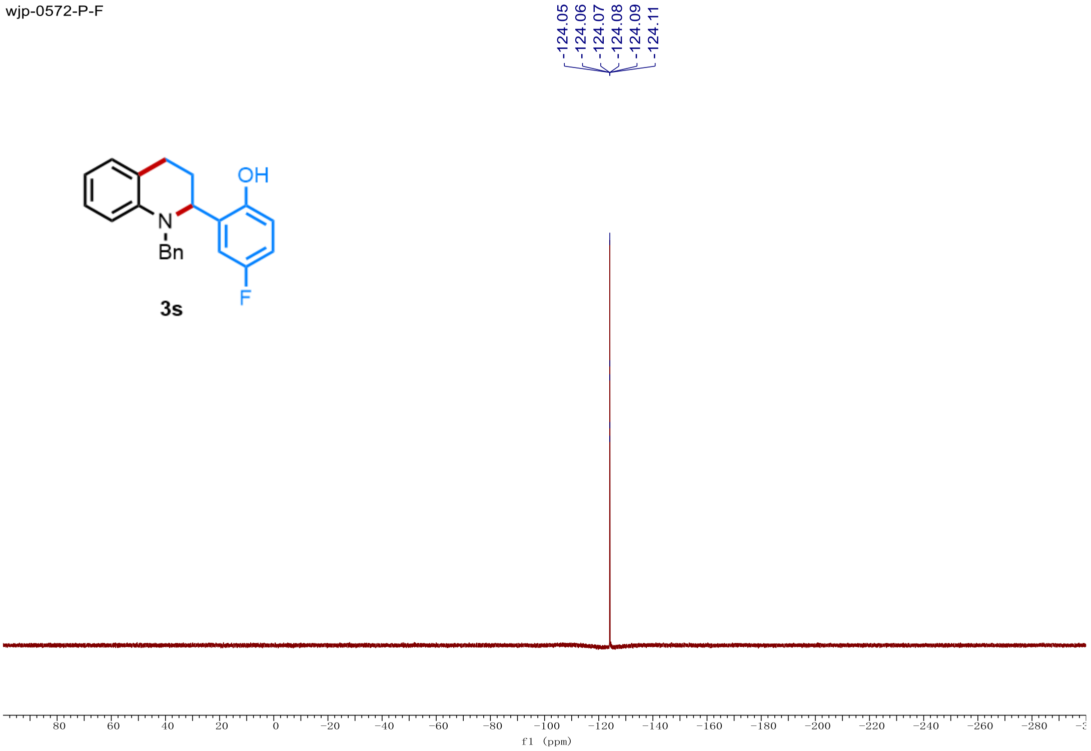

Supplementary

Figure 106. ^{19}F **NMR (375 MHz,** $CDCl_3$**) spectrum of 3s.**

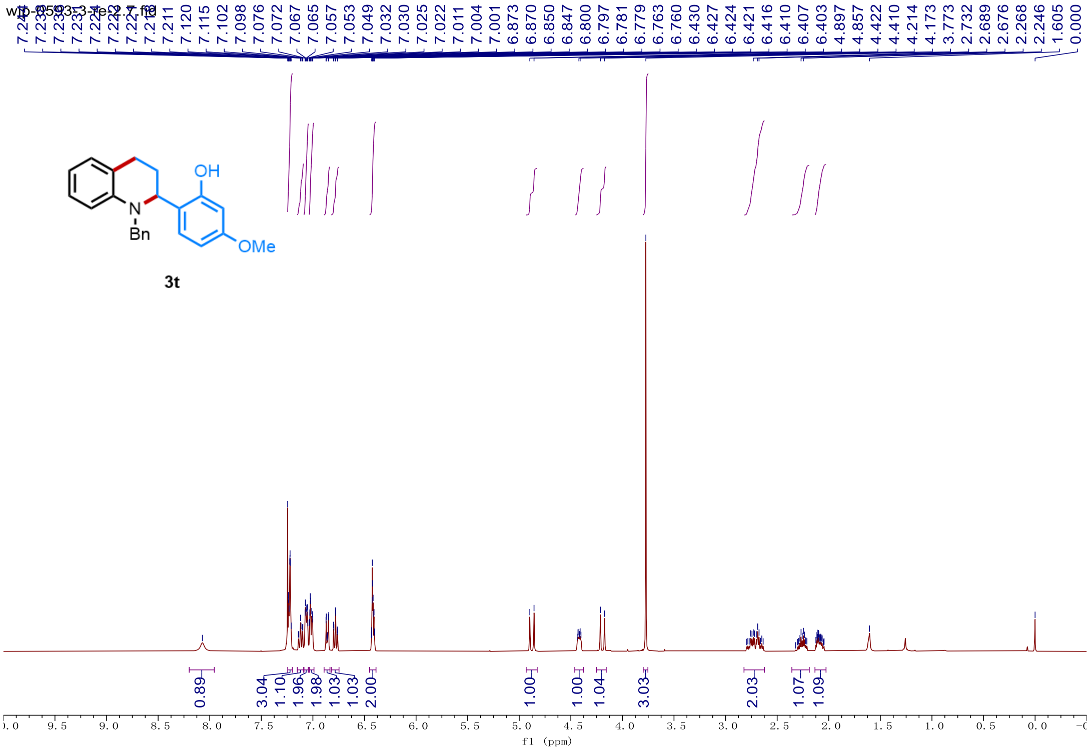

Supplementary Figure 107. ^{1}H NMR (400 MHz, $CDCl_3$) spectrum of 3t.

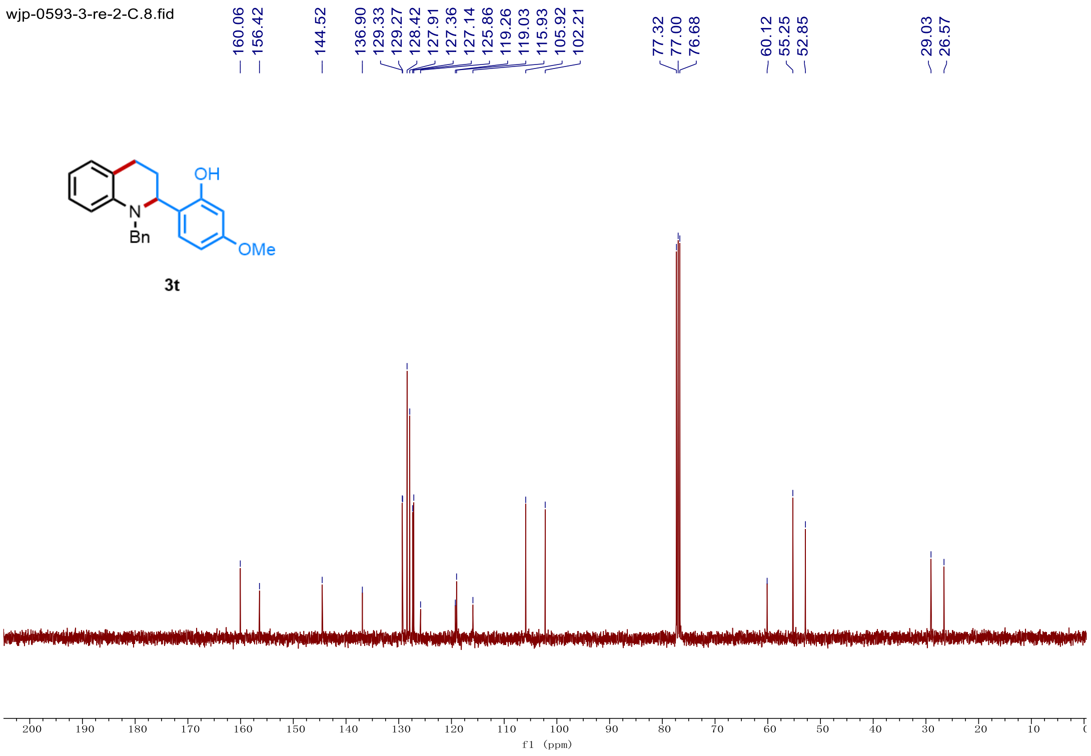

Supplementary Figure 108. ^{13}C NMR (100 MHz, $CDCl_3$) spectrum of 3t.

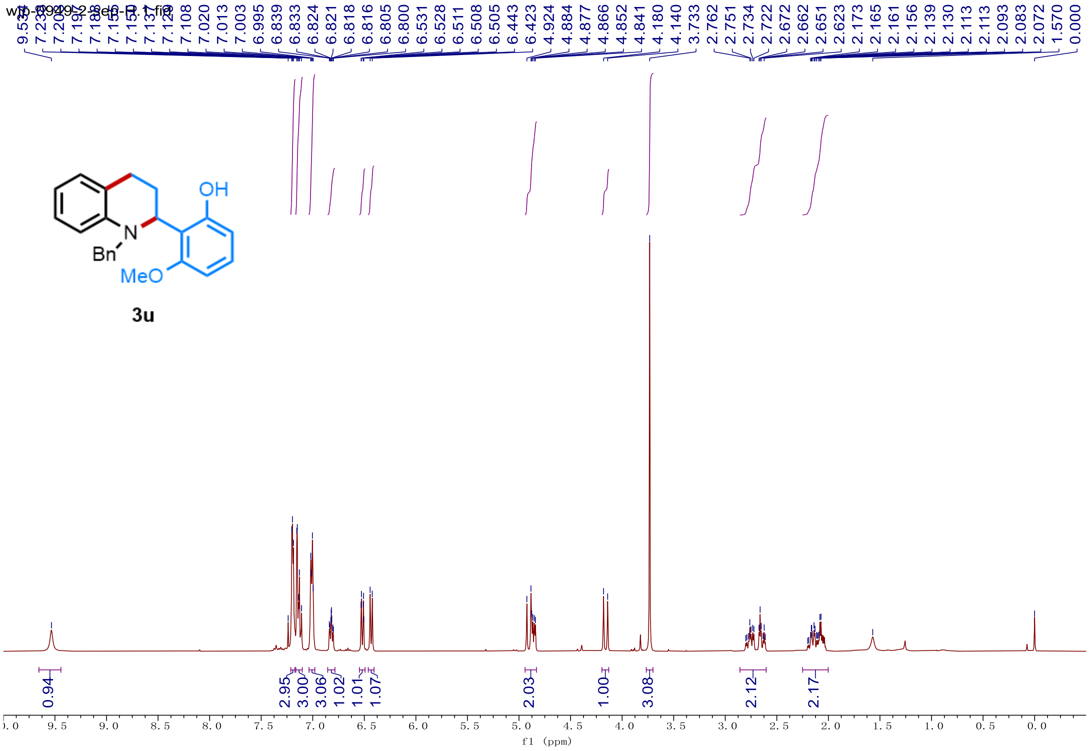

Supplementary Figure 109. **^{1}H NMR (400 MHz, $CDCl_3$) spectrum of 3u.**

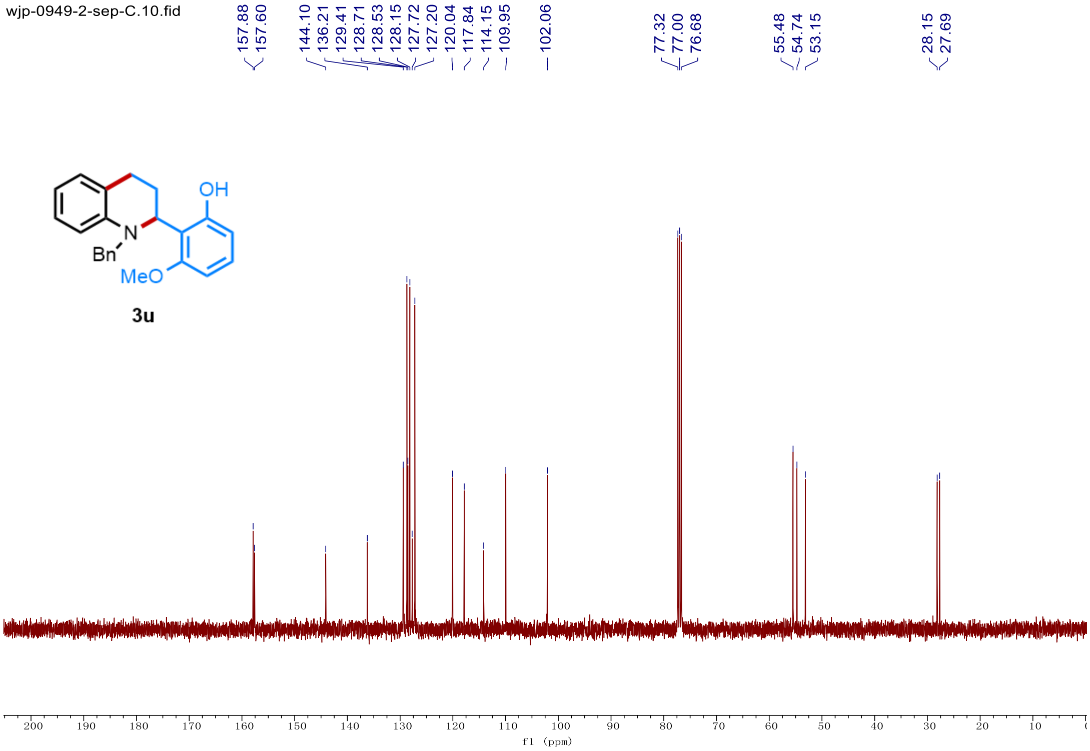

Supplementary Figure 110. **^{13}C NMR (100 MHz, $CDCl_3$) spectrum of 3u.**

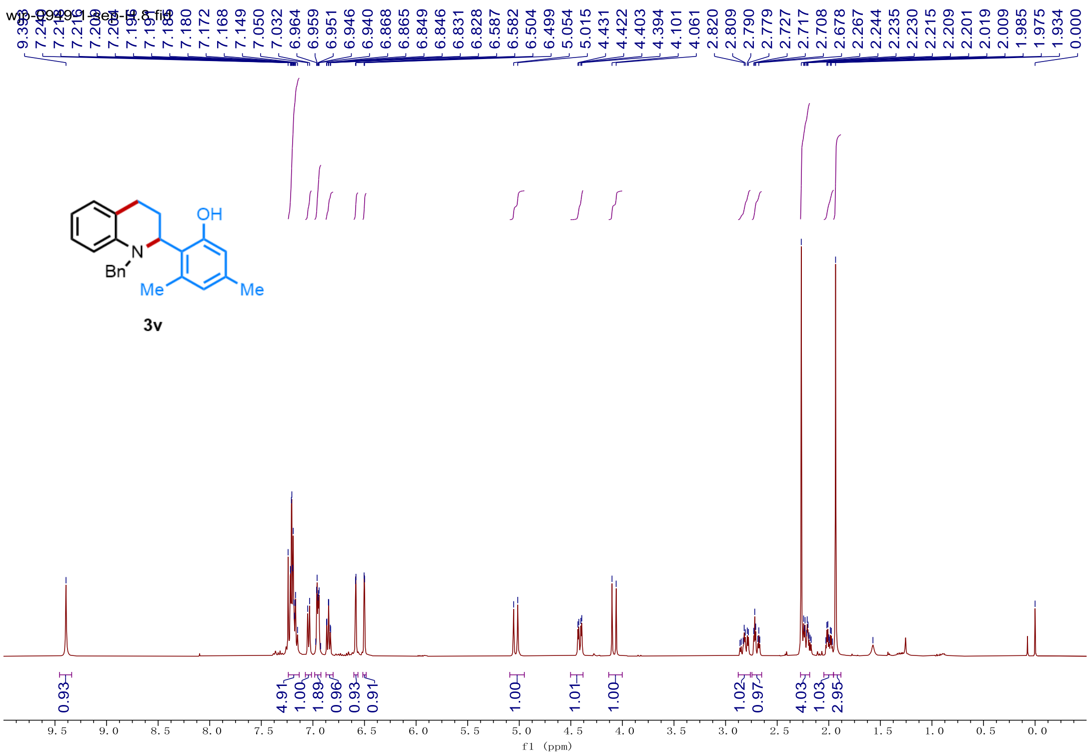

Supplementary Figure 111. ^{1}H NMR (400 MHz, $CDCl_3$) spectrum of 3v.

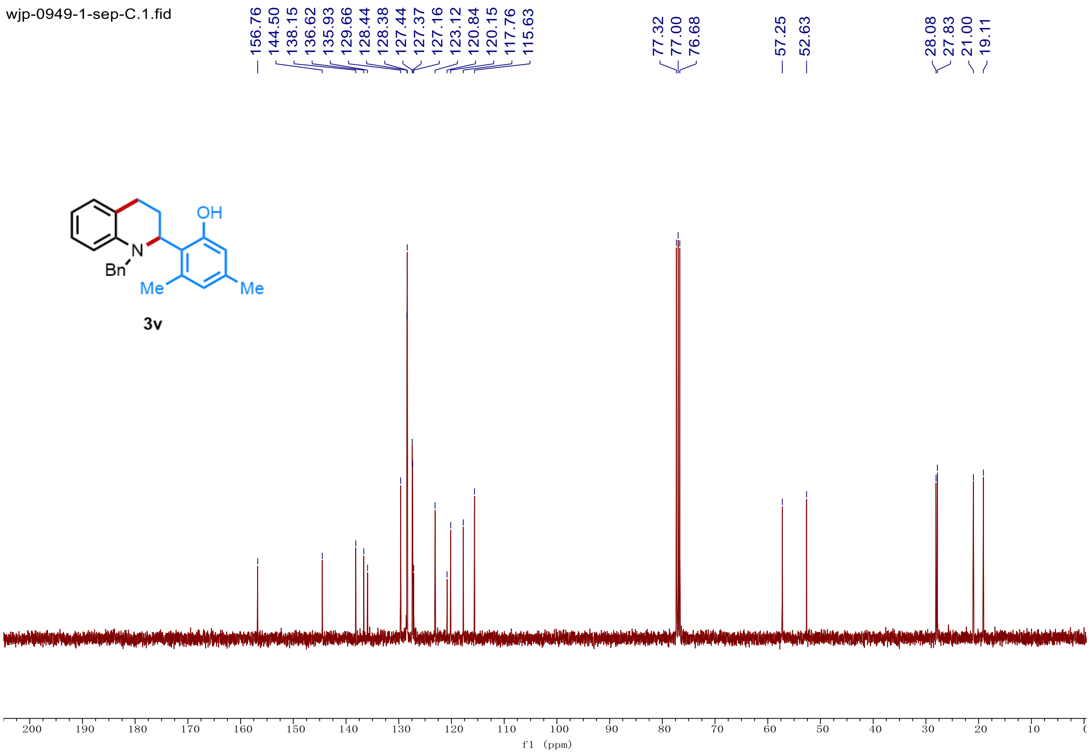

Supplementary Figure 112. ^{13}C NMR (100 MHz, $CDCl_3$) spectrum of 3v.

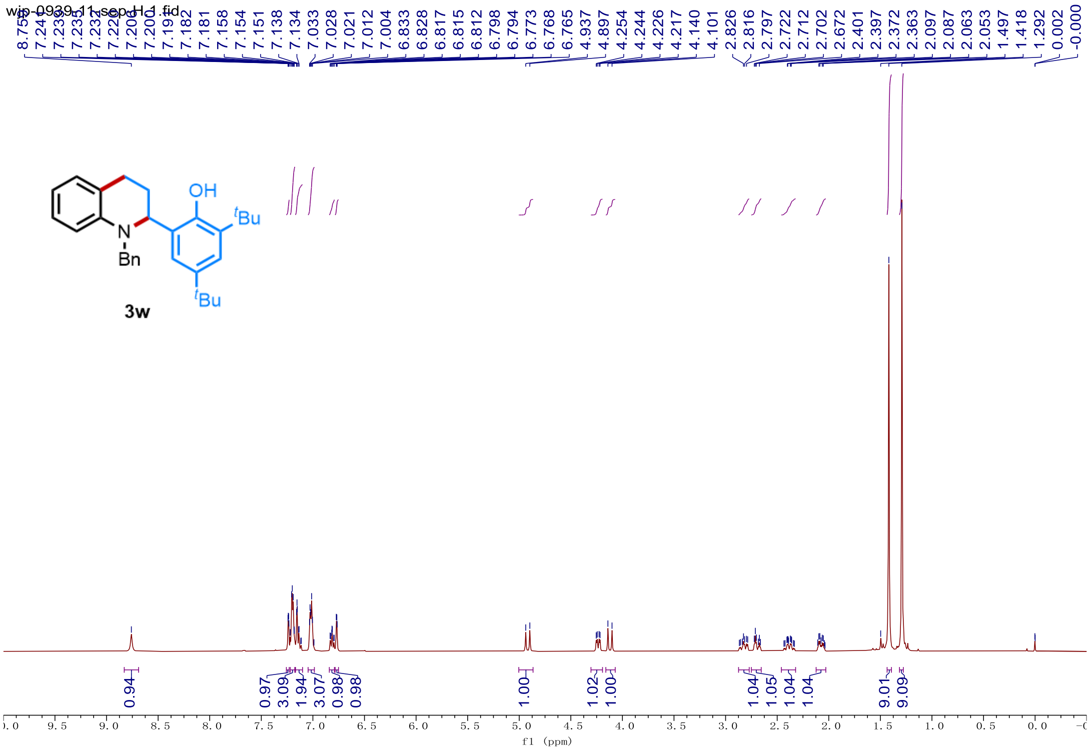

Supplementary Figure 113. ^{1}H NMR (400 MHz, CDCl$_3$) spectrum of 3w.

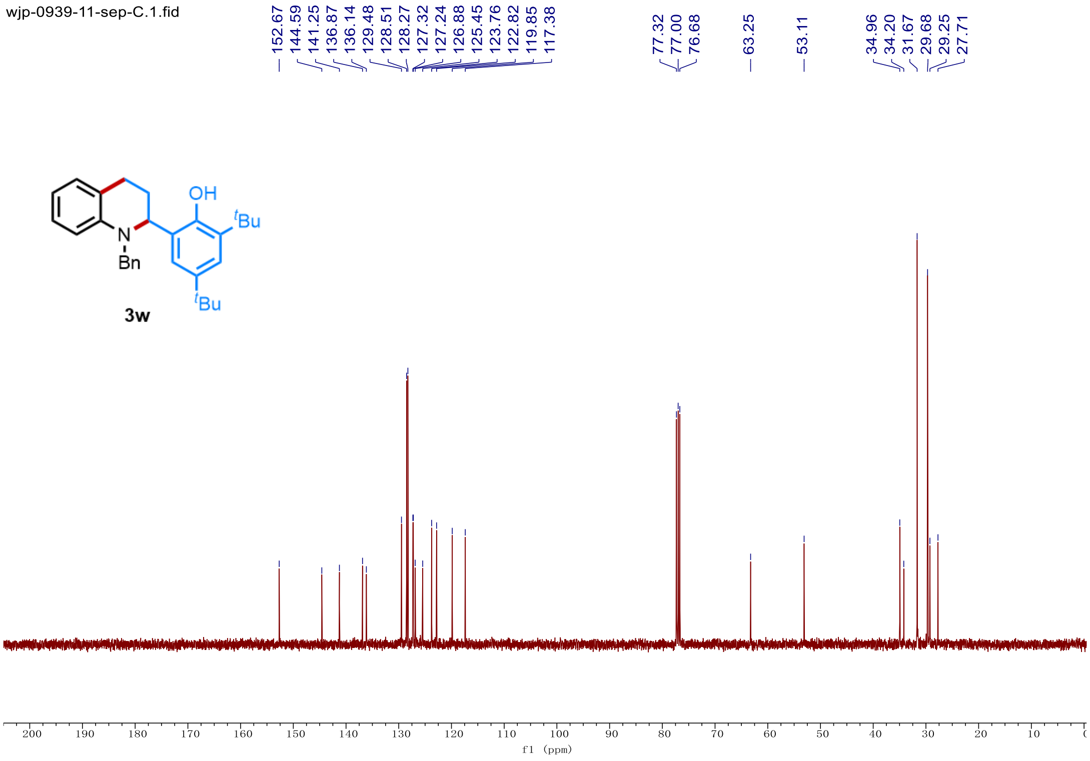

Supplementary Figure 114. ^{13}C NMR (100 MHz, CDCl$_3$) spectrum of 3w.

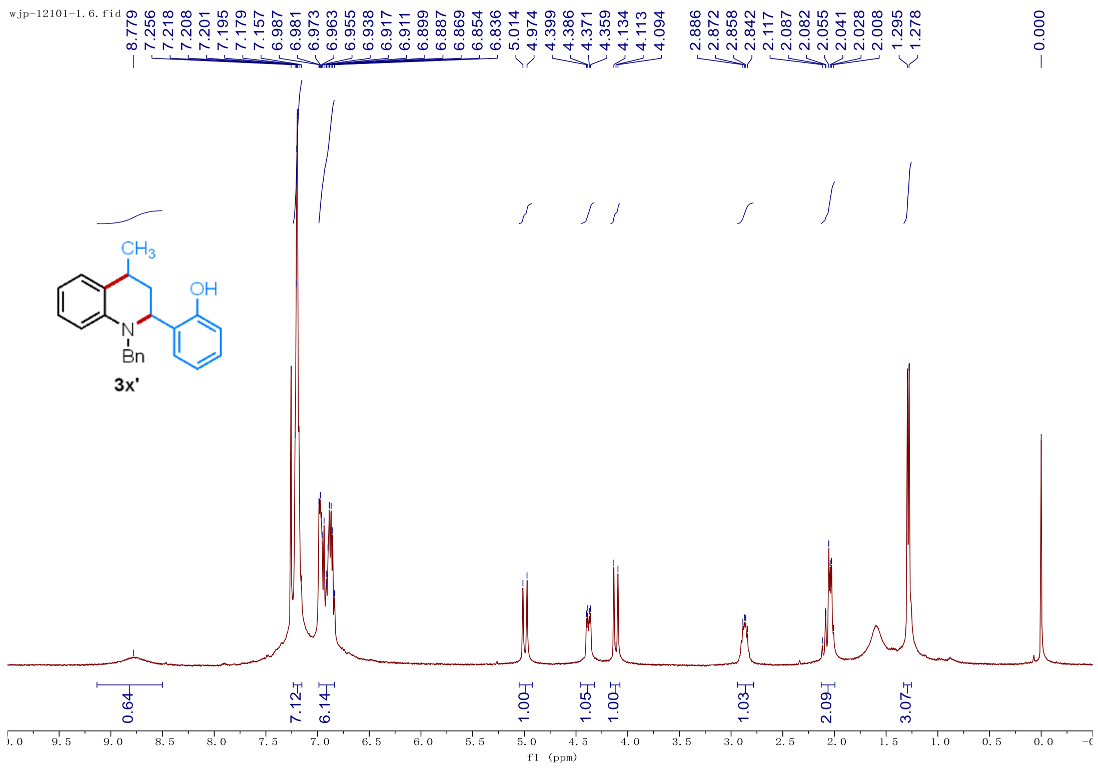

Supplementary Figure 115. ^{1}H NMR (400 MHz, $CDCl_3$) spectrum of 3x’.

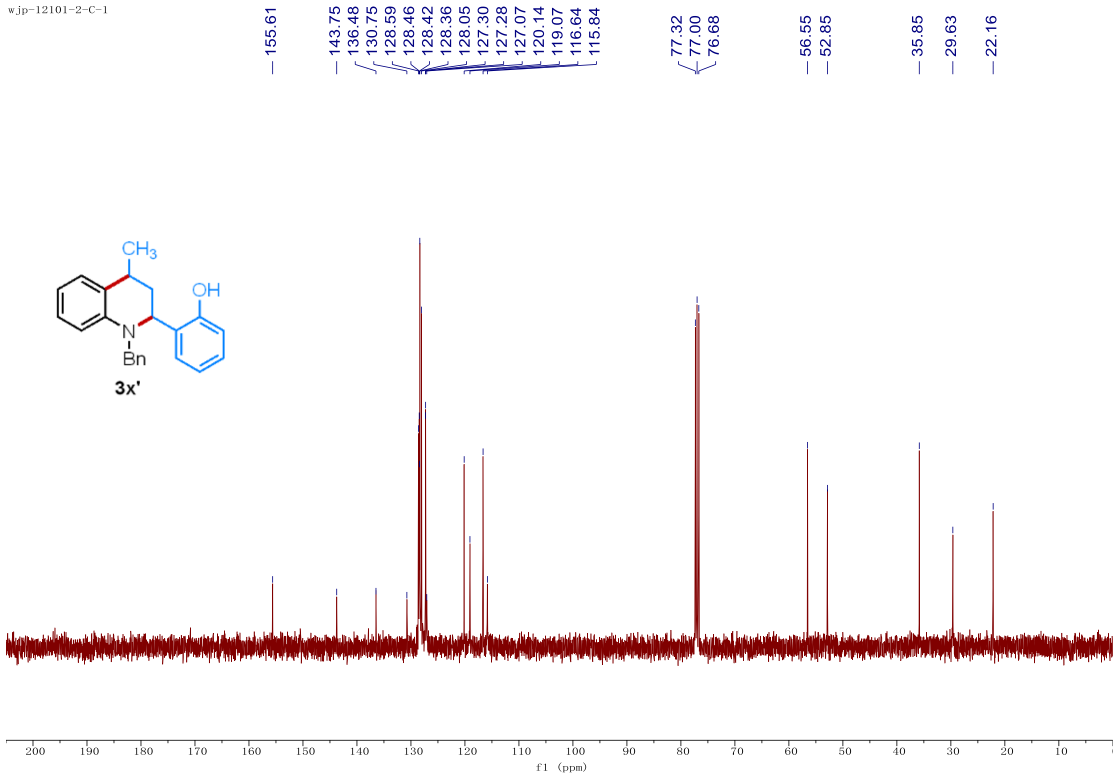

Supplementary Figure 116. ^{13}C NMR (100 MHz, $CDCl_3$) spectrum of 3x’.

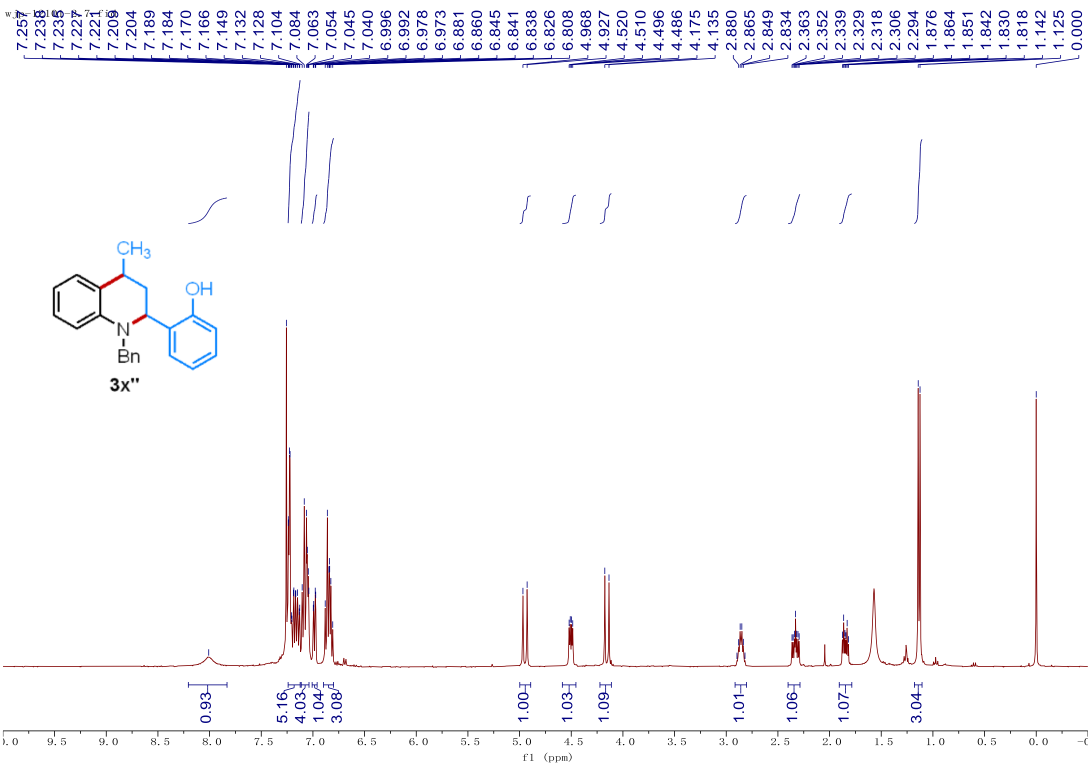

Supplementary Figure 117. ^{1}**H NMR (400 MHz, CDCl$_3$) spectrum of 3x".**

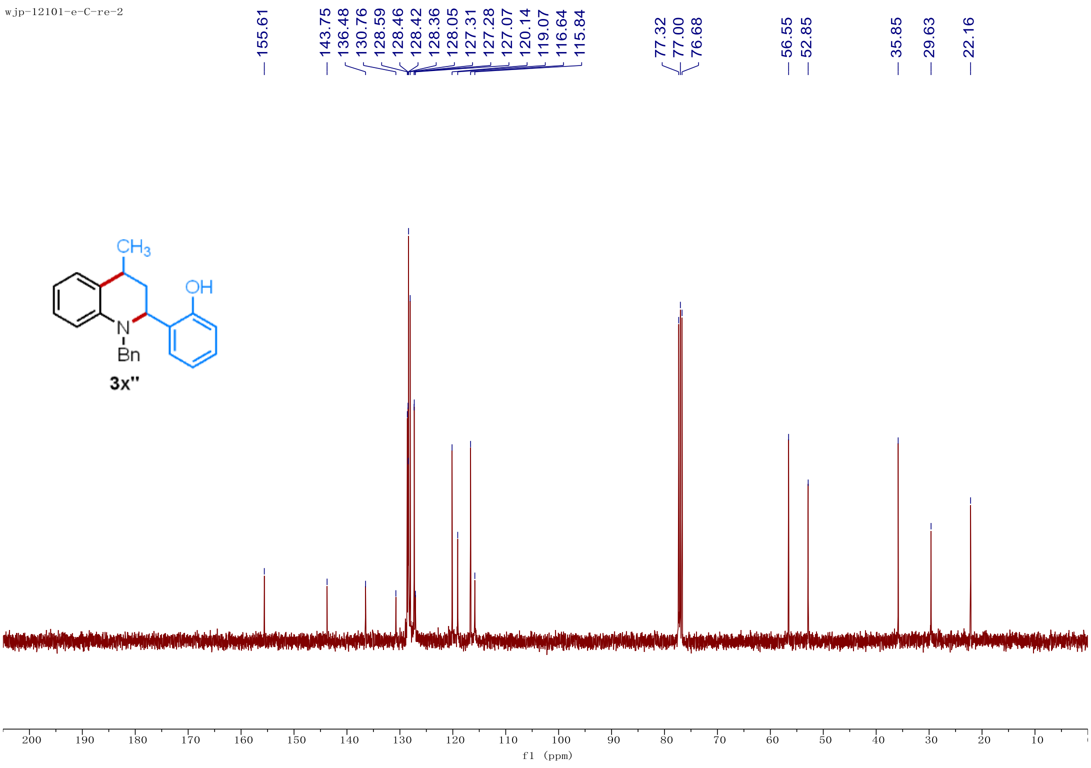

Supplementary Figure 118. ^{13}**C NMR (100 MHz, CDCl$_3$) spectrum of 3x".**

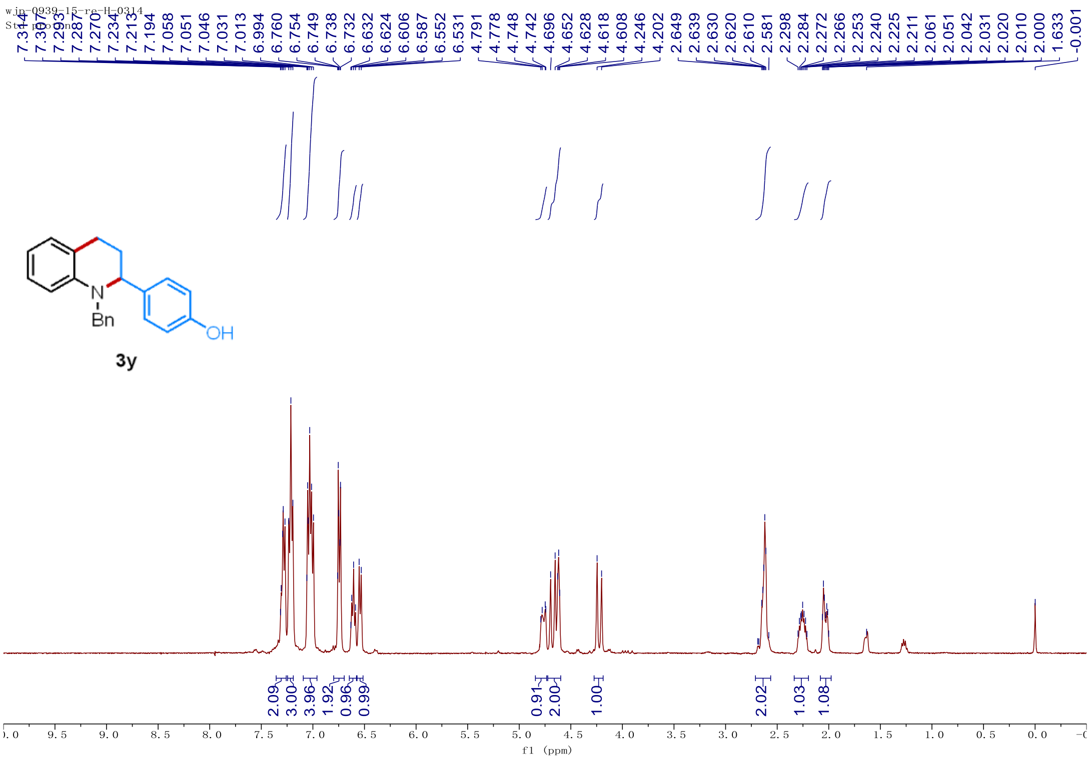

Supplementary Figure 119. ^{1}H NMR (400 MHz, $CDCl_3$) spectrum of 3y.

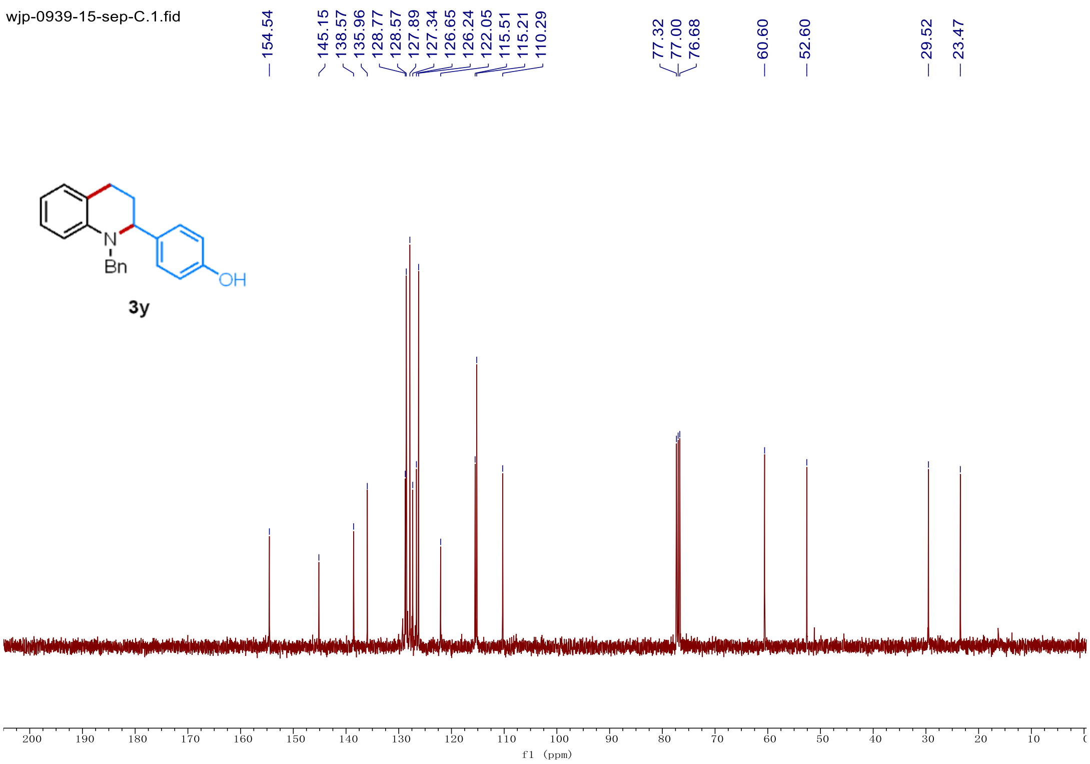

Supplementary Figure 120. ^{13}C NMR (100 MHz, $CDCl_3$) spectrum of 3y.

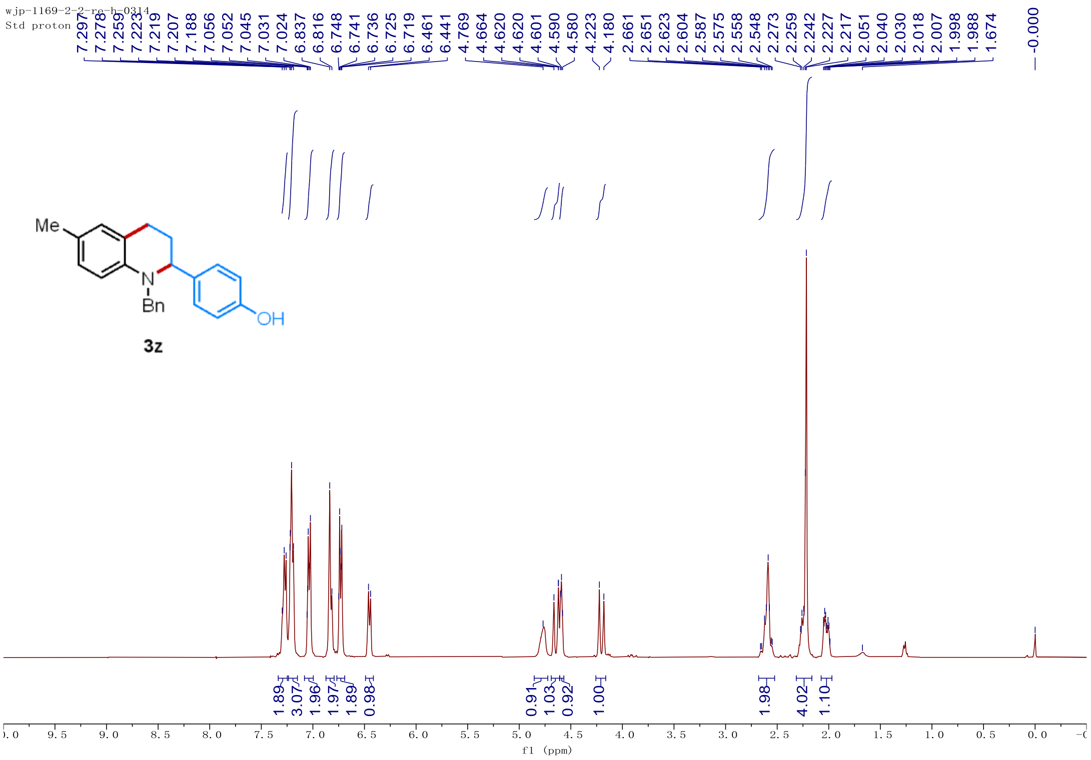

Supplementary Figure 121. ^{1}H NMR (400 MHz, $CDCl_3$) spectrum of 3z.

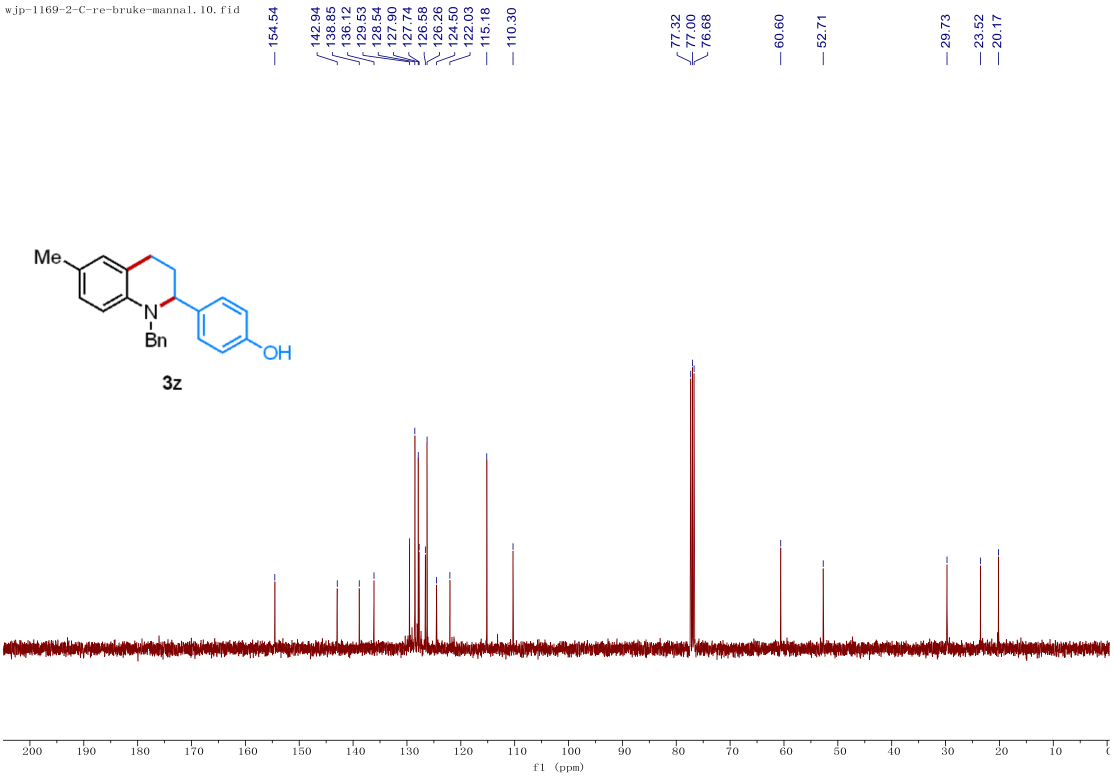

Supplementary Figure 122. ^{13}C NMR (100 MHz, $CDCl_3$) spectrum of 3z.

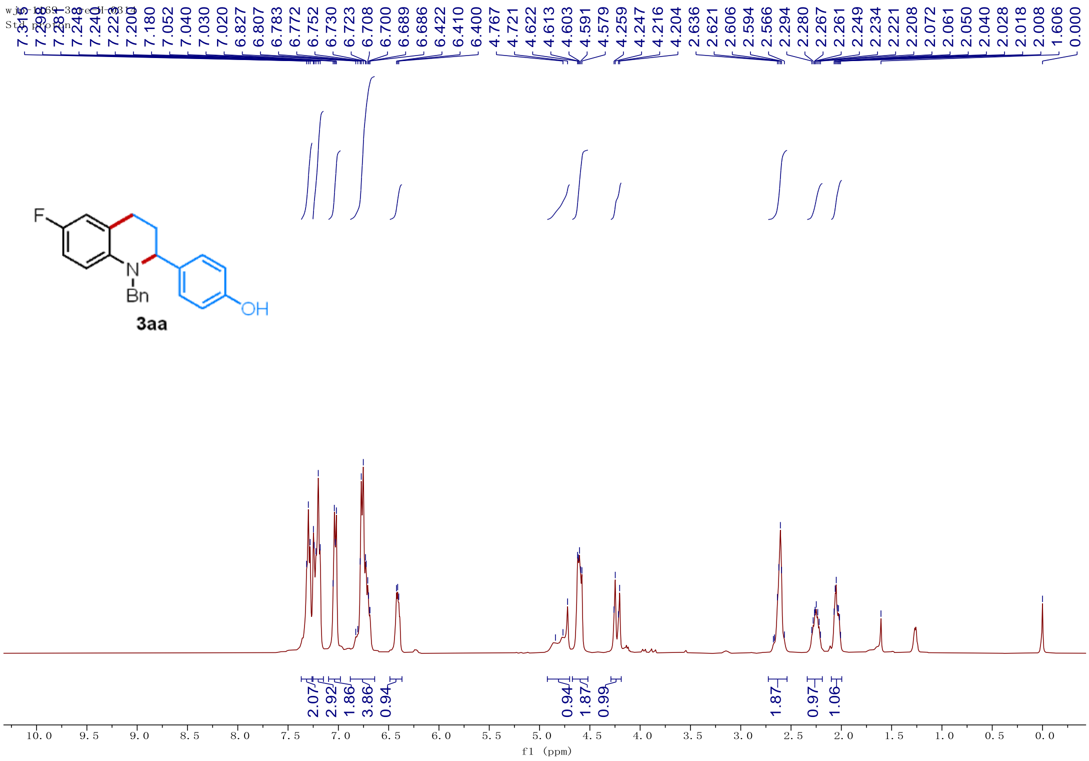

Supplementary Figure 123. ^{1}H NMR (400 MHz, $CDCl_3$) spectrum of 3aa.

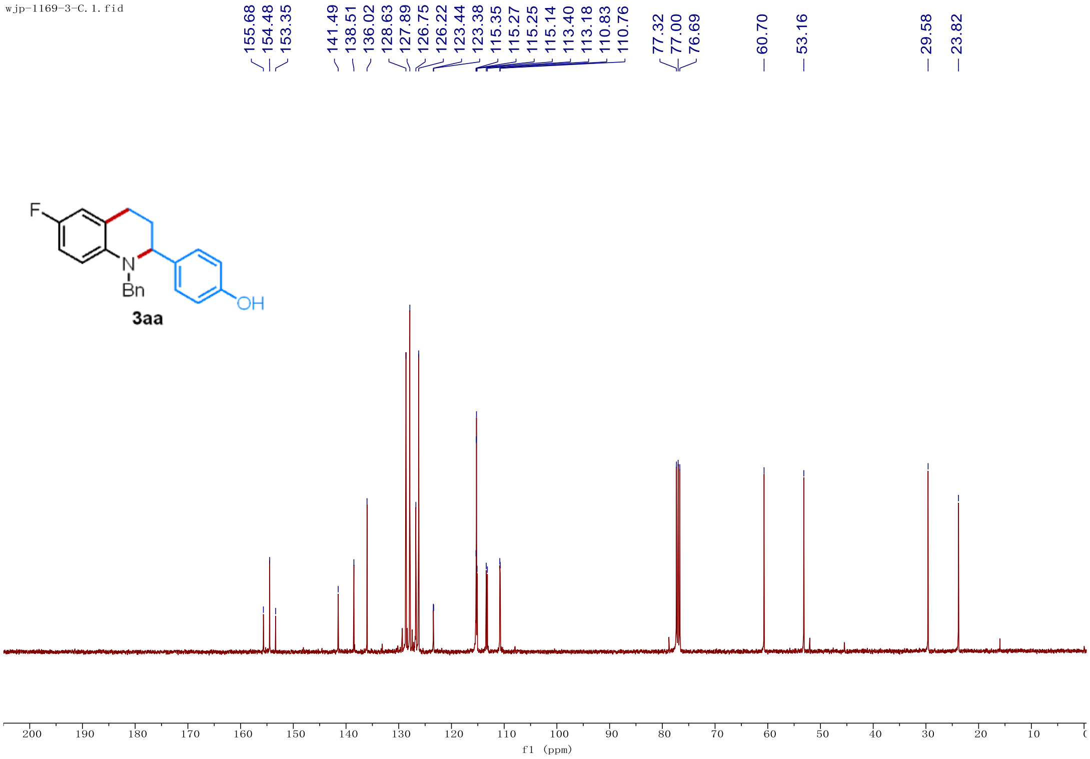

Supplementary Figure 124. ^{13}C NMR (100 MHz, $CDCl_3$) spectrum of 3aa.

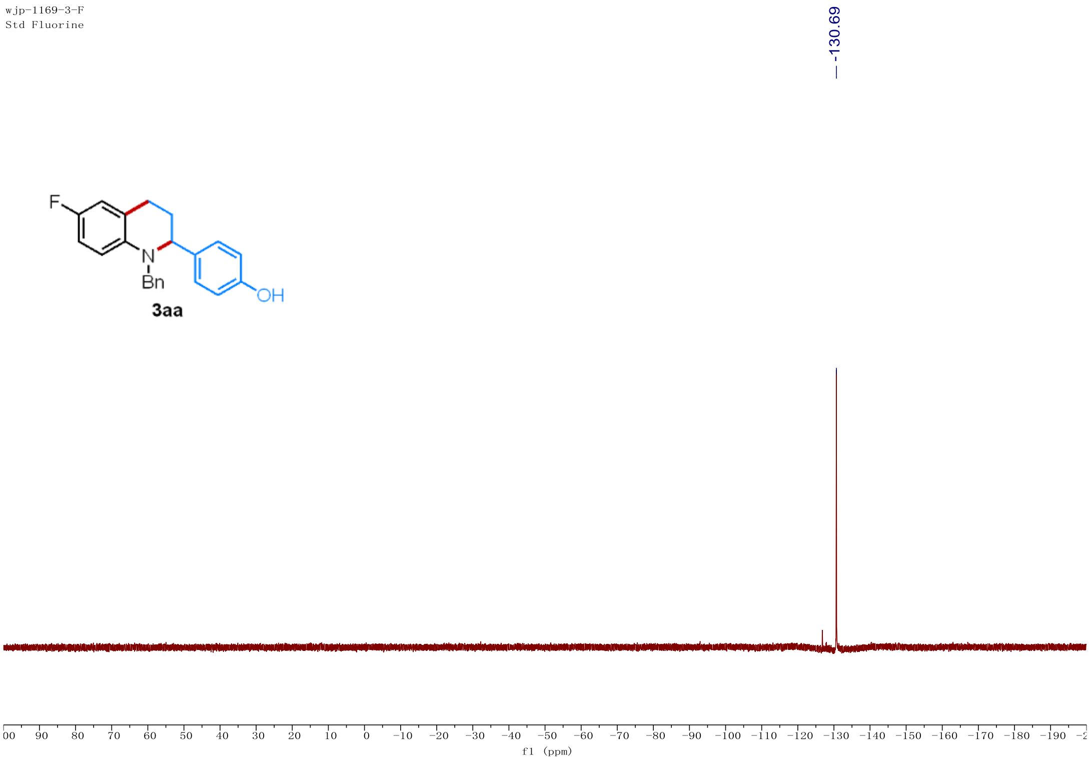

Supplementary Figure 125. ^{19}**F NMR (375 MHz, CDCl$_3$) spectrum of 3aa.**

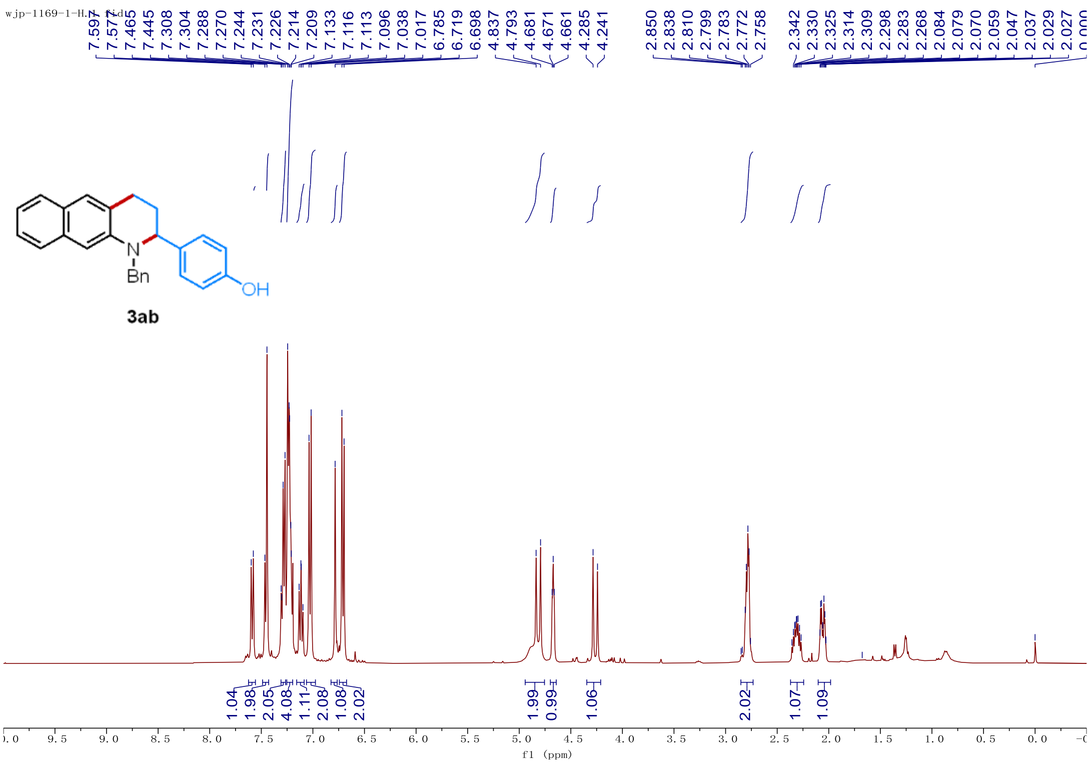

Supplementary Figure 126. ^{1}**H NMR (400 MHz, CDCl$_3$) spectrum of 3ab.**

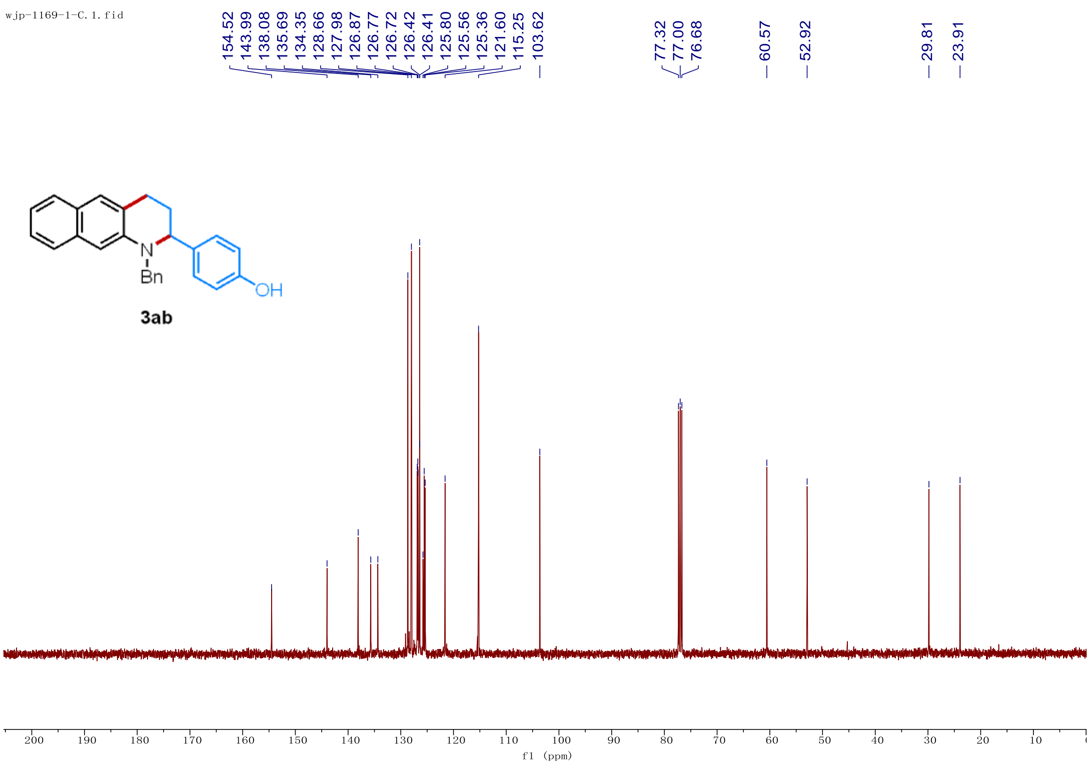

Supplementary Figure 127. ^{13}C NMR (100 MHz, $CDCl_3$) spectrum of 3ab.

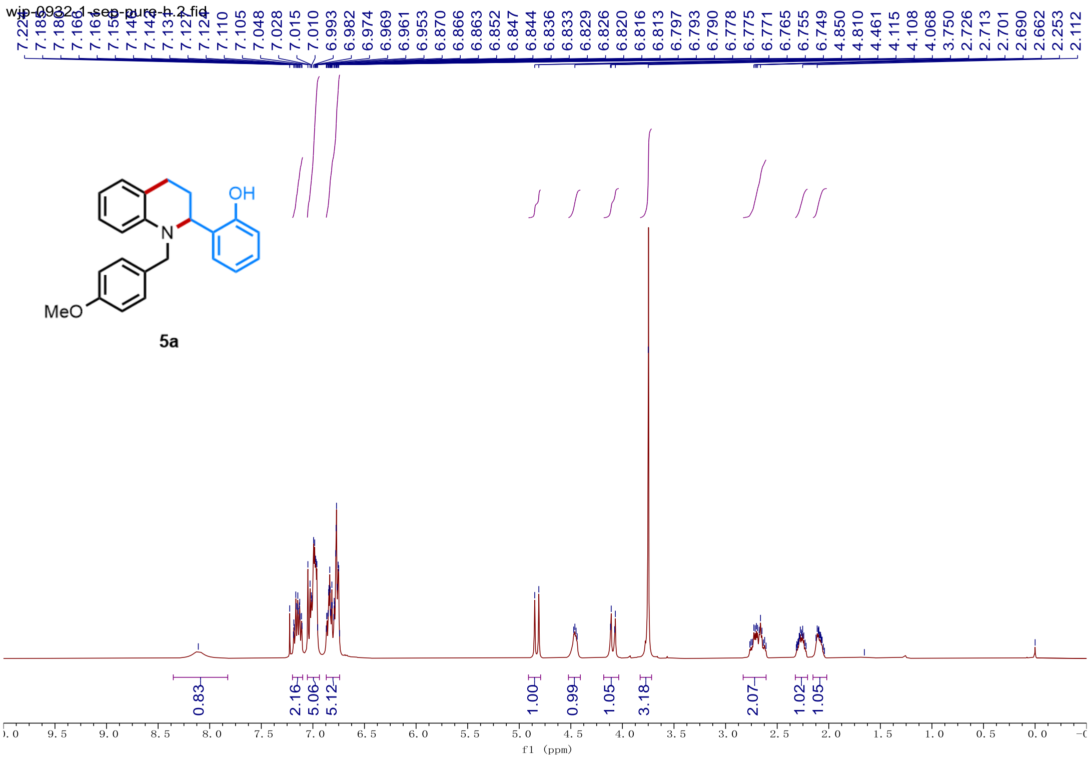

Supplementary Figure 128. ^{1}H NMR (400 MHz, $CDCl_3$) spectrum of 5a.

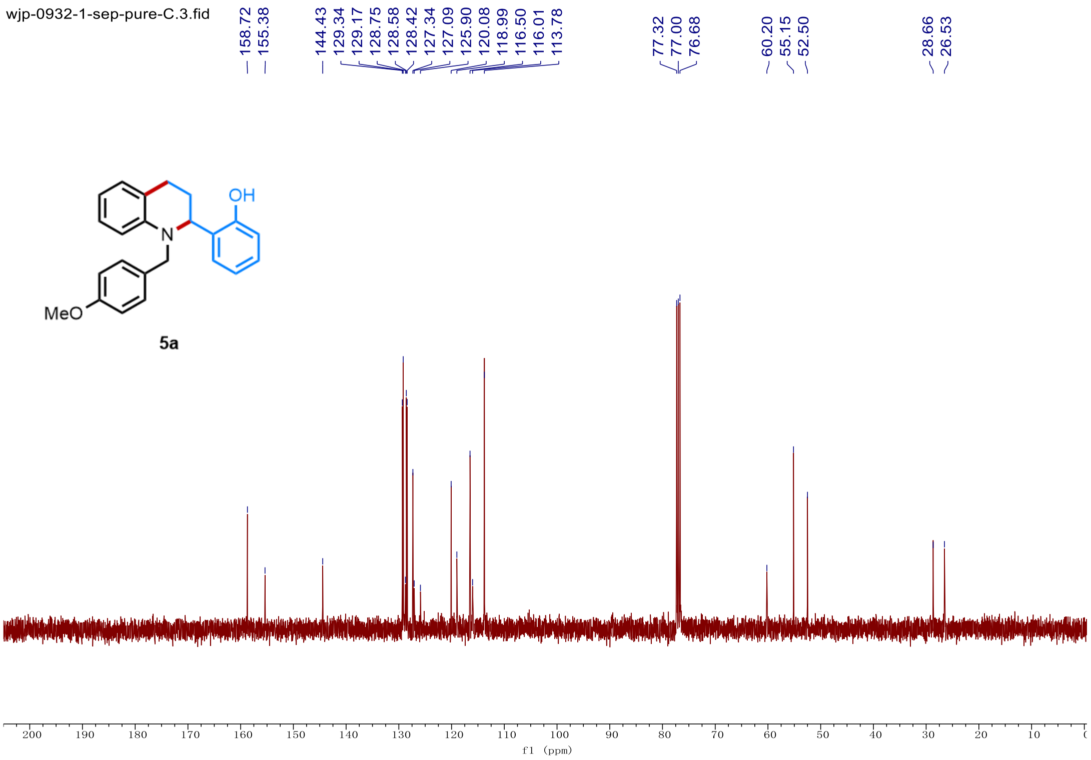

Supplementary Figure 129. ^{13}**C NMR (100 MHz, CDCl$_3$) spectrum of 5a.**

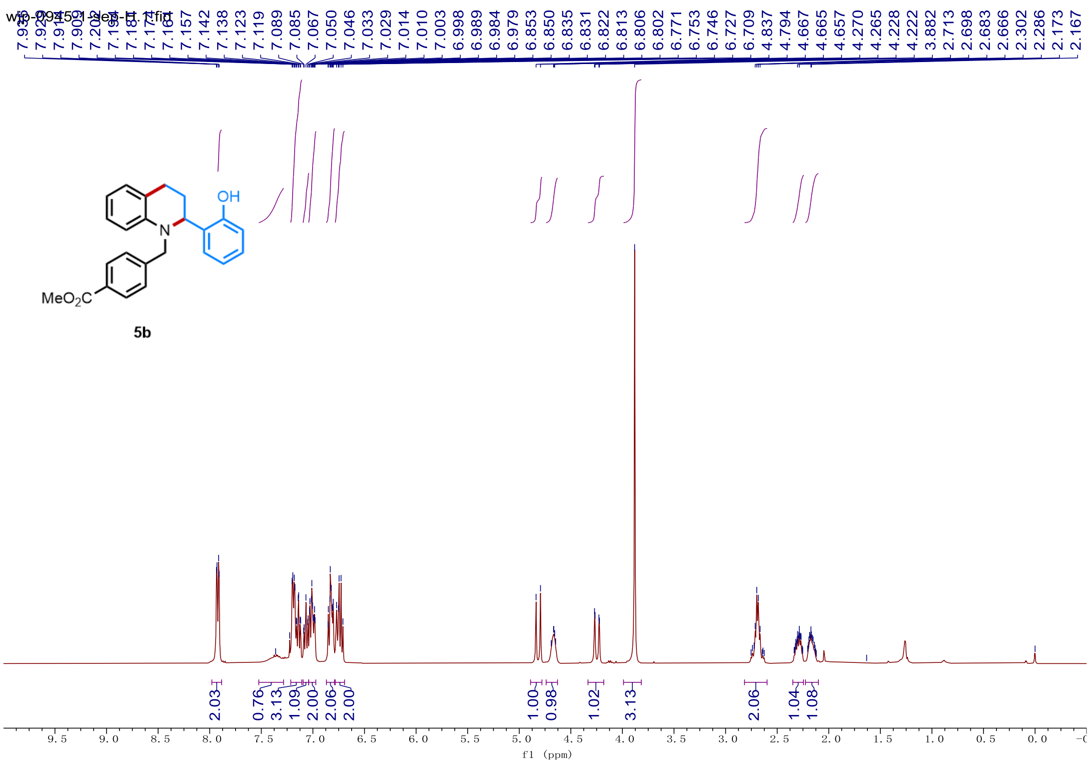

Supplementary Figure 130. ^{1}**H NMR (400 MHz, CDCl$_3$) spectrum of 5b.**

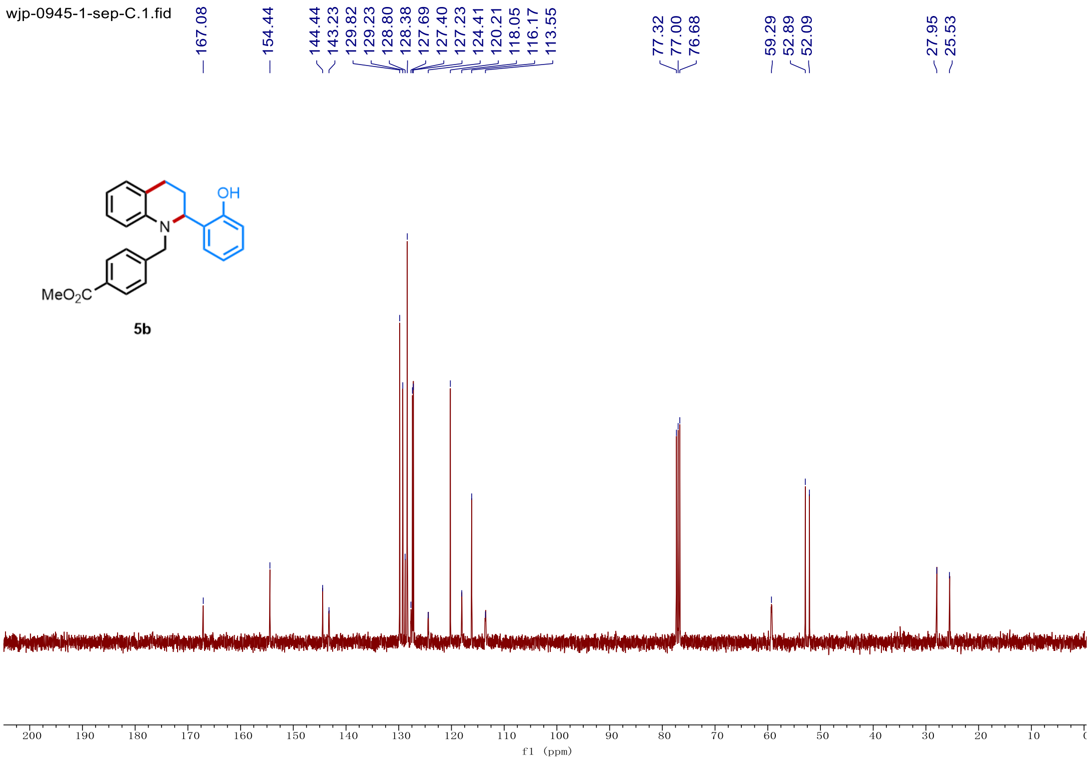

Supplementary Figure 131. ^{13}C NMR (100 MHz, CDCl$_3$) spectrum of 5b.

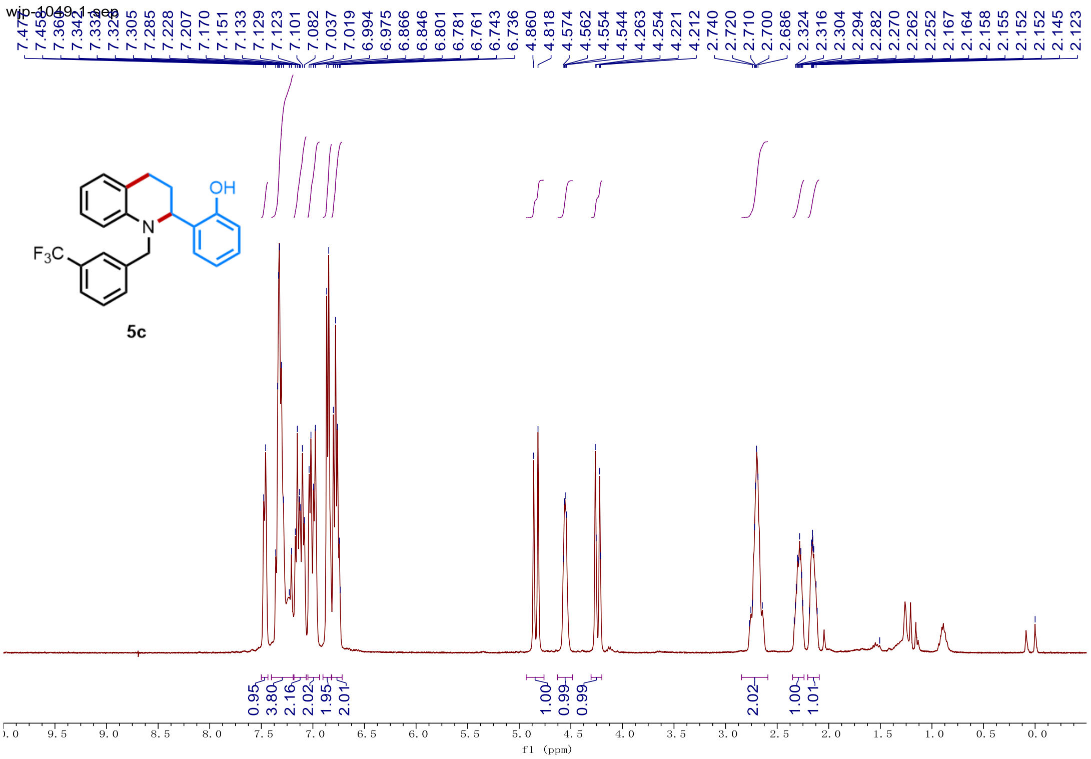

Supplementary Figure 132. ^{1}H NMR (400 MHz, CDCl$_3$) spectrum of 5c.

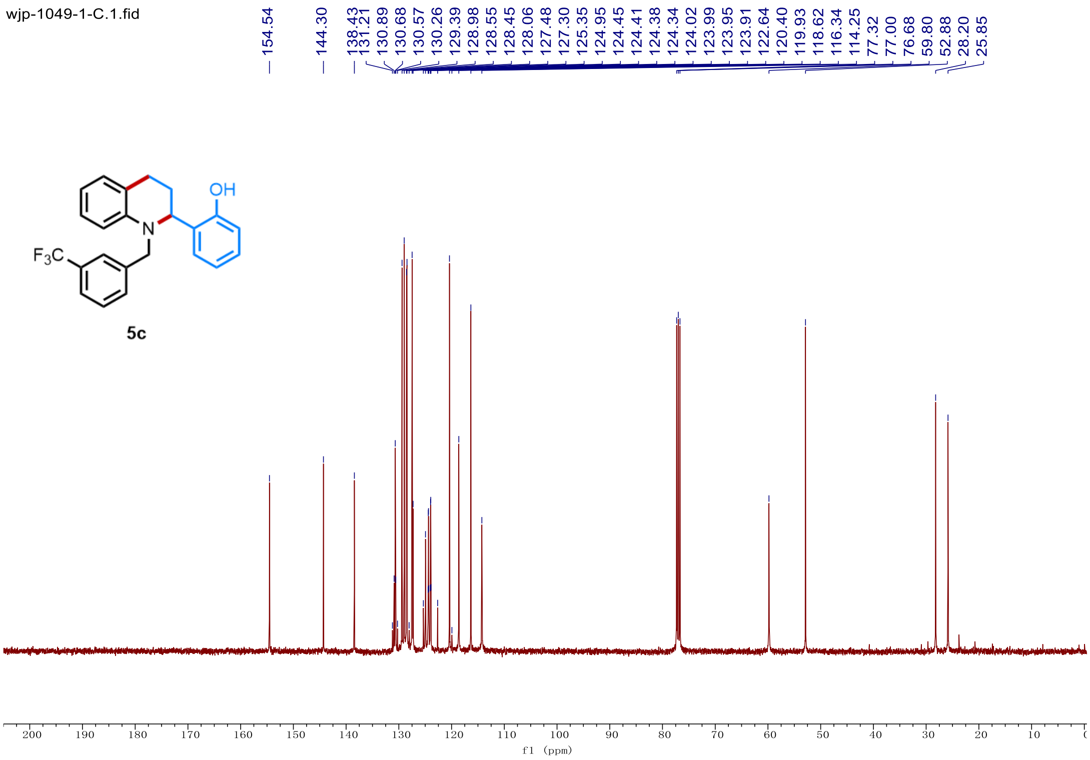

Supplementary Figure 133. ^{13}C NMR (100 MHz, $CDCl_3$) spectrum of 5c.

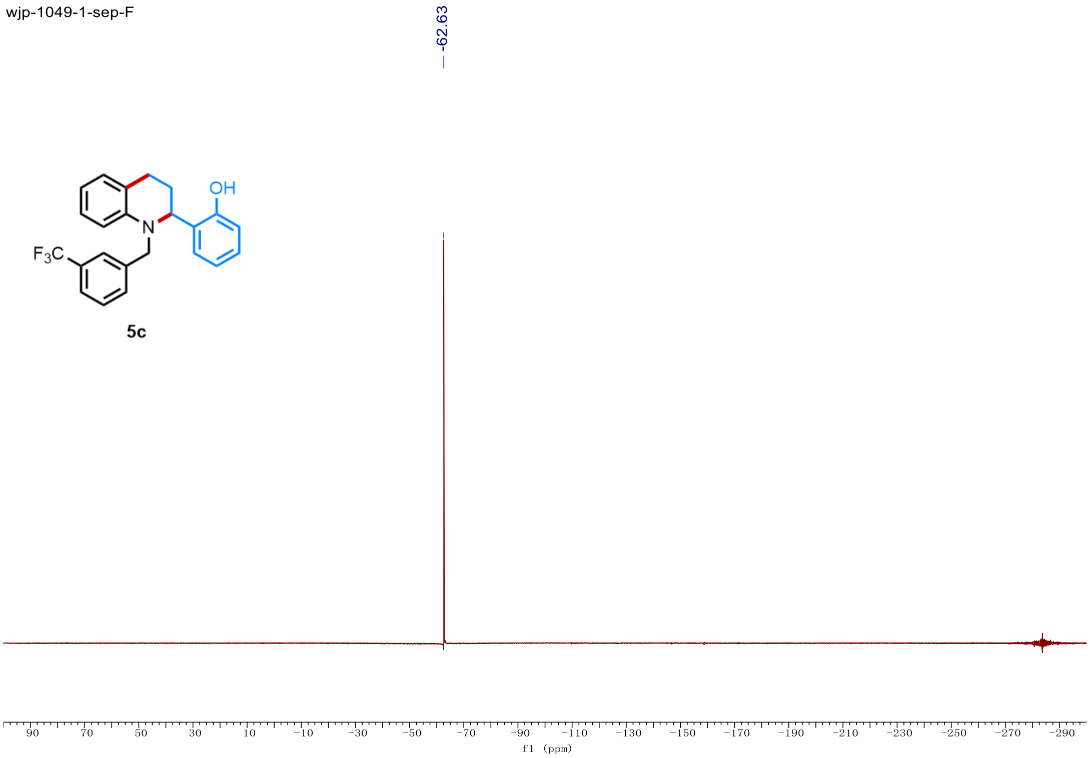

Supplementary Figure 134. ^{19}F NMR (375 MHz, $CDCl_3$) spectrum of 5c.

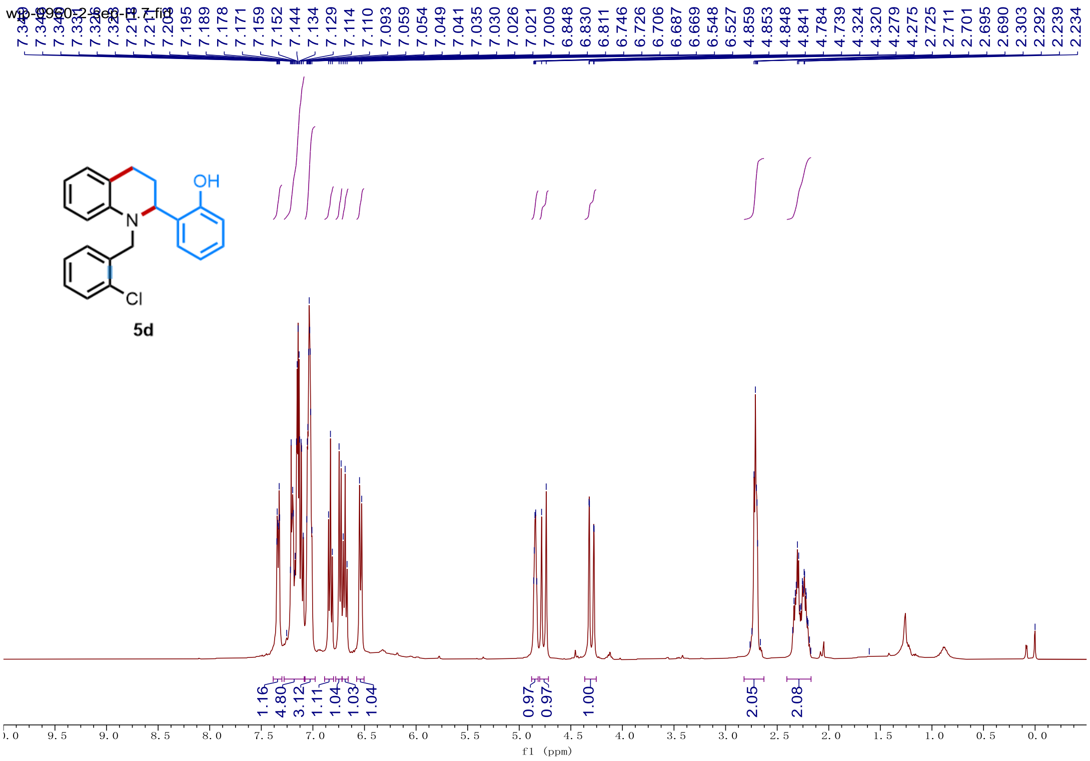

Supplementary Figure 135. ^{1}H NMR (400 MHz, $CDCl_3$) spectrum of 5d.

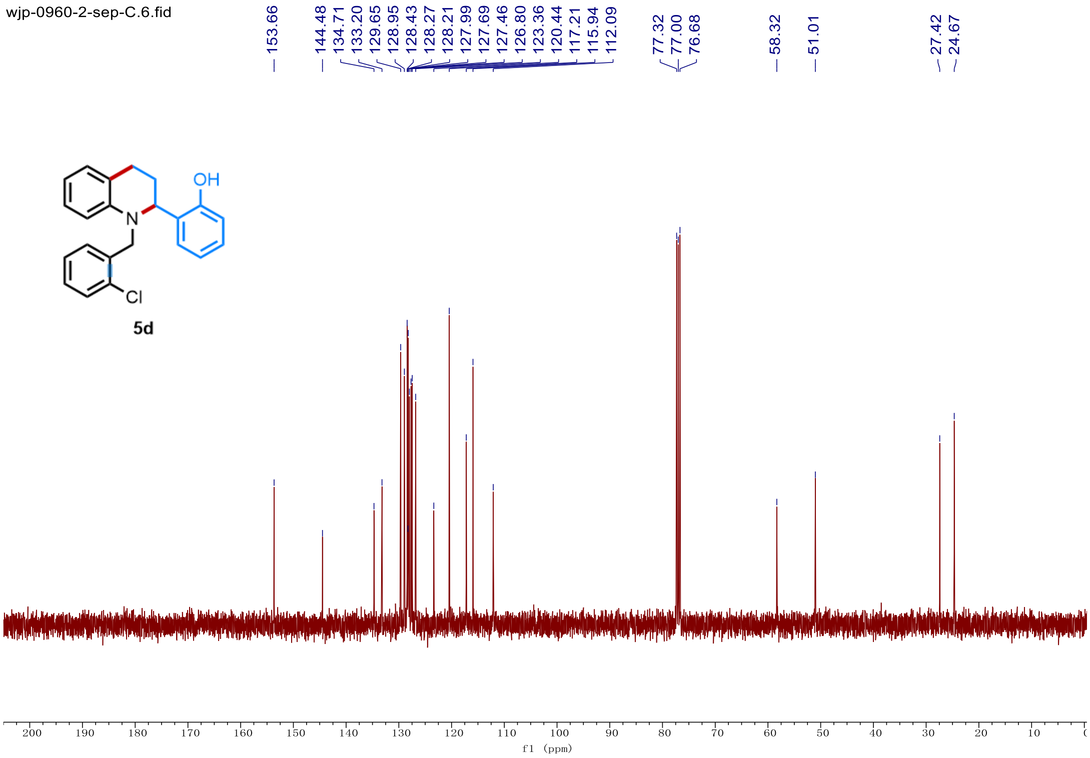

Supplementary Figure 136. ^{13}C NMR (100 MHz, $CDCl_3$) spectrum of 5d.

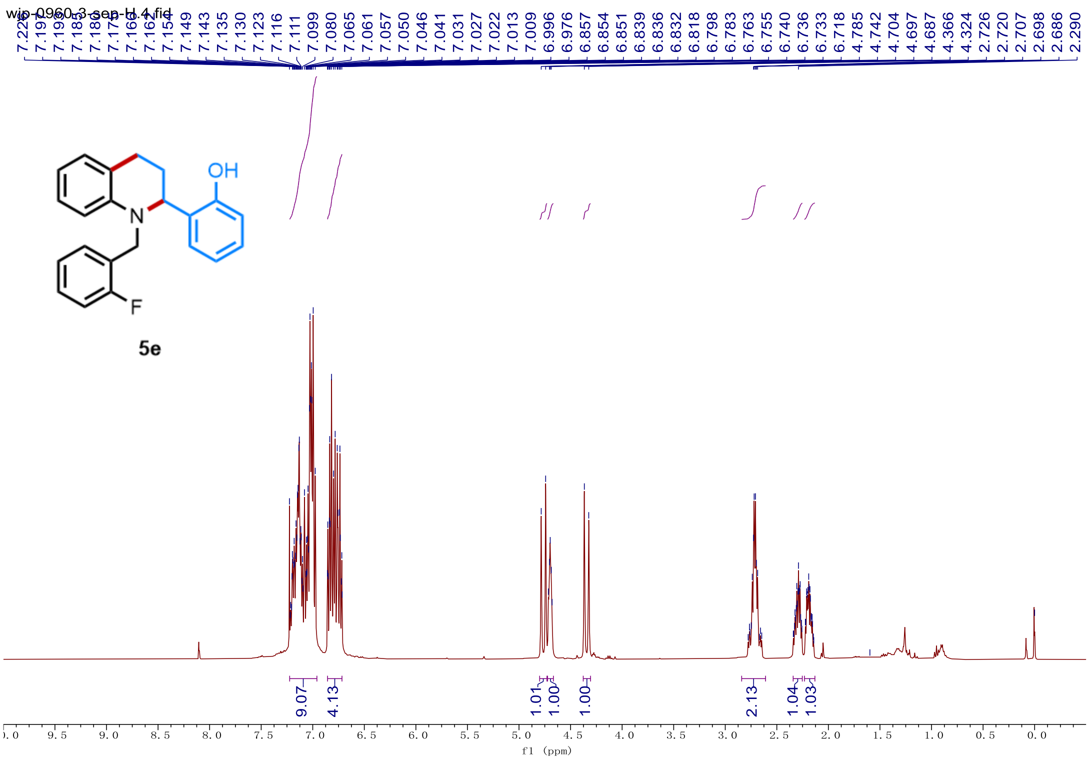

Supplementary Figure 137. ^{1}H NMR (400 MHz, $CDCl_3$) spectrum of 5e.

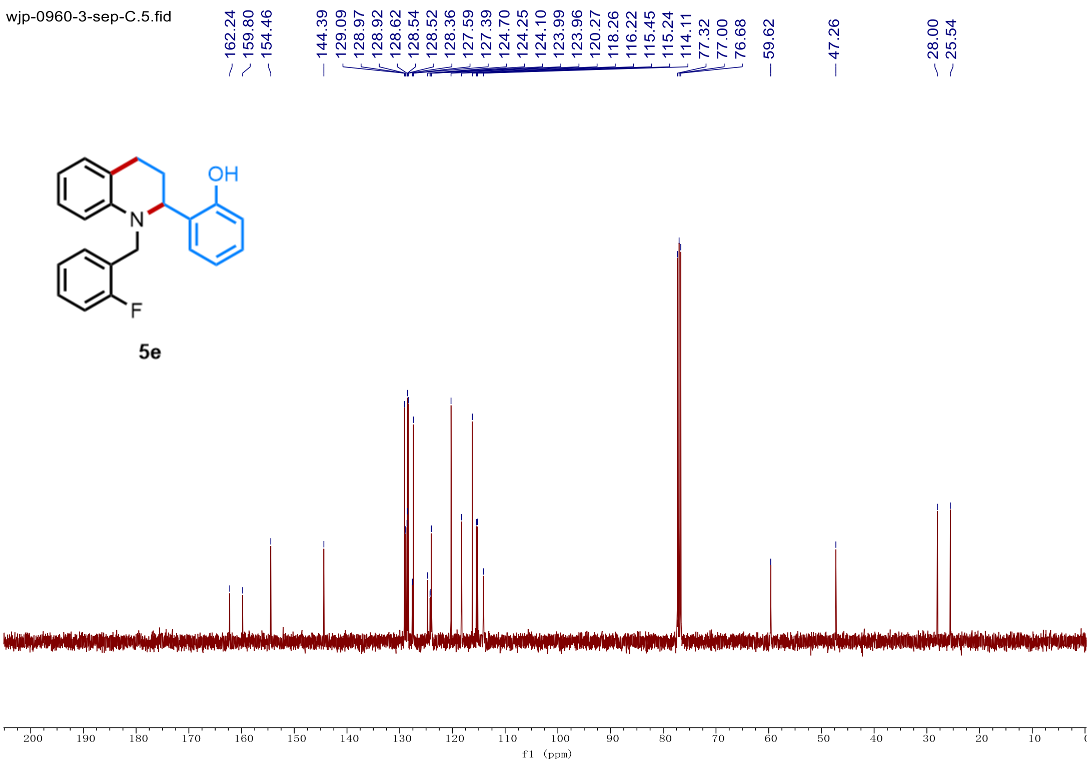

Supplementary Figure 138. ^{13}C NMR (100 MHz, $CDCl_3$) spectrum of 5e.

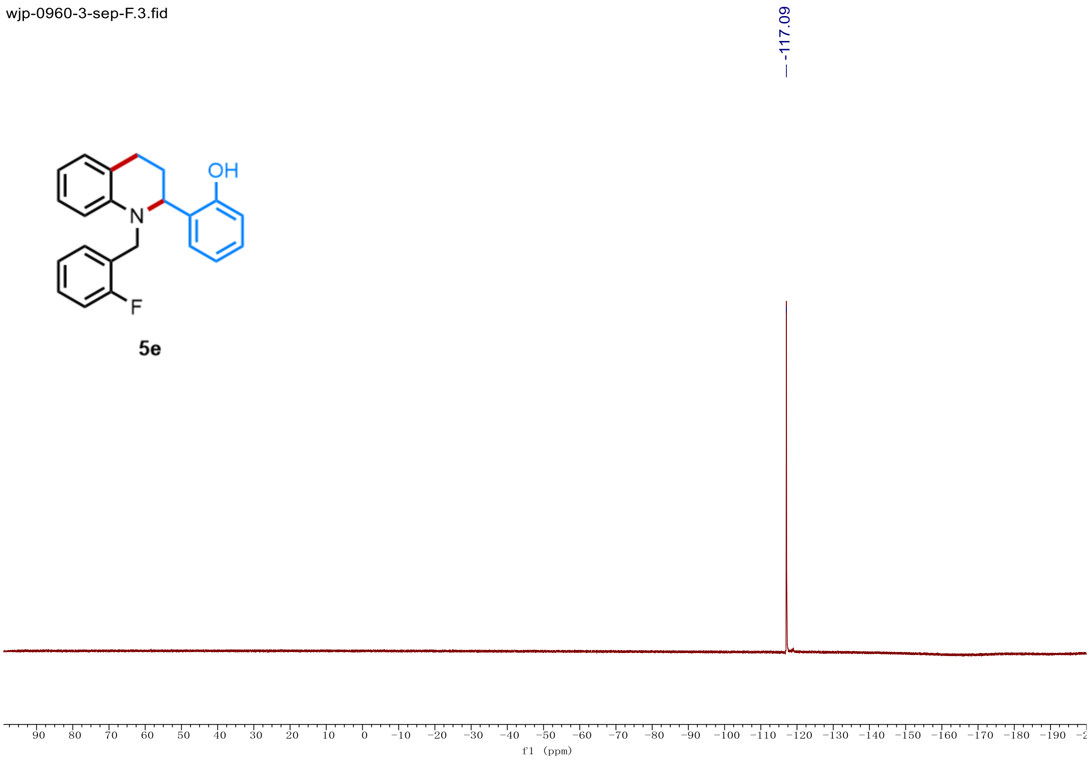

Supplementary Figure 139. ^{19}F NMR (375 MHz, CDCl$_3$) spectrum of 5e.

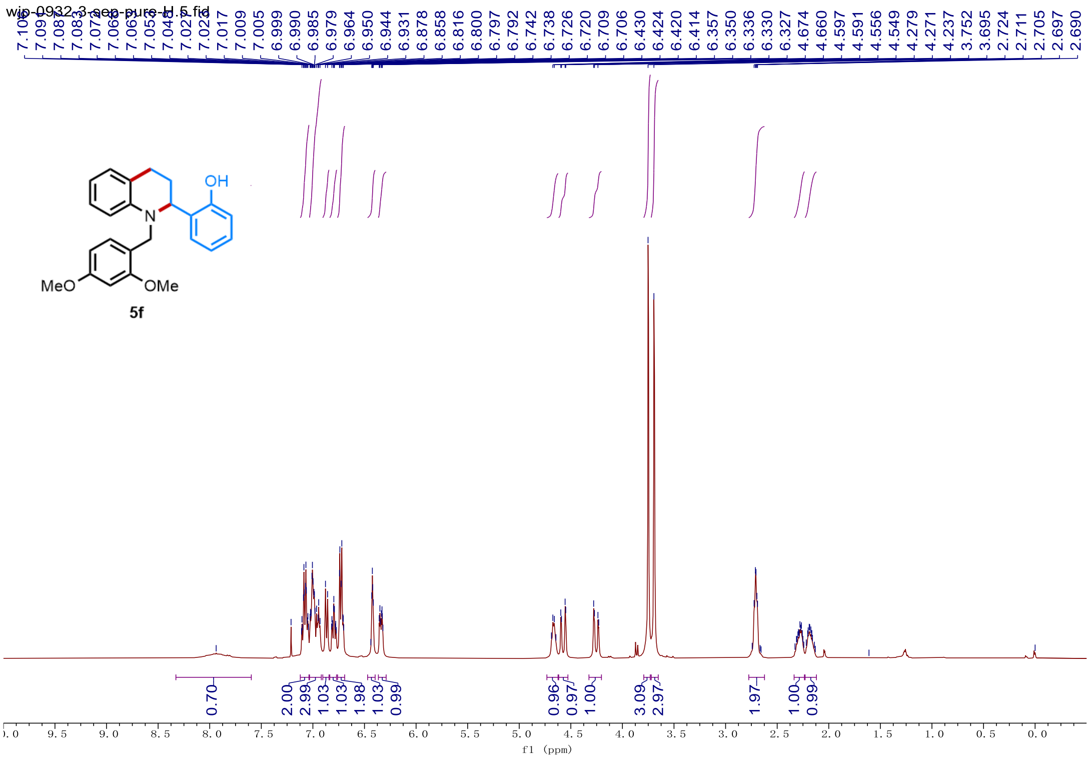

Supplementary Figure 140. ^{1}H NMR (400 MHz, CDCl$_3$) spectrum of 5f.

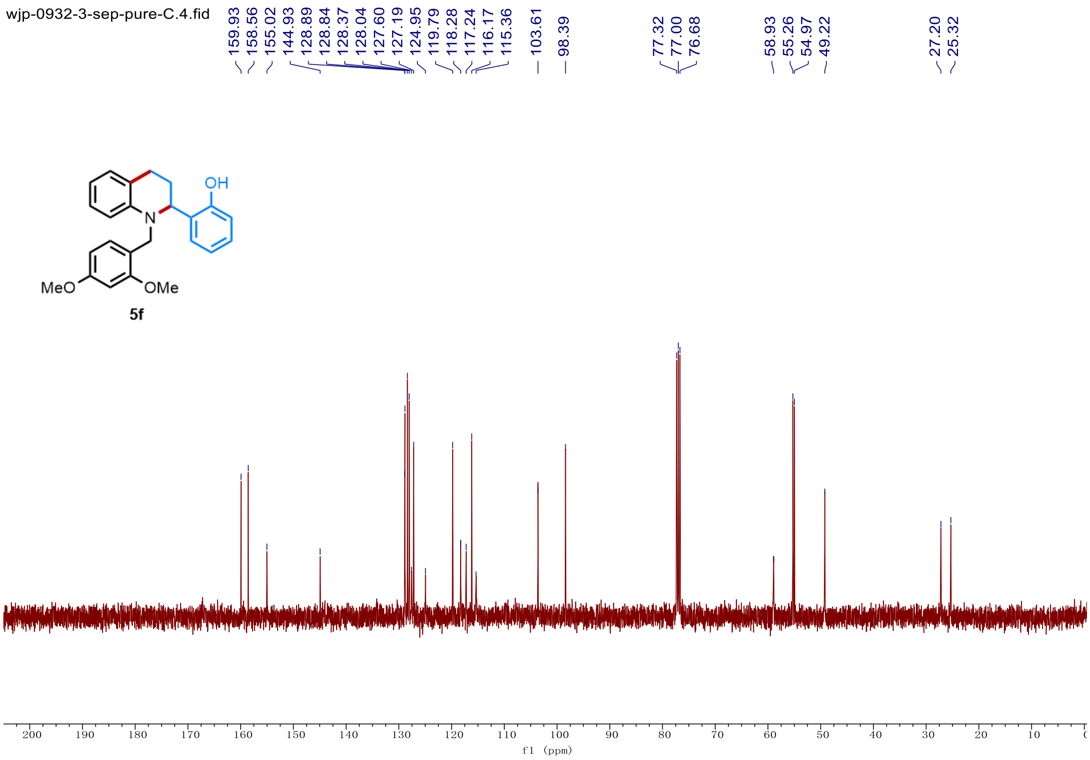

Supplementary Figure 141. ^{13}**C NMR (100 MHz, CDCl$_3$) spectrum of 5f.**

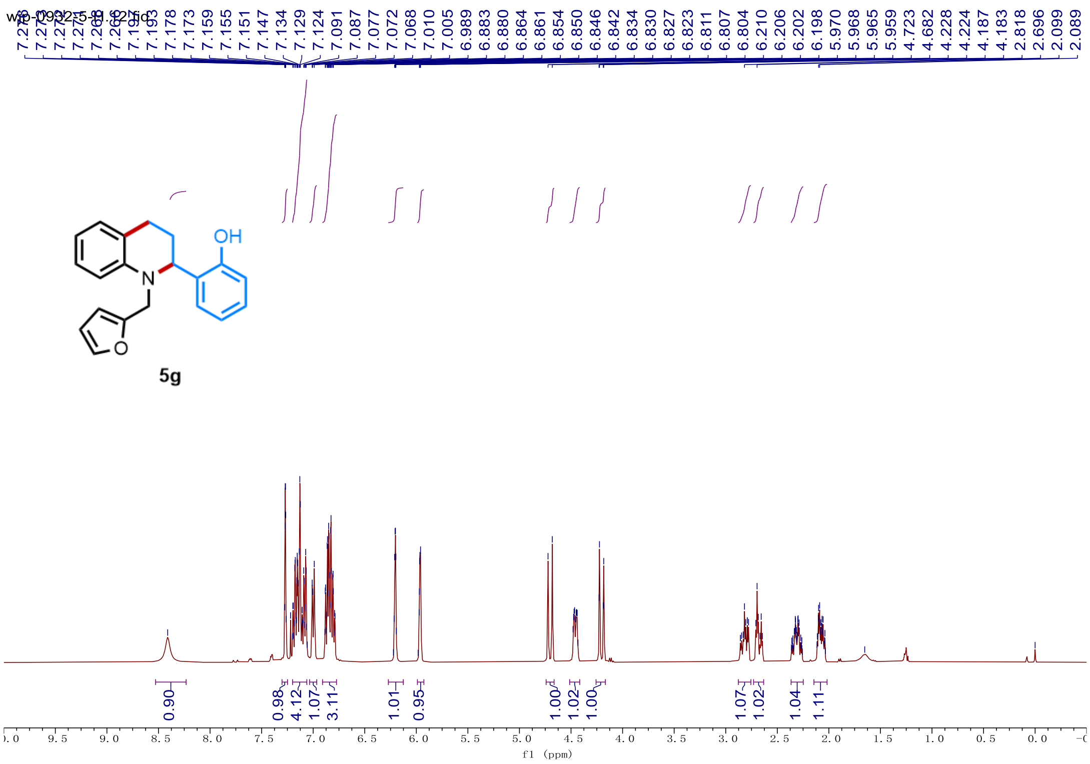

Supplementary Figure 142. ^{1}**H NMR (400 MHz, CDCl$_3$) spectrum of 5g.**

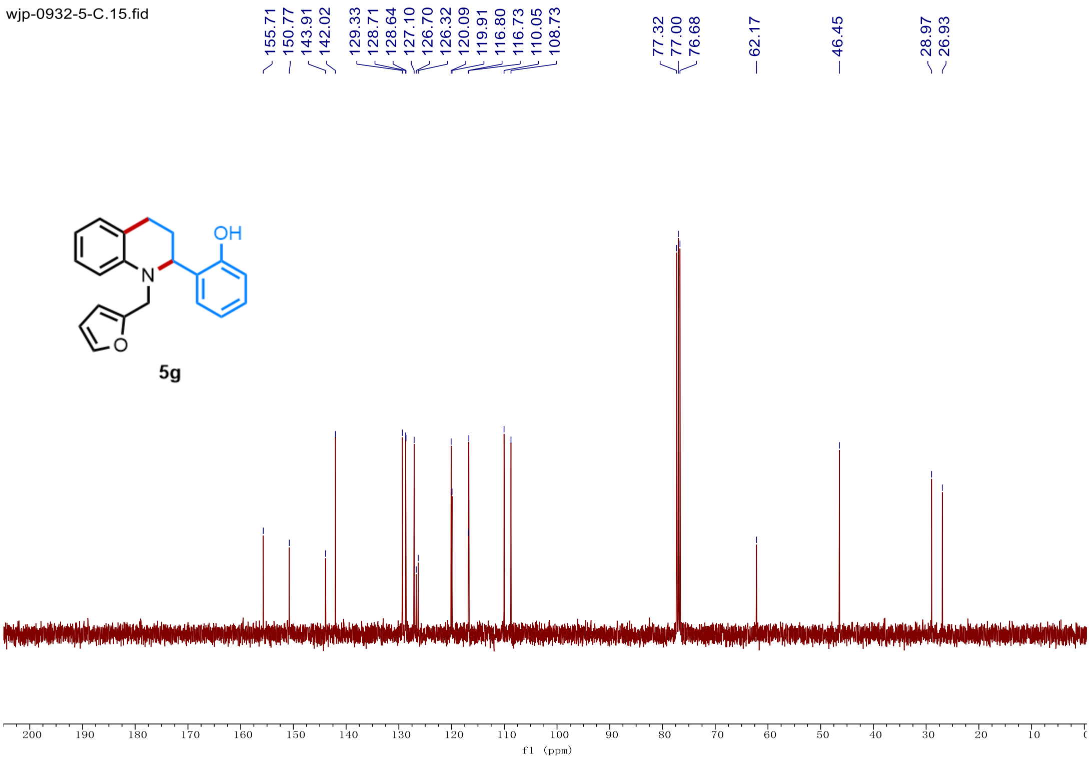

Supplementary Figure 143. **^{13}C NMR (100 MHz, CDCl$_3$) spectrum of 5g.**

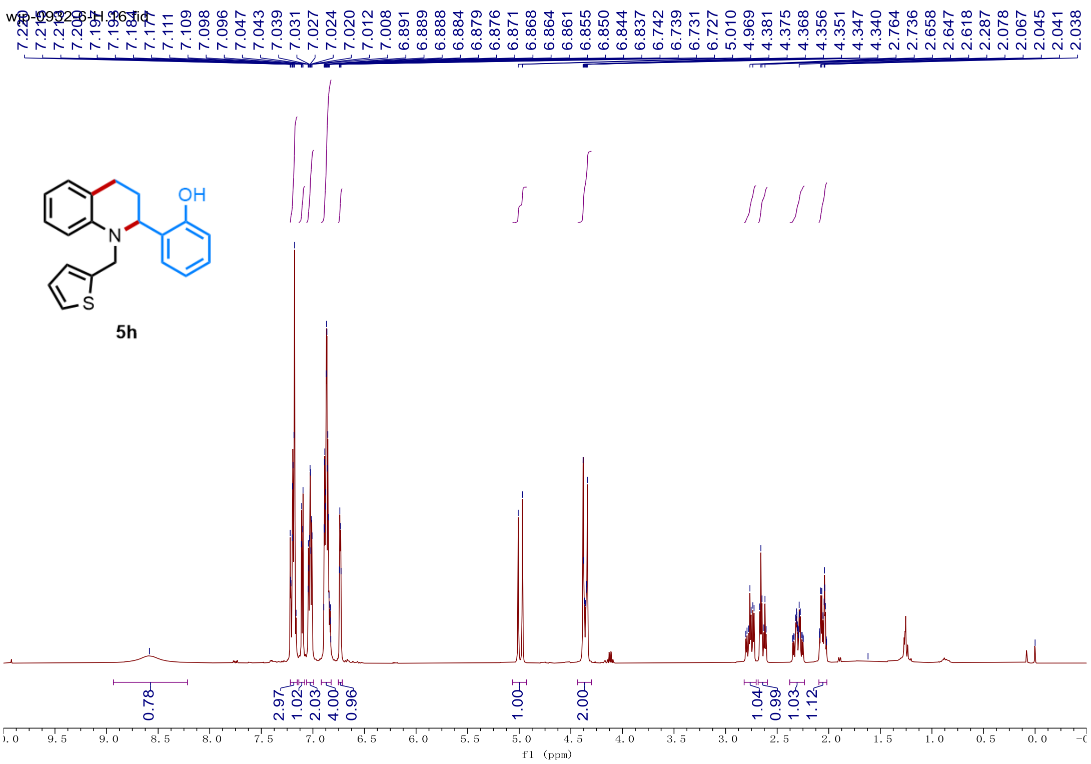

Supplementary Figure 144. **^{1}H NMR (400 MHz, CDCl$_3$) spectrum of 5h.**

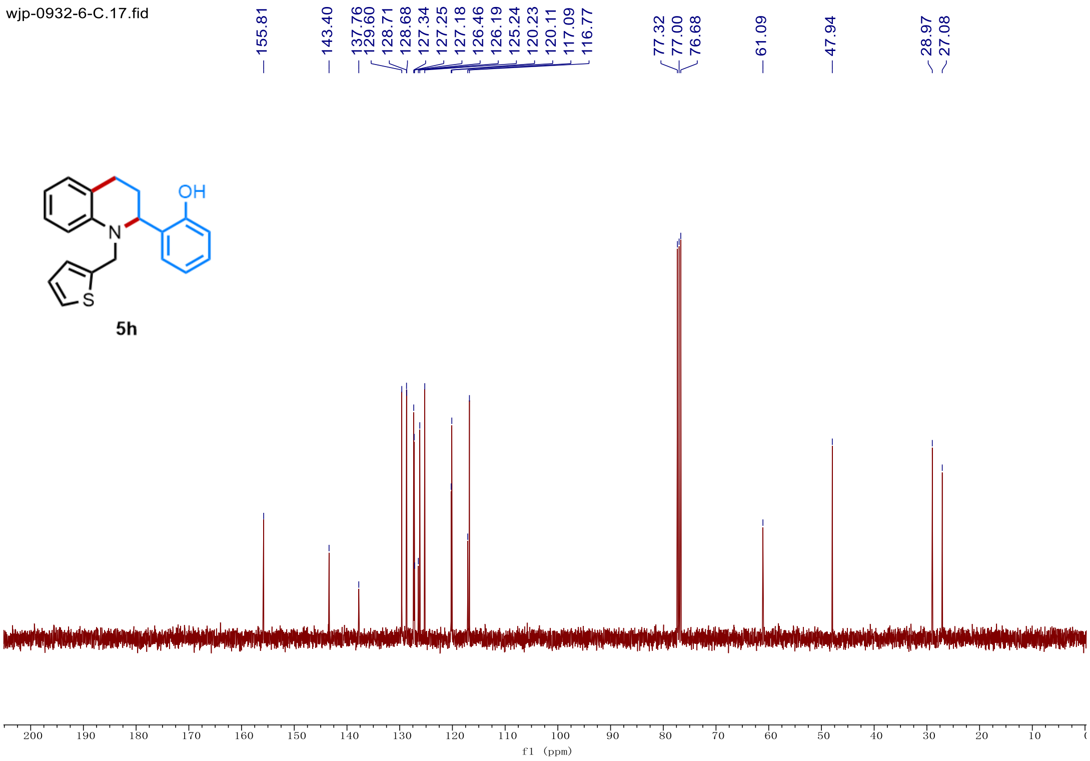

Supplementary Figure 145. ^{13}C NMR (100 MHz, $CDCl_3$) spectrum of 5h.

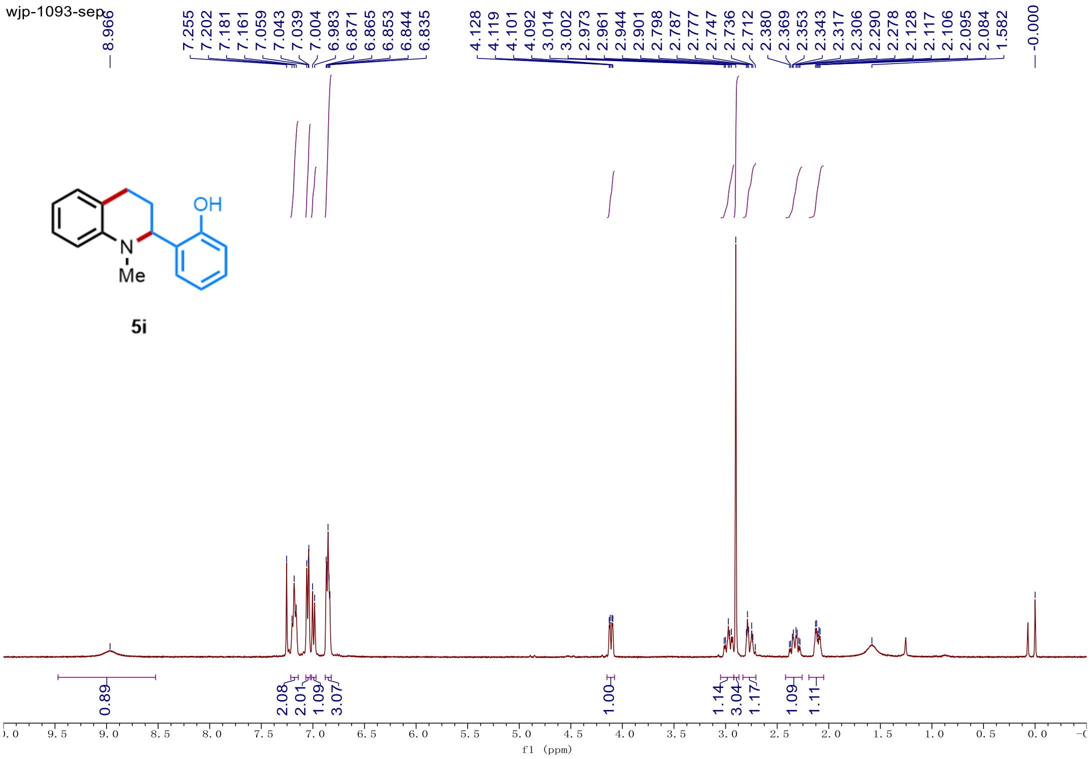

Supplementary Figure 146. ^{1}H NMR (400 MHz, $CDCl_3$) spectrum of 5i.

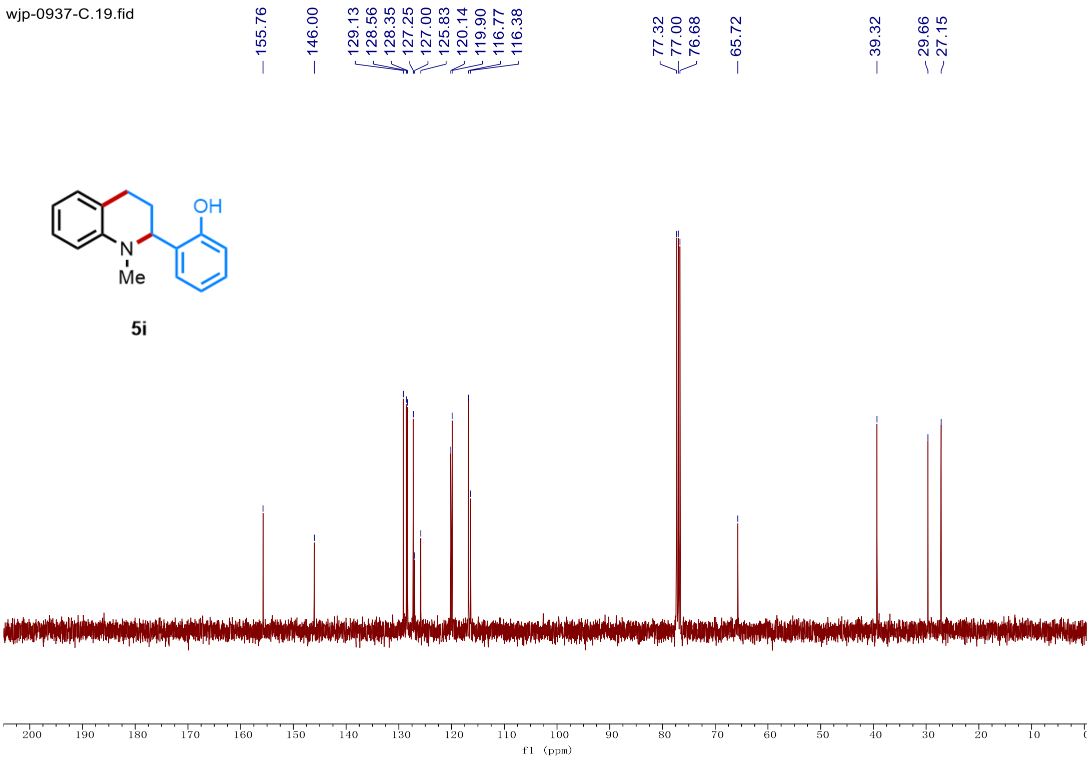

Supplementary Figure 147. ^{13}C NMR (100 MHz, $CDCl_3$) spectrum of 5i.

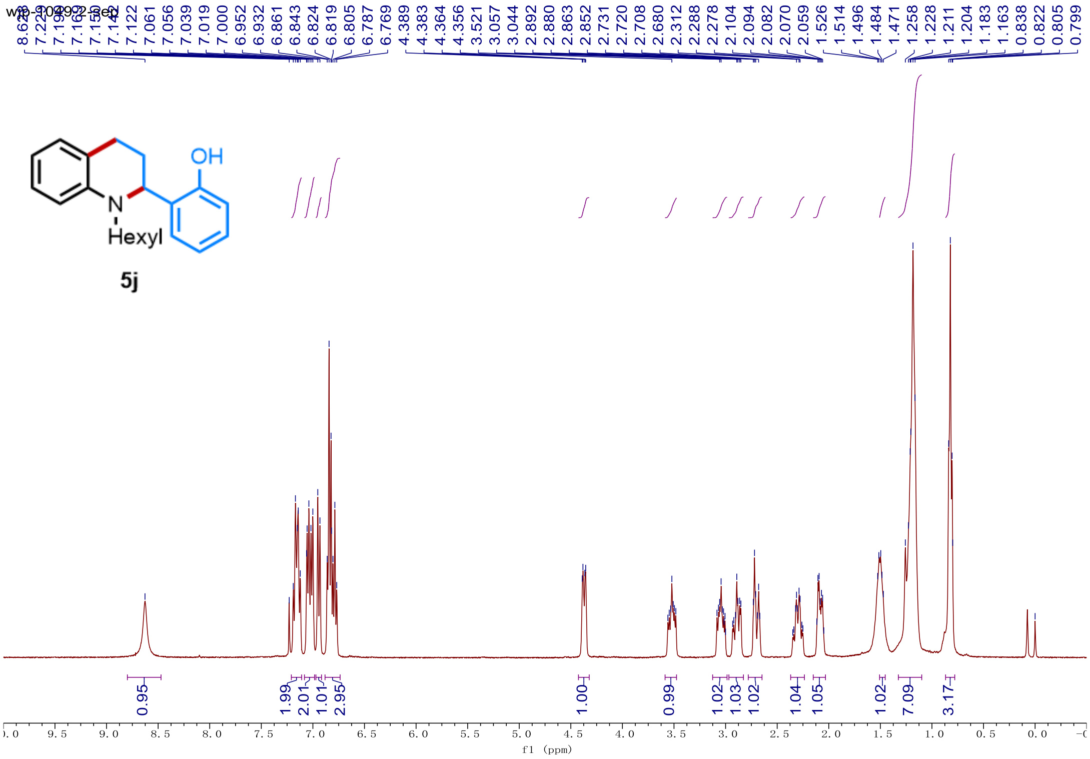

Supplementary Figure 148. ^{1}H NMR (400 MHz, $CDCl_3$) spectrum of 5j.

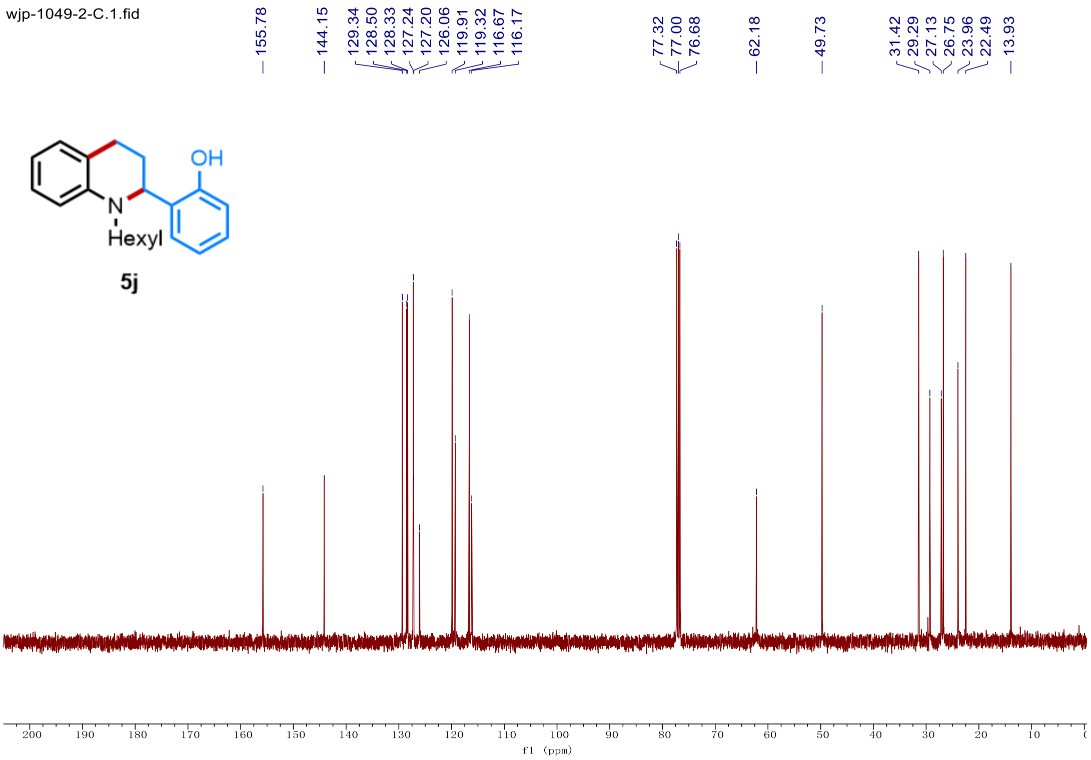

Supplementary Figure 149. ^{13}C NMR (100 MHz, $CDCl_3$) spectrum of 5j.

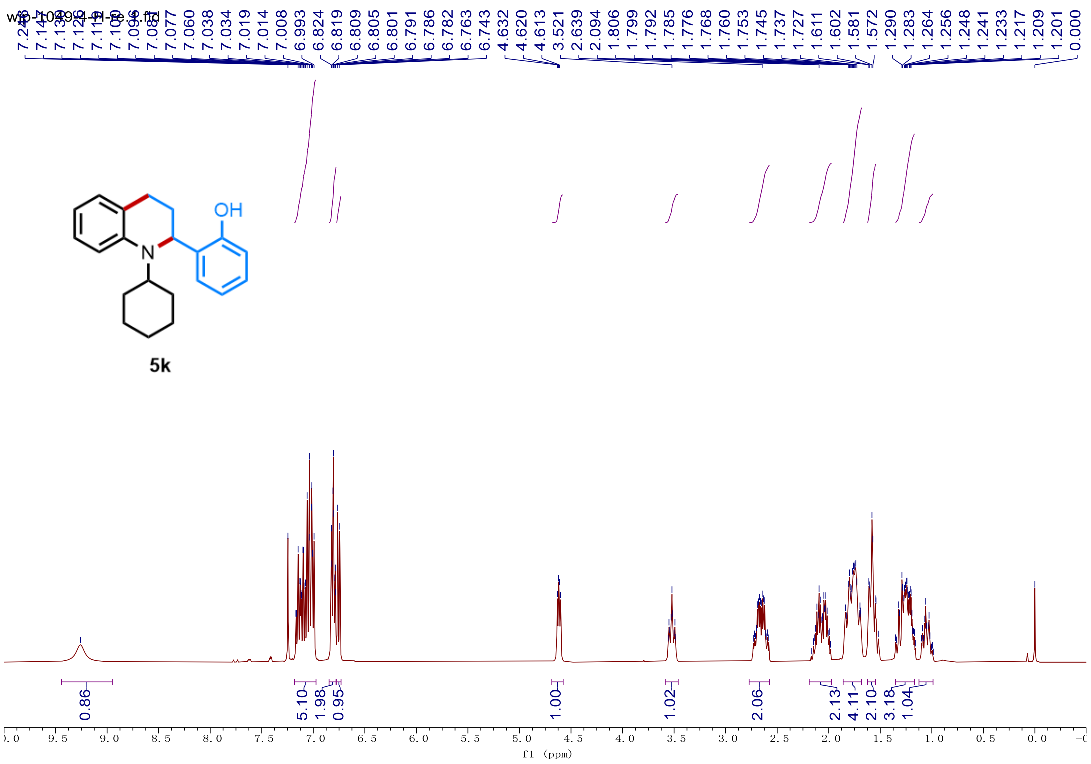

Supplementary Figure 150. ^{1}H NMR (400 MHz, $CDCl_3$) spectrum of 5k.

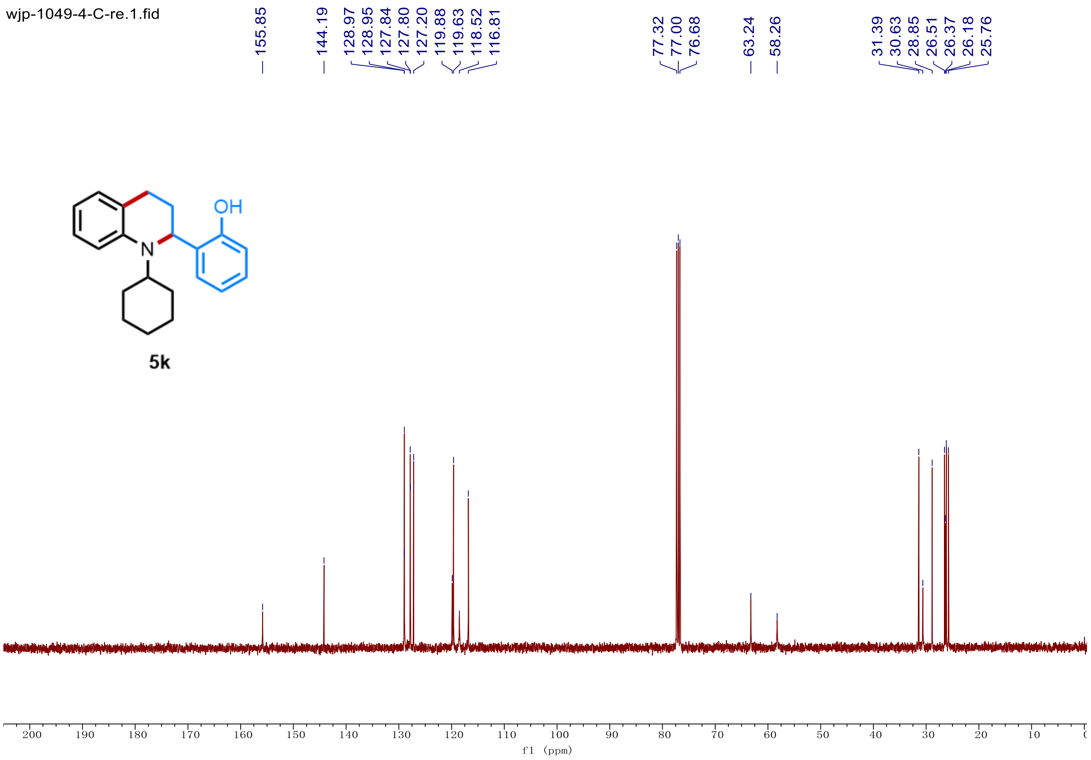

Supplementary Figure 151. ^{13}**C NMR (100 MHz, CDCl$_3$) spectrum of 5k.**

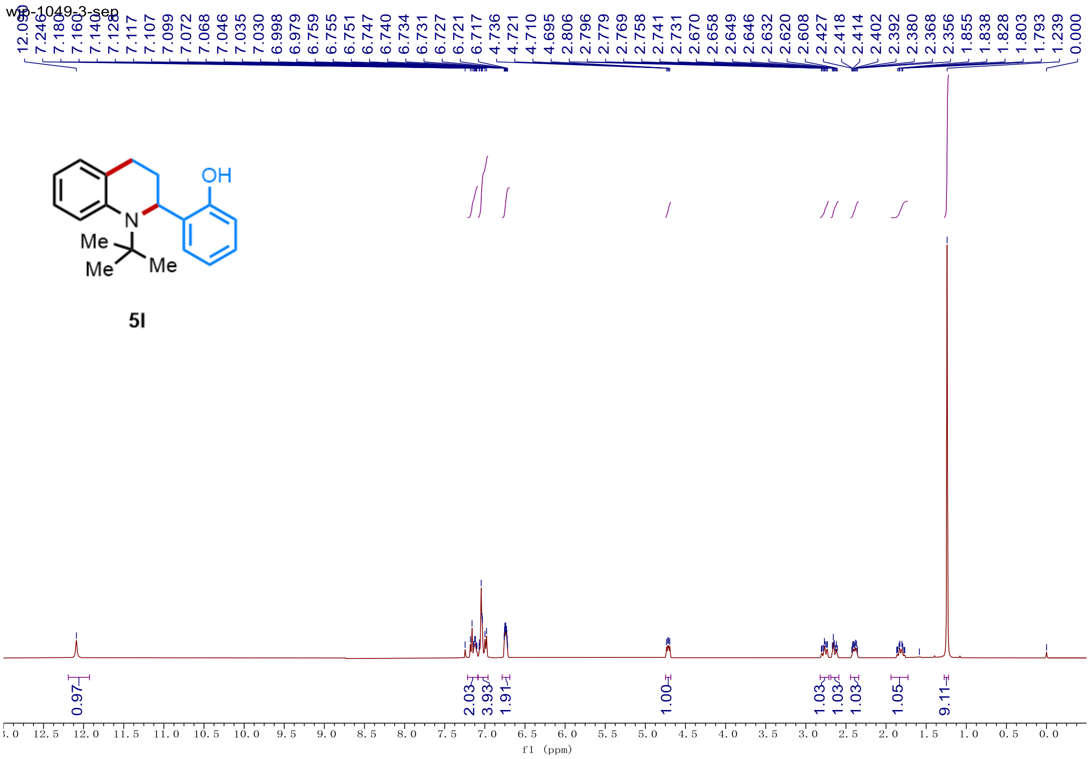

Supplementary Figure 152. ^{1}**H NMR (400 MHz, CDCl$_3$) spectrum of 5l.**

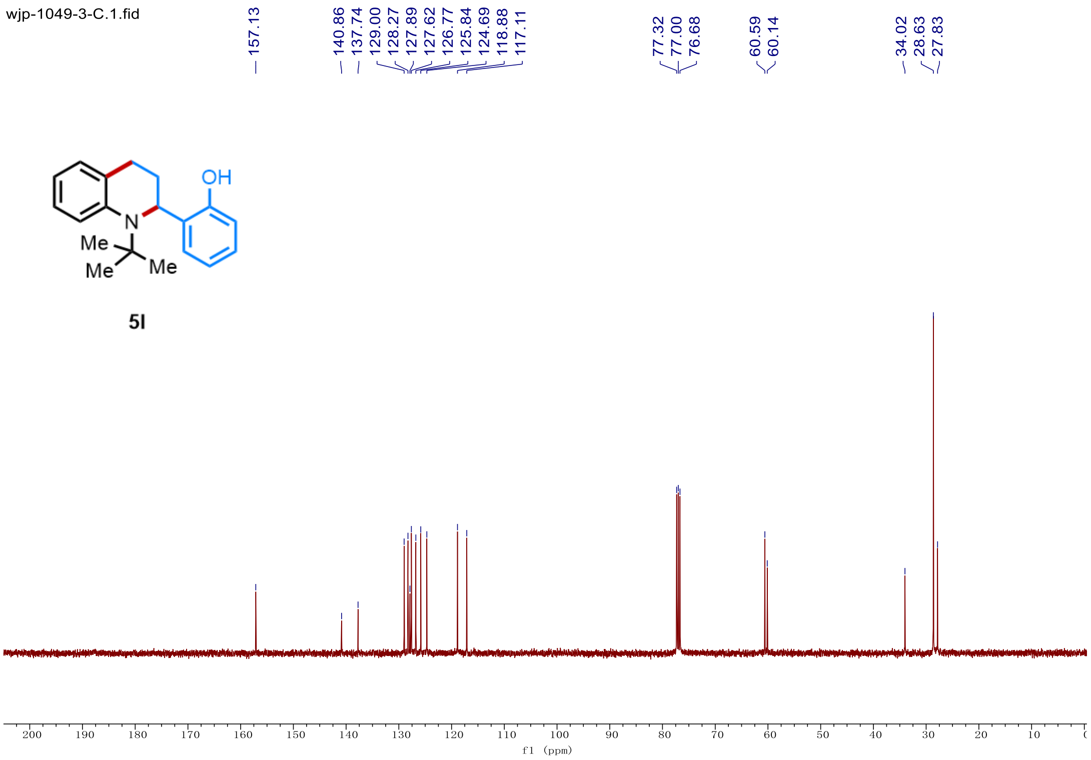

Supplementary Figure 153. ^{13}C NMR (100 MHz, $CDCl_3$) spectrum of 5l.

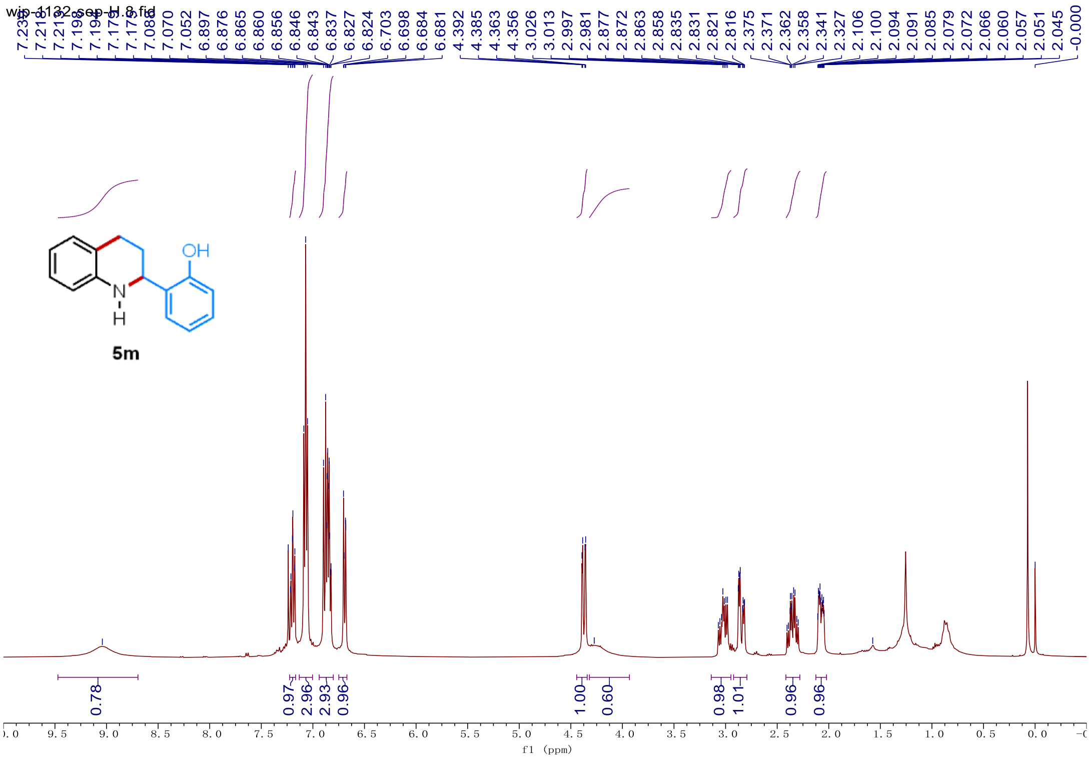

Supplementary Figure 154. ^{1}H NMR (400 MHz, $CDCl_3$) spectrum of 5m.

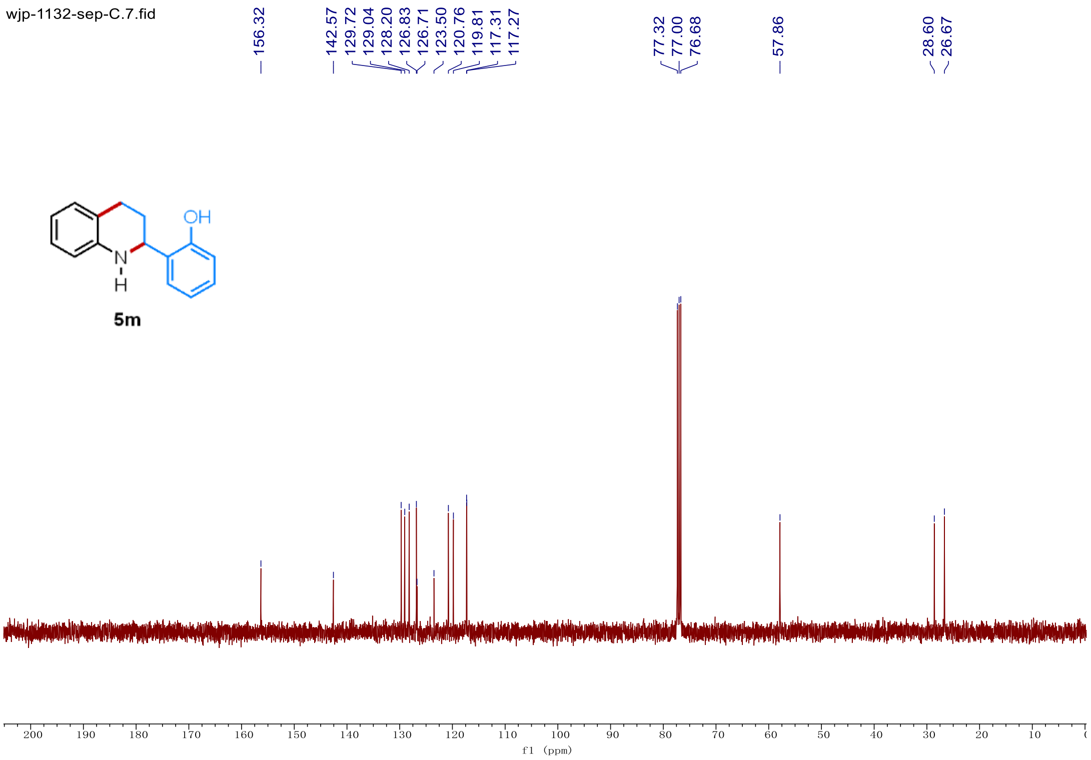

Supplementary Figure 155. ^{13}C NMR (100 MHz, $CDCl_3$) spectrum of 5m.

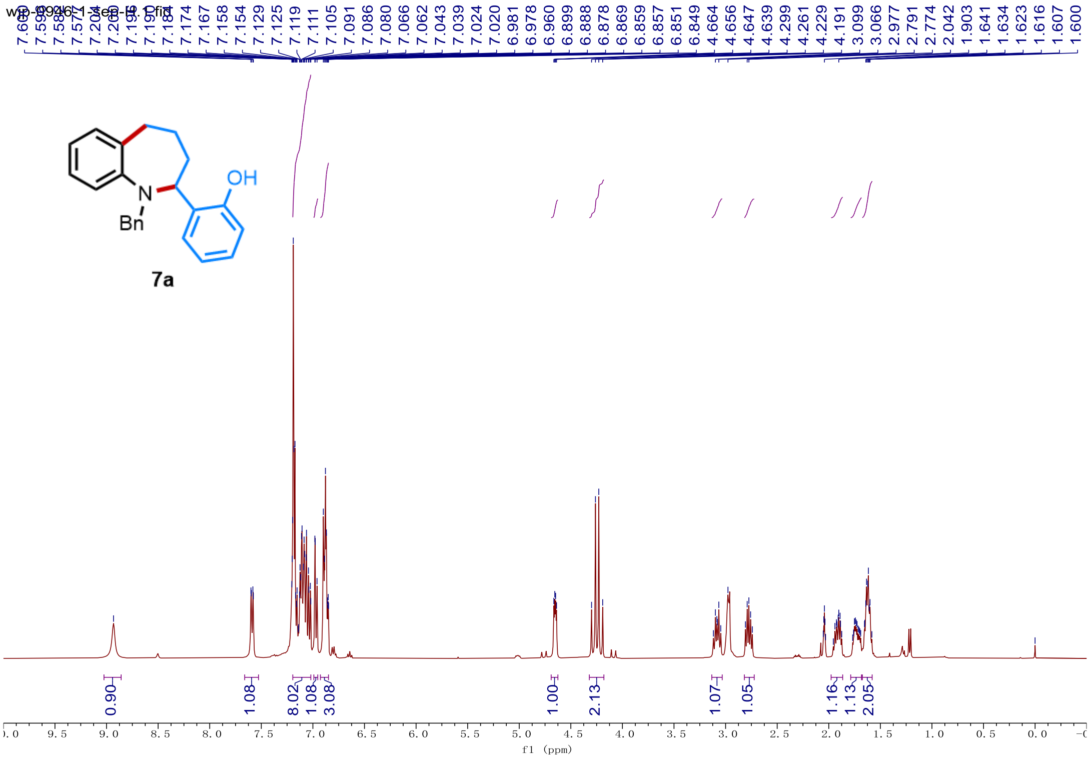

Supplementary Figure 156. ^{1}H NMR (400 MHz, acetone-d_6) spectrum of 7a.

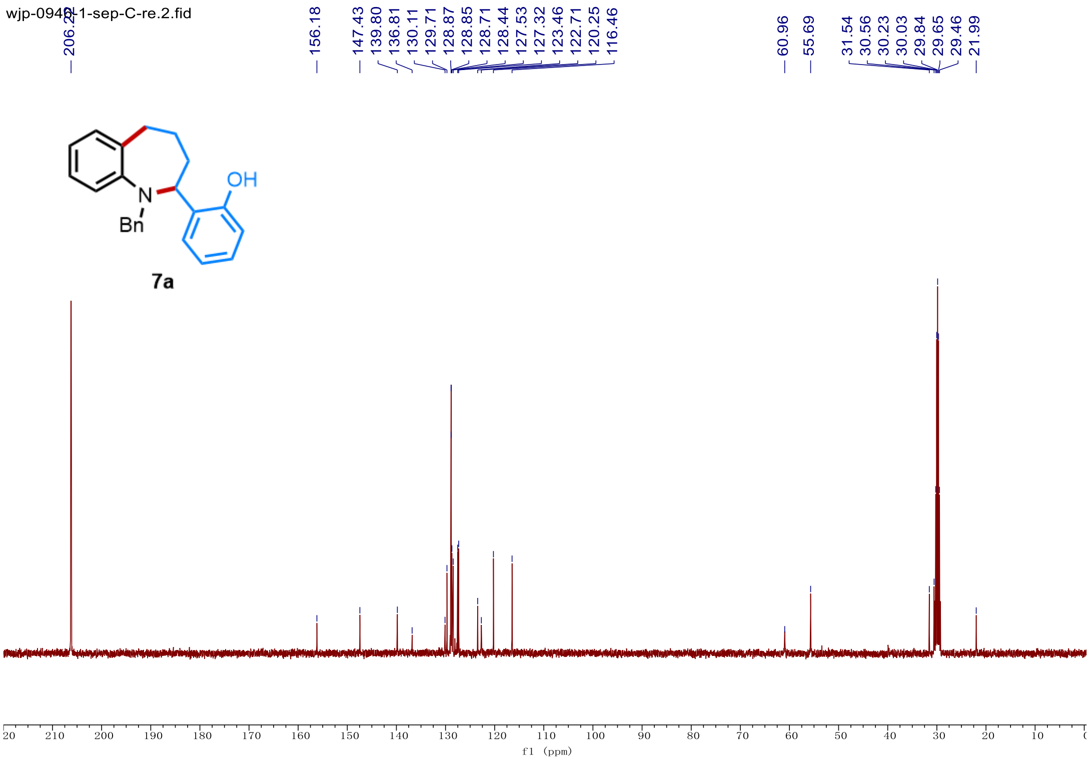

Supplementary Figure 157. **^{13}C NMR (100 MHz, acetone-d_6) spectrum of 7a.**

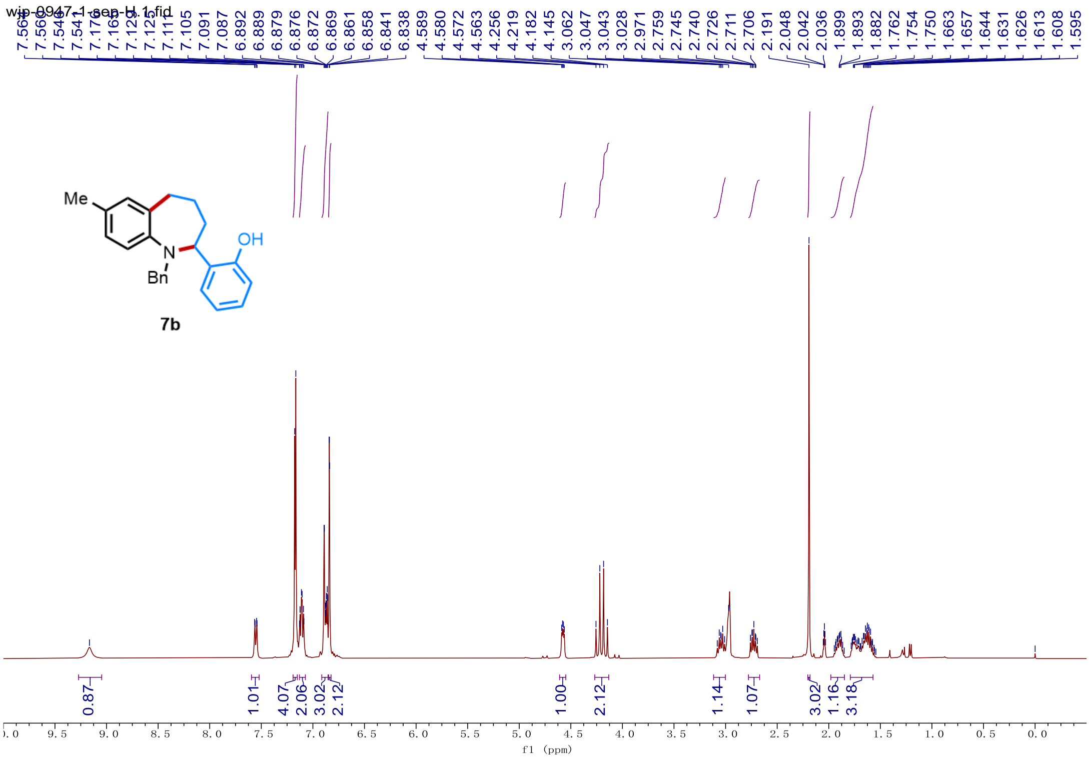

Supplementary Figure 158. **^{1}H NMR (400 MHz, acetone-d_6) spectrum of 7b.**

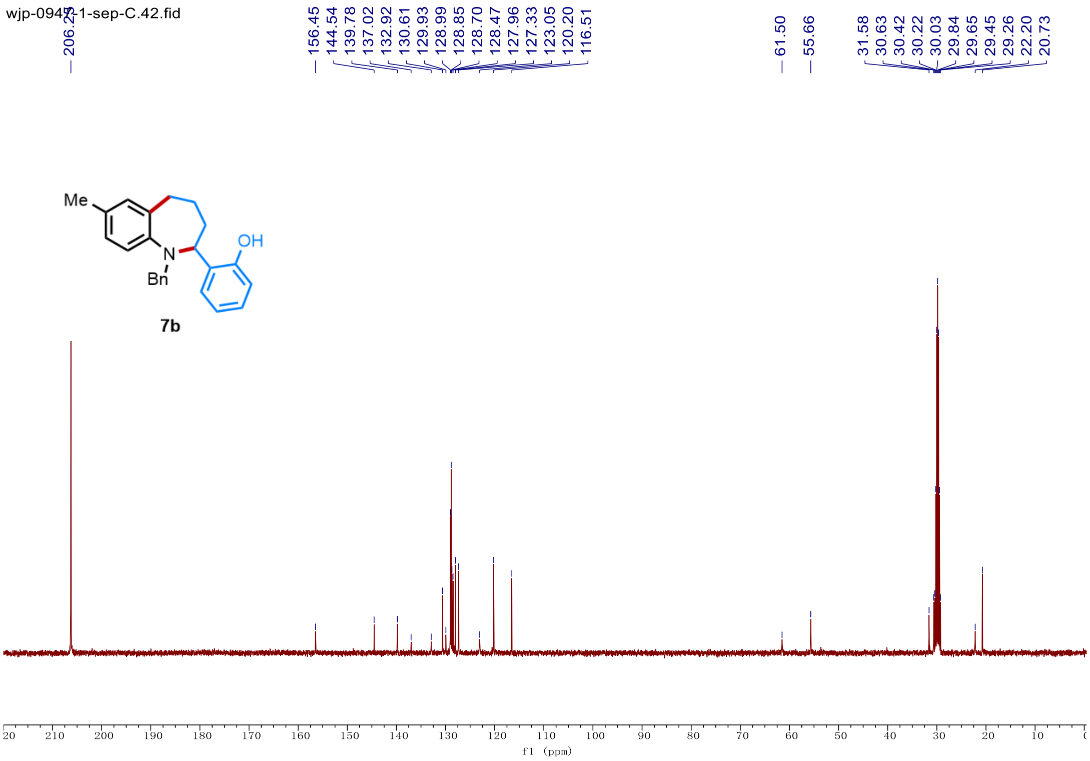

Supplementary Figure 159. ^{13}C NMR (100 MHz, acetone-d_6) spectrum of 7b.

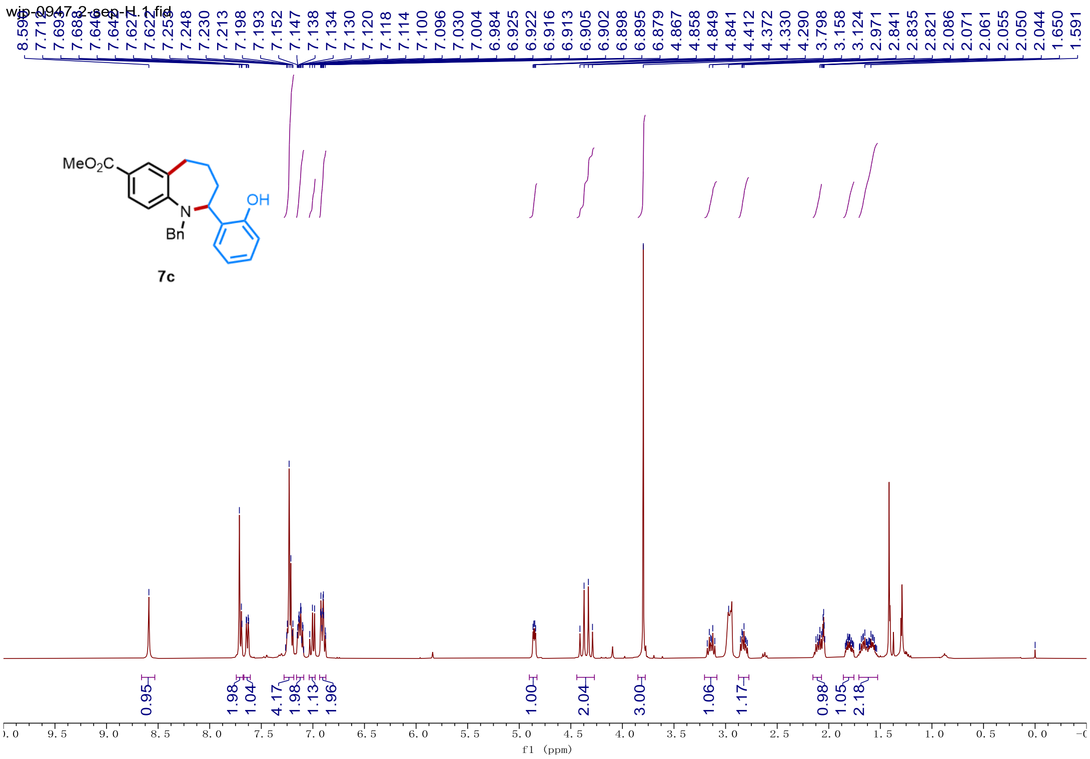

Supplementary Figure 160. ^{1}H NMR (400 MHz, acetone-d_6) spectrum of 7c.

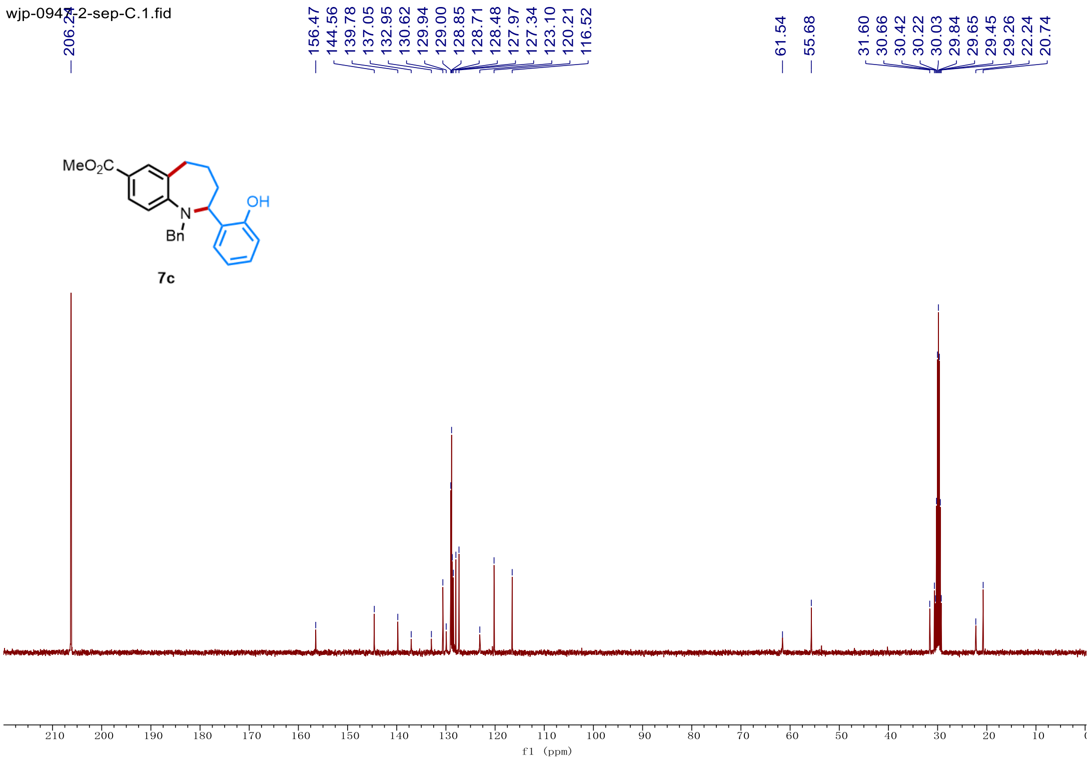

Supplementary Figure 161. ^{13}**C NMR (100 MHz, acetone-*d*$_6$) spectrum of 7c.**

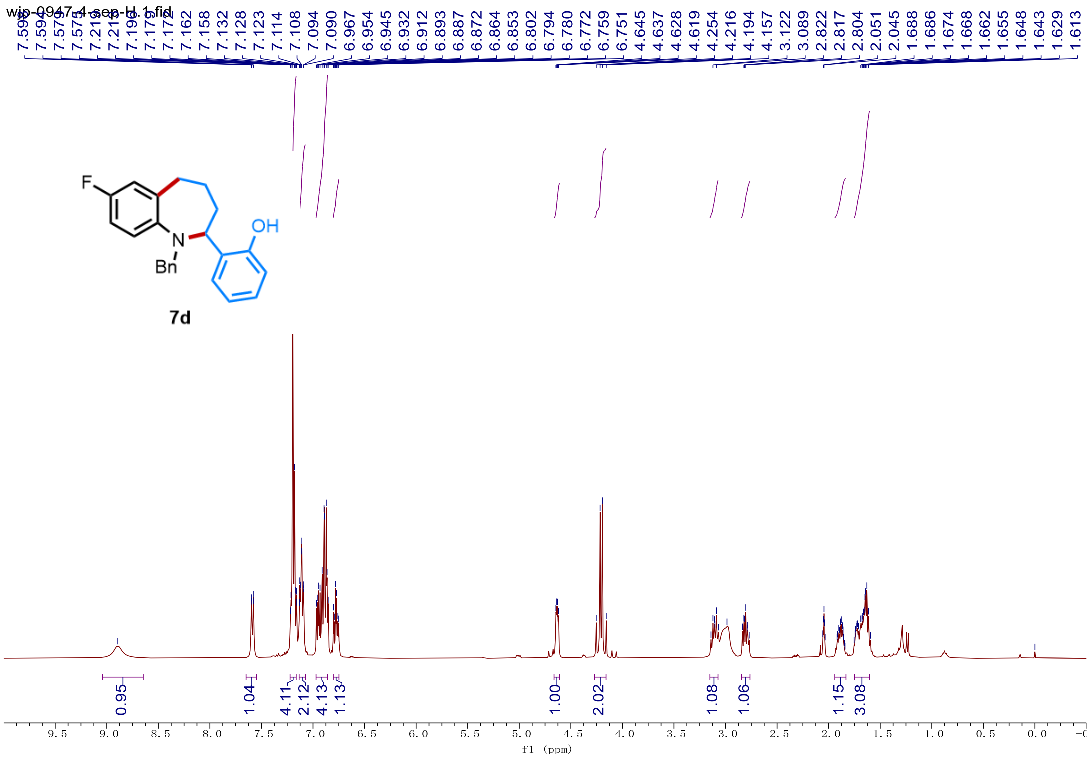

Supplementary Figure 162. ^{1}**H NMR (400 MHz, acetone-*d*$_6$) spectrum of 7d.**

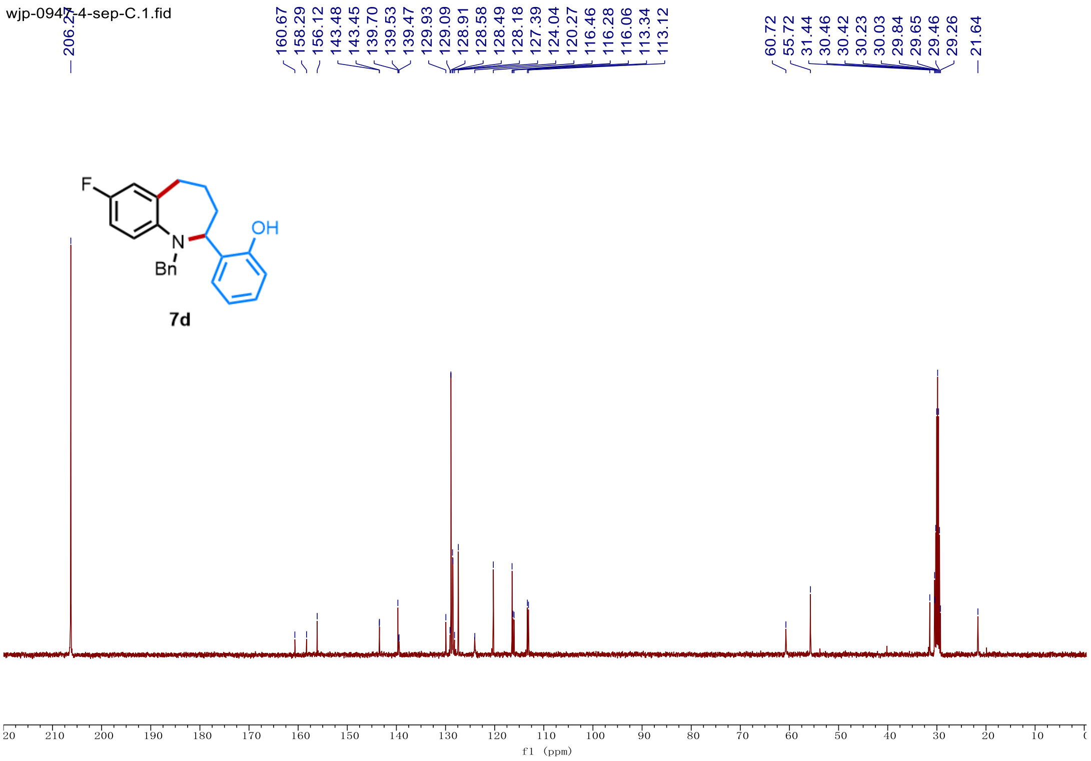

Supplementary Figure 163. ^{13}C NMR (100 MHz, acetone-d_6) spectrum of 7d.

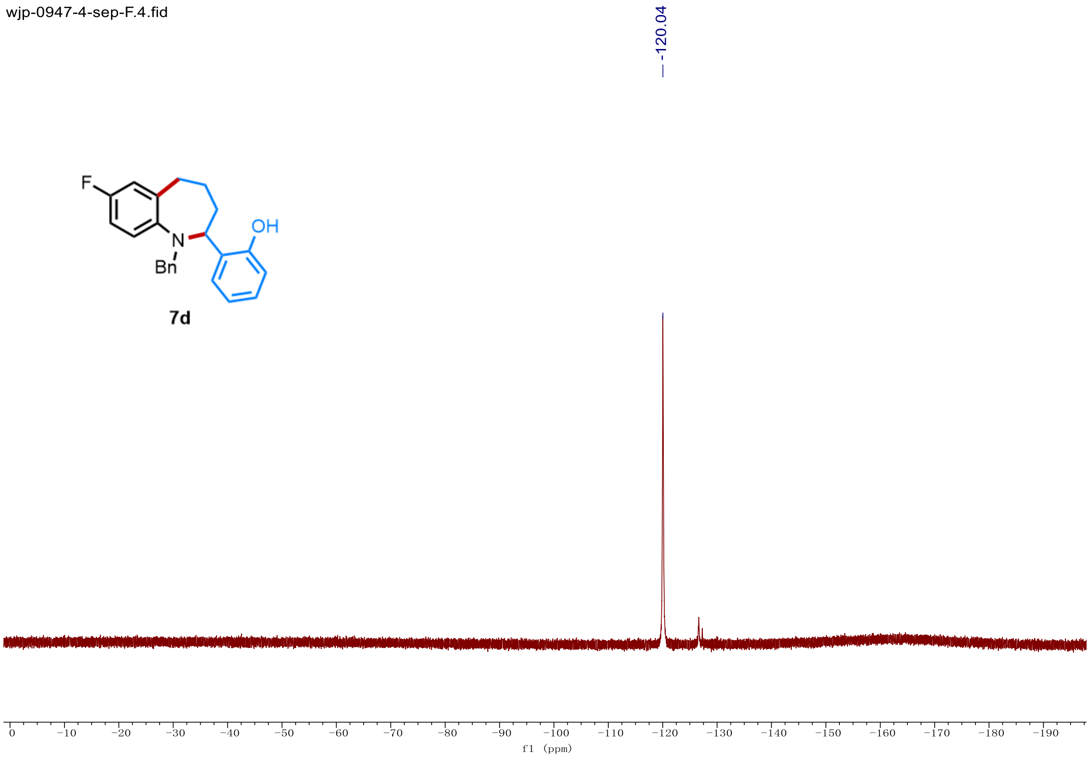

Supplementary Figure 164. ^{19}F NMR (375 MHz, acetone-d_6) spectrum of 7d.

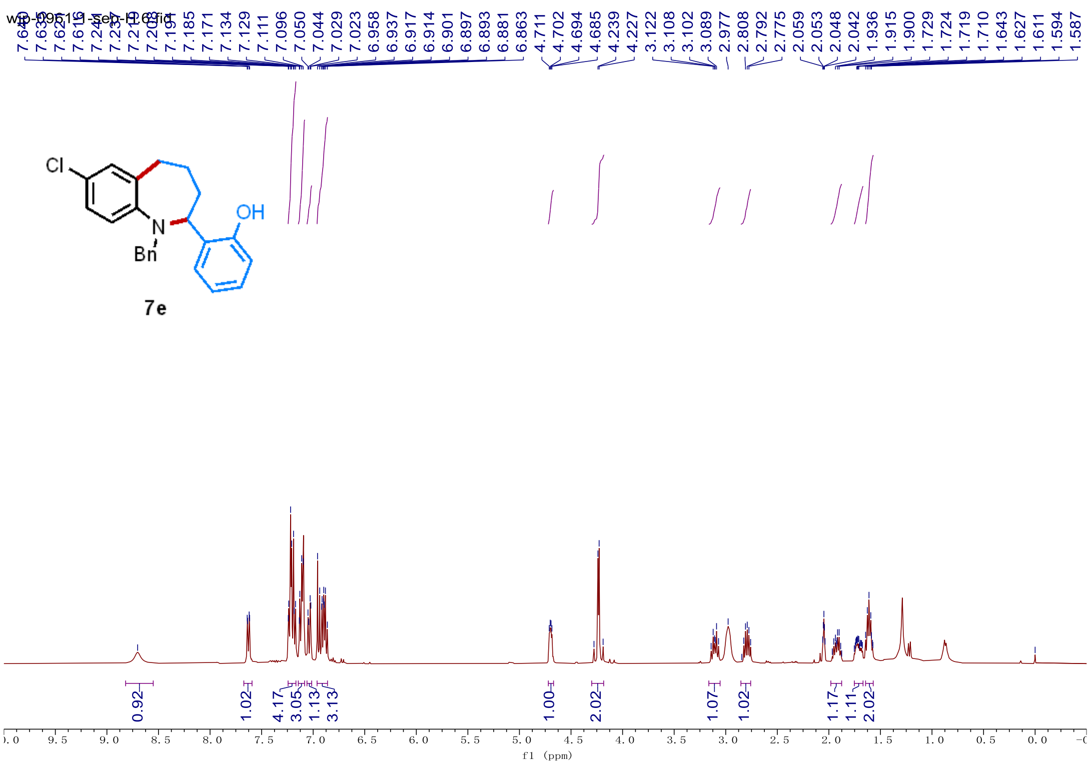

Supplementary Figure 165. ^{1}H NMR (400 MHz, acetone-d_6) spectrum of 7e.

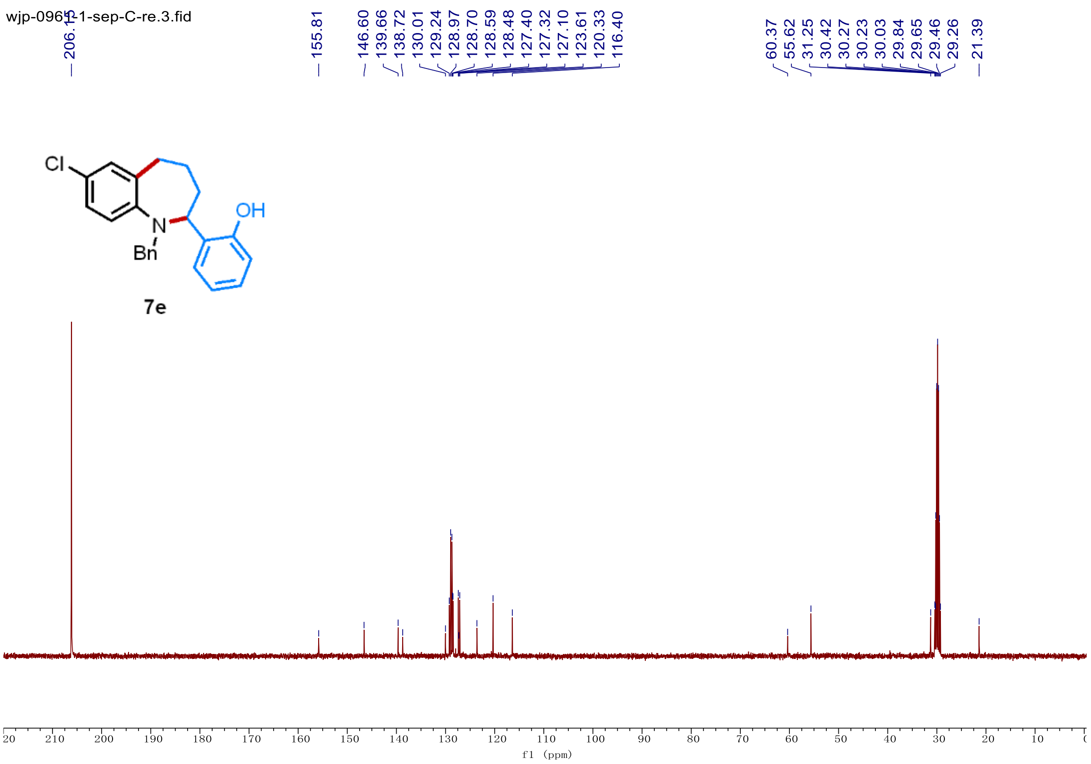

Supplementary Figure 166. ^{13}C NMR (100 MHz, acetone-d_6) spectrum of 7e.

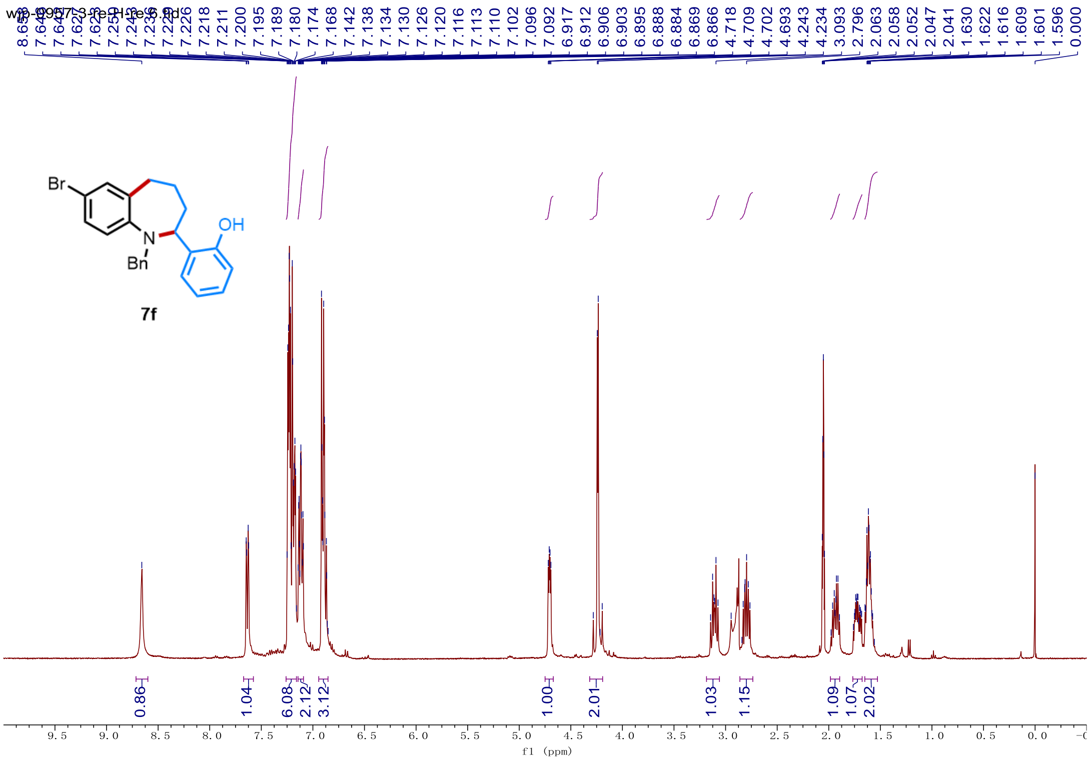

Supplementary Figure 167 ^{1}H NMR (400 MHz, acetone-d_6) spectrum of 7f.

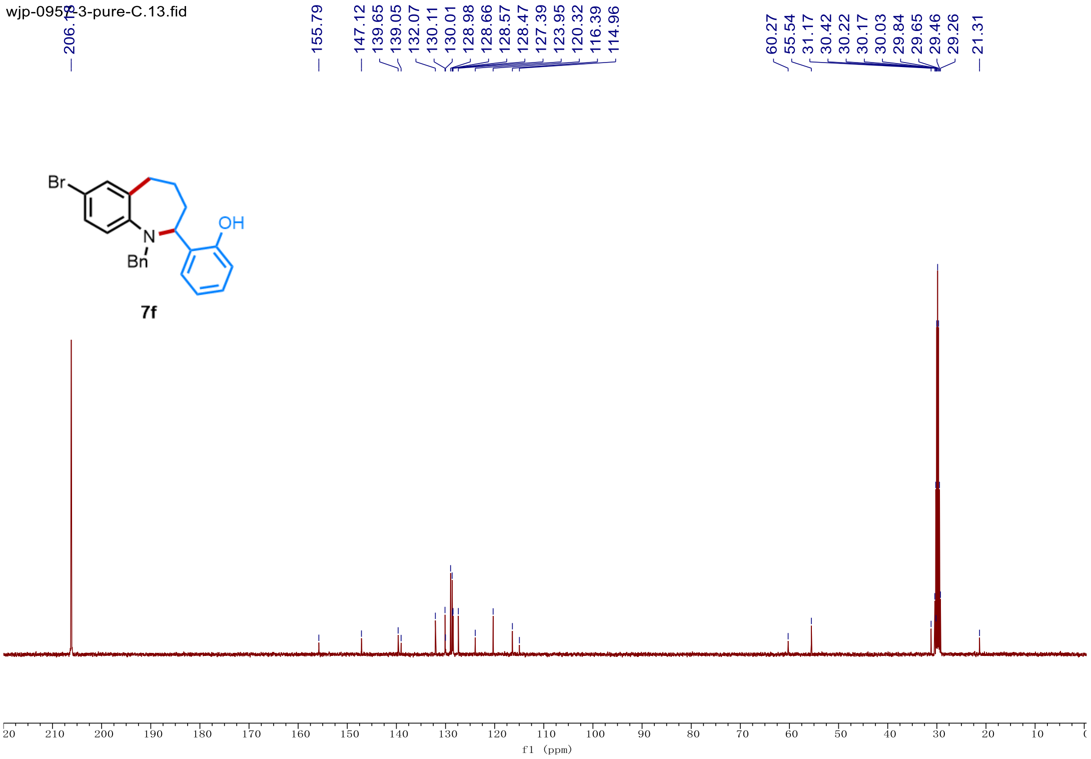

Supplementary Figure 168. ^{13}C NMR (100 MHz, acetone-d_6) spectrum of 7f.

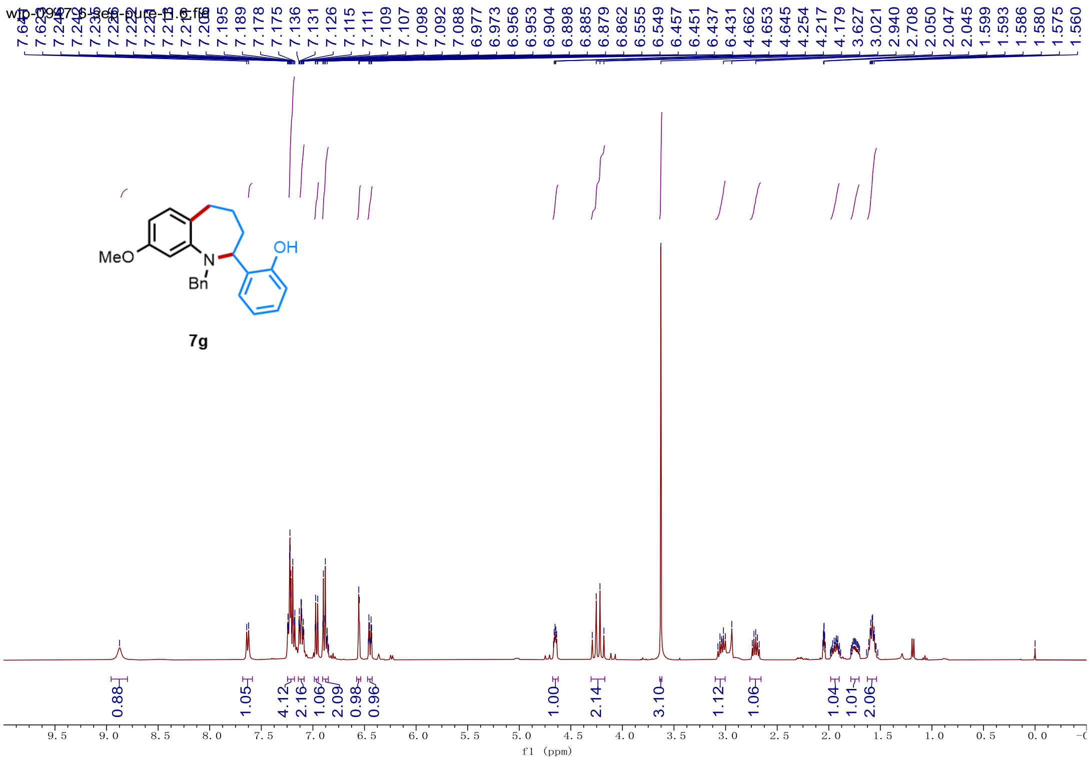

Supplementary Figure 169. ^{1}H NMR (400 MHz, acetone-d_6) spectrum of 7g.

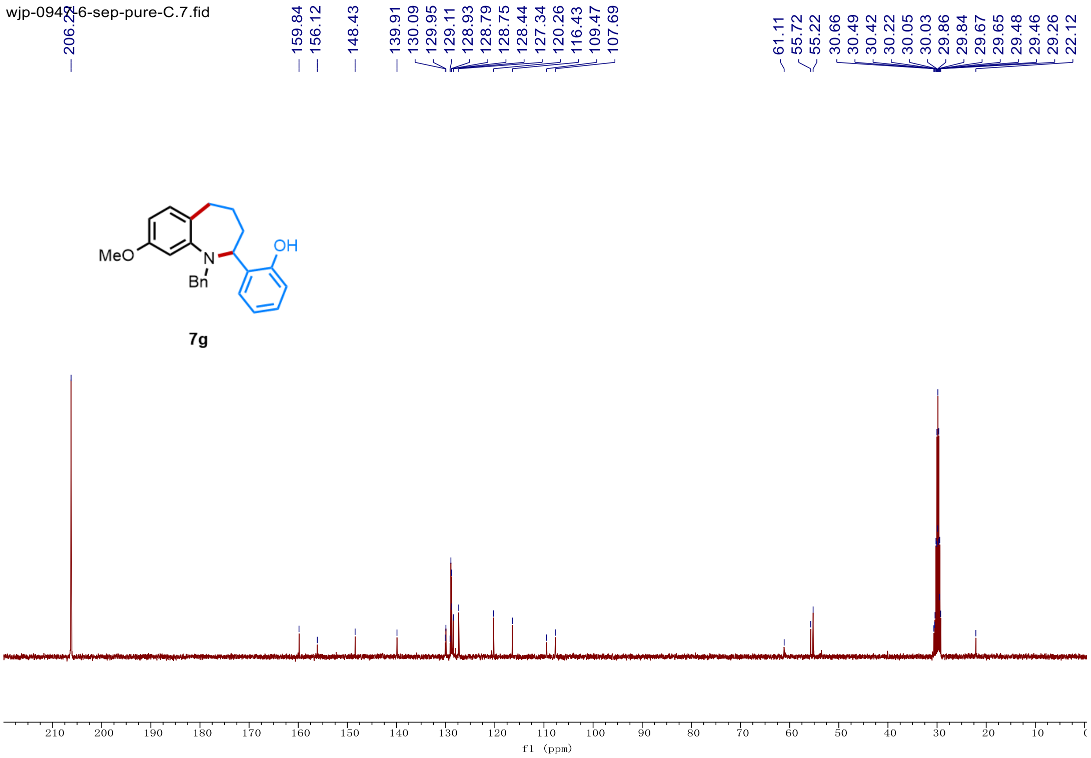

Supplementary Figure 170. ^{13}C NMR (100 MHz, acetone-d_6) spectrum of 7g.

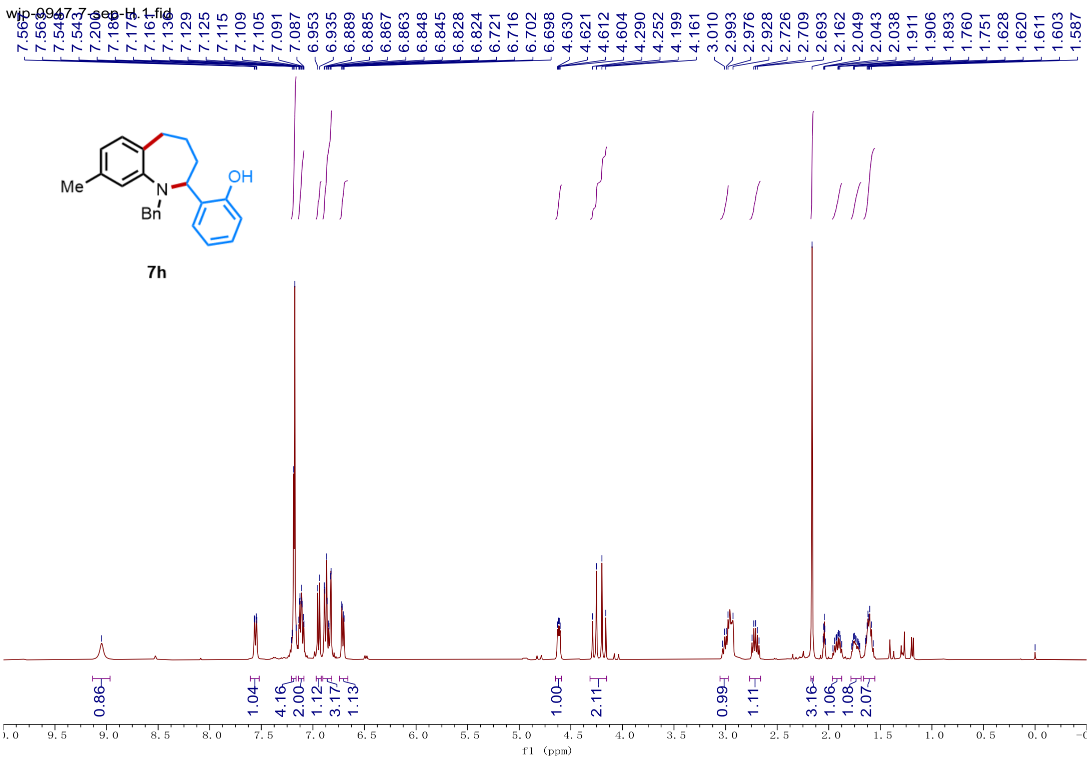

Supplementary Figure 171. ^{1}H NMR (400 MHz, acetone-d_6) spectrum of 7h.

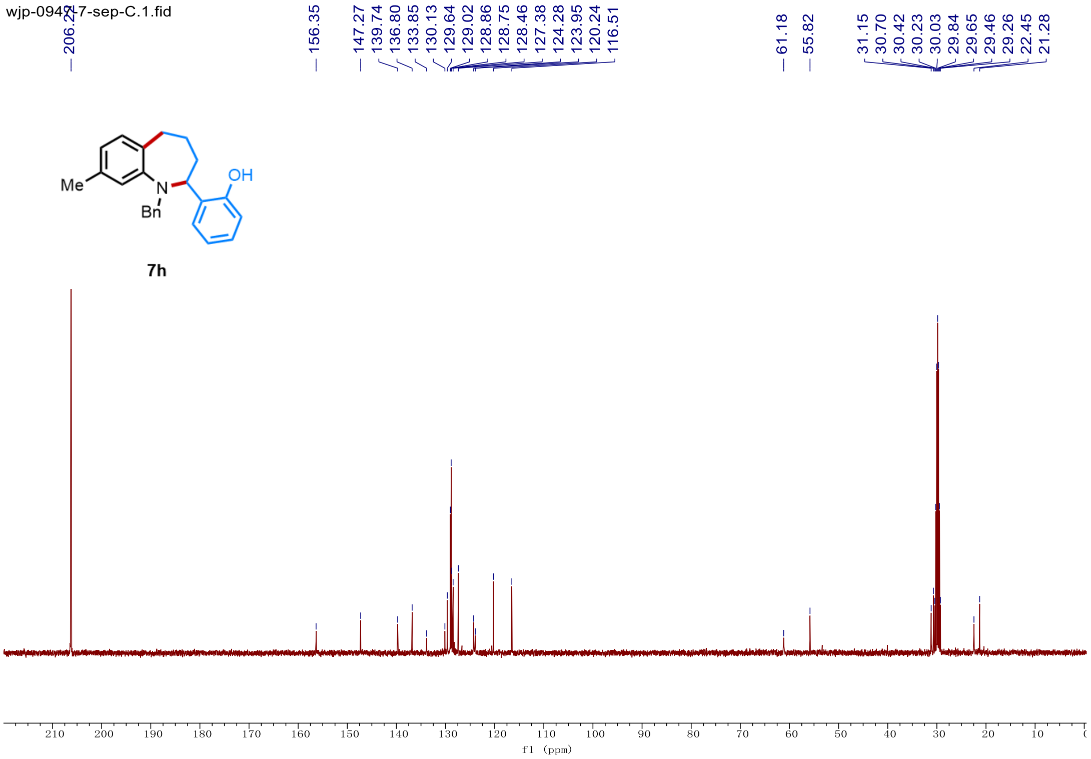

Supplementary Figure 172. ^{13}C NMR (100 MHz, acetone-d_6) spectrum of 7h.

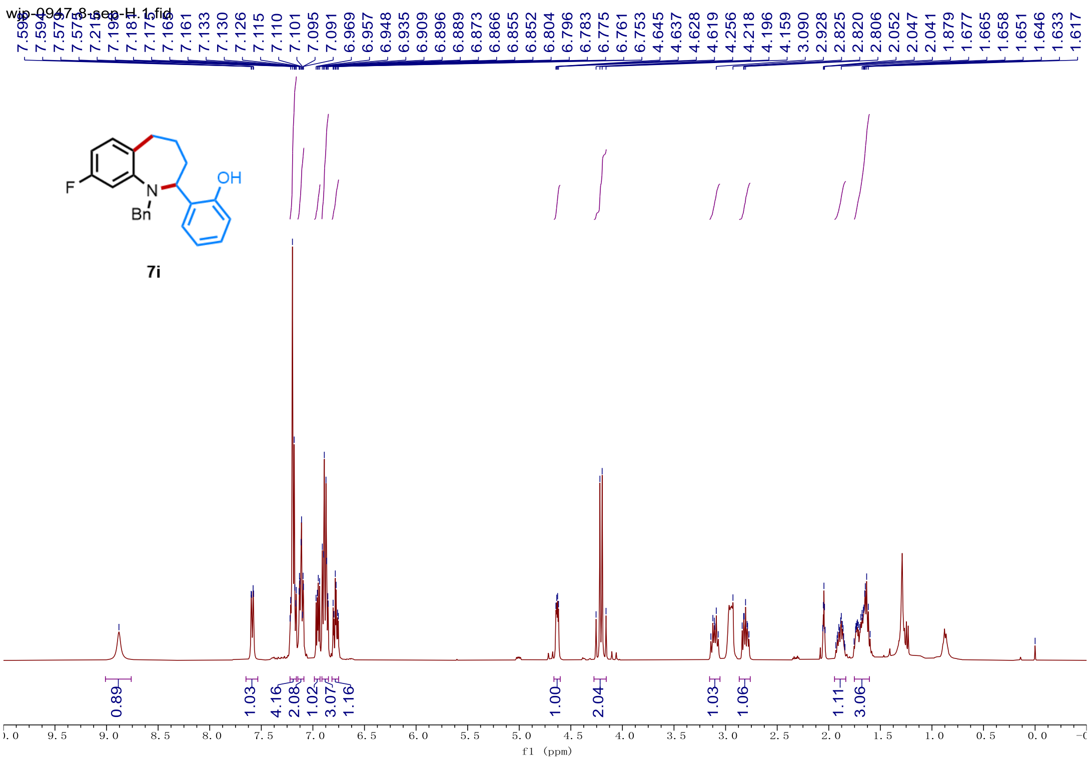

Supplementary Figure 173. **^{1}H NMR (400 MHz, acetone-d_6) spectrum of 7i.**

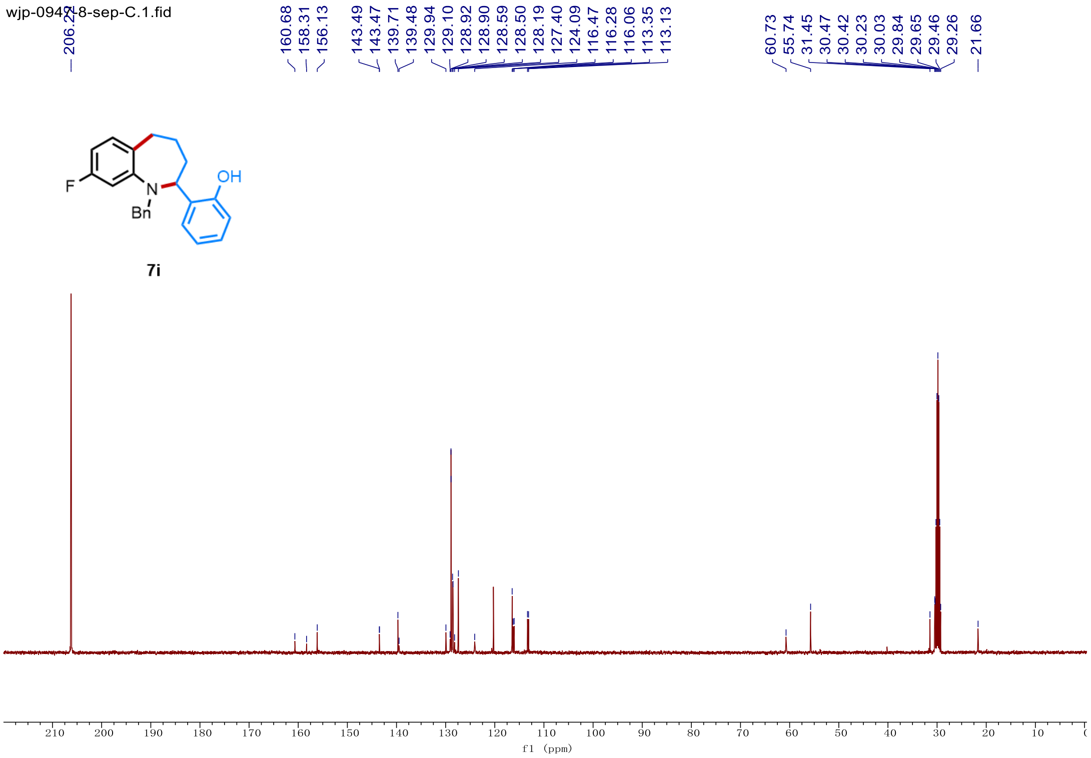

Supplementary Figure 174. **^{13}C NMR (100 MHz, acetone-d_6) spectrum of 7i.**

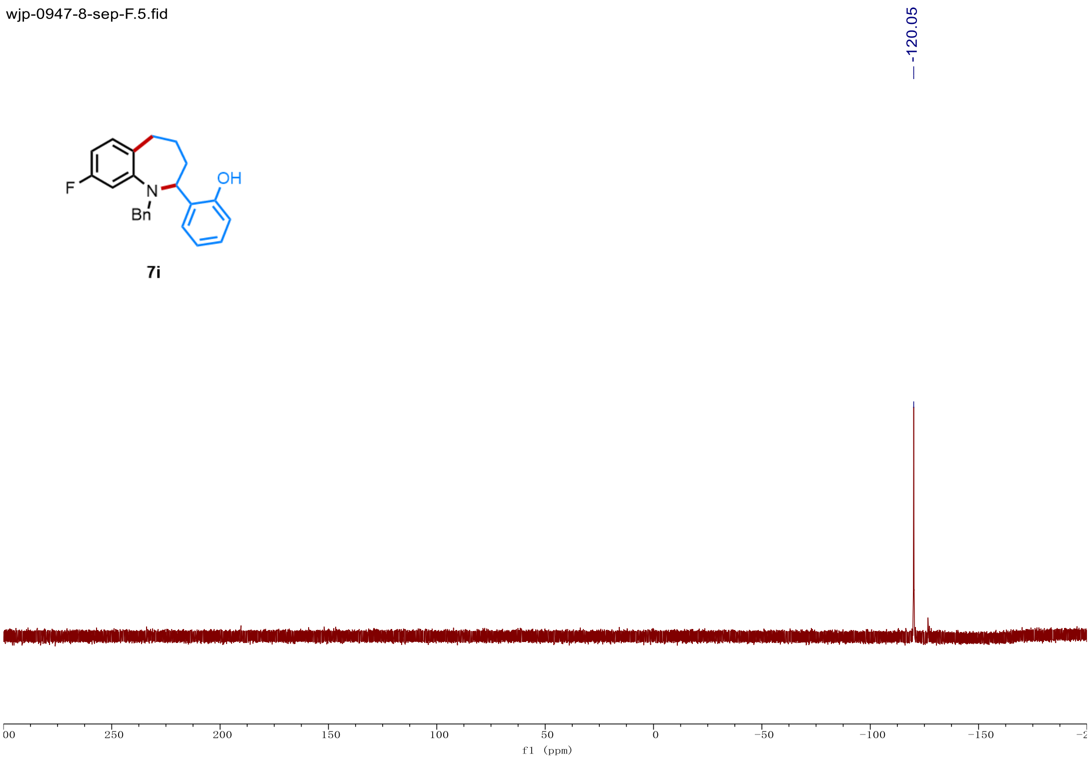

Supplementary Figure 175. ^{19}**F NMR (375 MHz, acetone-*d*$_6$) spectrum of 7i.**

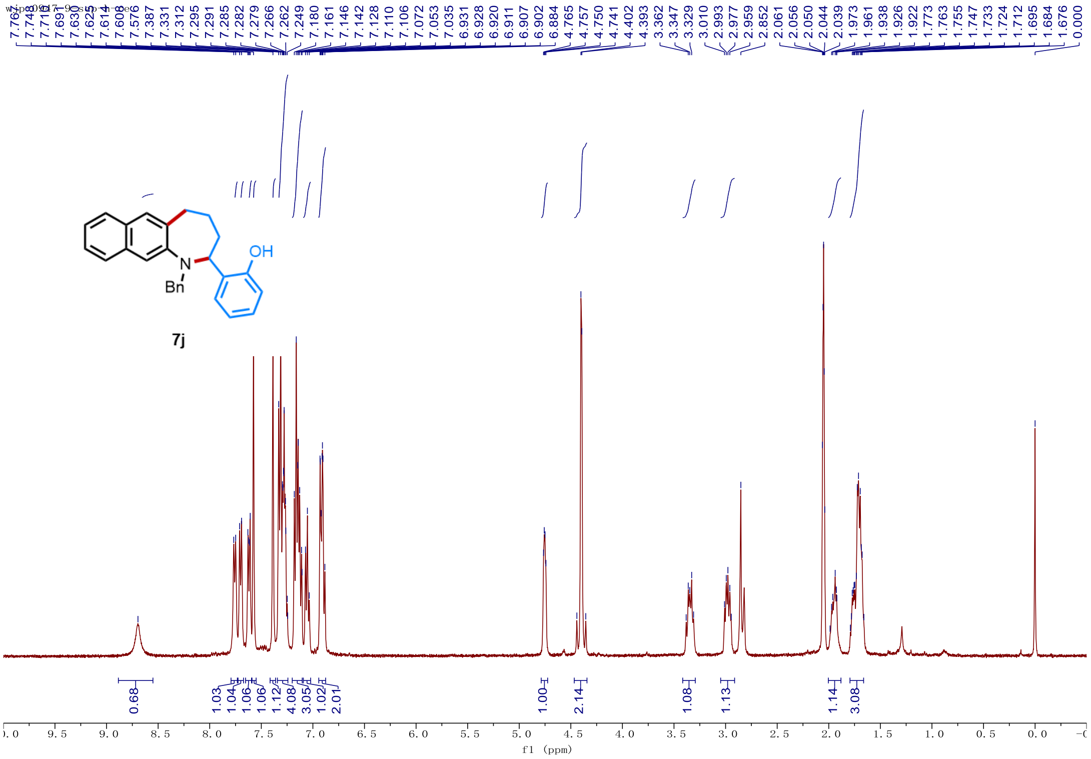

Supplementary Figure 176. ^{1}**H NMR (400 MHz, acetone-*d*$_6$) spectrum of 7j.**

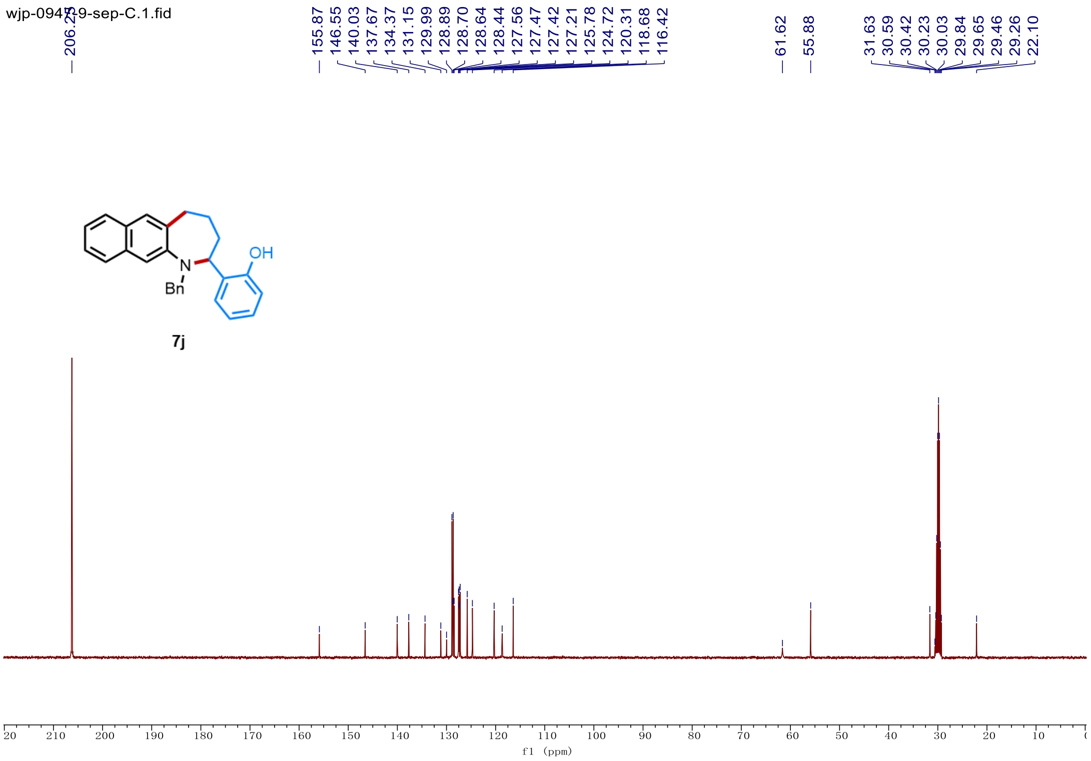

Supplementary Figure 177. ^{13}C NMR (100 MHz, acetone-d_6) spectrum of 7j.

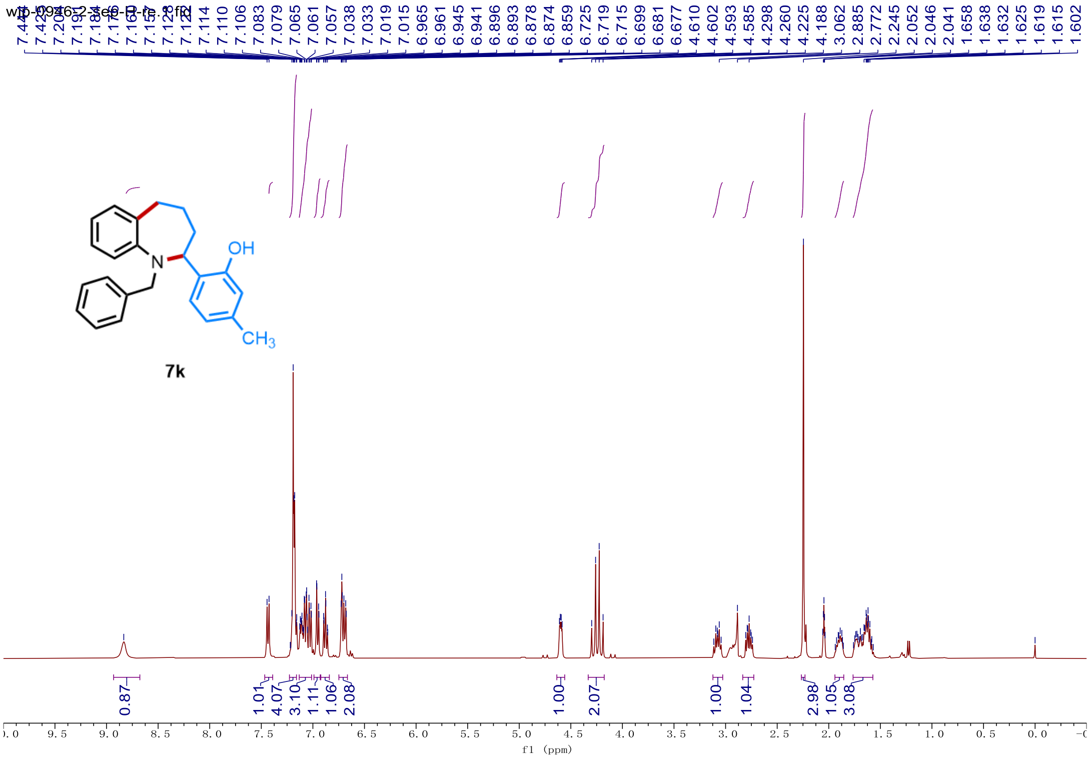

Supplementary Figure 178. ^{1}H NMR (400 MHz, acetone-d_6) spectrum of 7k.

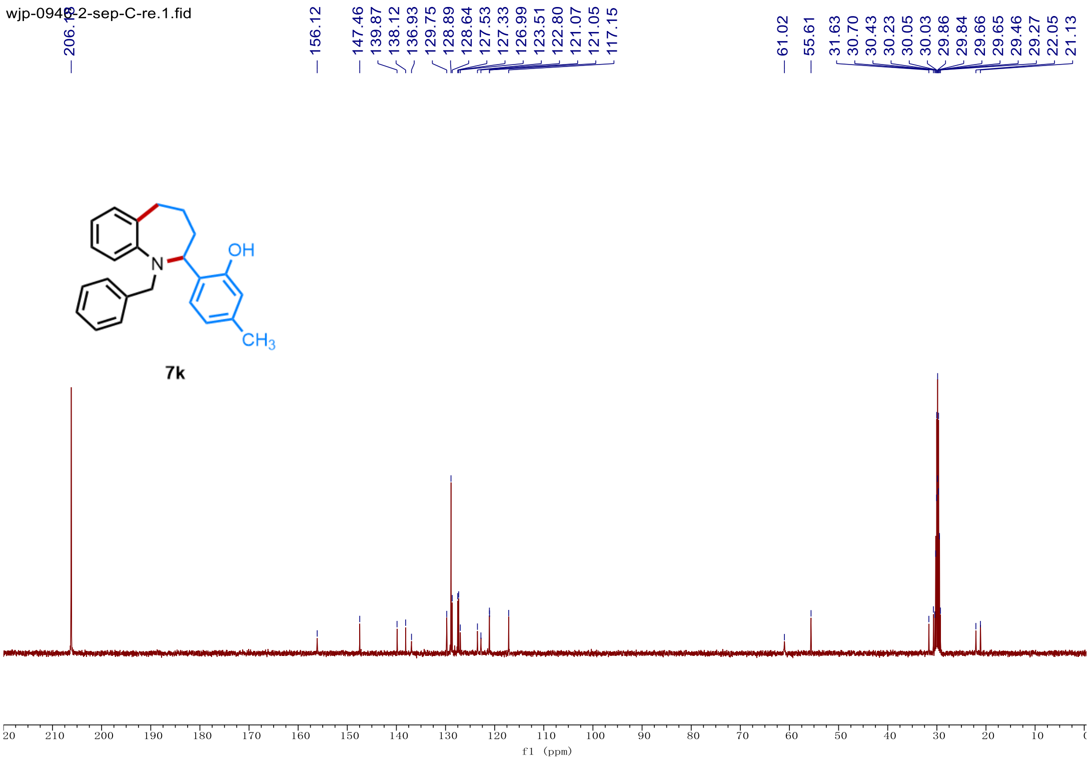

Supplementary Figure 179. ^{13}C NMR (100 MHz, acetone-d_6) spectrum of 7k.

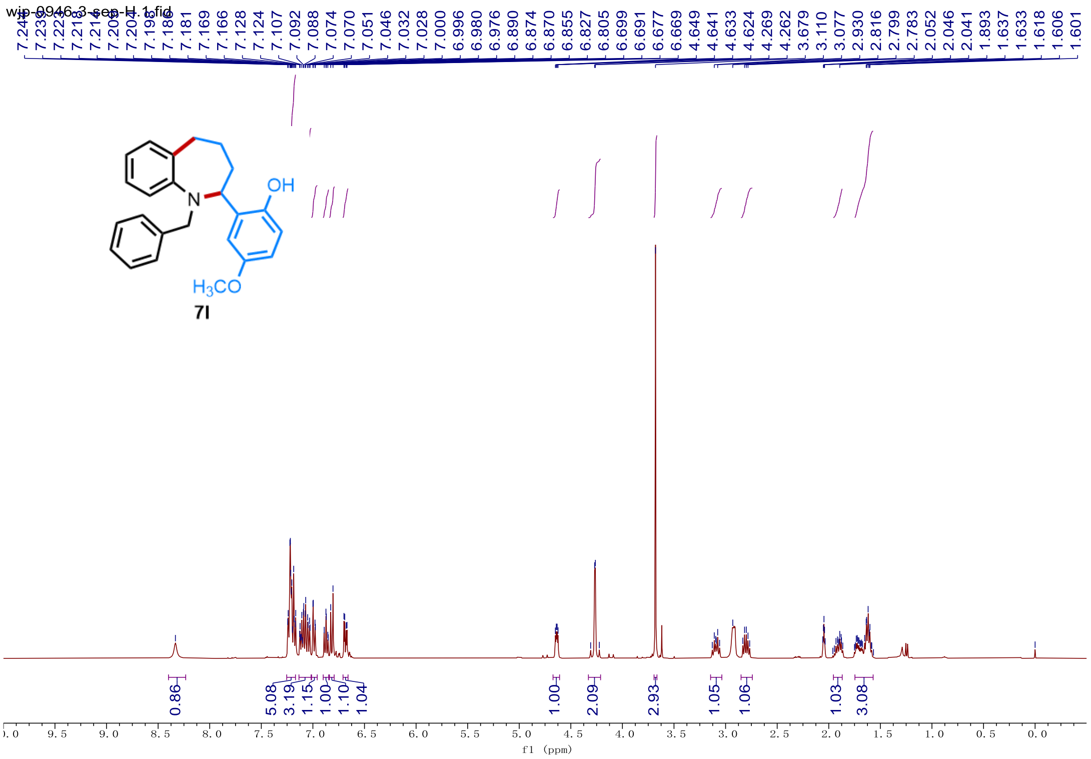

Supplementary Figure 180. ^{1}H NMR (400 MHz, acetone-d_6) spectrum of 7l.

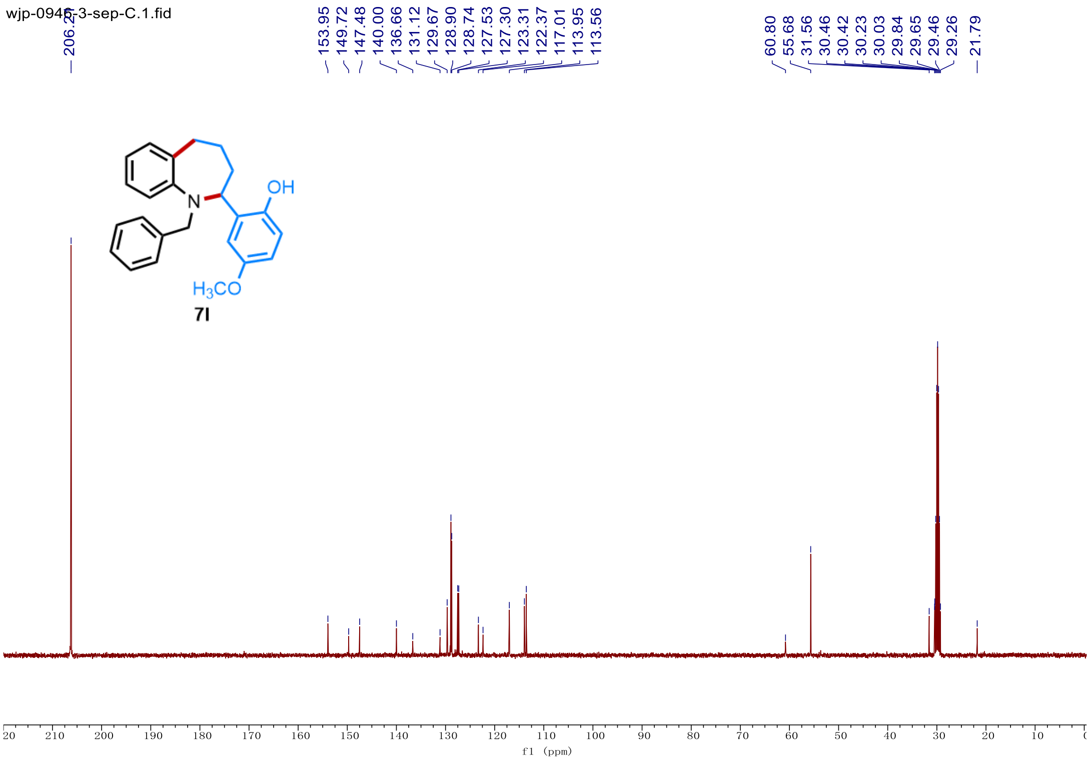

Supplementary Figure 181. ^{13}**C NMR (100 MHz, acetone-*d*$_6$) spectrum of 7l.**

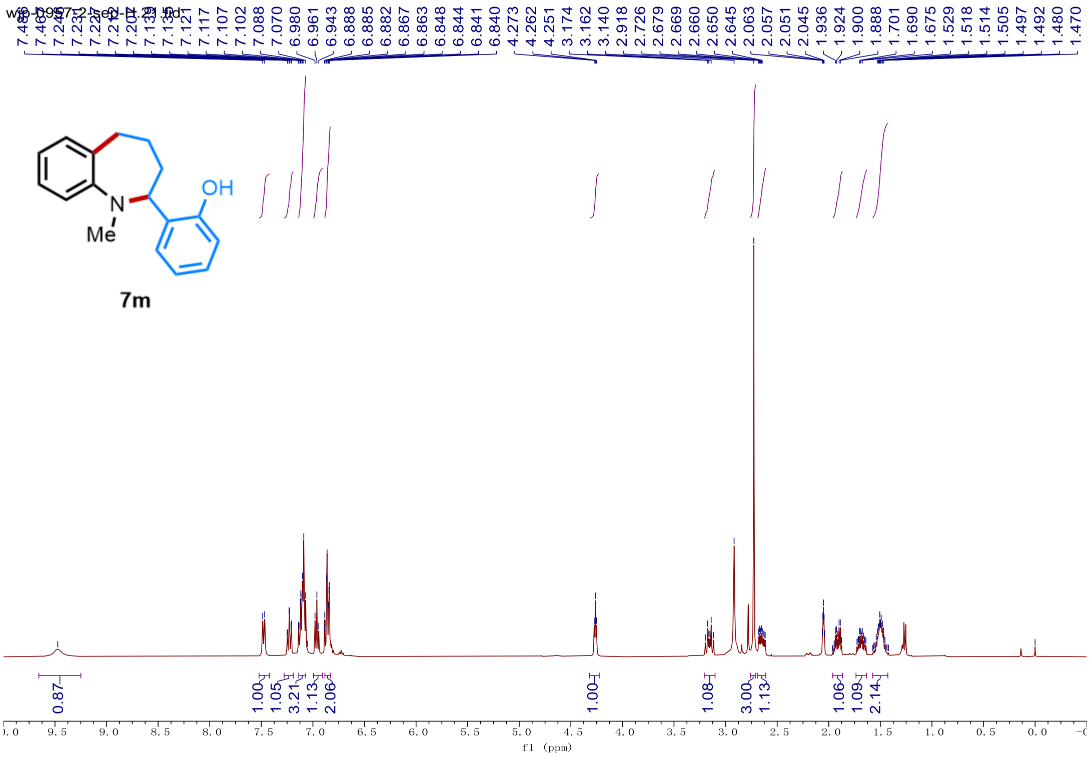

Supplementary Figure 182. ^{1}**H NMR (400 MHz, acetone-*d*$_6$) spectrum of 7m.**

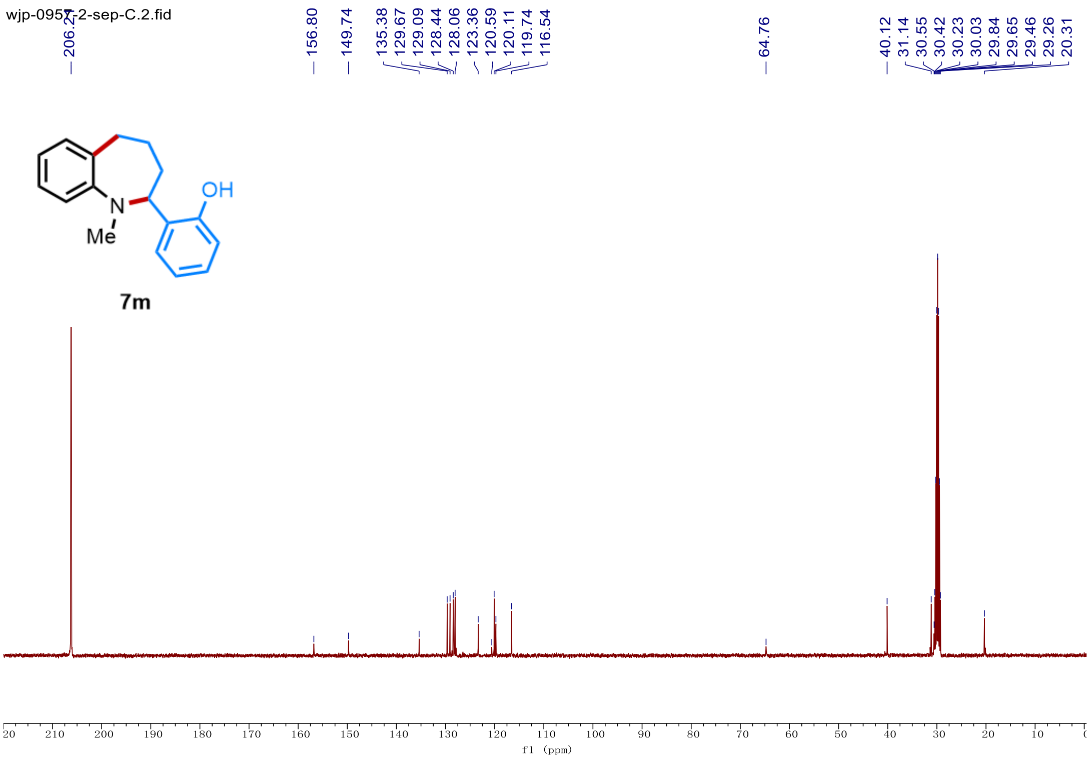

Supplementary Figure 183. ^{13}**C NMR (100 MHz, acetone-*d*$_6$) spectrum of 7m.**

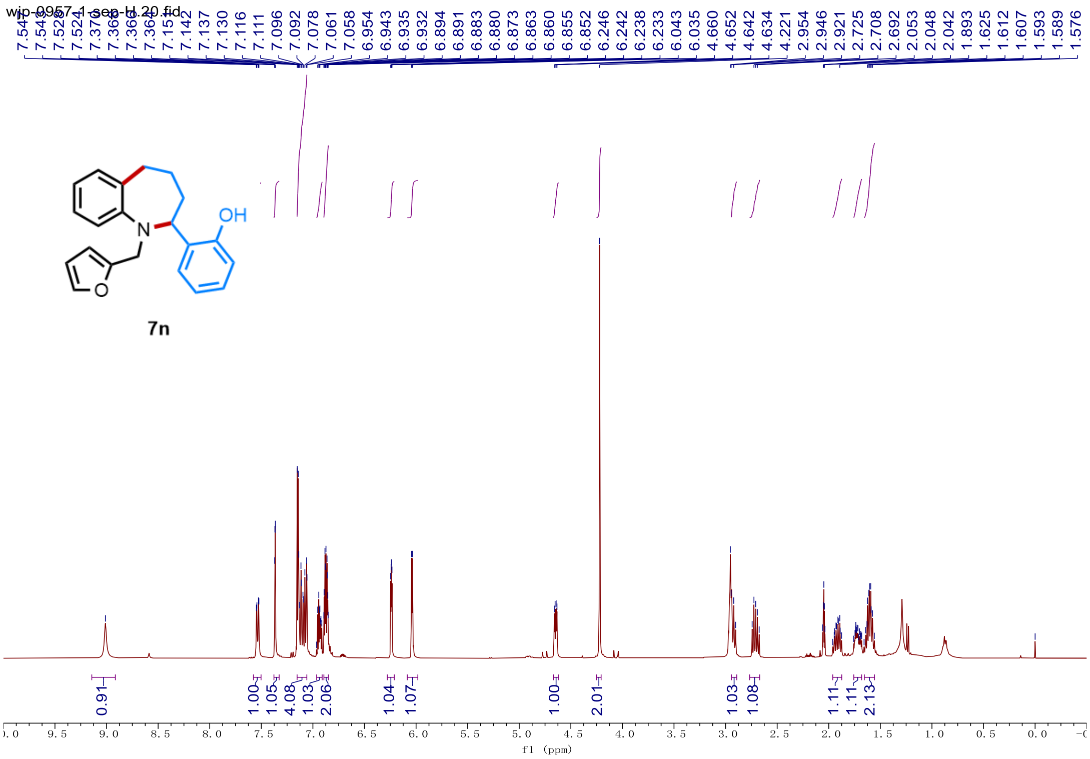

Supplementary Figure 184. ^{1}**H NMR (400 MHz, acetone-*d*$_6$) spectrum of 7n.**

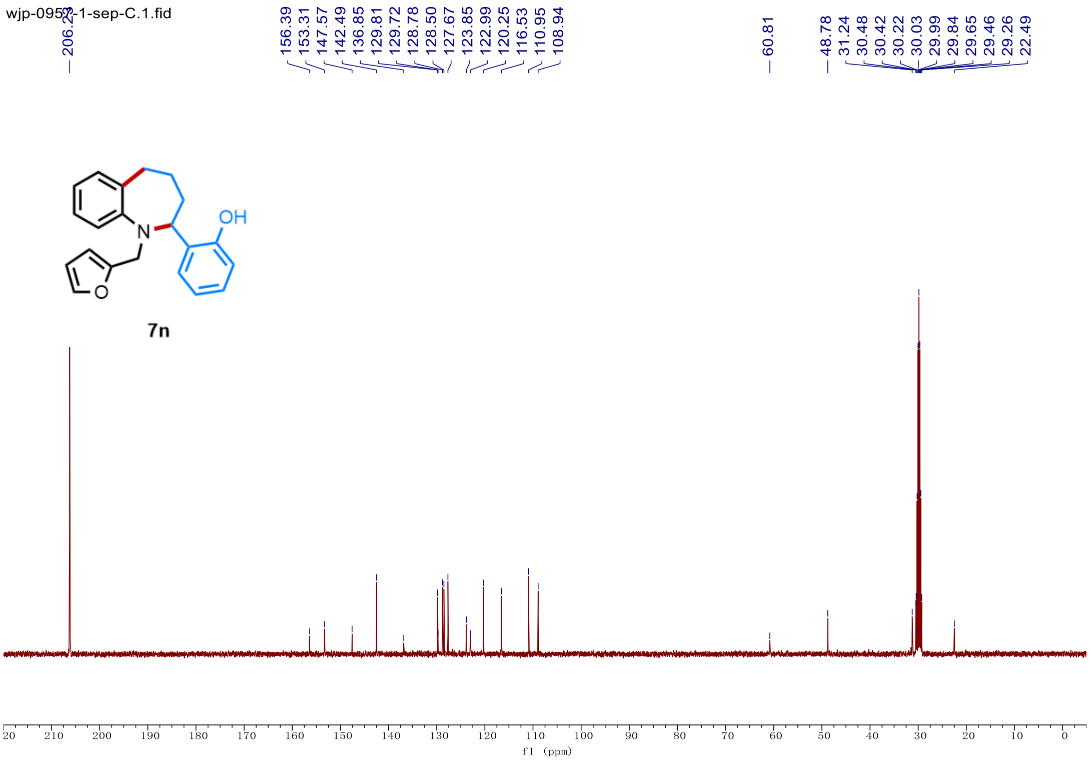

Supplementary Figure 185. ^{13}C NMR (100 MHz, acetone-d_6) spectrum of 7n.

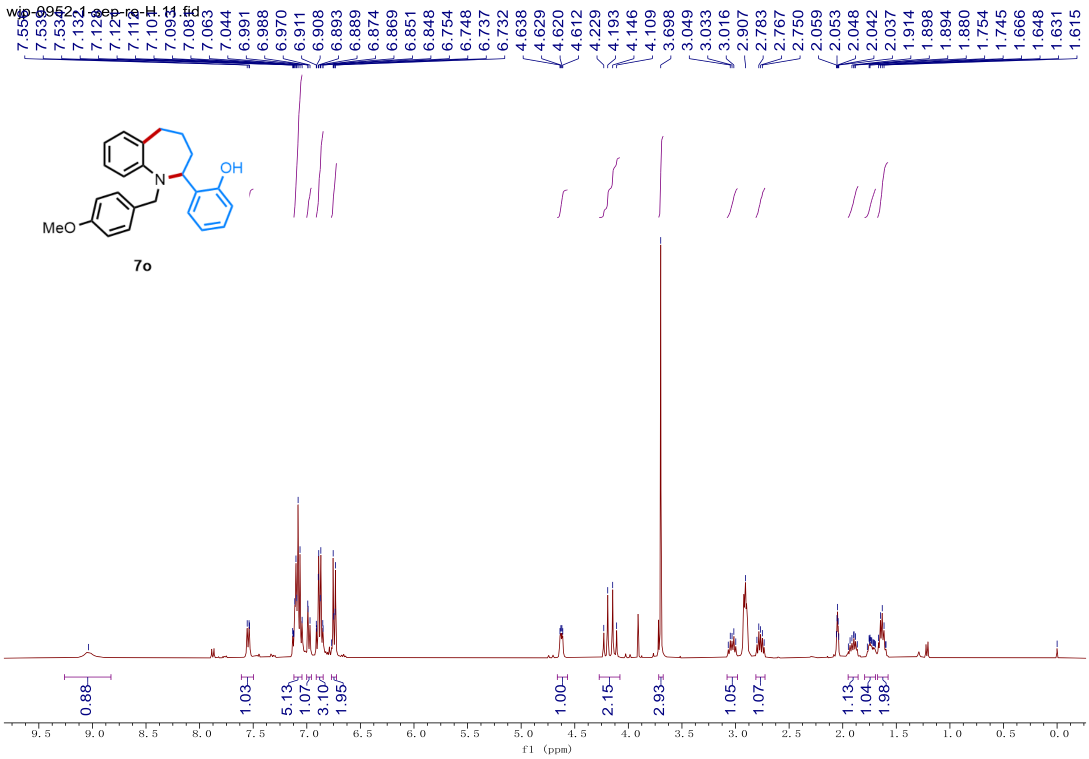

Supplementary Figure 186. ^{1}H NMR (400 MHz, acetone-d_6) spectrum of 7o.

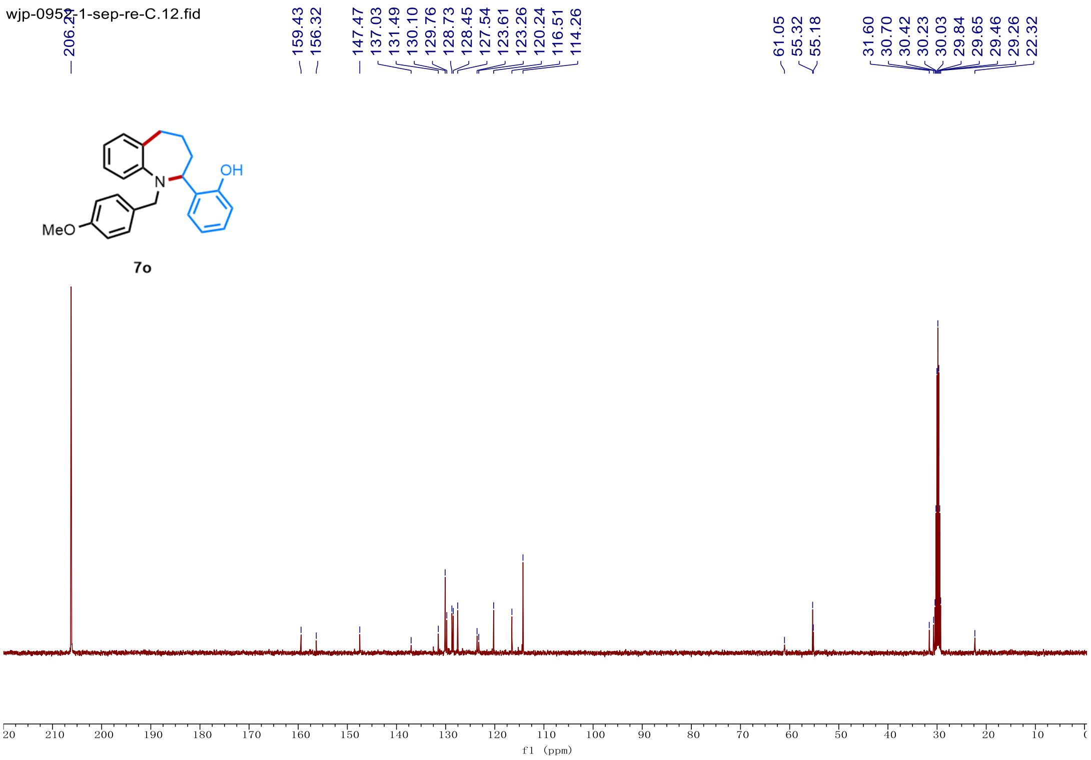

Supplementary Figure 187. ^{13}C NMR (100 MHz, acetone-d_6) spectrum of 7o.

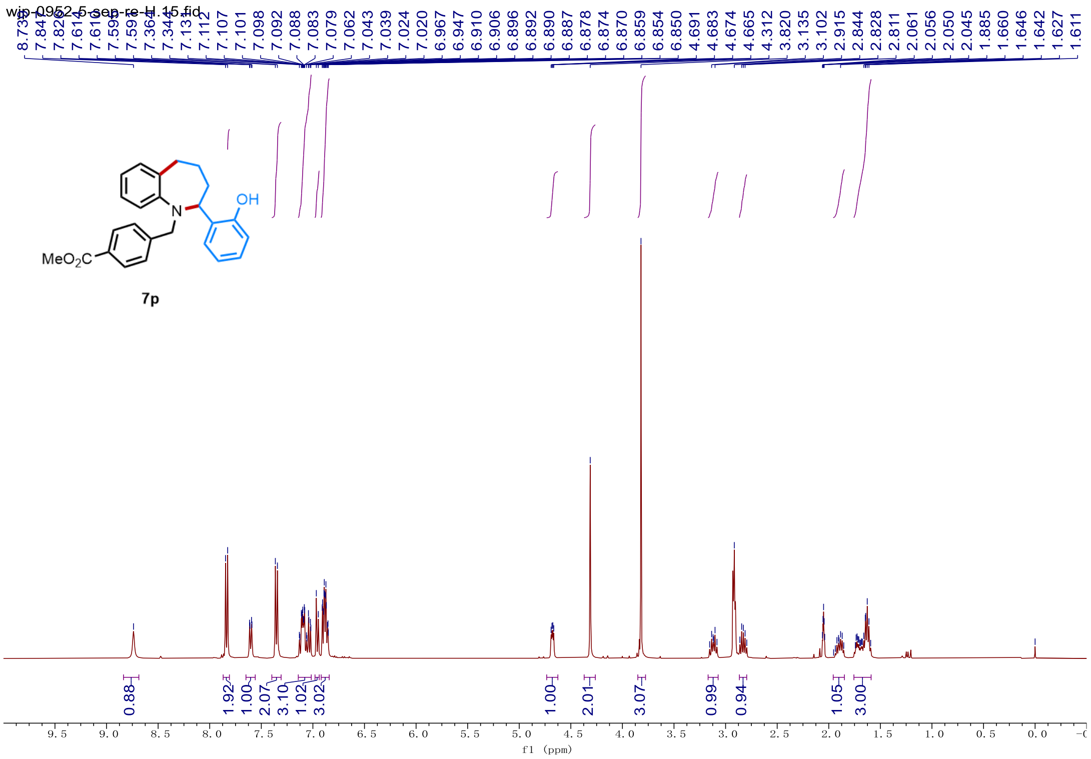

Supplementary Figure 188. ^{1}H NMR (400 MHz, acetone-d_6) spectrum of 7p.

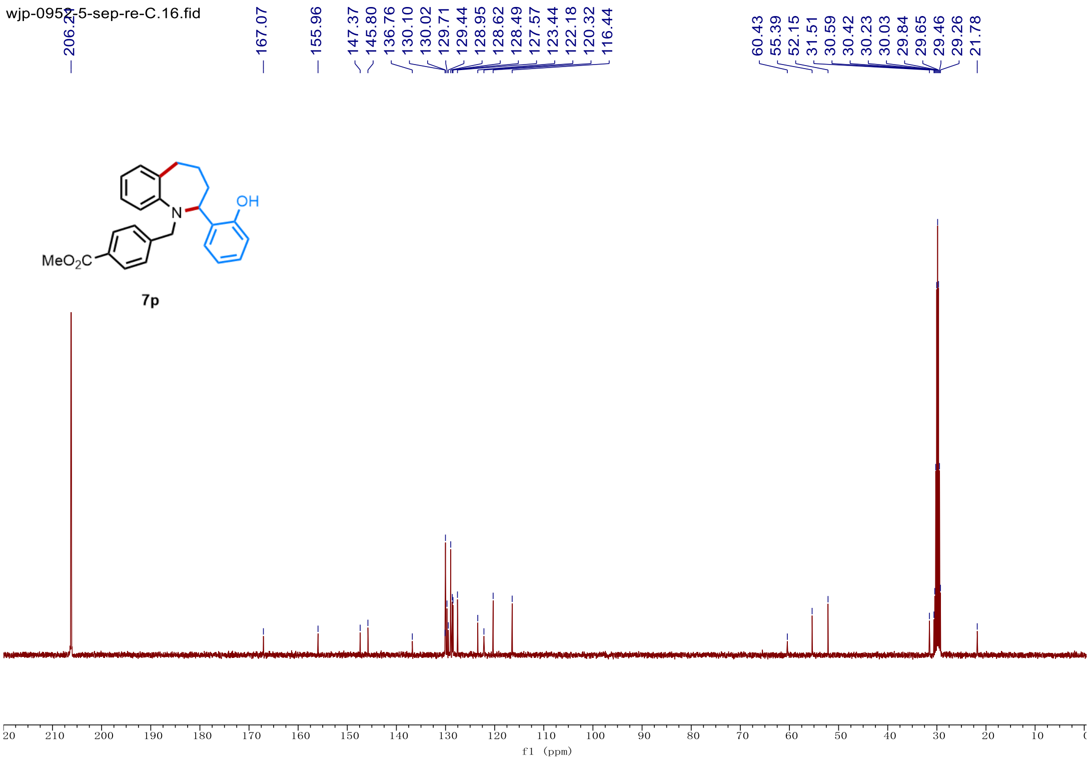

Supplementary Figure 189. ^{13}C NMR (100 MHz, acetone-d_6) spectrum of 7p.

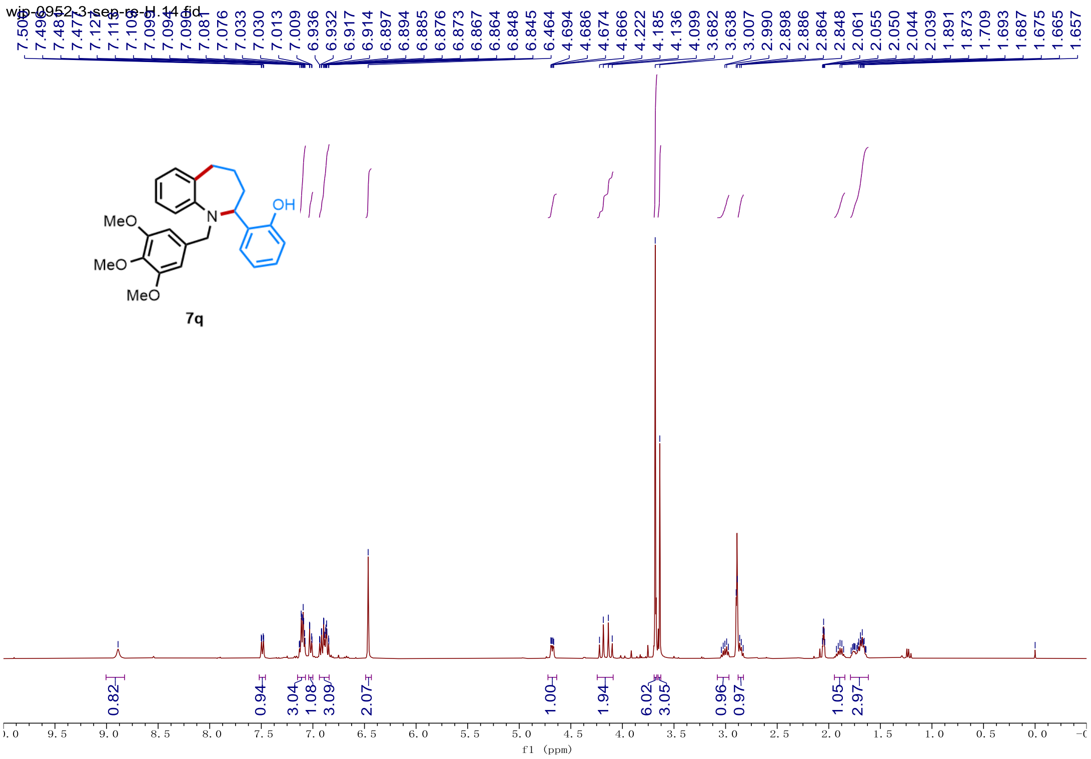

Supplementary Figure 190. ^{1}H NMR (400 MHz, acetone-d_6) spectrum of 7q.

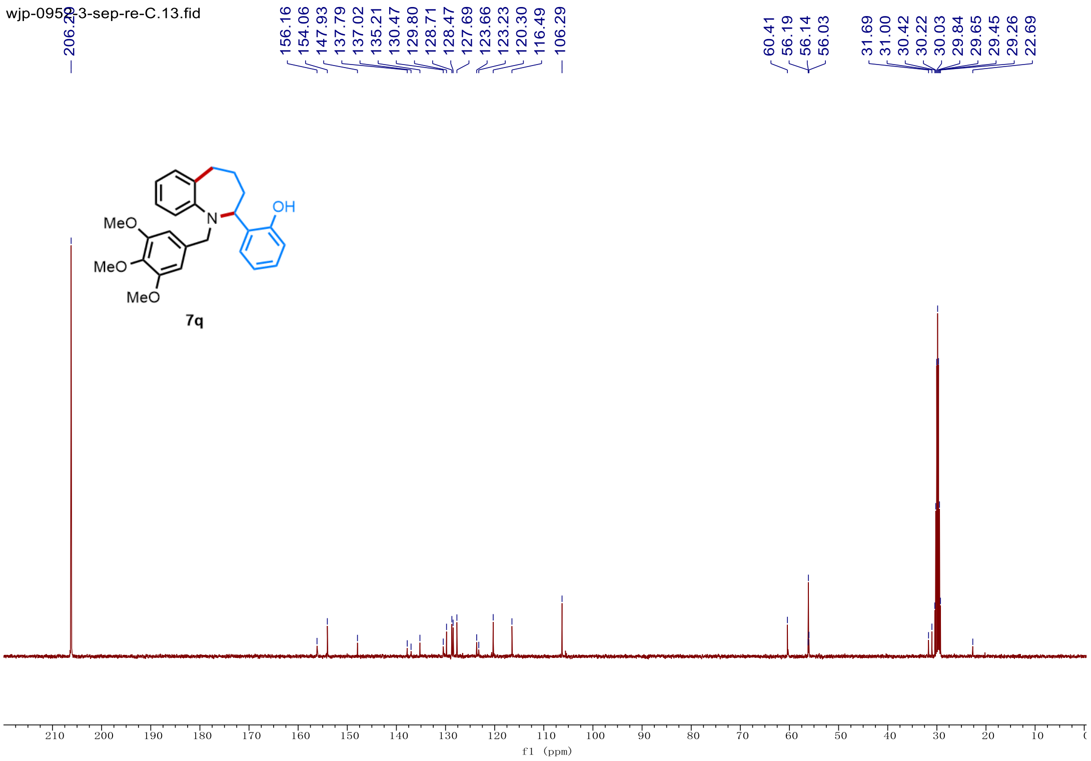

Supplementary Figure 191. ^{13}C NMR (100 MHz, acetone-d_6) spectrum of 7q.

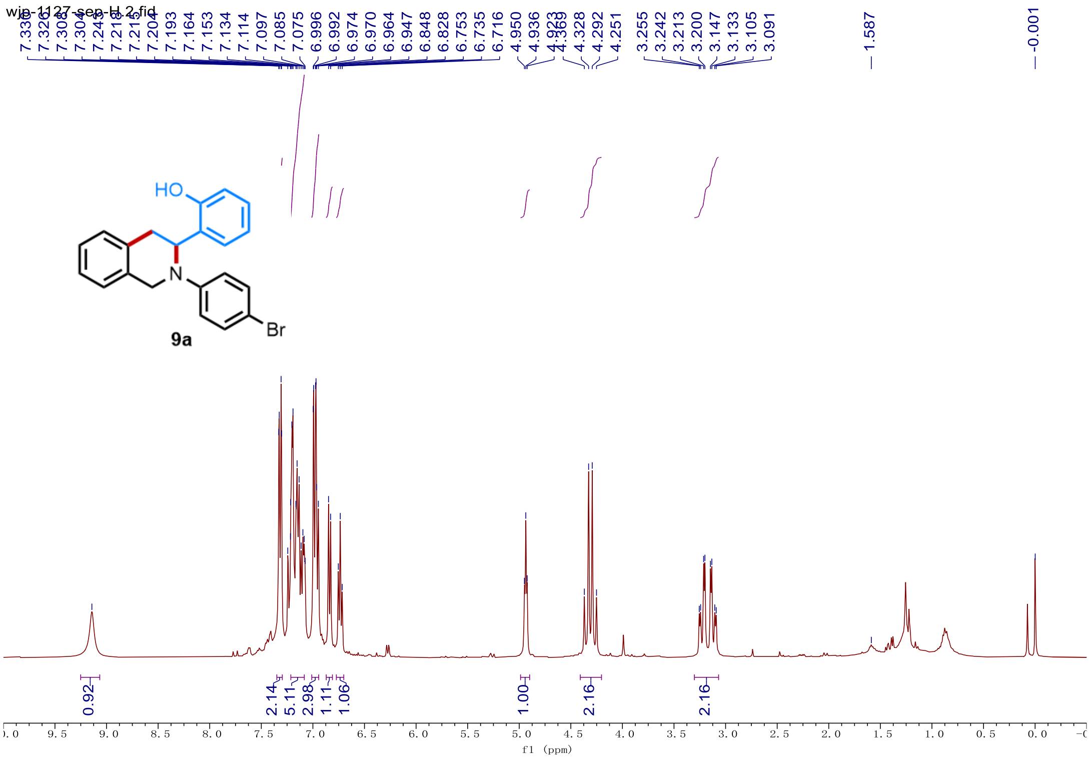

Supplementary Figure 192. ^{1}H NMR (400 MHz, $CDCl_3$) spectrum of 9a.

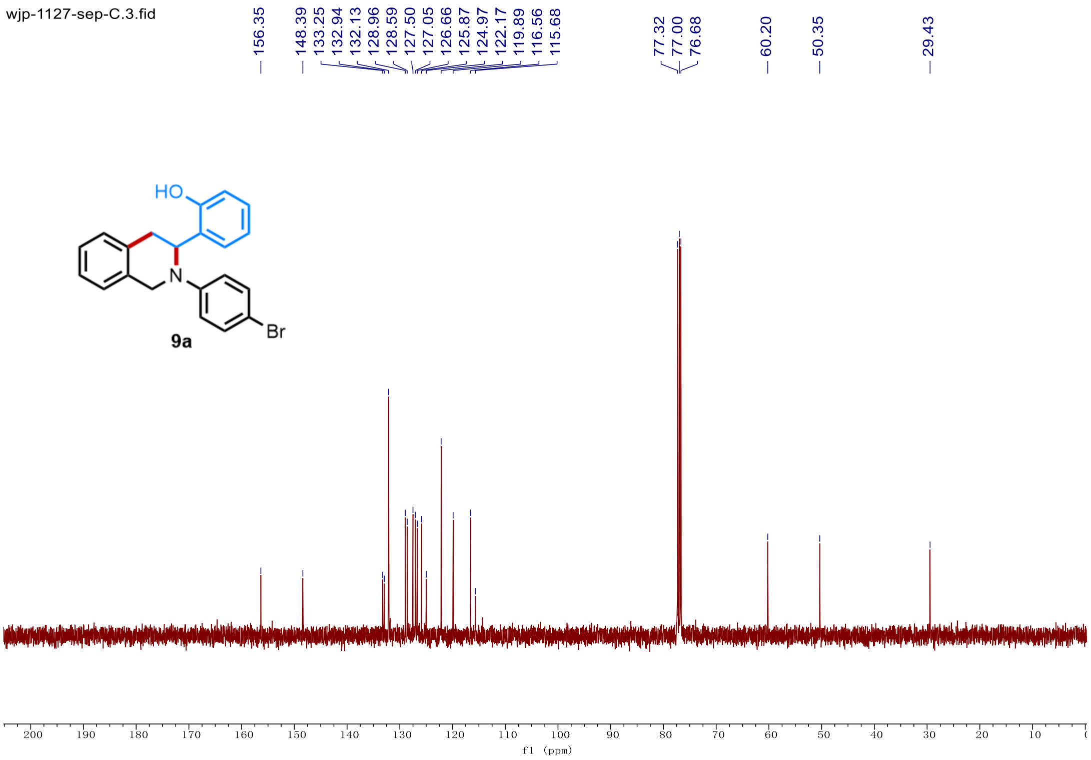

Supplementary Figure 193. ^{13}**C NMR (100 MHz, CDCl$_3$) spectrum of 9a.**

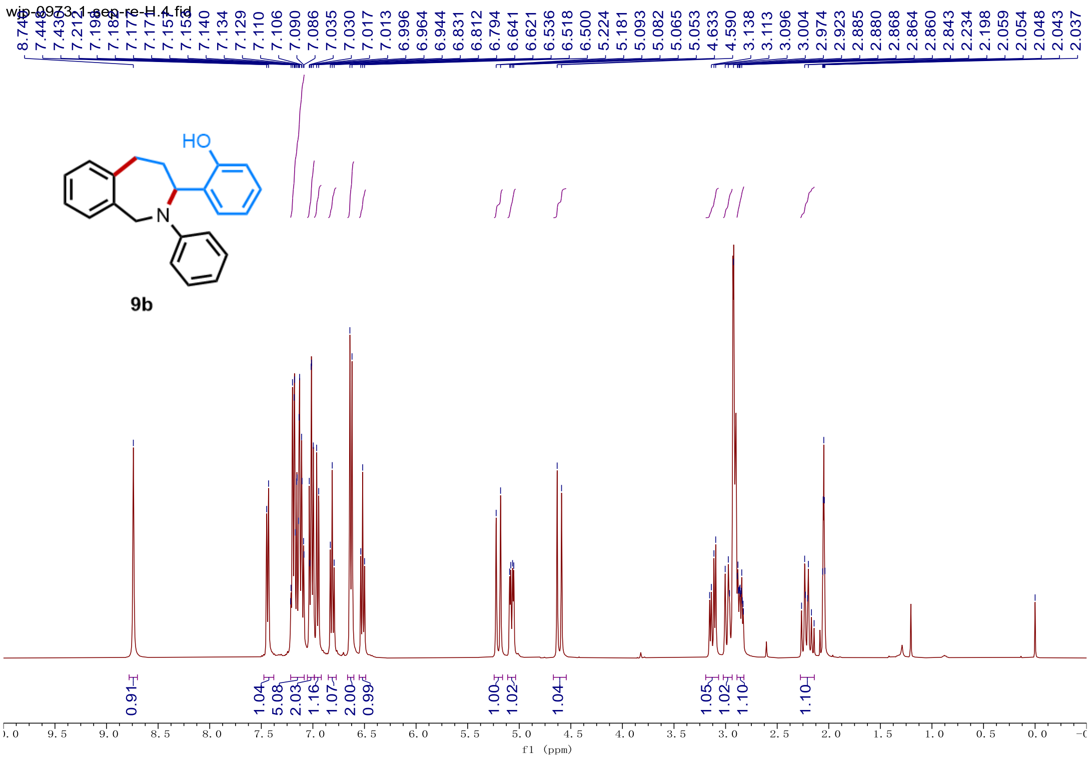

Supplementary Figure 194. ^{1}**H NMR (400 MHz, acetone-*d*$_6$) spectrum of 9b.**

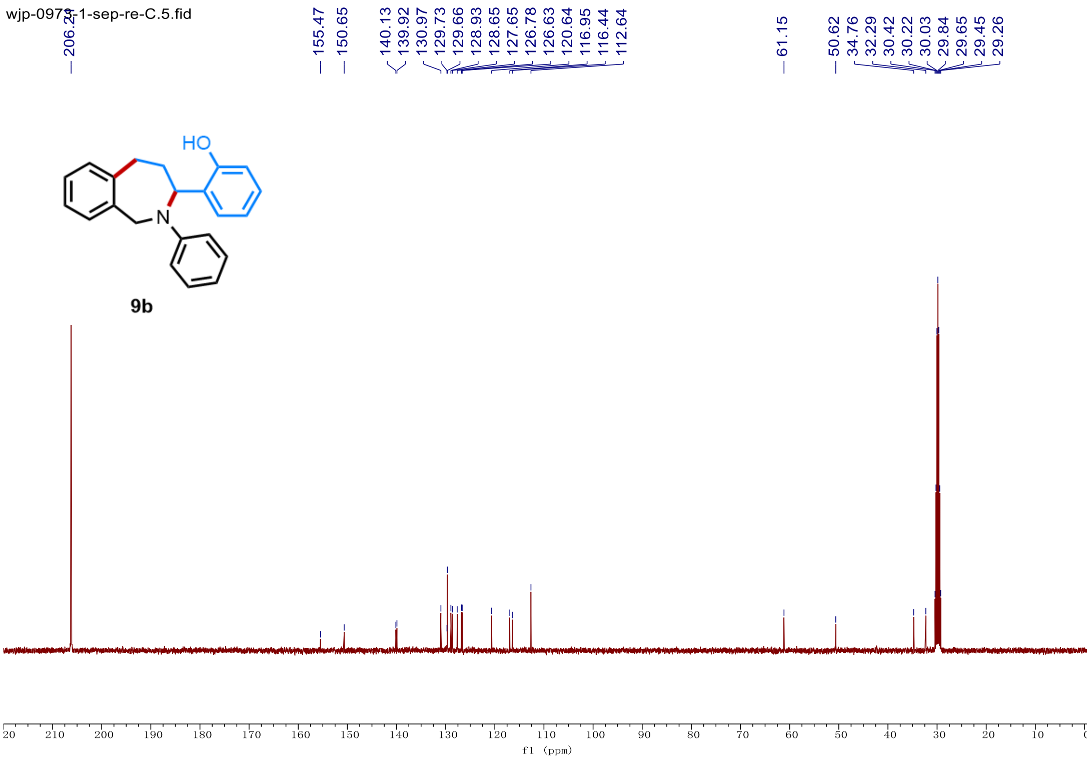

Supplementary Figure 195. ^{13}C NMR (100 MHz, acetone-d_6) spectrum of 9b.

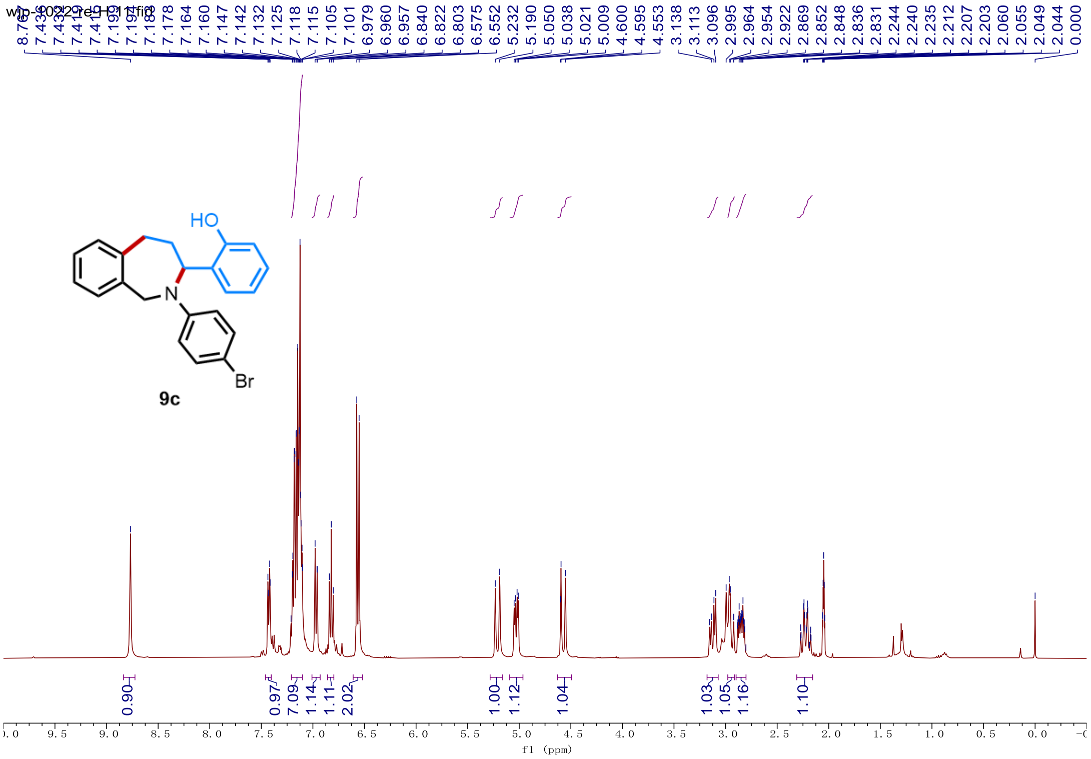

Supplementary Figure 196. ^{1}H NMR (400 MHz, acetone-d_6) spectrum of 9c.

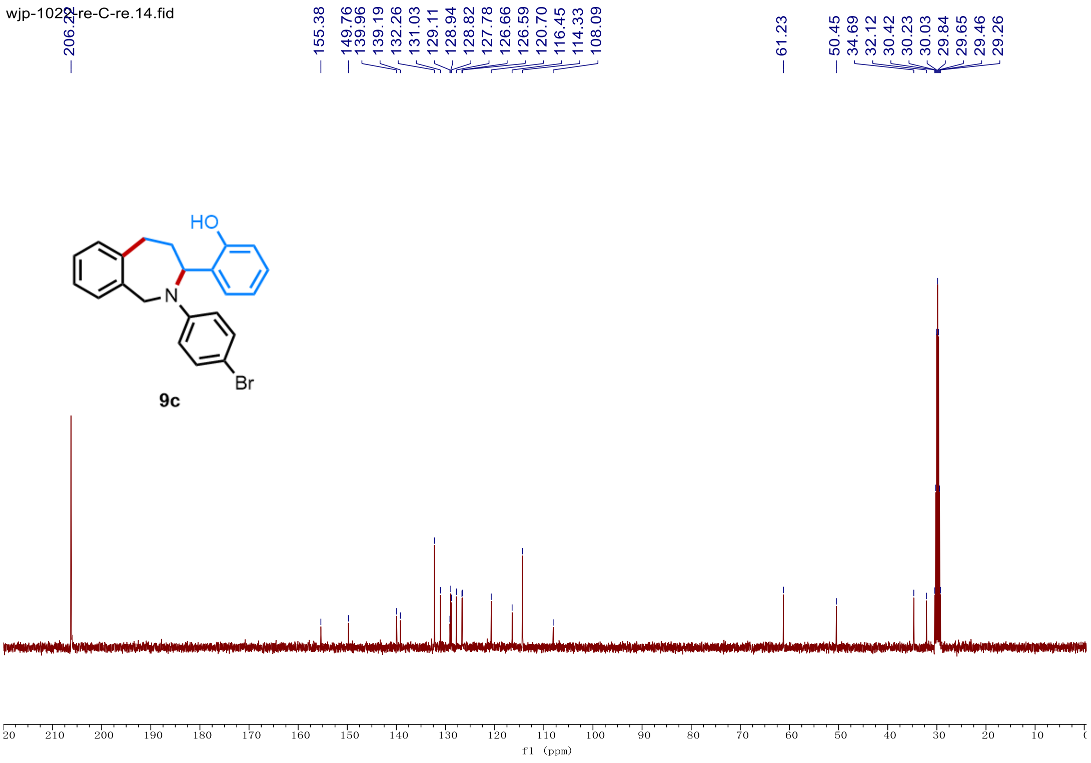

Supplementary Figure 197. ^{13}**C NMR (100 MHz, acetone-*d*$_6$) spectrum of 9c.**

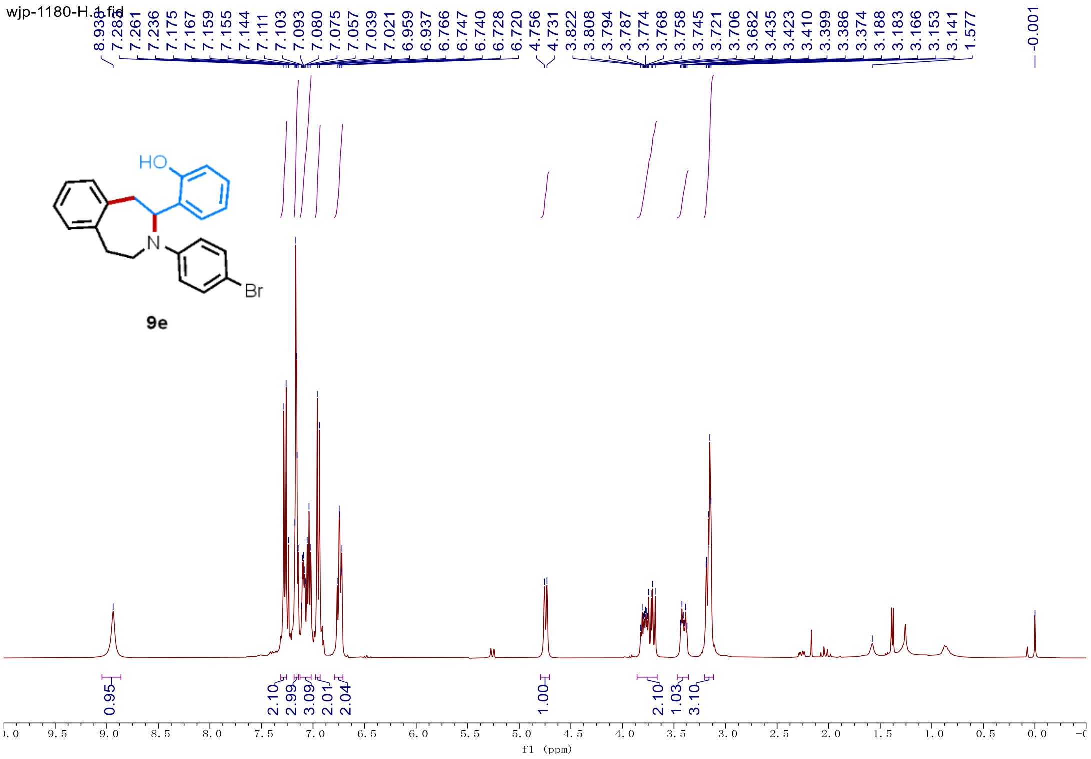

Supplementary Figure 198. ^{1}**H NMR (400 MHz, CDCl$_3$) spectrum of 9d.**

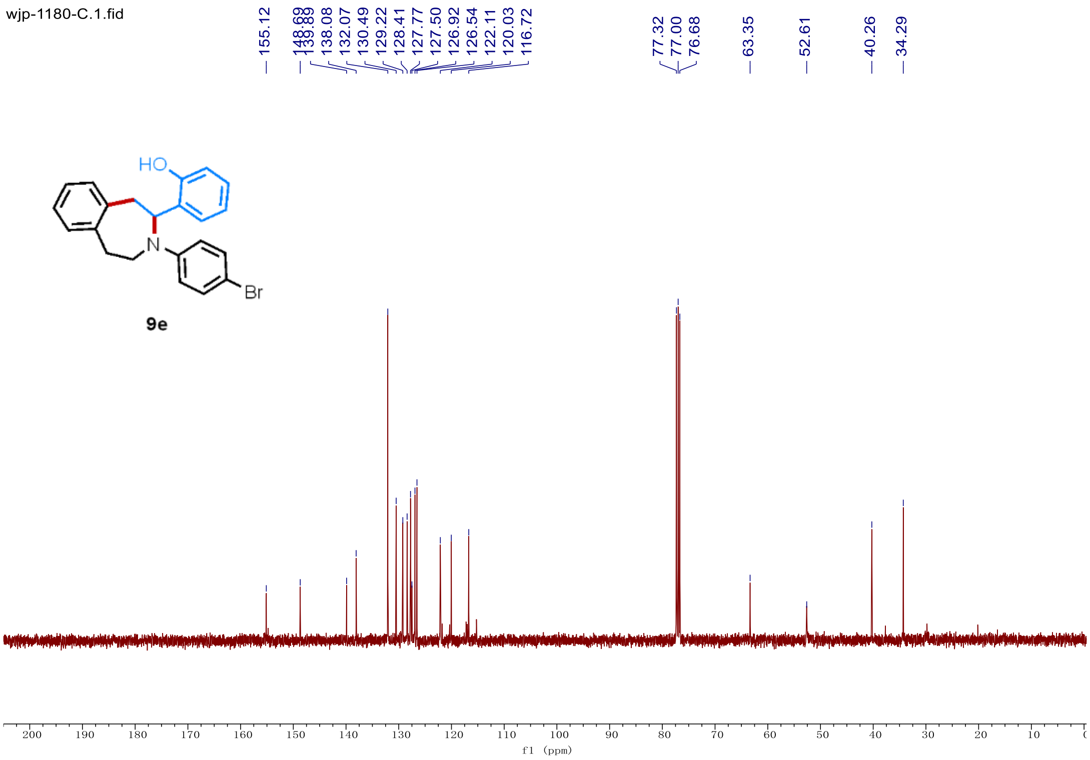

Supplementary Figure 199. ^{13}**C NMR (100 MHz, CDCl$_3$) spectrum of 9d.**

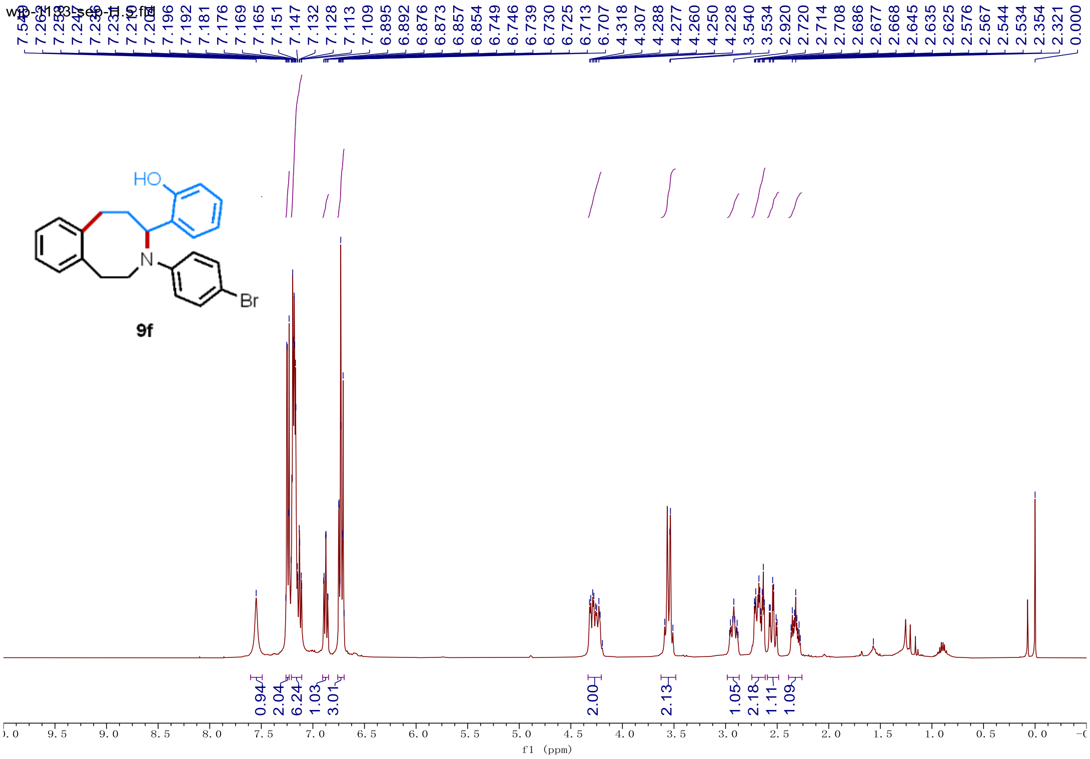

Supplementary Figure 200. ^{1}**H NMR (400 MHz, CDCl$_3$) spectrum of 9e.**

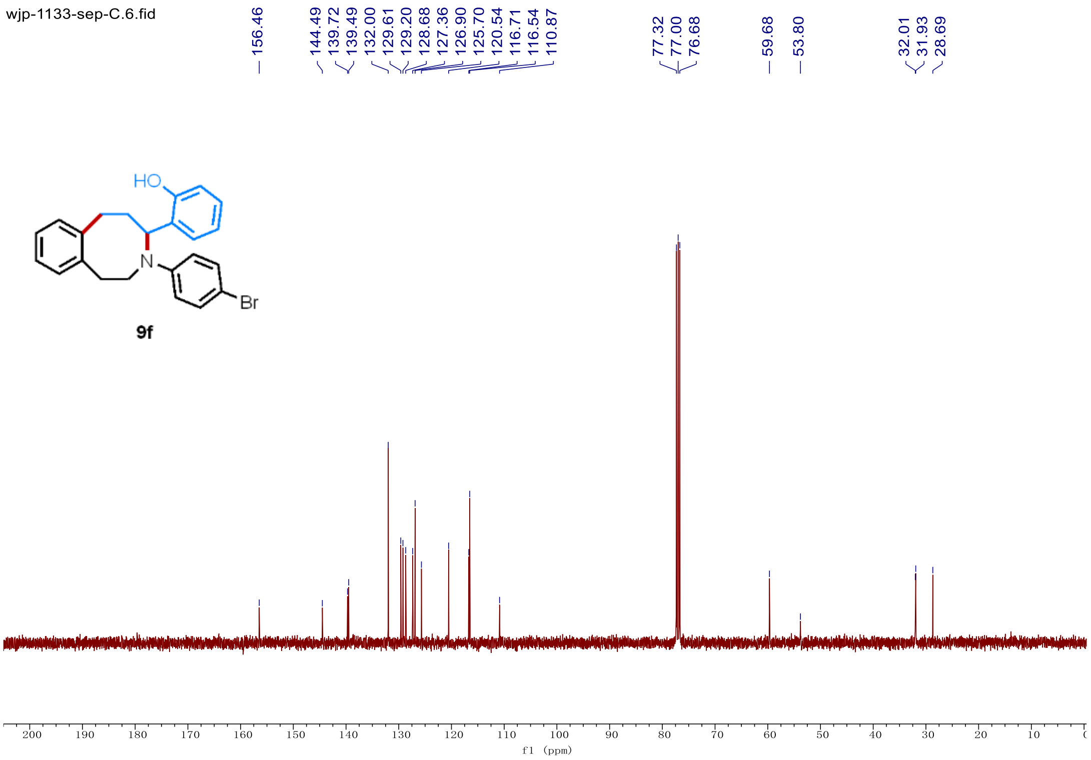

Supplementary Figure 201. ^{13}C **NMR (100 MHz,** $CDCl_3$**) spectrum of 9e.**

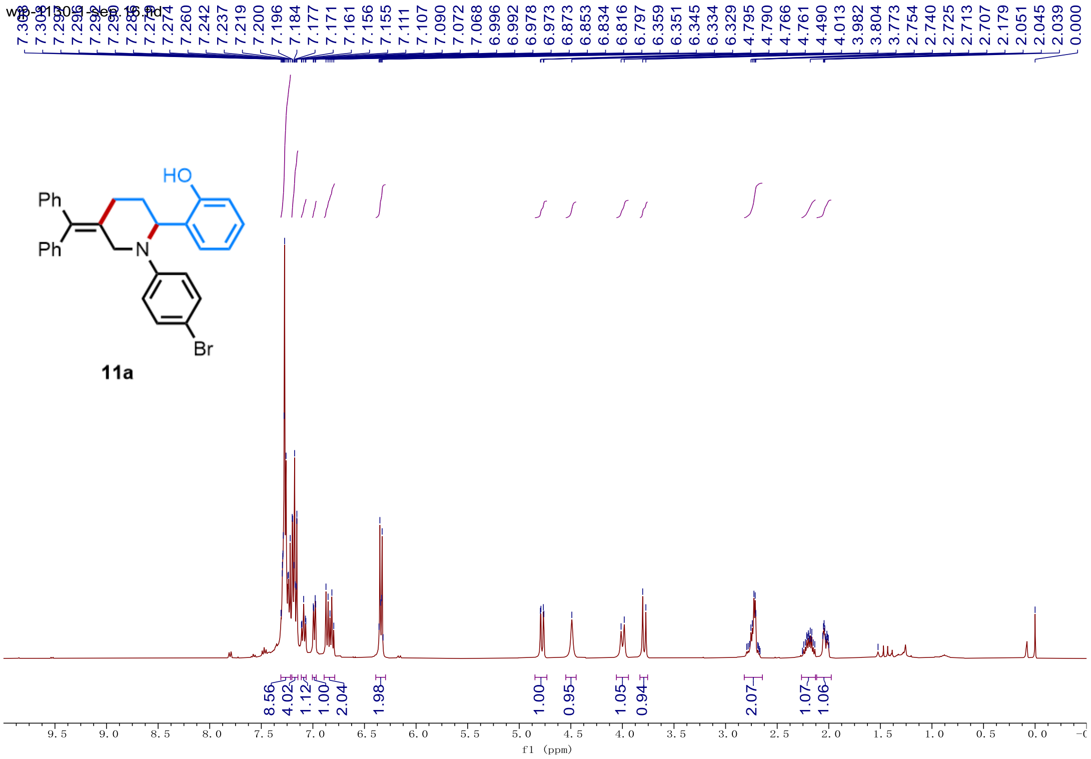

Supplementary Figure 202. ^{1}H **NMR (400 MHz,** $CDCl_3$**) spectrum of 11a.**

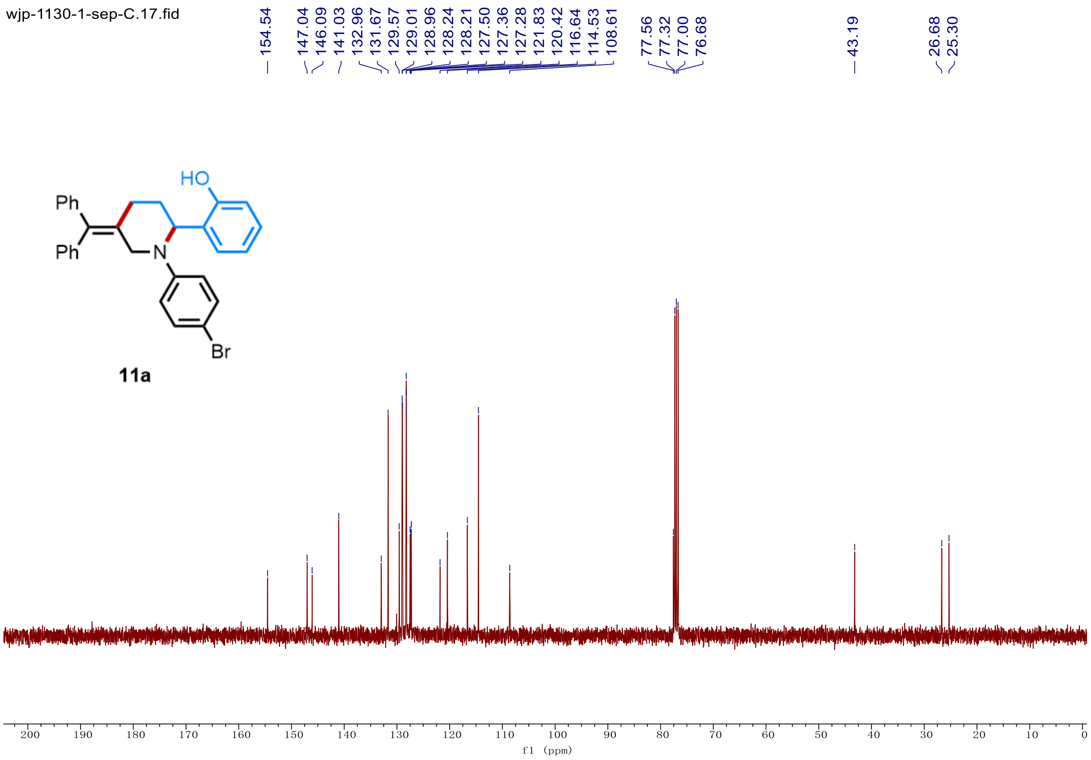

11a

Supplementary Figure 203. ^{13}C NMR (100 MHz, $CDCl_3$) spectrum of 11a.

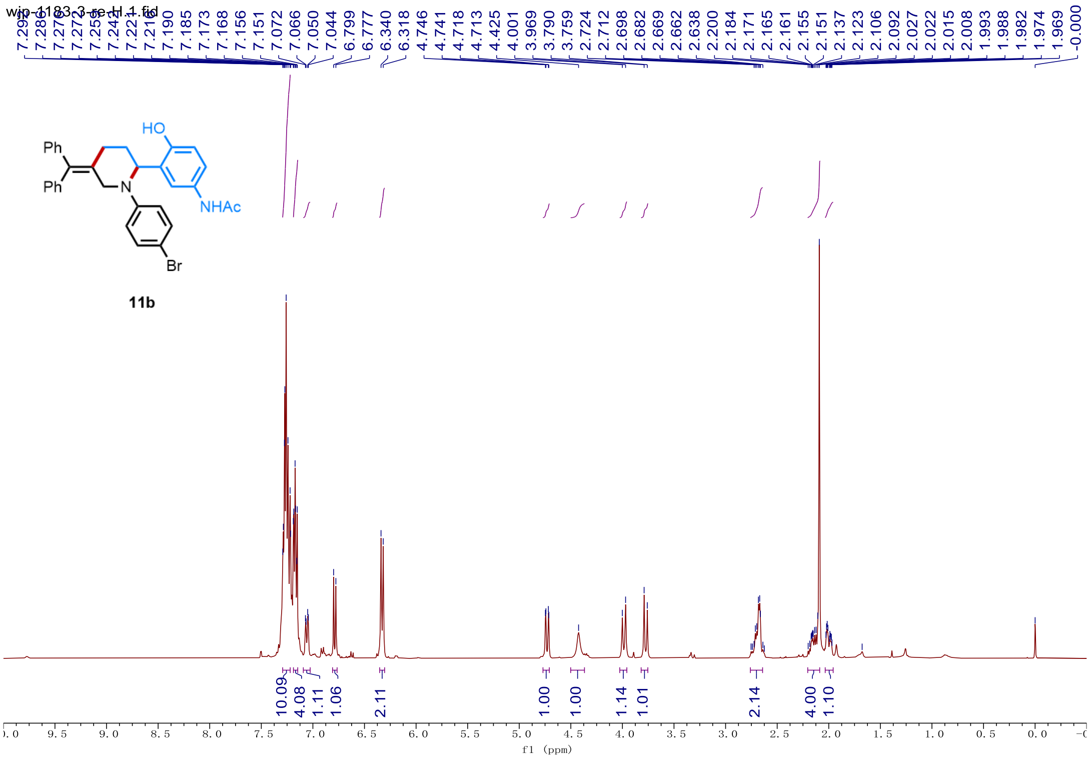

11b

Supplementary Figure 204. ^{1}H NMR (400 MHz, $CDCl_3$) spectrum of 11b.

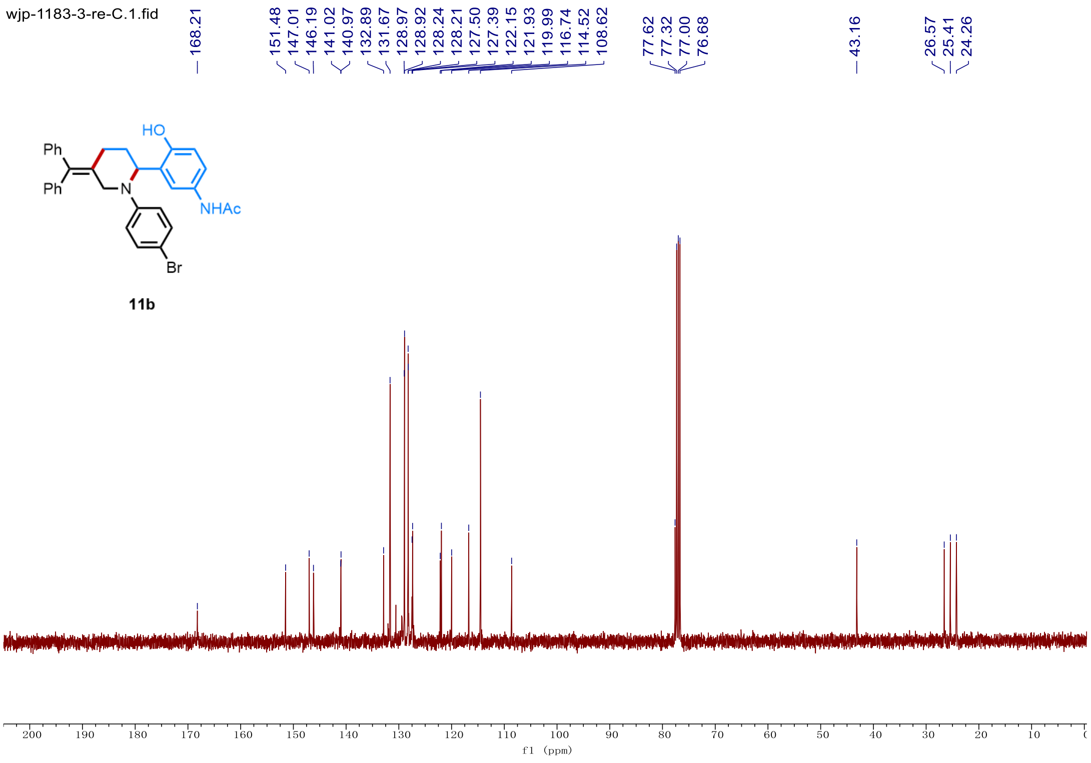

Supplementary Figure 205. ^{13}C NMR (100 MHz, $CDCl_3$) spectrum of 11b.

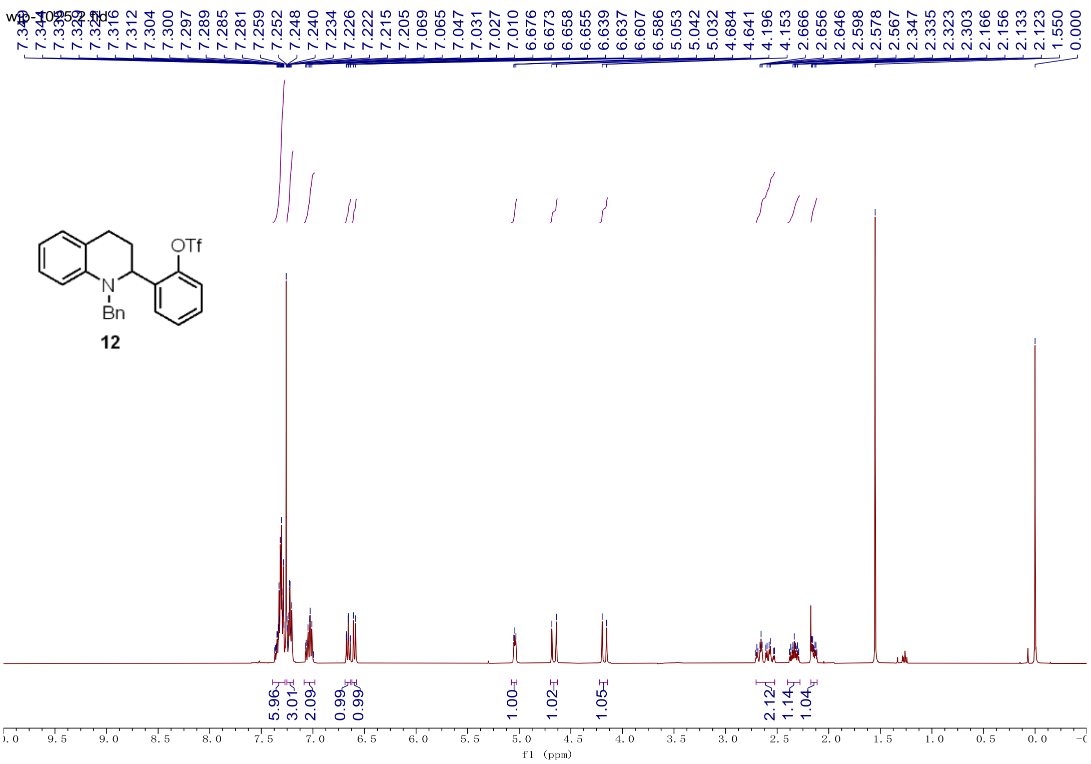

Supplementary Figure 206. ^{1}H NMR (400 MHz, $CDCl_3$) spectrum of 12.

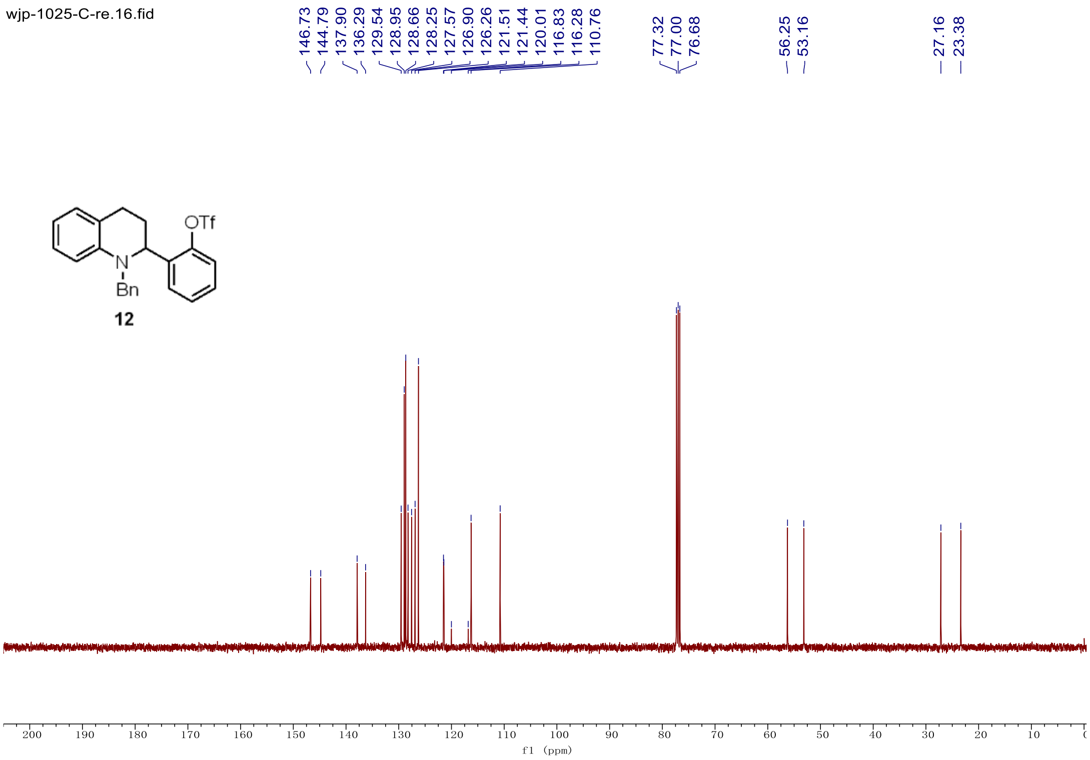

Supplementary Figure 207. ^{13}**C NMR (100 MHz, CDCl$_3$) spectrum of 12.**

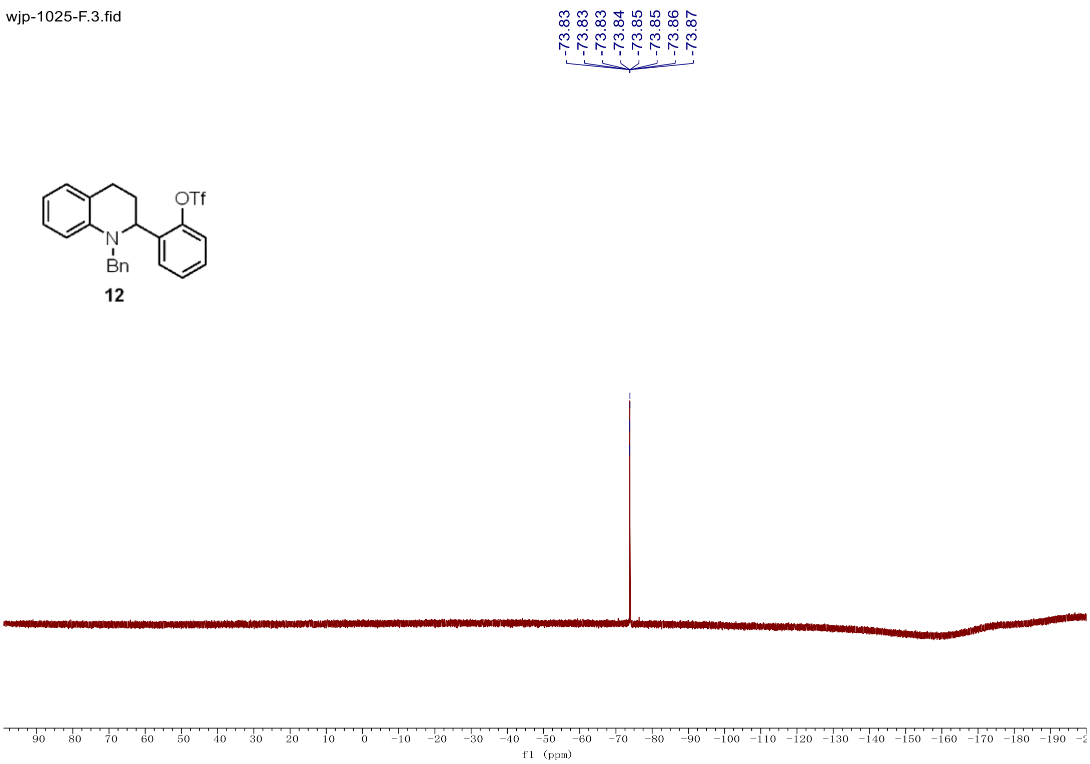

Supplementary Figure 208. ^{19}**F NMR (375 MHz, CDCl$_3$) spectrum of 12.**

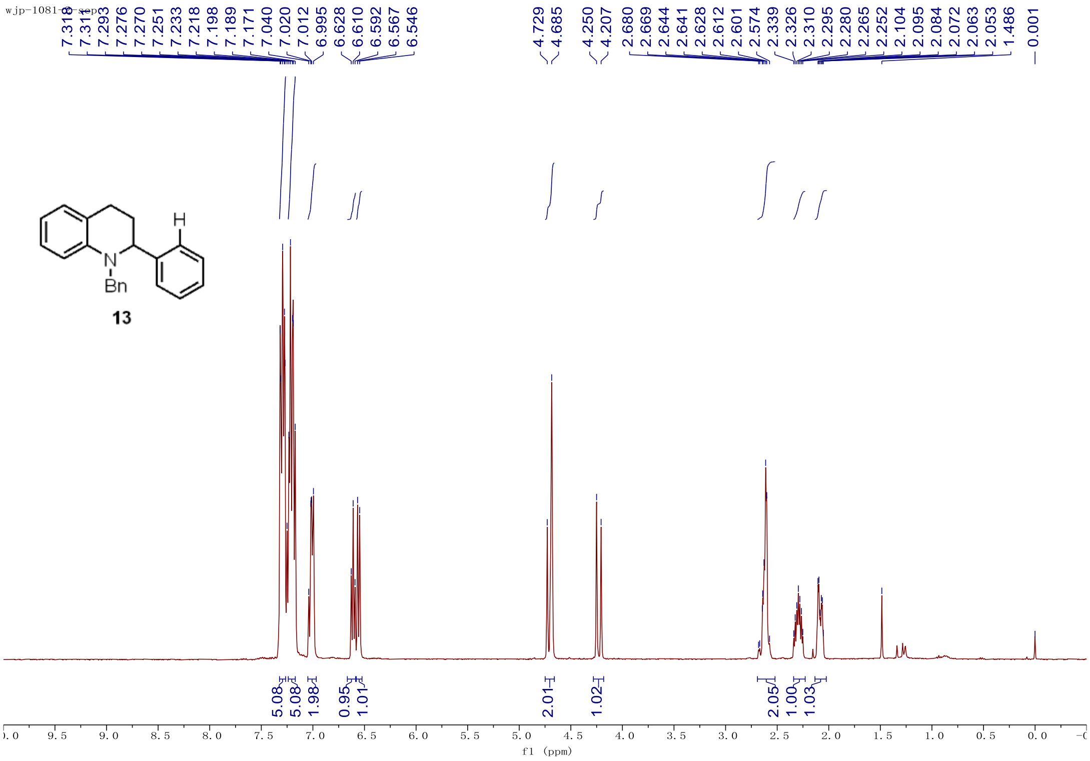

Supplementary Figure 209. ^{1}H NMR (400 MHz, CDCl$_3$) spectrum of 13.

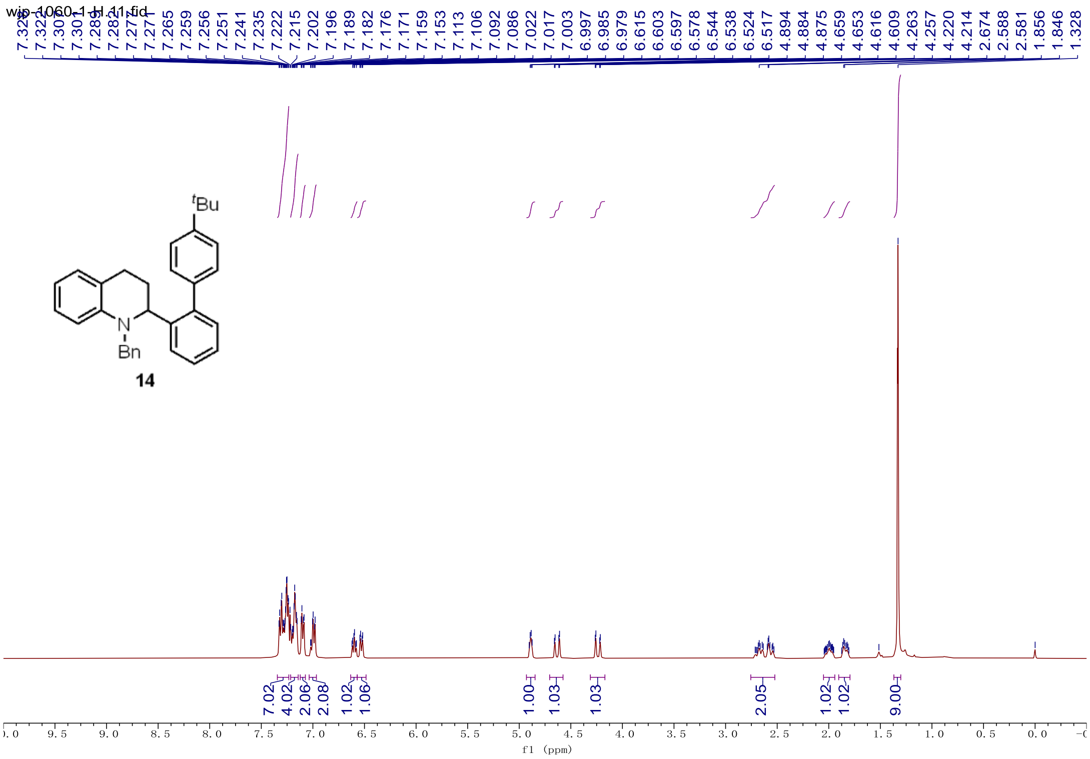

Supplementary Figure 210. ^{1}H NMR (400 MHz, CDCl$_3$) spectrum of 14.

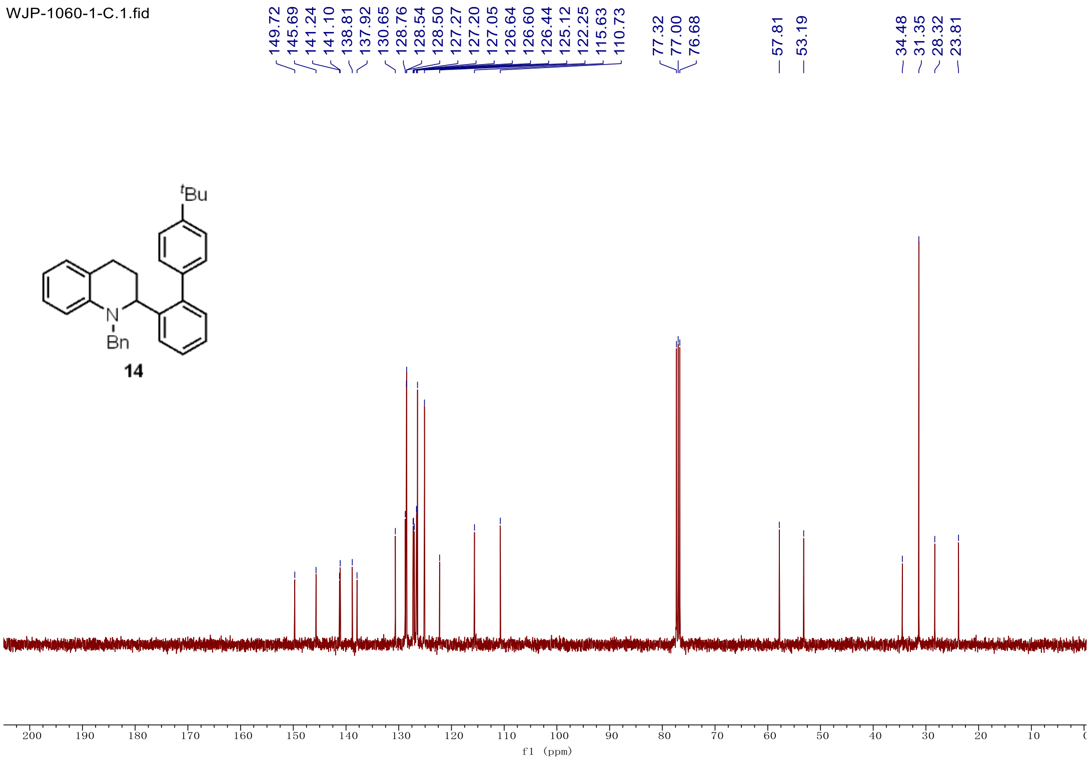

Supplementary Figure 211. ^{13}**C NMR (100 MHz, CDCl**$_3$**) spectrum of 14.**

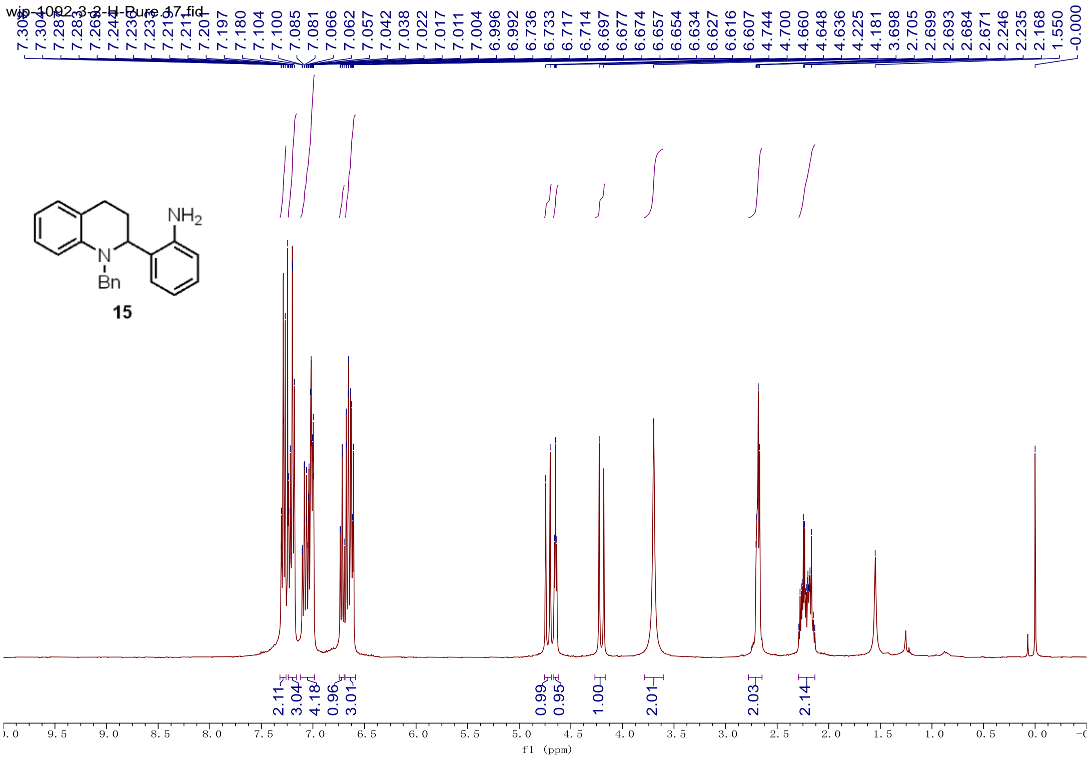

Supplementary Figure 212. ^{1}**H NMR (400 MHz, CDCl**$_3$**) spectrum of 15.**

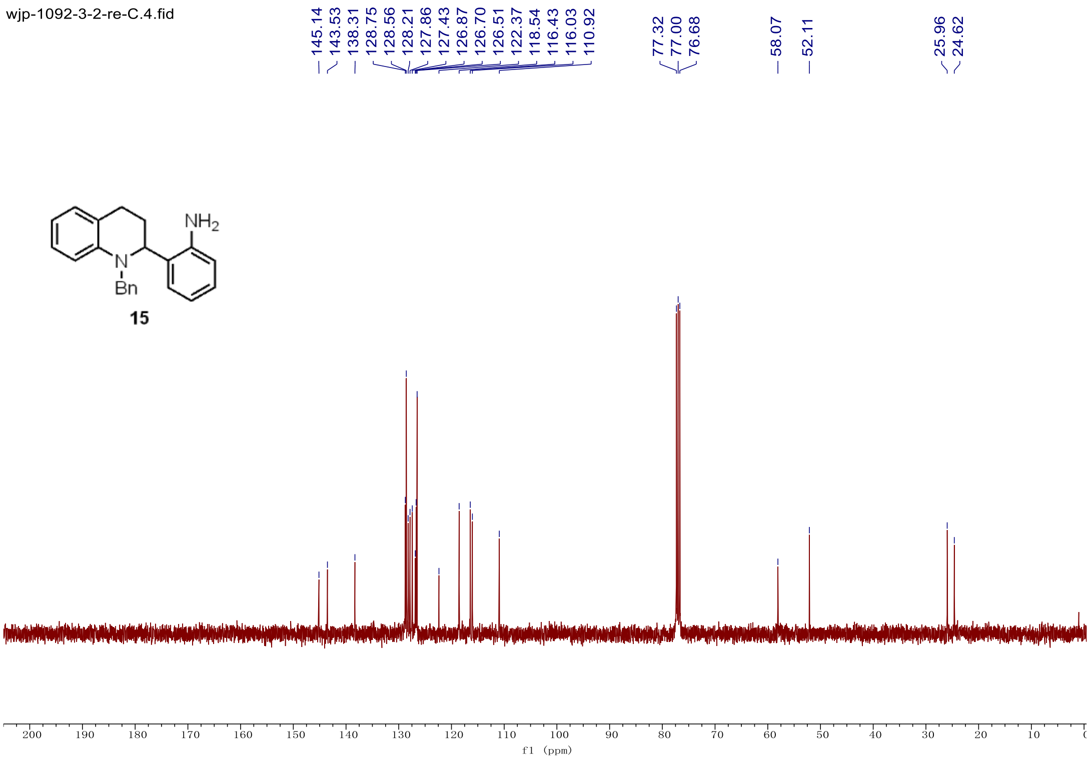

Supplementary Figure 213. ^{13}C NMR (100 MHz, CDCl$_3$) spectrum of 15.

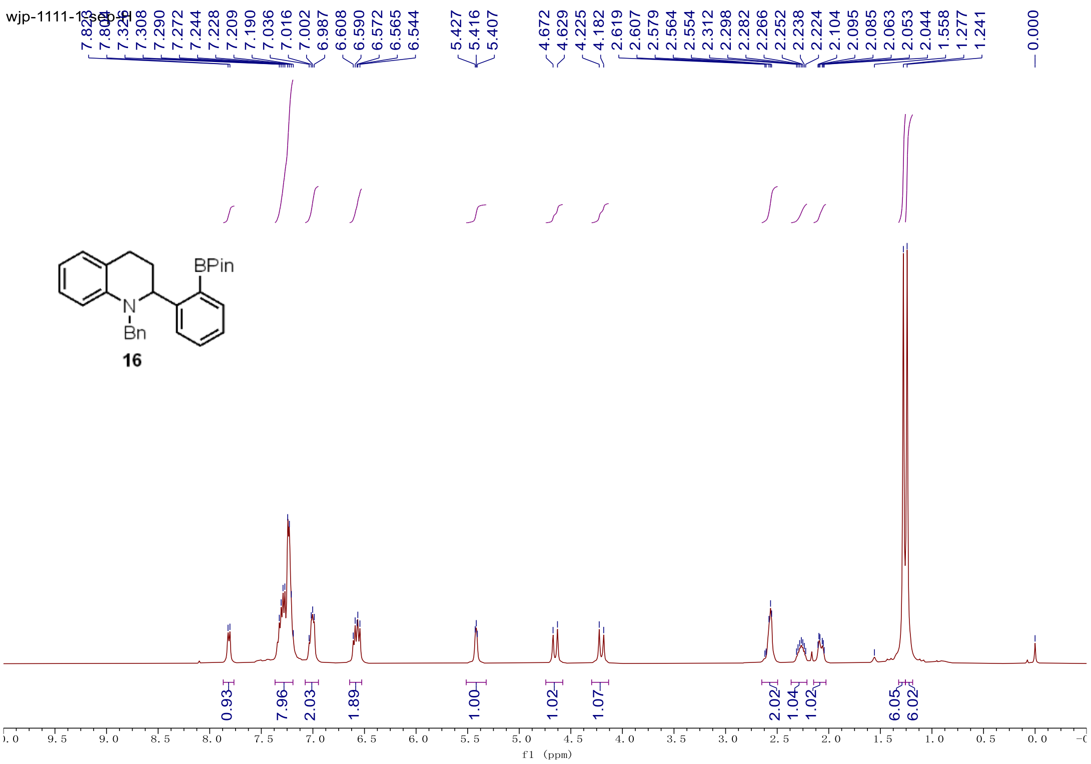

Supplementary Figure 214. ^{1}H NMR (400 MHz, CDCl$_3$) spectrum of 16.

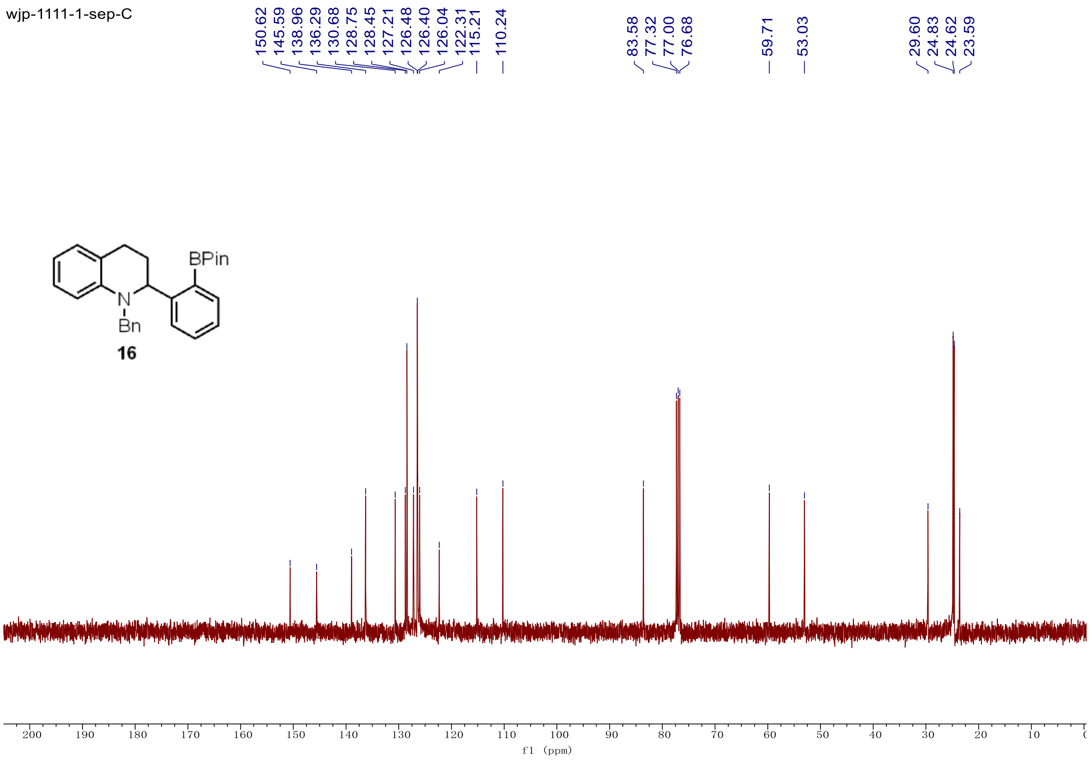

Supplementary Figure 215. ^{13}C NMR (100 MHz, CDCl$_3$) spectrum of 16.

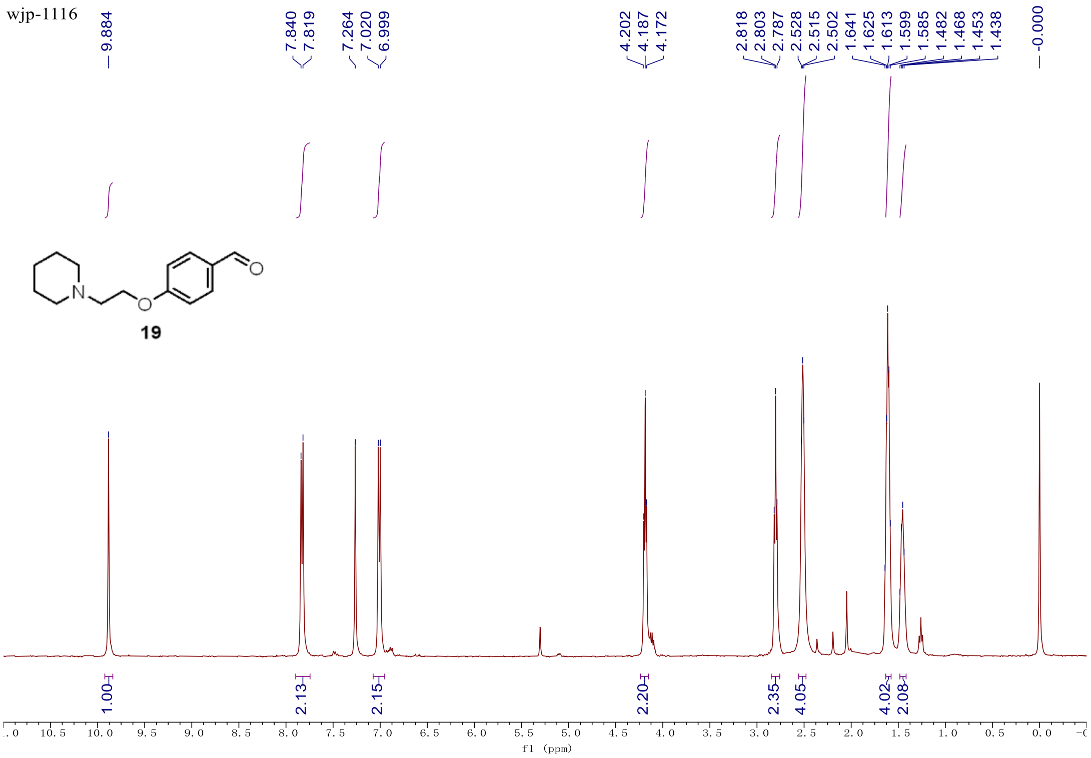

Supplementary Figure 216. ^{1}H NMR (400 MHz, CDCl$_3$) spectrum of 19.

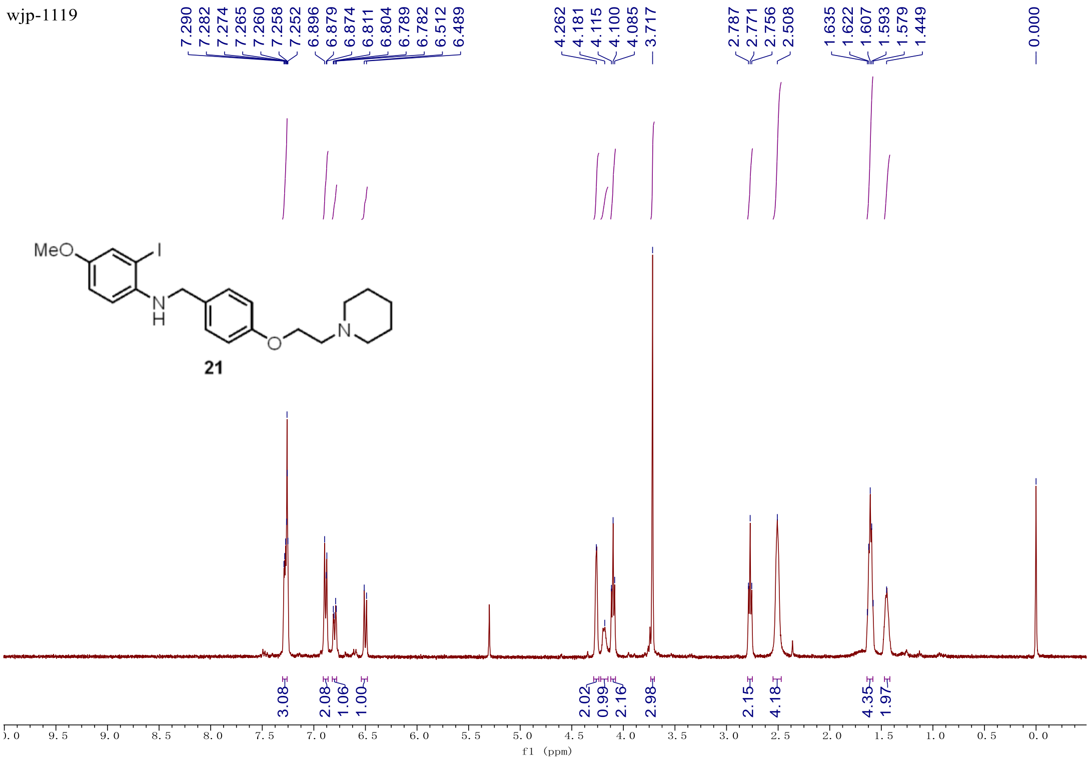

Supplementary Figure 217. ^{1}**H NMR (400 MHz, CDCl$_3$) spectrum of 21.**

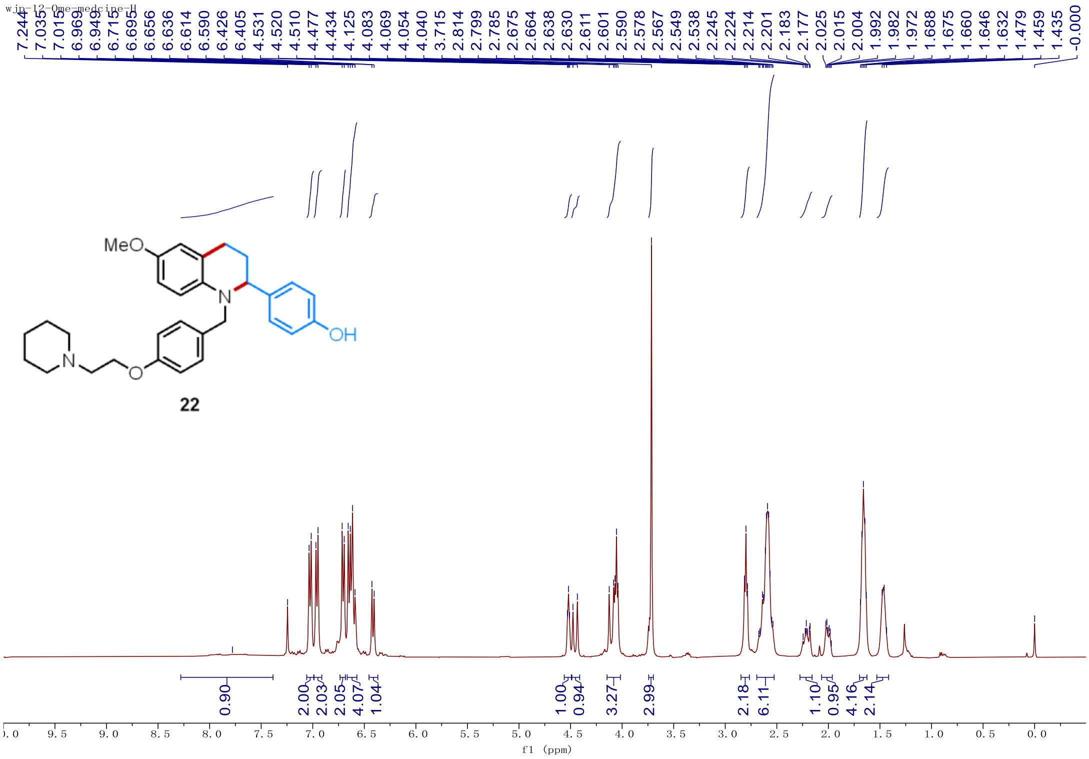

Supplementary Figure 218. ^{1}**H NMR (400 MHz, CDCl$_3$) spectrum of 22.**

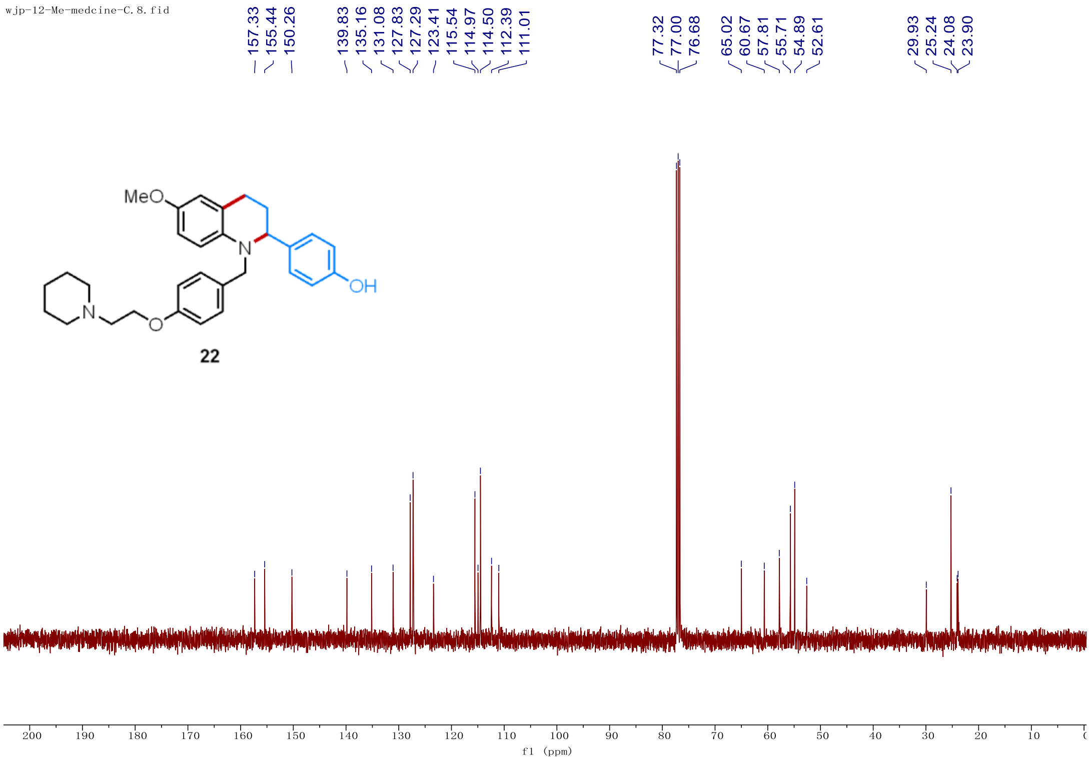

Supplementary Figure 219. ^{13}C NMR (100 MHz, $CDCl_3$) spectrum of 22.

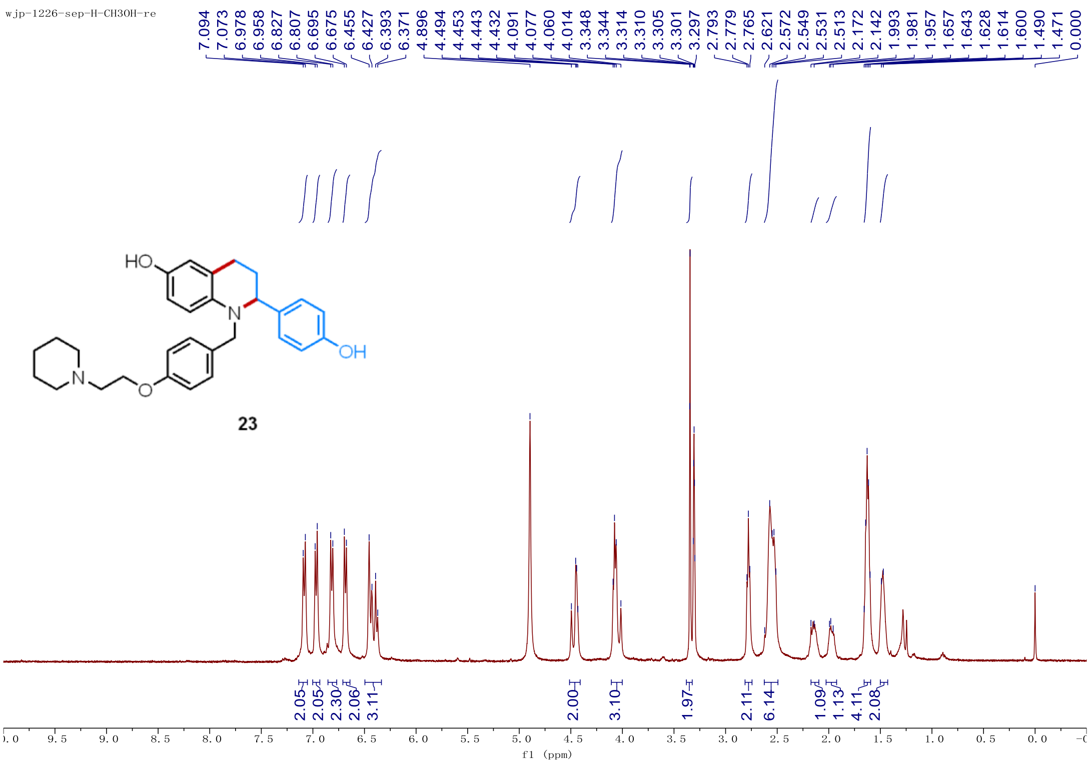

Supplementary Figure 220. ^{1}H NMR (400 MHz, CD_3OD) spectrum of 23.

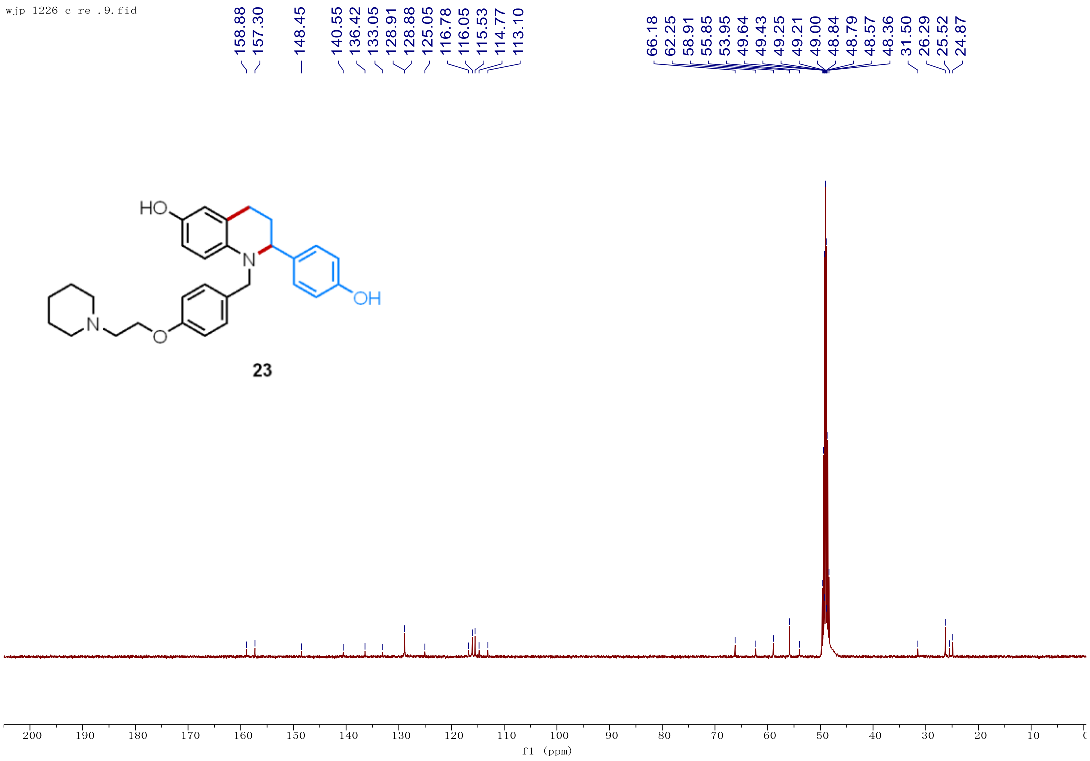

Supplementary Figure 221. ^{13}C NMR (100 MHz, CD_3OD) spectrum of 23.

6. Supplementary References

1. Laursen, S. R., Jensen, M. T., Lindhardt, A. T., Jacobsen, M. F. & Skrydstrup T. A palladium-catalyzed double carbonylation approach to isatins from 2-iodoanilines. *Eur. J. Org. Chem*. **10**, 1881–1885 (2016).
2. Le, C. M. *et al.* Stereoselective synthesis of methylene oxindoles via palladium(II)-catalyzed intramolecular cross-coupling of carbamoyl chlorides. *J. Am. Chem. Soc*. **138**, 14441–14448 (2016).
3. Chung, H., Kim, J., González-Montiel, G. A., Cheong, P. H. & Lee, H. G. Modular counter-Fischer–indole synthesis through radical-enolate coupling. *Org. Lett*. **23**, 1096–1102 (2021).
4. Hiroya, K., Itoh, S. & Sakamoto, T. Development of an efficient procedure for indole ring synthesis from 2-ethynylaniline derivatives catalyzed by Cu(II) salts and its application to natural product synthesis *J. Org. Chem*. **69**, 1126–1136 (2004).
5. Le, C. M., Hou, X., Sperger, T., Schoenebeck, F. & Lautens, M. An exclusively *trans*-selective chlorocarbamoylation of alkynes enabledby a palladium/phosphaadamantane catalyst. *Angew. Chem. Int. Ed*. **54**, 15897–15900 (2015).
6. Shen, R., Kusakabe, T., Takahashi, K. & Kato, K. Pd(II)-catalyzed ligand controlled synthesis of methyl 1-benzyl-1H-indole-3-carboxylates and bis(1-benzyl-1H-indol-3-yl)methanones. *Org. Biomol. Chem.* **12**, 4602–4609 (2014).
7. Zhang, B.-S. *et al.* Palladium-catalyzed synthesis of tricyclic indoles via a N–S Bond cleavage strategy. *Org. Lett*. **23**, 7518–7523 (2021).
8. Zhao Y., Foo, S. W., & Saito, S. Iron/Amino acidcatalyzed direct *N*-alkylation of amines with alcohols. *Angew. Chem. Int. Ed.* **50**, 3006–3009 (2011).
9. Bhakuni, B. S. *et al*. KO^{t}Bu mediated synthesis of phenanthridinones and dibenzoazepinones. *Org. Lett.* **14**, 2838–2841 (2012).
10. Yoshida, M., Higuchi, M. & Shishido, K. Stereoselective construction of substituted chromans by palladium-catalyzed cyclization of propargylic carbonates with 2-(2-hydroxyphenyl)acetates. *Org. Lett.* **11**, 4752–4755 (2009).
11. Rehan, M., Nallagonda, R., Das, B. G., Meena, T. & Ghorai, P. Synthesis of functionalized benzo[*b*]furans via oxidative cyclization of *o*-cinnamyl phenols. *J. Org. Chem*. **82**, 3411−3424 (2017).
12. Hemelaere, R., Carreaux, F. & Carboni, B. A diastereoselective route to trans-2-Aryl-2,3-dihydrobenzofurans through sequential cross-metathesis/isomerization/allylboration reactions: synthesis of bioactive neolignans. *Eur. J. Org. Chem*. **11**, 2470–2481 (2015).
13. Yokozawa, T., Suzuki, Y. & Hiraoka, S. Aromatic polyethers with low polydispersities from chain-growth polycondensation. *J. Am. Chem. Soc*. **123**, 9902–9903 (2001).
14. Kang, H. *et al.* Asymmetric oxidative coupling of phenols and hydroxycarbazoles. *Org. Lett*. **19**, 5505−5508

(2017).

15. Oleynik, A. S. *et al.* Synthesis and antioxidant properties of sodium S-[3-(hydroxyaryl)propyl] thiosulfates and [3-(hydroxyaryl)propane]-1-sulfonates. *Russ. Chem. Bull.* **56**, 1135–1143 (2007).
16. Peter, Y., Tadas S. M. & Wessely, T. Oxidation and intramolecular Diels–Alder reactions. III. synthesis of isotwistanes. *Can. J. Chem*. **66**, 1–10 (1988).
17. Kaga, A. *et al*. Nucleophilic amination of methoxy arenes promoted by a sodium hydride/iodide composite. *Angew. Chem. Int. Ed.* **56**, 11807–11811 (2017).
